# Design Rules for Sb and Bi Porphyrin Capsules: Para-Substitution Effects and Pnictogen Bond Conformational Control

**DOI:** 10.1021/acs.inorgchem.5c05993

**Published:** 2026-02-12

**Authors:** Daniel Cubero-Pascual, Álvaro García-Romero, Héctor Barbero, Raúl García-Rodríguez

**Affiliations:** GIR MIOMeT-IU Cinquima-Química Inorgánica Facultad de Ciencias, 16782 Universidad de Valladolid, Campus Miguel Delibes, 47011 Valladolid, Spain

## Abstract

Herein, we investigate the interplay between the heavy pnictogen bridgehead atom (E) in the tris­(3-pyridyl) linkers E­(3-py)_3_ (E = Sb (**1**), Bi (**2**)), and meso-aryl substituents on the metalloporphyrin scaffolds MTPPX (M = Zn, Mg; TPPX = substituted tetraphenylporphyrin) with respect to capsule formation and conformational control. Coordination of **1** and **2** to para-substituted zinc porphyrins ZnTPPOMe and ZnTPPBr yielded partially encapsulated semicapsules {[E­(3-py)_3_]·(ZnTPPX)_2_}, while MgTPPBr produced oligomeric structures, showing that relatively bulky para-substituents disfavor complete 1:3 capsule formation. In contrast, coordination of **1** and **2** to perfluorinated ZnTPPF_5_ promotes the formation of full 1:3 capsules {[E­(3-py)_3_]·(ZnTPPF_5_)_3_}, stabilized by three intramolecular E···F pnictogen bonds (PnBs) that give rise to a unique “blocked” conformation. DFT calculations indicate that distal porphyrin coordination enhances Lewis acidity at E, deepening its σ-holes and strengthening E···F interactions, thus overcoming the negative cooperativity typically associated with multiple PnBs. This remote coordination effect offers a new supramolecular strategy to fine-tune σ-hole depth and Lewis acidity. The steric shielding of the bridgehead in this conformation markedly affects reactivity, as shown by the inhibition of Sb-catalyzed α-hydroxyketone oxidation. These studies illustrate the crucial role of PnBs in stabilizing capsules of this type and modulating their reactivity through conformational control.

## Graphical abstract

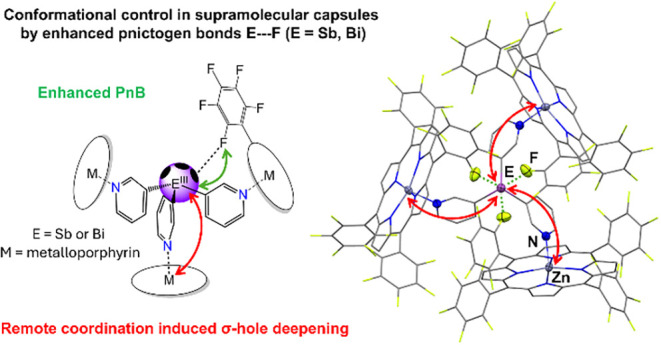

## Introduction

Supramolecular capsules, like enzyme binding pockets, provide confined spaces that can promote reaction outcomes inaccessible by conventional approaches.^1–4^ A variety of capsule-like structures with well-defined cavities reminiscent of the enzymatic active site have been designed,^5–8^ finding applications in drug delivery,^9–11^ gas storage^12–16^ and as nanoreactors^17–21^ for catalytic and stoichiometric transformations. Confinement within the cavity can enhance the reactivity or selectivity of guest molecules as well as stabilize otherwise reactive species.^22–24^ As in natural enzymes, the performance of these systems depends crucially on the shape, size, and microenvironment of their cavities.^2,6,25^ Therefore, understanding the factors that govern capsule formation and control their conformation is essential for the rational design and optimization of systems with tailored activity and selectivity.

One important family of capsules are the supramolecular assemblies based on the coordination of P­(3-py)_3_ to zinc­(II) porphyrins.^21,26,27^ In 2001, Reek and collaborators developed the first example, consisting of a tris­(3-pyridyl)­phosphine ligand coordinated to three zinc tetraphenylporphyrin (ZnTPPH) units, {[P­(3-py)_3_]·(ZnTPPH)_3_} (Figure 1a).^28^ Its X-ray diffraction structure, which was finally elucidated in 2013, revealed that weak peripheral CH−π interactions are important in maintaining the structure of {[P­(3-py)_3_]·(ZnTPPH)_3_}.^29^ The confined microenvironment around the bridgehead P allows encapsulation of transition-metal catalysts such as Rh and Pd, leading to accelerated rates and unprecedented selectivities in hydroformylation reactions, not achievable with unencapsulated catalysts.^30–35^ Further studies with substituted porphyrins indicated that small distortions in the capsule shape had a critical impact on the catalytic selectivity.^29,30,34,36,37^

**Figure 1:**
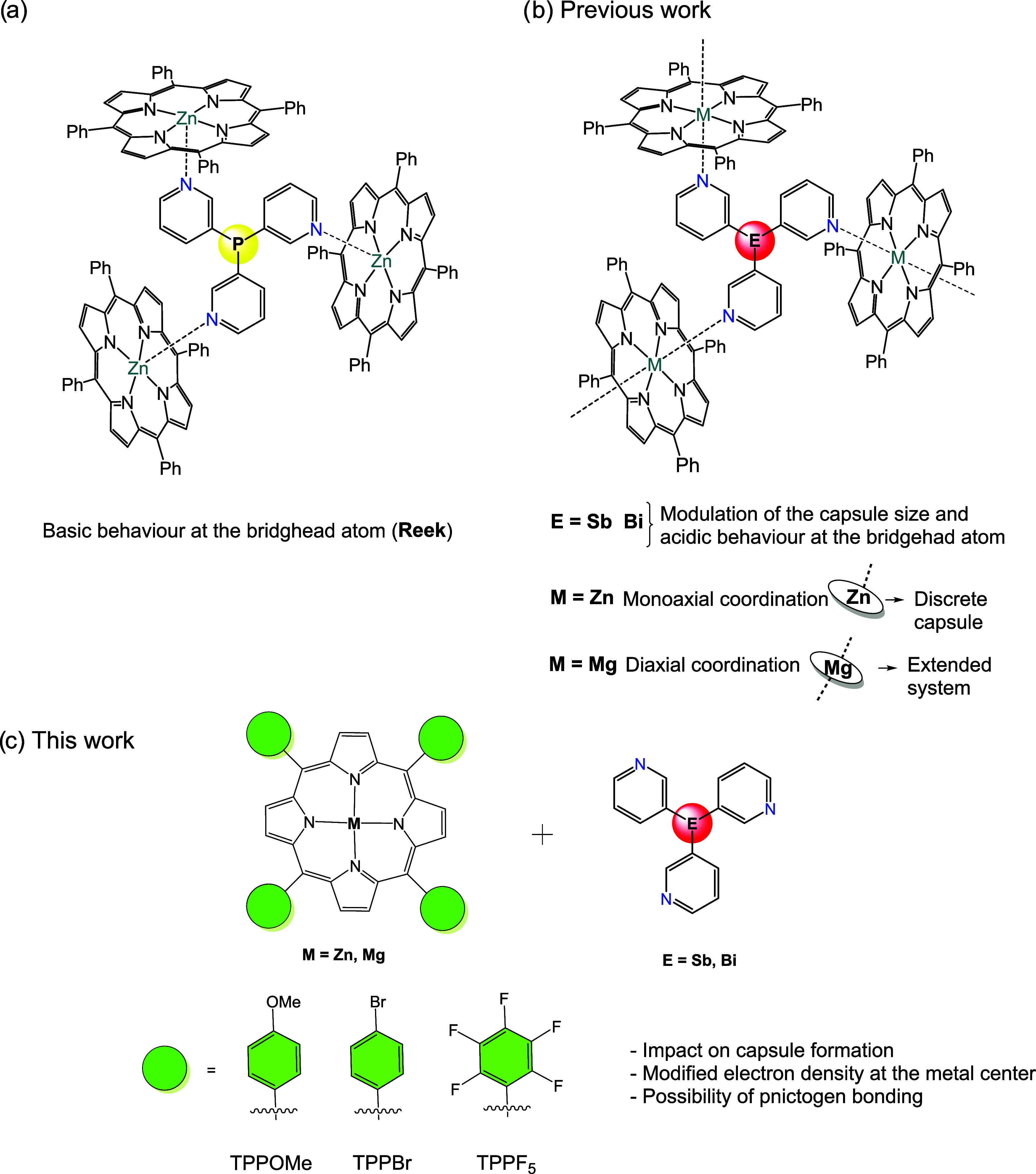
(a) First example of a {[P­(3-py)_3_]**·**(ZnTPPH)_3_} capsule. (b) Heavier group 15 bridgehead-based capsules {[E­(3-py)_3_]**·**(MTPPH)_3_} (E = Sb, Bi; M = Zn, Mg) and the formation of discrete (Zn) and extended structures (Mg) (c) current work: Effect of porphyrin substitution on Sb and Bi capsule formation and conformational control.

Despite the extensive developments in capsule design, the incorporation of heavy p-block elements as structural or functional components is very limited. In particular, supramolecular systems incorporating the heavier Group 15 elements Sb and Bi remain almost unexplored, despite the opportunities offered by their greater size, metallic character, and Lewis acidity as compared to their lighter group 15 counterparts.^38–40^ As part of our interest in heavier tris-pyridyl ligands,^41–45^ we have recently prepared the first heavier group 15 ligands E­(3-py)_3_ (E = Sb (**1**) and E = Bi (**2**))^46^ and demonstrated their ability to form discrete {[E­(3-py)_3_]·(ZnTPPH)_3_} capsules or extended {[E­(3-py)_3_]·(MgTPPH)_3_} frameworks (Figure 1b).^47^ Varying the bridgehead atom (E) in the tris­(3-pyridyl) linker effectively modulated the size of the capsule, with a linear increase in capsule dimensions relative to the lighter analogue {[P­(3-py)_3_]**·**(ZnTPPH)_3_} as group 15 is descended. Moreover, encapsulation of these heavier pnictogen centers modulated their catalytic activity, influencing the rate and selectivity in the oxidative cleavage of diols and the oxidation of α-hydroxyketones.

Changing the substitution pattern of the porphyrin in these heavier based systems (Figure 1c) could allow modulation of the shape and stability of the resulting assemblies by enhancing or disrupting weak interactions. In this context, the use of antimony and bismuth offer unique opportunities with respect to their lighter counterparts due to their higher Lewis acidity and ability to function as pnictogen bond (PnB) donors,^48^ both of which increase down group 15.^49,50^ Growing recognition of this has recently spurred the application of Sb and Bi compounds in catalysis, anion recognition and anion transport mediated by pnictogen bonding.^51–58^ PnB formation is usually correlated with the presence of regions of positive electrostatic potential, namely, σ-holes, located on the extension of covalent bonds.^48^ Trivalent stibines and bismuthines can act as PnB donors due to their low-lying σ* orbitals and their three associated σ-holes (Figure 2a). Previous strategies to deepen σ-holes and enhance the pnictogen-bond donor ability (or Lewis acidity) of Sb­(III) and Bi­(III) include the incorporation of electron-withdrawing substituents into the ligand framework^57,59^ (Figure 2a, covalent approach) or direct modification of the pnictogen atom via oxidation to the +V state^60^ (Figure 2b, oxidation approach). Although direct coordination at the pnictogen atom (Figure 2c) may increase Lewis acidity,^49,61^ it often introduces steric congestion, reducing σ-hole accessibility. Moreover, because Sb­(III) and Bi­(III) are relatively poor donors, such coordination is typically weak and can require polydentate ligands with auxiliary donor ligands, which can lead to coordination noninnocence, as illustrated by Gabbaï.^62–64^ In both the oxidation and direct coordination approaches, steric crowding around the pnictogen center limits the formation of multiple PnBs.

**Figure 2:**
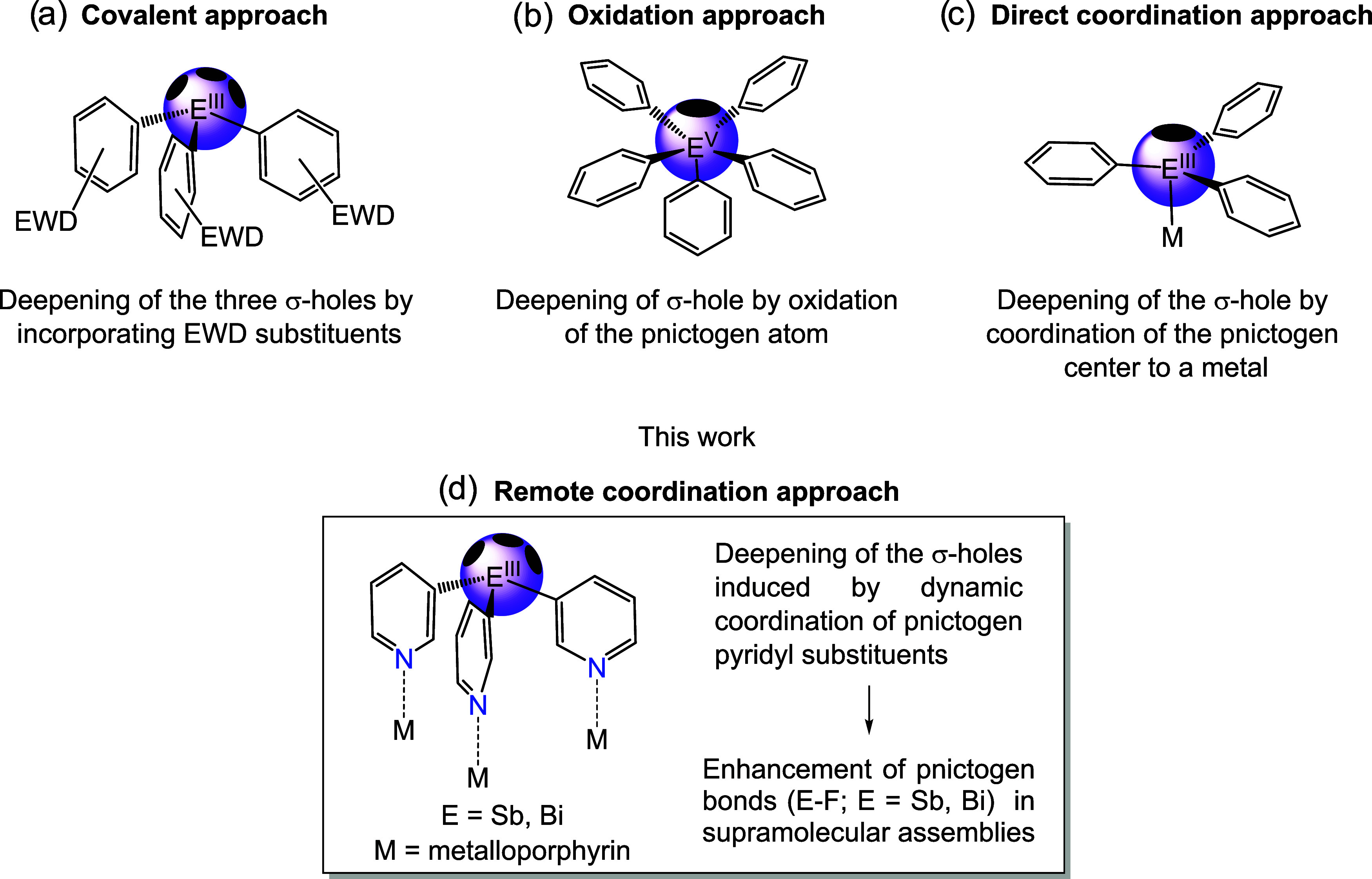
Previous strategies for σ-hole deepening (stabilization of the σ* orbital) and increasing Lewis acidity on pnictogen atoms: (a) covalent approach; (b) oxidation approach; (c) direct coordination approach. EWD = electron-withdrawing substituent. (d) Remote coordination approach in which σ-hole depth and Lewis acidity are modulated through polymetallic coordination to metalloporphyrins.

We wondered if distal porphyrin coordination could be used to modulate the Lewis acidity at the pnictogen bridgehead and, in turn, the structural and functional behavior of the resulting supramolecular capsules. Our previous studies were limited to the unsubstituted meso-tetraphenylporphyrins ZnTPPH and MgTPPH.^47^ In the present work, we explore how substituents on the porphyrin aryl rings (Figure 1c) influence the supramolecular organization of heavier group 15 Sb- and Bi-based assemblies and the factors governing capsule formation and the impact of pnictogen bonding. We show that the use of perfluorinated ZnTPPF_5_ affords complete 1:3 capsules that exhibit an unprecedented “blocked” conformation due to formation of three intramolecular E···F PnBs, rendering the pnictogen atom inaccessible. The remote coordination of the three porphyrins deepens the σ-hole and strengthens the E···F PnBs. This remote porphyrin coordination represents a new strategy (Figure 2d) to modulate the Lewis acidity and pnictogen-bond donor ability of the trivalent center by exploiting the polymetallic coordination capacity of the E­(3-Py)_3_ (E = Sb, Bi) ligands. In contrast, relatively bulky para-substituted porphyrins (Br or OMe) yield only semicapsules (1:2 ligand/porphyrin stoichiometry) instead of the anticipated 1:3 capsules. These findings highlight pnictogen bonding as a powerful and underutilized strategy for modulating the architecture and reactivity of supramolecular capsules, expanding the toolkit for precise supramolecular design.

## Results and Discussion

### Effect of Para-Substitution on Capsule Formation

To evaluate the effect of para-substitution on the aryl groups of the zinc metalloporphyrin, we studied the coordination of the ligands Sb­(3-py)_3_ (**1**) and Bi­(3-py)_3_ (**2**) with the methoxy- and bromine-substituted zinc metalloporphyrins ZnTPPOMe and ZnTPPBr, respectively (Scheme 1). First, we evaluated whether the formation of the capsules was possible when using the −OMe substituted porphyrin as a building block. Three equivalents of ZnTPPOMe were added to a solution of **1** or **2** in CDCl_3_ (Scheme 1). A large upfield shift in the ^1^H NMR signals of the pyridyl ring was observed for both ligands, which was associated with the ring current effect characteristic of axial binding of the pyridine to the Zn­(II) center of the metalloporphyrin. Slow diffusion of *n*-hexane into a CHCl_3_ solution of **1** or **2** (1 equiv) and ZnTPPOMe (3 equiv) gave purple crystals suitable for X-ray diffraction in both cases; however, to our surprise, the products did not exhibit the expected 1:3 supramolecular capsule structure {[E­(3-py)_3_]**·**(ZnTPPOMe)_3_} (E = Sb, Bi). Instead, the semicapsules {[E­(3-py)_3_]**·**(ZnTPPOMe)_2_} (**1·**ZnTPPOMe and **2·**ZnTPPOMe, respectively) were obtained, in which ligand **1** or **2** coordinates only two zinc metalloporphyrins through two of its N-donor groups, while its third pyridyl arm remains uncoordinated. ^1^H NMR spectra of **1·**ZnTPPOMe and **2·**ZnTPPOMe in CDCl_3_ also indicated a 1:2 ratio of **1** or **2** to ZnTPPOMe.^65^

**Scheme 1:**
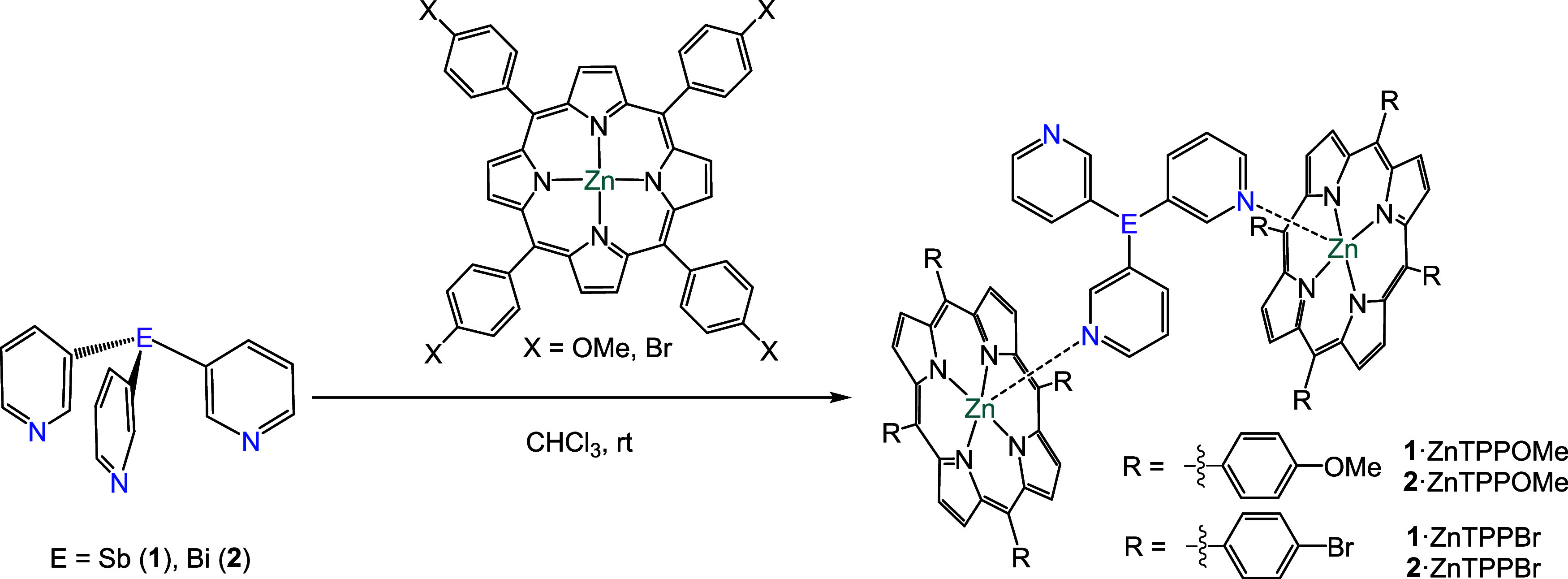
Synthesis of the Semicapsules {[Sb­(3-py)_3_]**·**(ZnTPPOMe)_2_} (**1·**ZnTPPOMe), {[Bi­(3-py)_3_]**·**(ZnTPPOMe)_2_} (**2·**ZnTPPOMe), {[Sb­(3-py)_3_]**·**(ZnTPPBr)_2_} (**1·**ZnTPPBr), and {[Bi­(3-py)_3_]**·**(ZnTPPBr)_2_} (**2·**ZnTPPBr)

Crystallographic analysis of **1·**ZnTPPOMe and **2·**ZnTPPOMe reveals that of the two coordinated arms, one has its nitrogen pointing upward, and the corresponding porphyrin is slightly above the bridgehead atom (Figure 3). The other has its nitrogen pointing downward and the corresponding porphyrin lies below the ligand, in an oblique orientation relative to the other porphyrin, with dihedral angles between the mean planes of the two porphyrins (defined by the four porphyrinic nitrogen atoms) of 51.91° and 61.43° for **1**·ZnTPPOMe and **2**·ZnTPPOMe, respectively. Overall, the solid-state structures of **1**·ZnTPPOMe and **2**·ZnTPPOMe are similar, although not isostructural. Compared to that of **1**·ZnTPPOMe, the upward-pointing pyridine arm in **2**·ZnTPPOMe is slightly tilted, and, therefore, the coordinated ZnTPPOMe unit is also rotated.

**Figure 3:**
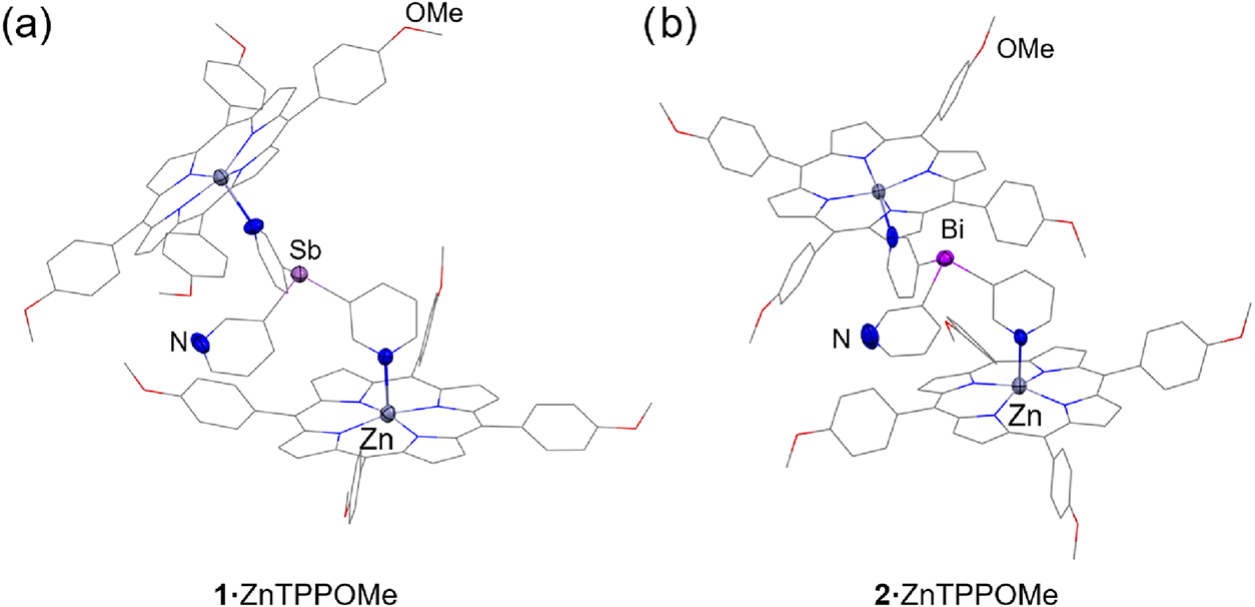
X-ray structures of the semicapsules **1**·ZnTPPOMe (a) and **2**·ZnTPPOMe (b). In both structures, the ligand coordinates two porphyrins through N–Zn interactions, while the third pyridyl arm remains uncoordinated. Displacement ellipsoids of the heteroatoms are shown at 50% probability. Solvent molecules and H atoms are omitted for clarity. Selected bond lengths (Å) and angles (deg): **1**·ZnTPPOMe, Sb–C_py_ range 2.132(5)–2.157(7); N_py_–Zn 2.180(5)–2.185(5); C_py_–Sb–C_py_ range 95.7(2)–100.2(2). **2**·ZnTPPOMe, Bi–C_py_ range 2.25(2)–2.30(1); N_py_–Zn 2.16(1); C_py_– Bi–C_py_ range 92.0(6)–96.1(5). Color key: C (gray), Zn (dark gray), N (blue), O (red), Sb (light purple), Bi (purple).

We then evaluated whether the 1:2 complexes of ligands **1** and **2** with ZnTPPOMe were also favored in solution via NMR titrations in CDCl_3_. The obtained data were systematically fitted to all potential models (1:1, 1:2, and 1:3, with four different flavors for the ternary and quaternary stoichiometries), and clearly indicated a 1:2 rather than a 1:3 stoichiometry **1·**ZnTPPOMe: *K*_1_ [(3.7 ± 0.5) × 10^3^ M^–1^], *K*_2_ [(3.0 ± 0.3) × 10^3^ M^–1^]; **2·**ZnTPPOMe: *K*_1_ [(6.5 ± 1.1) × 10^3^ M^–1^], *K*_2_ [(2.4 ± 0.3) × 10^3^ M^–1^]. Moreover, ^1^H NMR Job plot analysis also corroborates the formation of a 1:2 complex in both cases (see SI, Figures S35–S36 and S41–S44). These findings allow us to reasonably assume that the 1:2 stoichiometry is preferred in solution as well, indicating that any potential association of a third porphyrin in these heavier pnictogen systems is too weak to be observed. The ^1^H NMR signals of the semicapsules **1**·ZnTPPOMe and **2**·ZnTPPOMe exhibit broadening which could be consistent with a dynamic Zn–pyridine exchange. The size of the semicapsules **1·**ZnTPPOMe and **2·**ZnTPPOMe was evaluated through ^1^H DOSY experiments in CDCl_3_, and their calculated hydrodynamic radii (9.26 and 9.35 Å, respectively) were consistent with the crystallographic ones (see SI, Figure S27), indicating that the semicapsule arrangement retains its integrity in solution. Finally, ESI-TOF high-resolution mass-spectrometry of 1:3 mixtures of **1** or **2** and ZnTPPOMe revealed peaks corresponding to the 1:2 adducts (**1·**ZnTPPOMe [M + H]^+^: *m*/*z* 1952.4101 (calcd. 1952.4200); **2·**ZnTPPOMe [M + H]^+^: *m*/*z* 2040.4966 (calcd. 2040.4960) (see SI, Figures S60–S64)), while no evidence of the formation of 1:3 complexes was observed.

We then examined the use of ZnTPPBr to study the effect of replacing the electron-donating substituent (−OMe) at the para position of the phenyl rings of the ZnTPP building block with an electron-withdrawing group (−Br). The results were very similar to those obtained for ZnTPPOMe (Scheme 1). ^1^H NMR titration experiments were again consistent with a 1:2 model but not a 1:3 one (**1·**ZnTPPBr: *K*_1_ [(7.2 ± 1.8) × 10^3^ M^–1^], *K*_2_ [(1.9 ± 0.1) × 10^3^ M^–1^]; **2**·ZnTPPBr: *K*_1_ [(2.7 ± 0.4) × 10^3^ M^–1^], K_2_ [(2.6 ± 0.3) × 10^3^ M^–1^]). Further confirmation of 1:2 stoichiometry in solution was again provided by ^1^H NMR Job plot analysis, which revealed the formation of 1:2 complexes in both cases (see SI, Figures S37–S38 and S45–S48).^66^ Although attempts to isolate single crystals via slow diffusion of *n*-hexane into CHCl_3_ solutions of ligands **1** or **2** (1 equiv) and ZnTPPBr (3 equiv) were complicated by the presence of the free metalloporphyrin ZnTPPBr along with **1·**ZnTPPBr and **2·**ZnTPPBr, the use of the required 1:2 stoichiometry led to crystalline yields of 53 and 66% for **1·**ZnTPPBr and **2·**ZnTPPBr, respectively, and X-ray diffraction studies revealed the formation of complexes with 1:2 stoichiometry in the solid state (Figure 4). As observed for **1**·ZnTPPOMe and **2·**ZnTPPOMe, the ^1^H NMR signals of these semicapsules exhibit broadening which could be consistent with a dynamic Zn–pyridine exchange. The size of **1·**ZnTPPBr and **2·**ZnTPPBr in solution was also studied through ^1^H DOSY experiments in CDCl_3_, which indicated hydrodynamic radii of 8.93 and 8.99 Å, respectively (see SI, Figure S28). These values were very similar to those obtained for **1·**ZnTPPOMe and **2·**ZnTPPOMe and consistent with the crystallographic ones, indicating that while the formation of the supramolecular capsules is disfavored, these heavier group 15 based semicapsule arrangements are maintained in solution.

**Figure 4:**
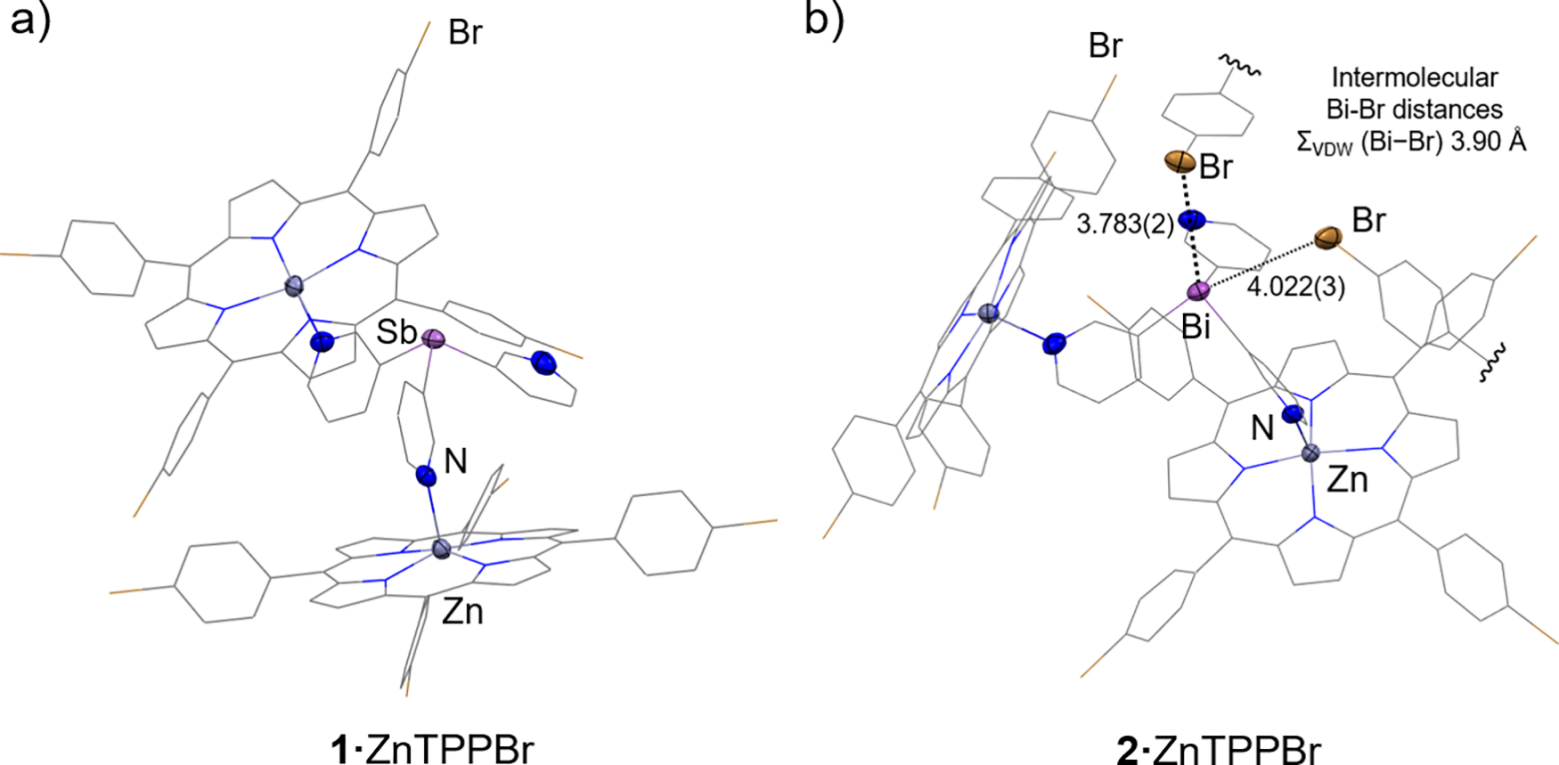
X-ray structures of the complexes **1**·ZnTPPBr (a) and the crystal packing of **2**·ZnTPPBr (b) showing that the Bi atom is flanked by two neighboring semicapsules, each directing one Br atom from an aryl group toward the bismuth center (only the relevant aryl groups are shown for clarity). The Bi···Br distances are 3.783(3) and 4.022(3) Å, *cf*. 3.90 Å for ∑_VDW_(Bi–Br), see SI (Figure S57) for similar interactions for complex **1**·ZnTPPBr. In both structures, the ligand coordinates two porphyrins through N–Zn interactions, while the third pyridyl arm remains uncoordinated, and in both structures one aryl group (which is not the one involved in the short intermolecular E–Br distances) is disordered over two orientations. This disorder, as well as H atoms and solvent molecules, is omitted for clarity (see SI for details and Figure S56). Displacement ellipsoids of the heteroatoms are shown at 50% probability. Selected bond lengths (Å) and angles (deg): **1**·ZnTPPOMe, Sb–C_py_ range 2.139(9)–2.16(1); N_py_–Zn 2.134(7)–2.161(7); C_py_–Sb–C_py_ range 96.4(4)–99.3(4). **2**·ZnTPPOMe, Bi–C_py_ range 2.25(2)–2.26(2); N_py_–Zn range 2.12(1)–2.13(1); C_py_– Bi–C_py_ range 93.1(7)–97.9(6). Color key: C (gray), Zn (dark gray), N (blue), O (red), Sb (light purple), Bi (purple).

**1**·ZnTPPBr and **2**·ZnTPPBr are isostructural in the solid state, with minor differences originating from the different geometric profiles of the ligands (Figure 4a for **1**·ZnTPPBr and SI, Figure S56, for 2·ZnTPPBr). Their arrangement results in an open semicapsule structure closely resembling that observed for their ZnTPPOMe counterparts. One coordinated arm exhibits an upward-oriented pyridyl nitrogen, positioning the corresponding ZnTPPBr unit slightly above the bridgehead atom, while the other arm displays a downward-oriented pyridyl nitrogen, placing the porphyrin unit beneath the ligand scaffold and oblique to the first porphyrin, with the two porphyrin planes forming dihedral angles of 52.27° and 52.31°, respectively. The location of the bromo substituent at the para position of the aryl group prevents its engagement in intramolecular interactions with the bridgehead atom. However, despite the steric constrains of the semicapsules, short contacts are observed between E···Br atoms of adjacent molecules, reflecting the Lewis acidity of the pnictogen centers. In **1**·ZnTPPBr, the Sb center is flanked by two neighboring semicapsules, each directing one Br atom toward the Sb, approximately trans to the Zn-coordinated E–C_py_ bonds, with Sb···Br distances of 3.854(1) and 4.115(2) Å *cf*. 3.89 Å ∑_VDW_(Sb–Br) (see SI, Figure S57). Although these distances are within or slightly beyond the sum of the van der Waals radii (the latter suggesting the absence of significant orbital overlap), shorter distances (3.783(3) and 4.022(3) Å) were observed for the bismuth semicapsule **2·**ZnTPPBr *cf*. 3.90 Å ∑_VDW_(Bi–Br) (see Figure 4b), consistent with the greater Lewis acidity of Bi­(III). For both complexes, the shorter of the two E···Br distances (E = Bi, Sb) can be identified as an intermolecular pnictogen bond, as also supported by DFT calculations (see SI, Figure S95–S102 and discussion in pages S78–S82).^48^ Similar manifestations of the Lewis acidity of the antimony and bismuth centers were observed in the packing of **1·**ZnTPPOMe and **2·**ZnTPPOMe, in which the semicapsules associated in the crystal lattice via intermolecular Sb···OMe and Bi···π pyrrole interactions (see SI, Figure S58–S59).

The inability to form capsules with TPPBr was also observed for the more-labile Mg-based assemblies. Our previous studies with aryl-unsubstituted MgTPPH revealed the formation of 2D polymeric structures {[E­(3-py)_3_]_2_ **·**(MgTPPH)_3_}_*n*_ (E = Sb, Bi), in which each ligand binds to three MgTPPH units and each MgTPPH is coordinated diaxially, retaining the structure of the capsule (Figure 1b, M = Mg). In contrast, the reaction of **1** with MgTPPBr using the same stoichiometry afforded a discrete heterobimetallic complex, {[Sb­(3-py)_3_]_2_·(MgTPPBr)_3_} (**1·**MgTPPBr). The assembly can be described as two semicapsules sharing a central MgTPPBr in which each ligand **1** coordinates only two MgTPPBr units, leaving one pyridyl arm uncoordinated [for full details, see SI (page S5), Discussion of (**1**·MgTPPBr)]. The formation of **1**·MgTPPBr shows that para substitution with −Br prevents the formation of both complete capsules and extended 2D assemblies, thereby altering the structural dimensionality.

Overall, these results indicate that independent of the heavier Group 15 ligand used (Sb or Bi) or the metal center of the porphyrin (Mg^2+^ or Zn^2+^), the presence of relatively bulky para substituents (R = Br, OMe) on the aryl groups of the porphyrin interferes with the assembly into supramolecular capsules by disfavoring the coordination of the third metalloporphyrin, most likely due to steric effects. These results align with Reek’s observations regarding the large decrease in the selectivity in olefin hydroformylation reactions carried out using P­(3-py)_3_ and the para-substituted porphyrins ZnTPPR (R = CF_3_, CH_3_, tBu, OMe). The presence of these substituents could hinder the in situ assembly of the capsule or severely distort it, preventing efficient encapsulation of the single-atom Rh catalyst and thus causing the loss of encapsulation-mediated selectivity in the catalysis^29,30^

### Effect of Perfluorinated Aryl Groups on Capsule Formation

We reasoned that the introduction of electron-withdrawing substituents at the periphery of the porphyrin could enhance the stability of the capsule by increasing the electrophilicity of the zinc atom, thus increasing the association constants and potentially enhancing the robustness of the resulting supramolecular assemblies. We explored the introduction of perfluorinated rings using the tetrakis-pentafluorophenyl analogue of ZnTPPH (ZnTPPF_5_), since the relatively small fluorine atoms (*r*_VDW_F = 1.47 Å, *cf*. *r*_VDW_Br = 1.83 Å and *r*_VDW_H = 1.10 Å)^67^ were expected to reduce steric hindrance and favor the formation of complete supramolecular capsules (i.e., 1:3 stoichiometry). Moreover, the presence of ortho-fluorine atoms near the bridgehead (E) could provide additional intramolecular stabilization through directional pnictogen···fluorine interactions. A preliminary titration of the porphyrin ZnTPPF_5_ with pyridine in CDCl_3_ yielded a 1:1 binding constant of (1.3 ± 0.3) × 10^4^ M^–1^ (see SI, Figures S49–S50), which is twice that reported for pyridine and ZnTPPH (6.9 × 10^3^ M^–1^, CH_2_Cl_2_).^68^ This result confirms the enhancement of the association between pyridine and ZnTPPF_5_ due to the increased electrophilicity of Zn in the meso-tetrasubstituted porphyrin decorated with perfluoroaryl moieties.

To evaluate the impact of aryl perfluorination on capsule formation, ligands **1** and **2** were reacted with ZnTPPF_5_ in CHCl_3_ (Scheme 2). Slow diffusion of *n*-hexane into a CHCl_3_ solution of the ligands **1** or **2** (1 equiv) and ZnTPPF_5_ (3 equiv) gave red hexagonal crystals of {[E­(3-py)_3_]**·**(ZnTPPF_5_)_3_} (E = Sb (**1·**ZnTPPF_5_), Bi (**2·**ZnTPPF_5_)) in 20 and 55% crystalline yields, respectively. The room-temperature ^1^H NMR spectra in CDCl_3_ of crystalline samples of **1·**ZnTPPF_5_ and **2·**ZnTPPF_5_ showed a 1:3 ratio of **1** or **2** to ZnTPPF_5_ and a large shift of the ^1^H NMR signals of the pyridyl proton indicative of axial binding. Mass spectrometry (see SI, Figures S74–S81) and X-ray studies further confirmed the formation of 1:3 complexes (*vide infra* and Figure 6).

**Scheme 2:**
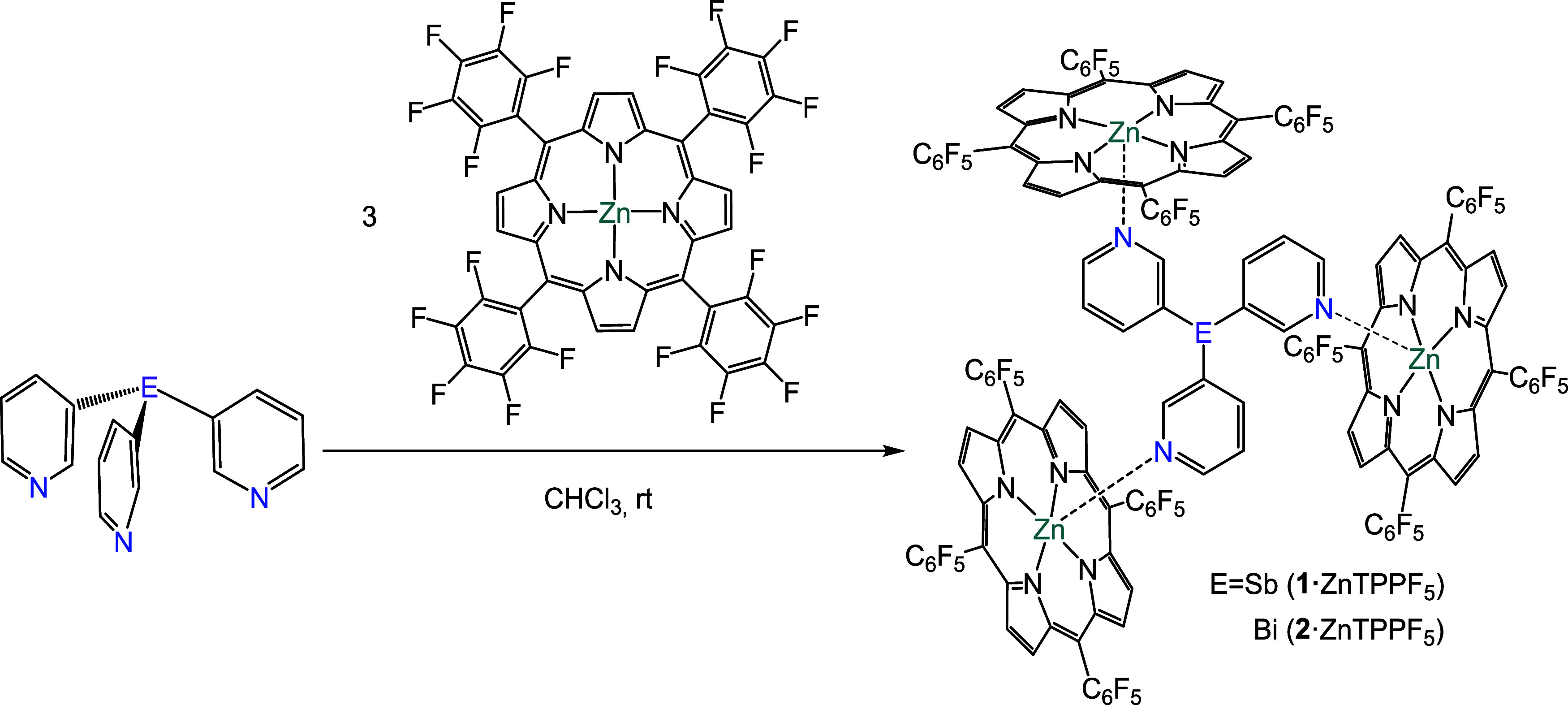
Synthesis of the Capsules {[Sb­(3-py)_3_]**·**(ZnTPPF_5_)_3_} (**1·**ZnTPPF_5_) and {[Bi­(3-py)_3_]**·**(ZnTPPF_5_)_3_} (**2·**ZnTPPF_5_)

The dynamic nature of the Zn–N interactions in **1·**ZnTPPF_5_ and **2·**ZnTPPF_5_ was investigated via ^1^H and ^19^F NMR in CDCl_3_. The ^19^F spectra of crystalline samples of **1·**ZnTPPF_5_ and **2·**ZnTPPF_5_ consist of three signals corresponding to the F_ortho_, F_meta_, and F_para_ atoms of the C_5_F_6_ groups. At 293 K, the F_para_ signal is sharp, while the F_ortho_ and F_meta_ peaks exhibit significant broadening. Upon heating, the signals sharpen, while reducing the temperature below 273 K results in splitting of the F_ortho_ and F_meta_ peaks, but not the F_para_ signal (Figure 5 and SI for **2**·ZnTPPF_5_, Figure S32). This behavior indicates restricted motion of the C_6_F_5_ rings. The low-temperature data is consistent with a conformation in which the C_6_F_5_ groups are oriented perpendicular to the plane of the metalloporphyrin to relieve steric congestion in the {E­(3-py)_3_-(ZnTPPF_5_)_3_} shell, as observed in the solid-state structures (Figure 6). This arrangement gives rise to two inequivalent sets of ortho and meta fluorine atoms, with one ortho and one meta fluorine atom oriented toward the inside of the capsule (in) and the others oriented away from the capsule (out) (see Figure 5). Although no signals corresponding to free ZnTPPF_5_ are observed upon lowering the temperature, it should be noted that lowering the temperature favors molecular association processes.

**Figure 5:**
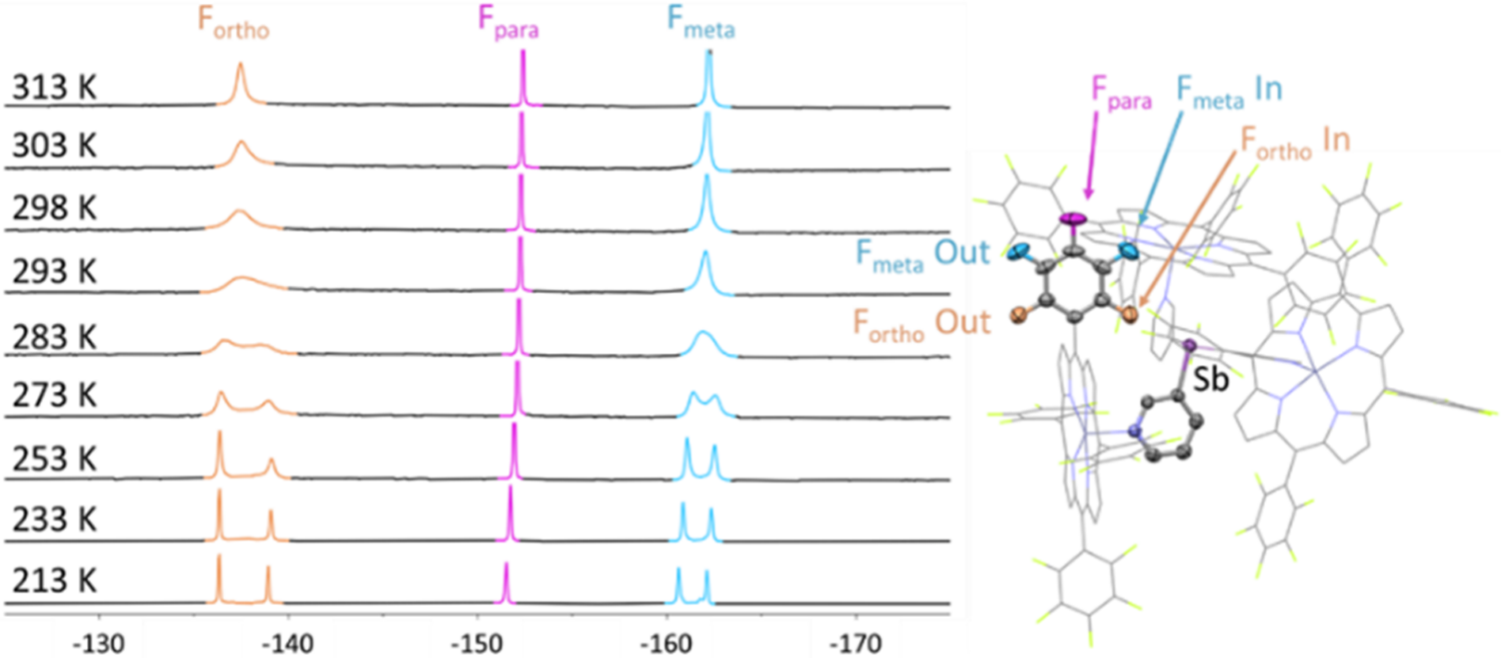
Variable temperature ^19^F NMR spectra (470.17 MHz) of a sample of **1·**ZnTPPF_5_ in CDCl_3_. Reducing the temperature below 273 K results in the splitting of the ^19^F signals corresponding to the F_ortho_ and F_meta_ atoms.

The exchange observed between the inequivalent ortho and meta positions may be due to either rotation around the C–C bond or reversible coordination and decoordination of the ZnTPPF_5_ moieties. However, the activation free energy value (Δ*G* ^‡^ ≈ 12–13 kcal·mol^–1^ for **1·**ZnTPPF_5_ and **2·**ZnTPPF_5_) estimated from the coalescence temperature is significantly lower than the typical rotational barriers for the aryl group in related porphyrins.^69,70^

Addition of free ZnTPPF_5_ to **1·**ZnTPPF_5_ resulted in one set of signals in the ^19^F NMR spectrum; no additional signals were observed, indicating fast exchange of bound and unbound ZnTPPF_5_ on the NMR time scale at room temperature. Cooling to 213 K resulted in two distinct sets of signals: one set for the free (unbound) ZnTPPF_5_, and another set of five signals identical to those observed for the intact capsule **1**·ZnTPPF_5_, indicating that the exchange process becomes slow at low temperature (see SI, Figure S33). Consistent with this, the ^1^H NMR spectrum shows signals for free ZnTPPF_5_ along with **1·**ZnTPPF_5_. In sharp contrast, Zn–pyridine exchange in {[P­(3-py)_3_]­(ZnTPPH)_3_} (E = P, Sb, Bi) capsules remained fast even at 213 K.^30,47^

Notably, the ^1^H NMR spectrum of **1**·ZnTPPF_5_ at 213 K shows only one singlet for the β-pyrrole protons of ZnTPPF_5_, while the ^19^F NMR spectrum displays five signals, consistent with an effective *C*_4*v*_ symmetry for the ZnTPPF_5_ units and the magnetic equivalence of all three porphyrin moieties, likely due to Zn–N bond rotation. Similarly, only one set of resonances for the pyridyl rings is observed, consistent with an apparent *C*_3*v*_ symmetry in solution for the ligand. These simple NMR features suggest that the capsule is still relatively dynamic at 213 K, even though the Zn pyridine exchange is slow at this temperature, suggesting fast intramolecular motions that do not require ZnTPPF_5_ dissociation. Although the capsules most likely adopt the blocked conformation observed in the solid state (see Figure 6), they retain a degree of flexibility in solution even at 213 K.

**Figure 6:**
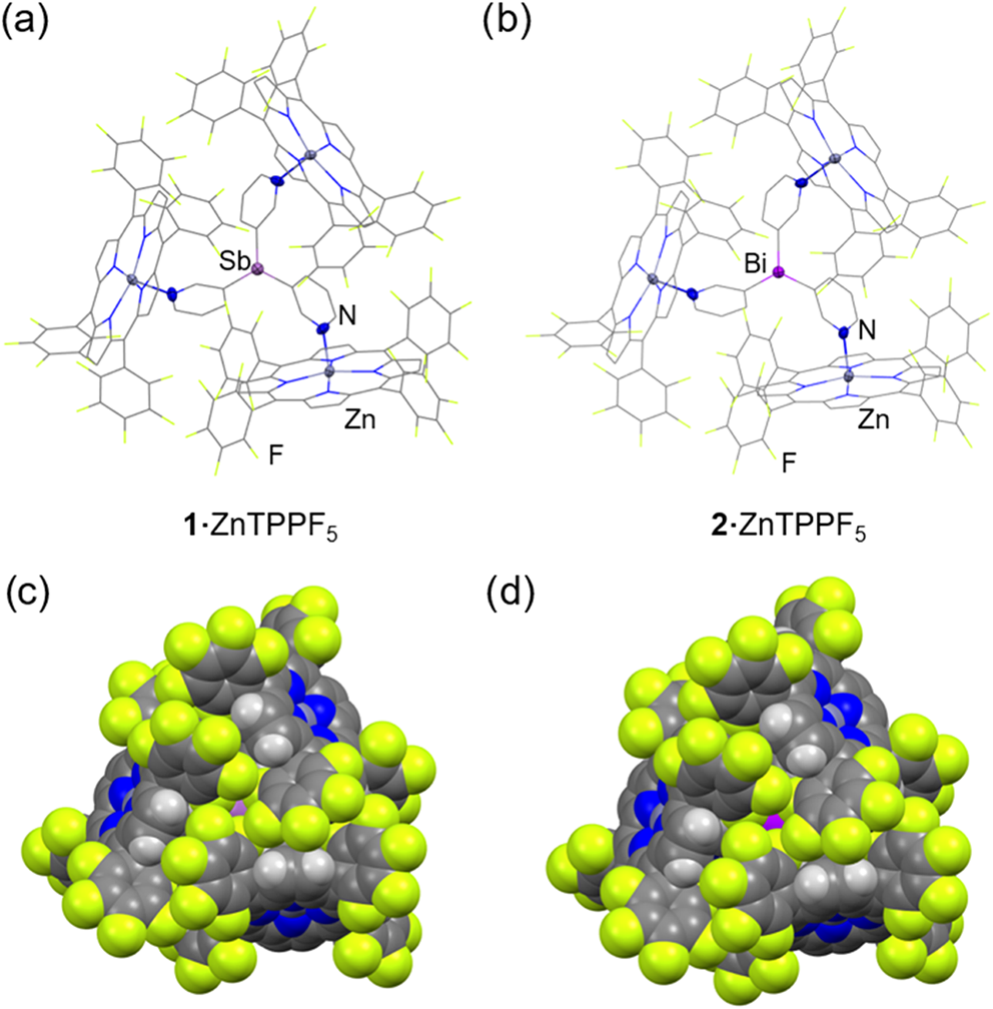
Solid-state structures of the supramolecular capsules {[Sb­(3-py)_3_]**·**(ZnTPPF_5_)_3_} (**1·**ZnTPPF_5_) (a) and {[Bi­(3-py)_3_]**·**(ZnTPPF_5_)_3_} (**2·**ZnTPPF_5_) (b). Displacement ellipsoids of the heteroatoms are shown at 50% probability; H atoms and solvent molecules are omitted for clarity. (b) Space-filling view of {[Sb­(3-py)_3_]**·**(ZnTPPF_5_)_3_} (**1·**ZnTPPF_5_) (c) and {[Bi­(3-py)_3_]**·**(ZnTPPF_5_)_3_} (**2·**ZnTPPF_5_) (d). Selected bond lengths (Å) and angles (deg): **1·**ZnTPPF_5_, Sb–C_py_ 2.142(5); N_py_– Zn 2.137(4); C_py_–Sb–C_py_ 95.4(2). **2·**ZnTPPF_5_, Bi–C_py_ 2.227(6); N_py_–Zn 2.127(5); C_py_– Bi–C_py_ range 94.3(2). Color key: C (gray), Zn (dark gray), N (blue), F (yellow), H (white), Sb (light purple), Bi (purple).

Crystallographic analysis showed that **1·**ZnTPPF_5_ and **2·**ZnTPPF_5_ crystallize in the cubic space group *Pa*3̅, forming isostructural bimetallic supramolecular capsules in which each ligand **1** or **2** coordinates three ZnTPPF_5_ metalloporphyrins (Figure 6a,6b, respectively). The group 15 bridgehead atom of the ligand sits on a crystallographic 3-fold rotation axis, producing three equivalent (3-py)-ZnTPPF_5_ fragments. The pyridine rings are slightly rotated, with one perfluorinated phenyl group from each porphyrin oriented upward and inward toward the bridgehead atom, thus producing a “blocked” conformation in which the bridgehead atom is entirely enclosed and inaccessible (Figure 6c,d). This novel conformation contrasts with the two observed in our previous structural studies of the ZnTPPH capsules {[E­(3-py)_3_]·(ZnTPPH)_3_} (E = Sb, Bi), namely, an “open” form with one porphyrin unit displaced downward, and a “closed” cylindrical arrangement featuring a well-defined cavity around the bridgehead atom (Figure 7).^47^ Notably, the closed form was the only conformation previously observed for {[P­(3-py)_3_]·(ZnTPPH)_3_}.^29^ Although the present “blocked” conformation is somewhat reminiscent of the “closed” conformation, it lacks the cavity around the bridgehead atom (Sb or Bi) and renders the bridgehead atom entirely inaccessible. The space-filling X-ray structures of **1**·ZnTPPF_5_ and **2**·ZnTPPF_5_ show this steric shielding clearly, with the fluorine atoms of the upper perfluorinated rings flanking and fully enclosing the bridgehead center (Figure 6c,d). Beyond the aesthetic appeal of this highly symmetrical supramolecular assembly, it can be anticipated that this new blocked conformation should significantly influence the capsule properties and reactivity.

**Figure 7:**
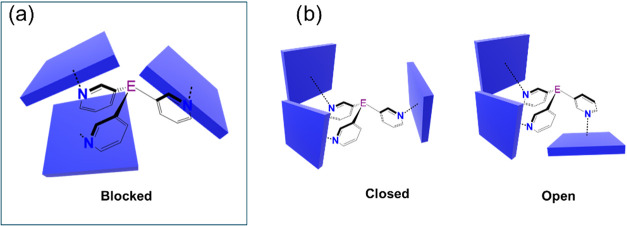
Schematic representation of the observed conformations for capsules of the type [(E­(3-py)_3_)**·**(ZnTPPX)_3_] (ZnTPPX = ZnTPPF_5_ (a) or ZnTPPH (b)). E = Sb, Bi. Blue blocks represent the corresponding metalloporphyrin.

**1·**ZnTPPF_5_ and **2·**ZnTPPF_5_ also illustrate how changing the bridgehead ligand can impact the size of the capsules. Although the C–E–C angle decreases slightly from Sb to Bi (95.4(2)° to 94.3(2)°), the longer Bi–C bond (2.227(6) Å vs 2.142(5)­Å) leads to a net increase in capsule size, as reflected in the longer Zn···Zn distance in **2**·ZnTPPF_5_ compared to **1·**ZnTPPF_5_ 10.0798(9) vs 10.0589(9) Å, see SI (Figure S55). A similar periodic trend was previously observed for the ZnTPPH capsules; however, the capsules based on ZnTPPF_5_ are more compact and smaller, consistent with their shorter Zn–N bonds (∼0.04 Å shorter, 2.137(4) Å for **1**·ZnTPPF_5_ and 2.127(5) Å for **2**·ZnTPPF_5_), which are indicative of stronger coordination to the porphyrin.

The X-ray structures of **1**·ZnTPPF_5_ and **2**·ZnTPPF_5_ feature various key intramolecular interactions that shape their morphology. In **1·**ZnTPPF_5_, three F_ortho_···H_β_ interactions [2.503(5) Å, cf. ∑_VDW_(F–H) = 2.57 Å][Bibr ref67] involving adjacent porphyrin fragments are observed (Figure 8a,8b). Interactions between the ligand and the porphyrin (besides the Zn–N coordination) are also present, namely, three F_ortho_···H_py_ [2.548(4) Å, *cf*. ∑_VDW_(F–H) = 2.57 Å][Bibr ref67] and three Sb···F interactions [3.487 (3) Å, *cf*. ∑_VDW_(Sb–F) = 3.53 Å][Bibr ref67] (Figure 8c,d). Similarly, **2·**ZnTPPF_5_ displays three equivalent F_ortho_–H_β_ interactions [2.524(6) Å, *cf*. ∑_VDW_(F–H) = 2.57 Å],^67^ three F_ortho_···H_py_ interactions [2.519 Å *cf*. ∑_VDW_(F–H) = 2.57 Å][Bibr ref67] and three Bi···F interactions [3.355 (4) Å, *cf*. ∑_VDW_(Bi–F) = 3.54 Å].^67^ In both cases, the E···F (E = Sb, Bi) distances are within the sum of the van der Waals radii of the two elements, suggesting the presence of three pnictogen bonds.

**Figure 8:**
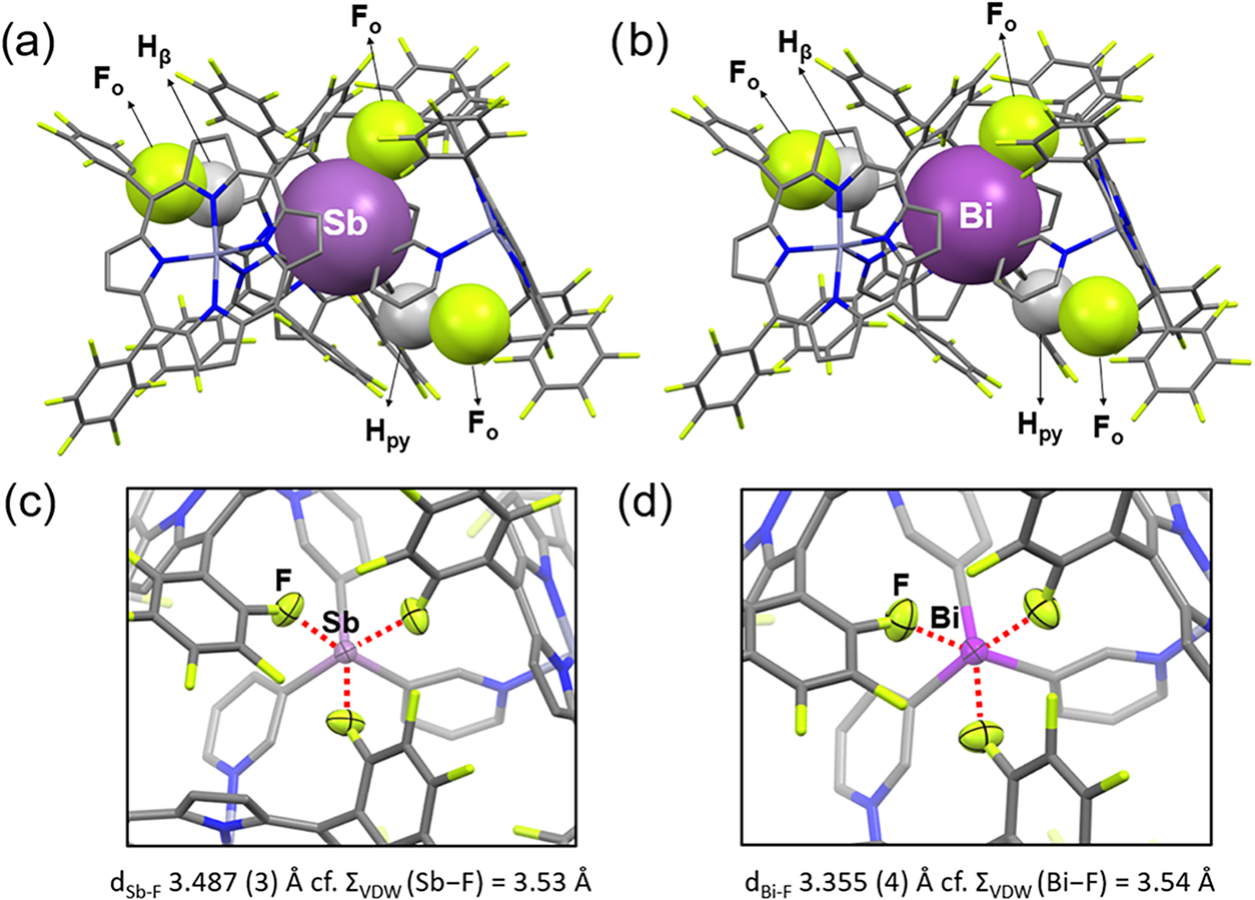
Molecular structure of the supramolecular capsules {[Sb­(3-py)_3_]**·**(ZnTPPF_5_)_3_} (**1·**ZnTPPF_5_) (a) and {[Bi­(3-py)_3_]**·**(ZnTPPF_5_)_3_} (**2·**ZnTPPF_5_) (b). Atoms involved in intramolecular interactions of the type H–F and E–F (E = Sb or Bi) are shown in space-filling view. Note: For simplicity, only one of the three interactions of each type has been highlighted. Top view of **1·**ZnTPPF_5_ (c) and **2·**ZnTPPF_5_ (d). The interactions between the three fluorine atoms of the porphyrin fragments and the bridgehead atom of the tris­(3-pyridyl) ligand are highlighted with red dashed lines, showing the formation of three E···F pnictogen bonds to form a distorted octahedral coordination around the bridgehead through the three-point binding. Both the Sb···F contacts in **1·**ZnTPPF_5_ (3.487 (3) Å) and the Bi···F contacts in **1·**ZnTPPF_5_ (3.355 (4) Å) are below the sum of the van der Waals radii (∑_VDW_(Sb–F) = 3.53 and ∑_VDW_(Bi–F) = 3.54 Å). Displacement ellipsoids of the heteroatoms are shown at 50% probability; H atoms and solvent molecules are omitted for clarity. Color key: C (gray), Zn (dark gray), N (blue), F (yellow), H (white), Sb (light purple), Bi (purple).

The nature of the three bridgehead–fluorine (E···F) interactions observed in **1**·ZnTPPF_5_ and **2**·ZnTPPF_5_ merits further analysis, as they account for a significant portion of the total attractive intramolecular interactions (50% (−3.09 kcal mol^–1^) and 68% (−6.75 kcal mol^–1^), respectively) based on second-order perturbation theory from NBO analysis (see SI, Table S12).^71,72^ The presence of bond critical points (BCPs) along each E···F axis in the QTAIM (Quantum Theory of Atoms-In-Molecules) analysis further supports the existence of directional interactions consistent with pnictogen bonding.^73^ NBO calculations also identified three F→E donor–acceptor interactions involving the lone pairs of the fluorine atoms and the unoccupied σ*­(E–C) orbitals (SI, Figures S84–S85).

The trend observed from Sb­(III) to Bi­(III) is consistent with the increasing Lewis acidity of the bridgehead atom down group 15, resulting in better pnictogen donors. However, the moderate magnitude of the E···F interaction energies could indicate that these interactions arise from crystal packing forces. To differentiate between packing-dominated contacts and intrinsic noncovalent interactions, the geometries of the assemblies **1·**ZnTPPF_5_ and **2·**ZnTPPF_5_ were optimized in the gas phase using DFT methods (see SI Figure S83). These optimizations revealed a consistent shortening of the E···F distances by 0.17 Å on average relative to the crystal structures, accompanied by a substantial increase in the calculated stabilization energy (−9.87 kcal mol^–1^ and −15.12 kcal mol^–1^ for **1·**ZnTPPF_5_ and **2·**ZnTPPF_5_, respectively). These values represent an approximately 60% enhancement in magnitude compared to the crystallographic geometries. This pronounced reinforcement in the gas phase strongly suggests that the E···F interactions are not imposed by solid-state constraints, but instead reflect genuine intramolecular pnictogen bonds.

### Effect of Remote Coordination on Pnictogen Bonding

Although the heavier and more-Lewis-acidic pnictogen atoms Bi and Sb favor pnictogen bonding (PnB), the participation of the bridgehead atom (E­(III) = Sb­(III), Bi­(III)) in three directional pnictogen bonds (PnBs) in the solid-state structures of **1**·ZnTPPF_5_ and **2**·ZnTPPF_5_ is not trivial. Each PnB reduces the Lewis acidity at the bridgehead atom, thereby weakening subsequent PnBs. This negative cooperativity is further enhanced by the increased stereochemical activity of the pnictogen lone pair, which repels incoming PnB acceptors, as well as steric constraints around the bridgehead atom, which impede simultaneous approach of multiple PnB acceptors.^74–76^ Although conceptually distinct, it is worth noting recent efforts to design chelating multidentate pnictogen-bonding systems in which each pnictogen center forms a *single* PnB to cooperatively chelate guests.^77–79^

However, during the self-assembly of the supramolecular capsules **1**·ZnTPPF_5_ and **2**·ZnTPPF_5_, the formation of each PnB is accompanied by the coordination of a pyridine arm to a ZnTPPF_5_ porphyrin. This distal coordination could enhance the Lewis acidity of the bridgehead (by deepening the σ-hole and lowering the energy of the σ *E–R orbital), counteracting the negative cooperativity intrinsically associated with the formation of multiple PnBs. To test this idea, we examined the electrostatic potential (ESP) of the linkers **1** and **2** upon coordination of 1, 2, or 3 simple ZnP units (with no substitution on the meso positions of the porphyrin, Figure 9). Although this simplified model emphasizes the electrostatic component of the pnictogen bonding, the linear correlation (*R* ^2^ > 0.99, see SI Figure S90) observed between the deepening of the σ*-*holes on E (E = Sb, Bi) and the number of coordinated ZnP moieties is consistent with an enhancement of the PnB donor properties of the bridgehead. The ESP maps of free ligands **1** and **2** show three regions of high electrostatic potential at the bridgehead (*V*_max_ 19.68 and 22.88 kcal mol^–1^, respectively, see SI Figure S88) along the extension of the E–C_py_ bond. It was estimated that each porphyrin coordination results in a charge depletion of 2.2 kcal mol^–1^ of the σ-hole (Figures 9a and S89) for both ligands. Additionally, the local minima attributed to the lone pair of the pnictogen bridgehead atom became progressively less negative with each coordinated ZnP, exhibiting almost perfectly linear (*R* ^2^ = 0.9998) charge depletion at a rate of 1.1 kcal mol^–1^ per attached porphyrin (suggesting increased stereochemical inactivity, see SI, Figure S92). Supporting this, the NBO atomic natural charges showed progressively greater positive charge on the bridgehead atom with each ZnP coordination, consistent with an enhancement of its Lewis acidity (see SI, Table S13 for more details).

**Figure 9:**
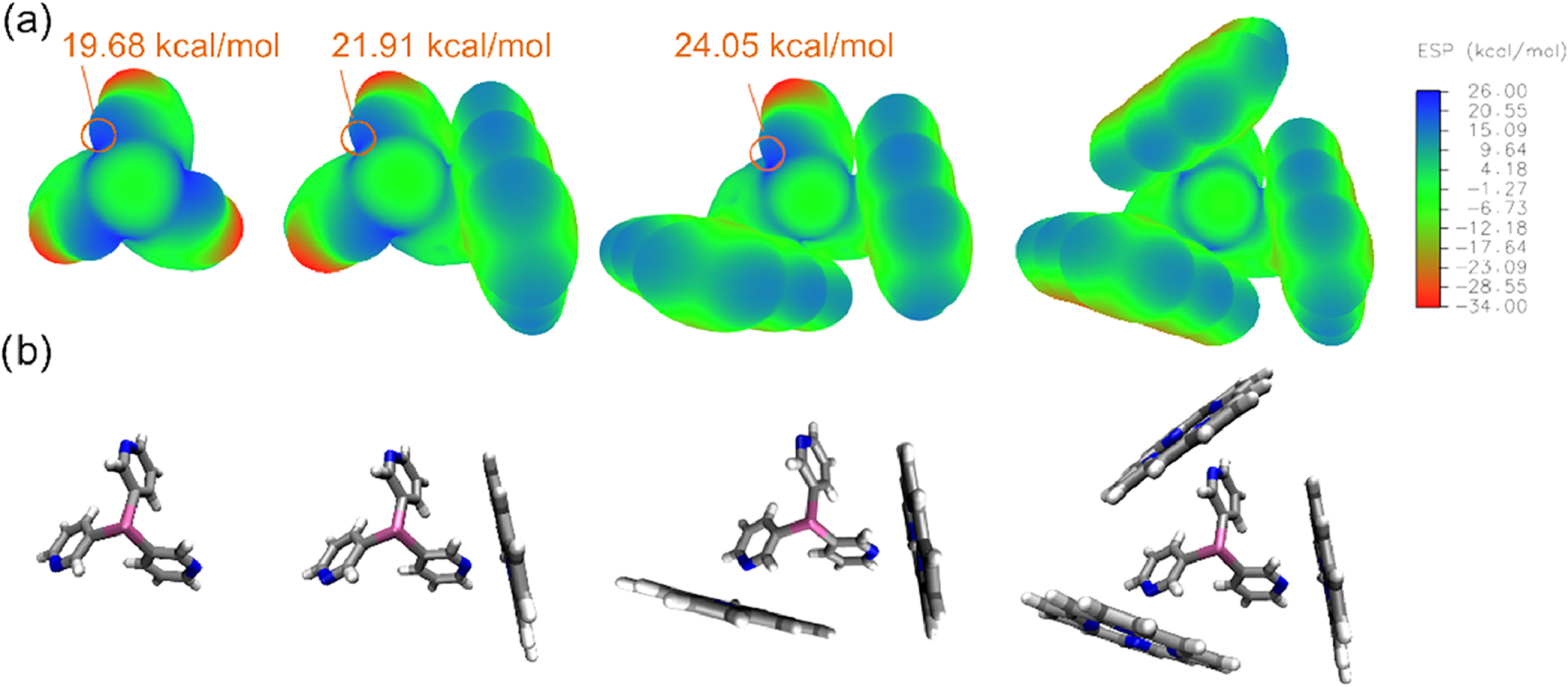
(a) ESP maps of compound Sb­(3-py_3_)_3_ (**1**) and its mono-, bis- and tris-adducts with the porphyrin ZnP along with the corresponding color scale bar (in kcal mol^–1^). Values of maxima (σ-holes) are highlighted (*V*_max_). (b) Molecular representations of the above-described species. A very similar trend is observed for the Bi­(3-py_3_)_3_ linker (**2**), see SI, Figure S94.^80^

Similarly, coordination of **1** and **2** to ZnTPPF_5_ should increase the Lewis acidity of E­(III), deepening its σ-hole and orienting a C_6_F_5_ moiety toward the bridgehead, enabling the formation of the three E···F PnBs observed experimentally. This coordination could reverse the negative cooperativity typically observed in multipnictogen-bond systems, dynamically reinforcing the otherwise weakened pnictogen interactions.

To investigate this interesting possibility, we carried out the same computational analysis using 1:1 binary assemblies of **1** and **2** with a library of zinc porphyrins: unsubstituted (ZnP), tetraphenyl (ZnTPPH), tetra­(3,4,5-trifluorophenyl) (ZnTPPH_2_F_3_), which exerts a strong electron-withdrawing effect but lacks ortho-fluorine atoms capable of forming intramolecular pnictogen bonds with the bridgehead, and tetraperfluorophenyl (ZnTPPF_5_), which combines stronger electron-withdrawing C_6_F_5_ groups with the ability to form intramolecular pnictogen bonds via the ortho-fluorine atoms (Figure 10 a,b). The evolution of the sigma hole (*V*_max_) with respect to that of the free ligands **1** and **2** is shown in Figure 10c,d, respectively, and exhibits consistent trends across both systems. Coordination to ZnTPPH deepens the sigma hole by ca. 2.7 kcal mol^–1^ relative to the free ligand (and by 0.2 kcal mol^–1^ with respect to ZnP, indicating that phenyl substitution at the meso hydrogens has only a modest effect). ZnTPPH_2_F_3_ coordination significantly enhances the σ-hole depth by an additional 5 kcal mol^–1^, yielding a total of ≈7.7 kcal mol^–1^ relative to the uncoordinated ligand. This reflects the strong electron-withdrawing effect of the C_6_H_2_F_3_ substituents. Finally, the coordination of ZnTPPF_5_ results in a reduction in the σ-hole depth by ≈4.1 kcal mol^–1^ compared to ZnTPPH_2_F_3_, despite its stronger electron-withdrawing capacity. This is due to the intramolecular pnictogen bonding between the ortho-fluorine atom and the pnictogen bridgehead, which decreases the electrophilicity of the remaining σ-holes, a hallmark of negative cooperativity often observed in multi-PnB systems. Crucially, however, even in ZnTPPF_5_, the σ-hole shows a greater net depth relative to the uncoordinated ligand (ca. 3.6 kcal mol^–1^), indicating that Zn coordination dominates over σ-hole depletion from PnB formation. This simple electrostatic model helps to explain why the formation of subsequent PnBs at the same E atom is feasible, as experimentally observed. Importantly, these findings suggest net positive cooperativity: metal coordination dynamically reinforces pnictogen bonding and sustains the formation of all three E···F interactions. To the best of our knowledge, the formation of the ‘blocked’ capsule conformation stabilized by three E–F bonds constitutes the first example of a synergistic interplay in which coordination to a remote donor site strengthens pnictogen bonding. Interestingly, coordination to ZnTPPBr deepens the σ-hole of the pnictogen center by ca. 8 kcal mol^–1^ relative to the free ligand (and by 4 ca. kcal mol^–1^ with respect to ZnP and ZnTPPH, see SI for details, Figures S100–S102). This increase in Lewis acidity contributes to the formation of the intermolecular E···Br PnBs observed in the solid-state structures of **1**·ZnTPPBr and **2**·ZnTPPBr. We note, however, that the formation of multiple intermolecular pnictogen bonds is strongly influenced by steric factors and crystal-packing constraints, as it requires the simultaneous approach of multiple semicapsules to the same pnictogen center.

**Figure 10:**
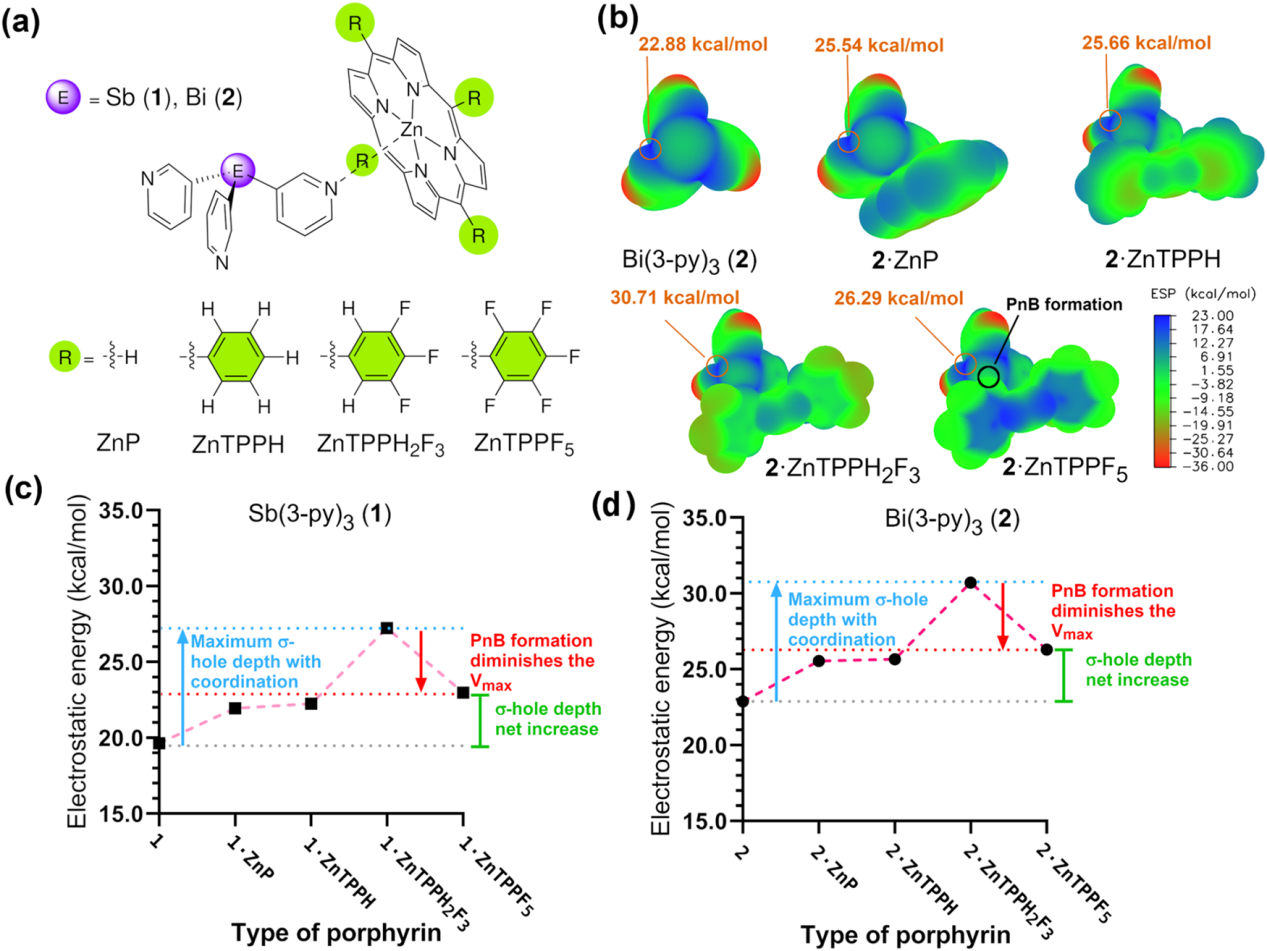
(a) Line drawing representations of the species in (b). (b) ESP maps of compound Bi­(3-py)_3_ (**2**) and its monoadducts with porphyrins ZnP, ZnTPPH, ZnTPPH_2_F_3_ and ZnTPPF_5_ along with the corresponding color scale bar (in kcal mol^–1^). Values of maxima (σ-holes) are highlighted (*V*_max_). See SI Figure S93 for the plot with Sb­(3-py_3_)_3_ linker (**1**). (c) and (d) Sigma holes (*V*_max_) for the monoadducts with ZnP, ZnTPPH, ZnTPPH_2_F_3_ and ZnTPPF_5_ and Sb­(3-py)_3_ (c) and Bi­(3-py)_3_ (d). The sigma hole that is not located trans to the E–C_py_ bond (not plotted in this figure) shows a very similar effect (see also Note 80).

To investigate the thermodynamic implications of the cooperative coordination–pnictogen bonding effect, NMR titration of the ligands E­(3-py)_3_ [E = Sb­(**1**), Bi­(**2**)] with ZnTPPF_5_ in CDCl_3_ were carried out. The above-described nonlinear regression protocol was applied here, and indicated a very strong preference toward a 1:3 model in both cases (**1**: *K*_1_ [(2.97 ± 1.4) × 10^4^ M^–1^], *K*_2_ [(1.40 ± 0.9) × 10^4^ M^–1^], *K*_3_ [(2.28 ± 0.3) × 10^3^ M^–1^]; **2**: *K*_1_ [(1.9 ± 1.0) × 10^5^ M^–1^], *K*_2_ [(2.17 ± 0.3) × 10^5^ M^–1^], and *K*_3_ [(5.64 ± 0.7) × 10^3^ M^–1^]). Job plot analysis further confirmed the formation of a 1:3 complex (see SI, Figures S51–S54), and DOSY experiments indicated that the capsules retained their integrity in CDCl_3_. Importantly, the first stepwise constants (*K*_1_) for **1** and **2** are higher than the binding constant between free pyridine and ZnTPPF_5_, consistent with additional stabilization arising from pnictogen-bond formation. Moreover, the larger association constants obtained for Bi­(3-py)_3_ suggest a larger stabilization due to Bi···F pnictogen bond formation in the capsule **2** compared to the weaker Sb···F one in **1·**ZnTPPF_5_. Additionally, the association constants of **1·**ZnTPPF_5_ and **2·**ZnTPPF_5_ are one to 2 orders of magnitude greater than those obtained for the capsules based on the metalloporphyrin ZnTPPH ({[E­(3-py)_3_]**·**(ZnTPPH)_3_} (E = Sb, Bi)).^47^ This enhancement likely results from a combination of (i) the stronger coordination of the N-py donor groups toward ZnTPPF_5_ compared to ZnTPPH, and (ii) additional intramolecular stabilization through the formation of three E···F pnictogen bonds.

Thus, while the use of ligands **1** and **2** with ZnTPPOMe and ZnTPPBr disfavored capsule formation, the use of ZnTPPF_5_ produced 1:3 supramolecular capsules. The presence of three strong E···F PnBs consistent with σ-hole deepening by means of coordination to electrophilic guests led to robust capsules **1·**ZnTPPF_5_ and **2·**ZnTPPF_5_ featuring an unprecedented “blocked conformation”, effectively isolating the pnictogen center within the supramolecular framework, a feature expected to also manifest kinetically.

### Steric Shielding of the Bridgehead Atom: Inhibition of Catalysis

Organopnictogen catalysis has emerged as a rapidly advancing area within organic chemistry, offering distinctive properties and reactivities that not only mimic but also complement those of transition metals.^39,50,81^ We thus investigated the possible kinetic effect of the blocked conformation on the reactivity with the aim of establishing capsule shape–reactivity relationships, as this conformation was expected to profoundly influence the kinetic behavior. As a test reaction, we chose the Sb-catalyzed oxidation of the α-hydroxyketones^39,82^ acetoin (MeCOCH­(OH)­Me) and benzoin (PhCOCH­(OH)­Ph) to the corresponding diketones diacetyl (MeCOCOMe) and benzyl (PhCOCOPh) under aerobic oxidation conditions (air). In the absence of ligand **1**, no conversion was observed (Table 1, entries 1–2). When the free tris­(3-pyridyl) stibine (**1**) was used as the catalyst, the oxidation of both acetoin and benzoin was accomplished in moderate yield (entry 3), whereas the use of its encapsulated form **1·**ZnTPPF_5_ dramatically inhibited the oxidation of acetoin and benzoin (entry 4). Interestingly, the present catalytic behavior shows a contrast with that of the previously reported capsule {[Sb­(3-py)_3_]**·**(ZnTPPH)_3_}. While this previous capsule also exhibited decreased activity compared to **1** in the oxidation of acetoin to diacetyl, it did not in the oxidation of benzoin to benzyl.^47^

**Table 1:**
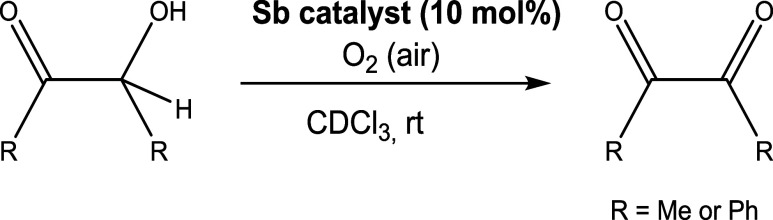
Sb-Catalyzed Oxidation of α-Hydroxyketones^c^

entry	Sb catalyst	yield (%) R = Me	yield (%) R = Ph
1	^a^	0	0
2	^b^	0	0
3	**1**	43	44
4	**1**·ZnTPPF_5_	6	7

a In the absence of the catalyst and with no ZnTPPF_5_ (30% mol).

b In the absence of the catalyst and in the presence of ZnTPPF_5_ (30% mol).

c Reaction conditions: α-hydroxyketone (0.04 mmol), Sb catalyst (**1** and **1·**ZnTPPF_5_, 10% mol), 1 mL of CDCl_3_ at room temperature. Yields were determined by ^1^H NMR after 24 h.

Presumably, the coordination of ZnTPPF_5_ to the pyridyl arms renders the Sb center unreactive due to the formation of the blocked conformation assisted by three Sb···F pnictogen bonds. Although Zn^2+^ binds at a site distal from the catalytic center, the resulting encapsulation effectively blocks access to Sb­(III), impeding the necessary oxidation of the Sb­(III) bridgehead and thus preventing turnover in a manner reminiscent of noncompetitive inhibition. This result highlights the key role of conformation and the interplay of coordination and pnictogen bonding in governing the assembly and reactivity of this class of supramolecular capsules. While in the present case, the inhibitory effect hampers the oxidation of α-hydroxyketones, it demonstrates how pnictogen bonding through the choice of building block can be used to modulate the structure, dynamics and accessibility of the bridgehead atom in this family of supramolecular capsules. Such understanding is key not only for harnessing their potential in catalysis, but also for exploring the potential of encapsulation as a means to stabilize reactive heavier pnictogen species.^83,84^

## Conclusions

This study has revealed the subtle yet profound effects of substituents on the porphyrin ring on the formation and conformational control of supramolecular capsules based on the coordination of the heavier Group 15 E­(3-py)_3_ ligands [E = Sb (**1**), Bi (**2**)] to metalloporphyrins and the importance of pnictogen bonding (PnB) for modulating their architecture and reactivity. The use of relatively bulky para-substituted metalloporphyrins was found to inhibit capsule formation, yielding 1:2 semicapsules instead of the 1:3 supramolecular capsules, irrespective of the metalloporphyrin used (Zn^2+^ or Mg^2+^) or the electronic nature of the substituent (−OMe or −Br). We have also demonstrated the use of pnictogen bonding (PnB) in the conformational control of these supramolecular capsules. The metalloporphyrin ZnTPPF_5_ produced the complete 1:3 capsules **1·**ZnTPPF_5_ and **2·**ZnTPPF_5_ exhibiting an unprecedented blocked conformation that is supported by three strong pnictogen bonds F···E (E = Sb, Bi) and renders the pnictogen atom completely encapsulated. Computational analysis indicated that the distal coordination of the pyridyl arms to the porphyrin deepens the σ-holes at the pnictogen bridgehead, enhancing its Lewis acidity and strengthening pnictogen bonding. As a result, these systems show net positive cooperativity rather than the intrinsic negative cooperativity associated with multiple pnictogen bond formation. Thus, this “remote coordination approach” represents a new tool for modulating PnB in supramolecular systems. The isolation of the pnictogen center within the capsule has a marked effect on its reactivity, as demonstrated by the suppression of the catalytic activity of the encapsulated Sb­(III) center in **1·**ZnTPPF_5_. The result shows how conformational control and pnictogen-bonding cooperativity can be exploited to modulate reactivity in this type of supramolecular capsules.

The fine-tuning of the properties of these supramolecular systems via the very subtle (and easy) modifications to their molecular design described in this work, and the importance of the integration of pnictogen bonding (PnB) in these supramolecular settings, could ultimately give rise to a vast library of on-demand tailored supramolecular assemblies. Ongoing work in our laboratory is focused on extending these systems to other catalytic transformations and exploring their ability to stabilize reactive heavier pnictogen species.

## Experimental Section

### General Experimental Techniques

All syntheses were carried out on a vacuum line under a N_2_ atmosphere. Products were isolated and handled under a N_2_ atmosphere. 3-Bromopyridine, NMR solvents, and reaction solvents were stored over molecular sieves and degassed using three freeze–pump–thaw cycles under N_2_ prior to use. Compounds Sb­(3-py)_3_ (**1**) and Bi­(3-py)_3_ (**2**) were synthesized as described previously.^46^ Porphyrins were synthesized following variations of described methods in the literature (see SI, pages S3–S4). When needed, microwave reactions were carried out with an Anton Paar Monowave 300 Reactor using sealed G10 and G30 reaction vessels (for volumes up to 10 and 30 mL, respectively) specially designed for the apparatus.

NMR spectra were recorded using 500 MHz Agilent DD2 instruments equipped with a cold probe and a 400 MHz Agilent instrument equipped with a ONEPROBE in the Laboratory of Instrumental Techniques (LTI) Research Facilities, University of Valladolid. Chemical shifts (δ) are reported in parts per million (ppm). ^1^H and ^13^C NMR spectra are referenced to TMS. ^19^F NMR is referenced to CFCl_3_. ^1^H spectra were acquired on a 500 MHz Agilent spectrometer using the 2D DOSY gradient compensated stimulated echo with convection compensation (DgcsteSL-cc) pulse sequence. Sixteen gradient levels ranging from 7 to 53 G/cm (12% to 88% of the maximum gradient strength) were used. The diffusion delay (Δ) was 50 ms and the diffusion gradient length (δ) was 1.7 ms. For each DOSY NMR experiment, a series of 16 spectra was collected. Spectra were recorded in CDCl_3_, and the temperature was set to and controlled at 298 K. Coupling constants (*J*) are reported in Hz. Standard abbreviations are used to indicate multiplicity: s = singlet, d = doublet, t = triplet, and m = multiplet. ^1^H and ^13^C peak assignments were performed with the help of additional 2D NMR experiments (^1^H–^13^C HSQC, ^1^H–^13^C HMBC, ^19^F–^13^C HSQC and ^19^F–^13^C HMBC). High-resolution mass spectra were recorded at the mass spectrometry service of the Laboratory of Instrumental Techniques (LTI) of the University of Valladolid. An Agilent TOF-LC/MS 6210 spectrometer (ESI-TOF, positive ion mode), a UPLC-MS system (UPLC: Waters ACQUITY H-class UPLC; MS: Bruker Maxis Impact) with electrospray ionization (ESI positive ion mode), a MALDI-TOF system (MALDI-TOF) and a Bruker autoflex speed (N_2_ laser: 337 nm, pulse energy: 100 μJ, 1 ns; acceleration voltage: 19 kV, reflector positive mode) were used. *Trans*-2-[3-(4-*tert*-butylphenyl)-2-methyl-2-propenylidene]­malonitrile (DCTB) was used as the matrix. Elemental analysis was obtained using a Thermo Scientific FLASH 2000 Elemental Analyzer at the Parque Científico Tecnológico (PTC) facilities of the University of Burgos.

Diffraction data were collected using an Oxford Diffraction Supernova diffractometer equipped with an Atlas CCD area detector and a four-circle kappa goniometer. For the data collection, a Mo microfocused source with multilayer optics was used. When necessary, crystals were mounted directly from solution using perfluorohydrocarbon oil to prevent atmospheric oxidation, hydrolysis, and solvent loss. Data integration, scaling, and empirical absorption correction were performed using the CrysAlisPro software package.^85^ The structure was solved by direct methods and refined by full-matrix-least-squares against F2 with SHELX^86^ in OLEX2.^87^ Non-hydrogen atoms were refined anisotropically, and hydrogen atoms were placed at idealized positions and refined using the riding model. Graphics were made with OLEX2 ^87^ and MERCURY.^88^ See SI, section “X-ray Crystallographic Studies” for further details.

### Synthesis of 1·ZnTPPOMe

A Schlenk tube under N_2_ was charged with **1** (5.6 mg, 0.016 mmol) and ZnTPPOMe (16.7 mg, 0.021 mmol), and 3 mL of CHCl_3_ was then added. The resulting dark purple solution was stirred for 15 min at r.t. Slow diffusion of hexane (20 mL) yielded **1**·ZnTPPOMe as purple crystals suitable for X-ray crystallography, which were dried under vacuum. Yield (calculated as {[Sb­(3-py)_3_]·(ZnTPPOMe)_2_}): 17.4 mg (0.009 mmol, 57%). ^1^H NMR (298 K, CDCl_3_, 400 MHz): δ = 8.79 (s, 16H, H_14_), 7.97 (d, *J* = 7.68 Hz, 16H, H_10_), 7.20 (d, *J* = 7.89 Hz, 16H, H_9_), 6.24 (br, 3H, H_5_), 6.07 (br, 3H, H_4_), 5.76 (br, 3H, H_6_), 5.34 (br, 3H, H_2_). ^13^C­{^1^H} NMR (298 K, CDCl_3_, 100.56 MHz): δ = 159.00 (C8), 152.30 (C2), 140.17 (C13), 147.30 (C_6_), 142.20 (C_4_), 135.67 (C_11_), 135.40 (C_10_), 131.48 (C_14_), 130.25 (C_3_), 124.04 (C_5_), 120.10 (C_12_), 111.79 (C_9_), 55.53 (C_7_). HR-MS [ESI, positive ion mode ESI-TOF]: *m*/*z* for C_111_H_84_N_11_O_8_SbZn_2_ [**1**·ZnTPPOMe + H]^+^: Calcd: 1952.4200. Found: 1952.4101 (2.0 ppm error).

### Synthesis of 2·ZnTPPOMe

A Schlenk tube under N_2_ was charged with **2** (6.9 mg, 0.016 mmol) and ZnTPPOMe (16.6 mg, 0.021 mmol), and 3 mL of CHCl_3_ was then added. The resulting dark purple solution was stirred for 15 min at r.t. Slow diffusion of hexane (20 mL) yielded **2**·ZnTPPOMe as purple crystals suitable for X-ray crystallography, which were dried under vacuum. Yield (calculated as {[Bi­(3-py)_3_]·(ZnTPPOMe)_2_}): 20.9 mg (0.01 mmol, 65%). ^1^H NMR (298 K, CDCl_3_, 400 MHz): δ = 8.79 (s, 16H, H_14_), 7.97 (d, *J* = 8.04 Hz, 16H, H_10_), 7.20 (d, *J* = 7.89 Hz, 16H, H_9_), 6.39 (br, 3H, H_4_), 6.27 (br, 3H, H_5_), 5.74 (br, 3H, H_6_), 5.43 (br, 3H, H_2_), 4.08 (s, 24H, H_12_). ^13^C­{^1^H} NMR (298 K, CDCl_3_, 100.56 MHz): δ = 159.17 (C_8_), 153.34 (C_2_), 150.37 (C_13_), 146.62 (C_6_), 144.08 (C_4_), 135.83 (C_11_), 135.58 (C_10_), 131.67 (C_14_), 126.01 (C_5_), 120.31 (C_12_), 111.97 (C_9_), 55.71 (C_7_). HR-MS [ESI, positive ion mode ESI-TOF]: *m*/*z* for C_111_H_84_N_11_O_8_BiZn_2_ [**2**·ZnTPPOMe + H]^+^: Calcd: 2040.4960. Found: 2040.4966 (0.6 ppm error).

### Synthesis of 1·ZnTPPBr

A Schlenk tube under N_2_ was charged with **1** (4.5 mg, 0.013 mmol) and ZnTPPBr (25 mg, 0.025 mmol), and 3 mL of CHCl_3_ was then added. The resulting dark purple solution was stirred for 15 min at r.t. Slow diffusion of hexane (20 mL) yielded **1**·ZnTPPBr as purple crystals suitable for X-ray crystallography, which were dried under vacuum. Yield (calculated as {[Sb­(3-py)_3_]·(ZnTPPBr)_2_} based on elemental analysis that indicates complete solvent loss from the crystal): 16.4 mg (0.007 mmol, 53%). ^1^H NMR (298 K, CDCl_3_, 400 MHz): δ = 8.79 (s, 16H, H_13_), 7.93 (d, *J* = 8.18 Hz, 16H, H_9_), 7.84 (d, *J* = 8.18 Hz, 16H, H_8_), 6.21 (br, 3H, H_4_), 6.05 (br, 3H, H_5_), 5.60 (br, 3H, H_6_), 5.08 (br, 3H, H_2_). ^13^C­{^1^H} NMR (298 K, CDCl_3_, 125.67 MHz): 152.01 (C_2_), 149.79 (C_12_), 147.15 (C_6_), 142.25 (C_4_), 141.79 (C_10_), 135.75 (C_9_), 131.78 (C_13_), 130.13 (C_3_), 129.63 (C_8_), 124.03 (C_5_), 122.17 (C_7_), 119.49 (C_11_). Elemental analysis (%) for **1**·ZnTPPBr (C_103_H_60_Br_8_N_11_SbZn_2_): Calcd: C 52.9, H 2.5, N 6.3. Found: C 53, H 2.6, N 6.4. HR-MS [ESI, positive ion mode ESI-TOF]: *m*/*z* for C_103_H_61_Br_8_N_11_SbZn_2_ [**1**·ZnTPPBr + H]^+^: Calcd: 2343.6112. Found: 2343.6122 (1.0 ppm error).

### Synthesis of 2·ZnTPPBr

A Schlenk tube under N_2_ was charged with **2** (5.6 mg, 0.013 mmol) and ZnTPPBr (25 mg, 0.025 mmol), and 3 mL of CHCl_3_ was then added. The resulting dark purple solution was stirred for 15 min at r.t. Slow diffusion of hexane (20 mL) yielded **2**·ZnTPPBr as purple crystals suitable for X-ray crystallography, which were dried under vacuum. Yield (calculated as [Bi­(3-py)_3_·(ZnTPPBr)_2_] based on elemental analysis that indicates complete solvent loss from the crystal): 20.9 mg (0.008, 66%). ^1^H NMR (298 K, CDCl_3_, 500 MHz): δ = 8.79 (s, 16H, H_13_), 7.92 (d, *J* = 8.28 Hz, 16H, H_9_), 7.82 (d, *J* = 8.28 Hz, 16H, H_8_), 6.38 (br, 3H, H_4_), 6.25 (br, 3H, H_5_), 5.64 (br, 3H, H_6_), 5.21 (br, 3H, H_2_). ^13^C­{^1^H} NMR (298 K, CDCl_3_, 125.67 MHz): 153.05 (C_2_), 149.80 (C_12_), 146.37 (C_6_), 143.89 (C_4_), 141.81 (C_10_), 135.76 (C_9_), 131.78 (C_13_), 129.63 (C_8_), 125.82 (C_5_), 122.16 (C_7_), 119.48 (C_11_). Elemental analysis (%) for **2**·ZnTPPBr (C_103_H_60_Br_8_N_11_BiZn_2_): Calcd: C 51.6, H 2.5, N 6.1. Found: C 51.4, H 2.6, N 6.2. HR-MS [ESI, positive ion mode ESI-TOF]: *m*/*z* for C_103_H_61_Br_8_N_11_BiZn_2_ [**2**·ZnTPPBr + H]^+^: Calcd: 2431.6871. Found: 2431.6874 (0.3 ppm error).

### Synthesis of 1·MgTPPBr

A Schlenk tube under N_2_ was charged with **1** (6.23 mg, 0.018 mmol) and MgTPPBr (25 mg, 0.026 mmol), and 3 mL of CHCl_3_ was then added. The resulting dark purple solution was stirred for 15 min at r.t. Slow diffusion of hexane (20 mL) yielded **1**·MgTPPBr as purple blocks suitable for X-ray crystallography, which were dried under vacuum. Yield (calculated as {[Sb­(3-py)_3_]_2_·(MgTPPBr)_3_}): 46.41 mg (0.013 mmol, 15%). ^1^H NMR (298 K, CDCl_3_, 400 MHz): δ = 8.77 (s, 24H, H_13_), 7.93 (d, *J* = 8.71 Hz, 24H, H_9_), 7.81 (d, *J* = 8.71 Hz, 24H, H_8_), 6.63–6.56 (m, 18H, H_4_ + H_5_ + H_6_), 6.24 (br, 6H, H_2_). ^13^C­{^1^H} NMR (298 K, CDCl_3_, 100.56 MHz): δ = 153.19 (C_2_), 149.68 (C_12_), 148.20 (C_6_), 142.71 (C_4_), 142.27 (C_10_), 135.90 (C_9_), 131.88 (C_13_), 130.63 (C_3_), 129.48 (C_8_), 124.27 (C_5_), 121.95 (C_11_), 120.37 (C_7_). HR-MS [ESI, positive ion mode ESI-TOF]: *m*/*z* for C_103_H_60_Br_8_Mg_2_N_11_Sb [Sb­(3-py)_3_·(MgTPPBr)_2_ + H]^+^: Calcd: 2283.708. Found: 2283.7112 (−0.2 ppm error).

### Synthesis of 1·ZnTPPF_5_

A narrow Schlenk tube under N_2_ was charged with **1** (3.4 mg, 0.010 mmol) and ZnTPPF_5_ (30 mg, 0.029 mmol), and 3 mL of CHCl_3_ was then added. The resulting purple solution was stirred for 15 min at r.t. Slow diffusion of hexane (20 mL) at −25 °C yielded **1**·ZnTPPF_5_ as red crystals suitable for X-ray crystallography, which were dried under vacuum. Yield (calculated as desolvated {[Sb­(3-py)_3_]·(ZnTPPF_5_)_3_} based on elemental analysis that indicates complete solvent loss from the crystal): 6.8 mg (0.002 mmol, 20%). ^1^H NMR (298 K, CDCl_3_, 500 MHz): δ = 8.70 (s, 24H, H_13_), 5.47 (br, 3H, H_5_), 4.91 (br, 3H, H_4_). ^1^H NMR (238 K, CDCl_3_, 500 MHz): δ = 8.60 (s, 24H, H_13_), 4.74 (br, 3H, H_5_), 3.25 (br, 3H, H_4_), 1.85 (br, 3H, H_6_), 1.27 (br, 3H, H_2_). ^19^F NMR (298 K, CDCl_3_, 470 MHz): δ = −137.36 (br, 24F, F_9_), −152.33 (t, *J* = 21.32 Hz, 12F, F_7_), −162.13 (br, 24F, F_8_). ^13^C­{^1^H} NMR (298 K, CDCl_3_, 125.67 MHz): δ = 149.85 (s, C_12_), 146.26 (d, *J* = 238.30 Hz, C_9_), 141.86 (d, *J* = 256.22 Hz, C_7_), 141.67 (s, C_4_), 137.31 (d, *J* = 254.43 Hz, C_8_), 131.52 (s, C_13_), 130.04 (s, C_3_), 123.28 (s, C_5_), 116.52 (s, C_10_), 103.22 (s, C_11_). Elemental analysis (%) for **1·**ZnTPPF_5_: Calcd: (C_147_H_36_F_60_N_15_SbZn_3_): C 50.88, H 1.05, N 6.06. Found: C 51.1, H 1.1, N 5.8. HR-MS [ESI, positive ion mode ESI-TOF]: *m*/*z* for C_147_H_36_F_60_N_15_NaSbZn_3_ [**1**·ZnTPPF_5_ + Na]^+^: Calcd: 3491.9123. Found: 3491.9182 (5.9 ppm error).

### Synthesis of 2·ZnTPPF_5_

A narrow Schlenk tube under N_2_ was charged with **2** (4.3 mg, 0.010 mmol) and ZnTPPF_5_ (30 mg, 0.029 mmol), and 3 mL of CHCl_3_ was then added. The resulting purple solution was stirred for 15 min at r.t. Slow diffusion of hexane (20 mL) at −25 °C yielded **2**·ZnTPPF_5_ as red crystals suitable for X-ray crystallography, which were dried under vacuum. Yield (calculated as desolvated {[Bi­(3-py)_3_]·(ZnTPPF_5_)_3_} based on elemental analysis that indicates complete solvent loss from the crystal): 15.7 mg (0.004 mmol, 55%). ^1^H NMR (298 K, CDCl_3_, 500 MHz): δ = 8.64 (s, 24H, H_13_), 5.04 (br, 3H, H_5_), 4.40 (br, 3H, H_4_). ^1^H NMR (238 K, CDCl_3_, 500 MHz): δ = 8.61 (s, 24H, H_13_), 4.78 (br, 3H, H_5_), 3.75 (br, 3H, H_4_), 1.89 (br, 3H, H_6_), 1.32 (br, 3H, H_2_). ^19^F NMR (298 K, CDCl_3_, 470 MHz): δ = −137.35 (br, 24F, F_9_), −152.30 (t, *J* = 20.97 Hz, 12F, F_7_), −162.14 (br, 24F, F_8_). ^13^C­{^1^H} NMR (298 K, CDCl_3_, 125.67 MHz): δ = 149.81 (s, C_12_), 146.18 (d, *J* = 237.82 Hz, C_9_), 143.32, (s, C_4_), 141.83 (d, *J* = 251.46 Hz, C_7_), 137.29 (d, *J* = 257.31 Hz, C_8_), 131.43 (s, C_13_), 124.95 (s, C_5_), 116.47 (m, C_10_), 103.22 (s, C_11_). Elemental analysis (%) for **1·**ZnTPPF_5_ (C_147_H_36_F_60_N_15_BiZn_3_): Calcd: C 49.63, H 1.02, N 5.91. Found: C 49.2, H 1.1, N 5.6. HR-MS [ESI, positive ion mode ESI-TOF]: *m*/*z* for C_147_H_37_F_60_N_15_BiZn_3_ [**2**·ZnTPPF_5_ + H]^+^: Calcd: 3558.0066. Found: 3558.0037 (−2.9 ppm error).

## Computational Details

### General Methods

DFT calculations were carried out using the Gaussian 16 package^89^ using the hybrid method of Austin, Petersson and Frisch with spherical atom dispersion terms (APFD).^90^ The triple-ζ cc-pVTZ-PP basis set with effective core potentials was used for the heavy atoms (Zn, Sb, Bi),^91–94^ as found in the basis set exchange library,^95–97^ and 6–31G­(d,p) was used for the rest of the atoms. Assemblies **1**·ZnTPPF_5_ and **2**·ZnTPPF_5_ were also optimized using the Perdew, Burke and Ernzerhof (PBE) functional,^98,99^ and later hybridized by Adamo (PBE0)^100,101^ using Ahlrichs’ def2SVP basis set^100,101^ and the addition of Grimme’s GD3BJ empirical dispersion (see Cartesian coordinates in SI, pages S68–S70 and Figure S83).^102^

NBO analysis was performed with the program NBO 7.0 ^103^ and visualization of the NBO molecular orbitals was performed using the program Chemcraft.^104^ The analysis was carried out on both the X-ray diffraction and optimized structures (see Figures S84–S85 and S96–S97).

Quantum Theory of Atoms in Molecules (QTAIM)^105^ topology analysis was carried out using Multiwfn 3.8 ^106^ on the X-ray diffraction structures (see Figures S86–S87 and S98–S99).

Electrostatic potentials (ESP) were mapped over the electron density surface with an isovalue of ρ = 0.001 e/bohr^3^ using Multiwfn 3.8 ^106^ and the wave function files obtained after a single point DFT calculation at the same level of theory as described above. Surfaces were then plotted with VMD 1.9.2 ^107^ and colored according to an RGB scale (see pages S74–S78 and S81–S82). Further computational details can be found in the SI.

## Supplementary Material



## Notes

Additional experimental details, NMR spectra, high-resolution mass data, binding data analysis, DOSY experiments, X-ray crystallographic details, and computational calculation details (PDF)
