# Supplementary material for: Design Rules for Sb and Bi Porphyrin Capsules: Para-Substitution Effects and Pnictogen Bond Conformational Control
Source: Inorg Chem. 2026 Feb 12;65(8):4721–37. doi: 10.1021/acs.inorgchem.5c05993 (PMC12958297; doi:10.1021/acs.inorgchem.5c05993)
Supplement: Supplementary file 1 [file ic5c05993_si_001.pdf]

Supporting Information

Design Rules for Sb and Bi porphyrin capsules: Para-substitution Effects and Pnictogen Bond Conformational Control

Daniel Cubero-Pascual,[a] Álvaro García-Romero,*[a] Héctor Barbero*[a] and Raúl García-Rodríguez*[a]

[a] GIR MIOMeT-IU Cinquima-Química Inorgánica Facultad de Ciencias, Universidad de Valladolid; Campus Miguel Delibes, 47011 Valladolid (Spain). E-mail: raul.garcia.rodriguez@uva.es; hector.barbero@uva.es; alvaro.garcia.romero@uva.es.

Table of contents

Experimental Details **3**

Synthetic procedures *3*

Discussion of {[Sb(3-py)$_3$]$_2$·(MgTPPBr)$_3$} (1·MgTPPBr) *5*

NMR studies and spectra **7**

DOSY experiments *23*

Variable temperature experiments *27*

Binding data analysis *30*

Method of continuous variation (Job Plot)[12,13] *30*

^{1}H NMR Titrations *34*

X-ray crystallographic studies **49**

High-resolution mass data **57**

Computational details **68**

Effect of Remote Coordination on Pnictogen Bonding *68*

Optimized structures *69*

NBO analysis *72*

Topology analysis *74*

ESP maps *75*

Intermolecular pnictogen bond studies *79*

Catalytic tests **84**

Procedure for Sb-catalyzed oxidations of α-hydroxyketones. *84*

Experimental Details

Synthetic procedures

Synthesis of 5,10,15,20-tetrakis(4-methoxyphenyl)porphyrin (2HTPPOMe): A variation of a method described elsewhere was followed.[1] Pyrrole (486 µL, 4 mmol), 4-methoxybenzaldehyde (278 µL, 4 mmol), propionic acid (12 mL, 160 mmol), and nitrobenzene (7 mL, 68 mmol) were mixed in a sealed vessel specifically designed for microwave irradiation. The mixture was stirred inside a microwave reactor at 200 °C for 15 min. Methanol (20 mL) was added to the dark crude, which was then filtered using a Büchner funnel and washed with MeOH (3 × 100 mL). The obtained solid was placed in an oven and kept under reduced pressure (100 °C, overnight, 90 Torr) to remove residual nitrobenzene and finally give the dark purple solid. Yield: 271.9 mg (0.37 mmol, 37%). NMR data matches the reported values.

Synthesis of 5,10,15,20-tetrakis(4-bromophenyl)porphyrin (2HTPPBr): A variation of a method described elsewhere was followed.[2] Pyrrole (486 µL, 4 mmol), 4-methoxybenzaldehyde (740 mg, 4 mmol), propionic acid (12 mL, 160 mmol), and nitrobenzene (7 mL, 68 mmol) were mixed in a sealed vessel specifically designed for microwave irradiation. The mixture was stirred inside a microwave reactor at 200 °C for 15 min. Methanol (20 mL) was added to the dark crude, which was then filtered in a Büchner funnel and washed with MeOH (3 × 100 mL). The obtained solid was placed in an oven and kept under reduced pressure (100 °C, overnight, 90 Torr) to remove residual nitrobenzene and finally give the dark purple solid. Yield: 644 mg (0.69 mmol, 69 %). NMR data matches the reported values.

Synthesis of 5,10,15,20-tetrakis(pentafluorphenyl)porphyrin (2HTPPF$_5$): A variation of a method described elsewhere was followed.[3] Pyrrole (0.89 mL, 12.8 mmol), pentafluoro benzaldehyde (2 g, 10.2 mmol) and boron trifluoride diethyl etherate (160 µL, 1.24 mmol) were stirred in 200 mL dichloromethane for 16 h under a nitrogen atmosphere at room temperature. Then, *o*-chloranil (1 g, 4.04 mmol) was added, and the mixture was stirred for 3 h. The solvent was removed under reduced pressure. The crude product was purified by column chromatography (*n*-hexane/chloroform = 3:2, Rf= 0.60). A violet powder was obtained. Yield: 241.6 mg (0.248 mmol, 10%). NMR data matches the reported values.

Synthesis of ZnTPPOMe: A variation of a method described elsewhere was followed.[4] 2HTPPOMe (100 mg, 0.136 mmol), zinc acetate (249.53 mg, 1.36 mmol), $CHCl_3$ (3 mL, 37 mmol) and MeOH (1.5 mL, 37 mmol) were loaded in a sealed vessel specifically designed for microwave irradiation. The mixture was stirred inside a microwave reactor at 120 °C for 1 h 30 min. The solvent was removed under reduced pressure, and the obtained solid was dissolved in 100 mL of $CHCl_3$, placed into a separatory funnel, and washed with 100 × 3 mL of H_2O. The organic layer was removed under low pressure, resulting in a pink solid. Yield: 55.1 mg (0.069 mmol, 51%). NMR data matches the reported values.

Synthesis of ZnTPPBr: A variation of a method described elsewhere was followed.[2] 2HTPPBr (150 mg, 0.159 mmol), zinc acetate (291.73 mg, 1.59 mmol), $CHCl_3$ (3 mL, 37 mmol) and MeOH (1.5 mL, 37 mmol) were loaded in a sealed vessel specifically designed for microwave irradiation. The mixture was stirred inside a microwave reactor at 120 °C for 1 h. The solvent was removed under reduced pressure, and the obtained solid was dissolved in 100 mL of $CHCl_3$, placed into a separatory funnel, and washed with 100 × 3 mL of H_2O. The organic layer was removed under low pressure, resulting in a pink solid. Yield: 88.7 mg (0.0855 mmol, 83%). NMR data matches the reported values.

Synthesis of MgTPPBr: A variation of a method described elsewhere was followed.[5] 2HTPPBr (150 mg, 0.159 mmol), $MgCl_2{\cdot}6H_2O$ (327.2 mg, 1.61 mmol), K_2CO_3 (187.97 mg, 1.36 mmol) and DMF (5 mL, 37 mmol) were loaded in a sealed vessel specifically designed for microwave irradiation. The mixture was stirred inside a microwave reactor at 200 °C for 1 h. The solvent was removed under reduced pressure, and the obtained solid was dissolved in 100 mL of $CHCl_3$, placed into a separatory funnel, and washed with 100 × 3 mL of H_2O. The organic layer was removed under low pressure, resulting in a pink solid. Yield: 144.2 mg (0.151 mmol, 94%). NMR data matches the reported values.

Synthesis of ZnTPPF$_5$: A variation of a method described elsewhere was followed.[6] 2HTPPF$_5$ (100 mg, 0.103 mmol), zinc acetate (189 mg, 1.03 mmol), $CHCl_3$ (3 mL, 37 mmol) and MeOH (1.5 mL, 37 mmol) were loaded in a sealed vessel specifically designed for microwave irradiation. The mixture was stirred inside a microwave reactor at 150 °C for 1 h. The solvent was removed under reduced pressure, and the obtained solid was dissolved in 100 mL of $CHCl_3$, placed into a separatory funnel, and washed with 100 × 3 mL of H_2O. The organic layer was removed under low pressure, resulting in a pink solid. Yield: 88.7 mg (0.0855 mmol, 83%). NMR data matches the reported values.

Discussion of {[Sb(3-py)$_3$]$_2$·(MgTPPBr)$_3$} (1·MgTPPBr)

Our previous studies of the coordination of E(3-py)$_3$ (E = Sb and Bi) ligands to the aryl-unsubstituted metalloporphyrin MgTPPH revealed the formation of two-dimensional polymeric structures {[E(3-py)$_3$]$_2$·(MgTPPH)$_3$}$_n$ with a 2:3 ligand:porphyrin stoichiometry. In these structures, each ligand is coordinated via its three pyridinic arms to three MgTPPH fragments, and each MgTPPH is coordinated diaxially by two N-donor atoms of different ligands to form an extended 2D structure in which the integrity of the monomeric capsule structure is retained. To investigate the effect of substitution at the para position of the phenyl group of the magnesium metalloporphyrin on these extended structures, antimony ligand **1** and the Br-substituted magnesium porphyrin (MgTPPBr) were selected.

The reaction was carried out in $CHCl_3$ with a 2:3 ligand:porphyrin ratio based on the expected stoichiometry. ^{1}H NMR indicated axial binding of **1** to MgTPPBr. Slow diffusion of *n*-hexane into a $CHCl_3$ solution of the ligand **1** (2 equiv.) and MgTPPBr (3 equiv.) gave crystals of {[Sb(3-py)$_3$]$_2$·(MgTPPBr)$_3$} (**1**·MgTPPBr) in 15% yield (Scheme 1). Single-crystal X-ray diffraction of **1**·MgTPPBr revealed the expected 2:3 stoichiometry; however, rather than forming a 2D polymeric structure like that previously observed for {[Sb(3-py)$_3$]$_2$·(MgTPPH)$_3$}$_n$,[7] **1**·MgTPPBr was present as a discrete heterobimetallic Mg(II)/Sb(III) complex composed of two Sb(3-py)$_3$ template ligand units and three MgTPPBr metalloporphyrins (Fig. S1). Importantly, in this discrete polymetallic Sb_2Mg_3 chain structure, each ligand **1** coordinates only two MgTPPBr porphyrins, with the third pyridyl arm remaining uncoordinated, paralleling the observations for the Zn metalloporphyrins. **1**·MgTPPBr features two terminal and one central MgTPPBr with the central Mg coordinated by both ligands; thus, it can be viewed as two semi-capsules that share the central MgTPPBr unit. The terminal monoaxial Mg^{2+} centers show shorter Mg–N distances (2.173(5) Å) than the diaxially coordinated central Mg^{2+} (2.300(5) Å), reflecting the difference between five- and six-coordinate environments in the metalloporphyrins. The coordination of **1** to only two MgTPPBr molecules was also confirmed by ESI-TOF high-resolution mass-spectrometry, which showed an $[M+H]^+$ peak at *m*/*z* 2283.7112 (calcd. 2283.7080) for {[Sb(3-py)$_3$]·(MgTPPBr)$_2$}. Notably, in contrast to the Zn(II)TPPX (X = Br, OMe)-based semi-capsules described above, the solid-state structure of **1**·MgTPPBr is not preserved in solution. ^{1}H DOSY NMR experiments in $CDCl_3$ reveal that the pentameric Sb_2Mg_3 complex undergoes disaggregation into smaller complexes, reflecting the weaker coordination of the ligand to the Mg metalloporphyrin (see section DOSY experiments).

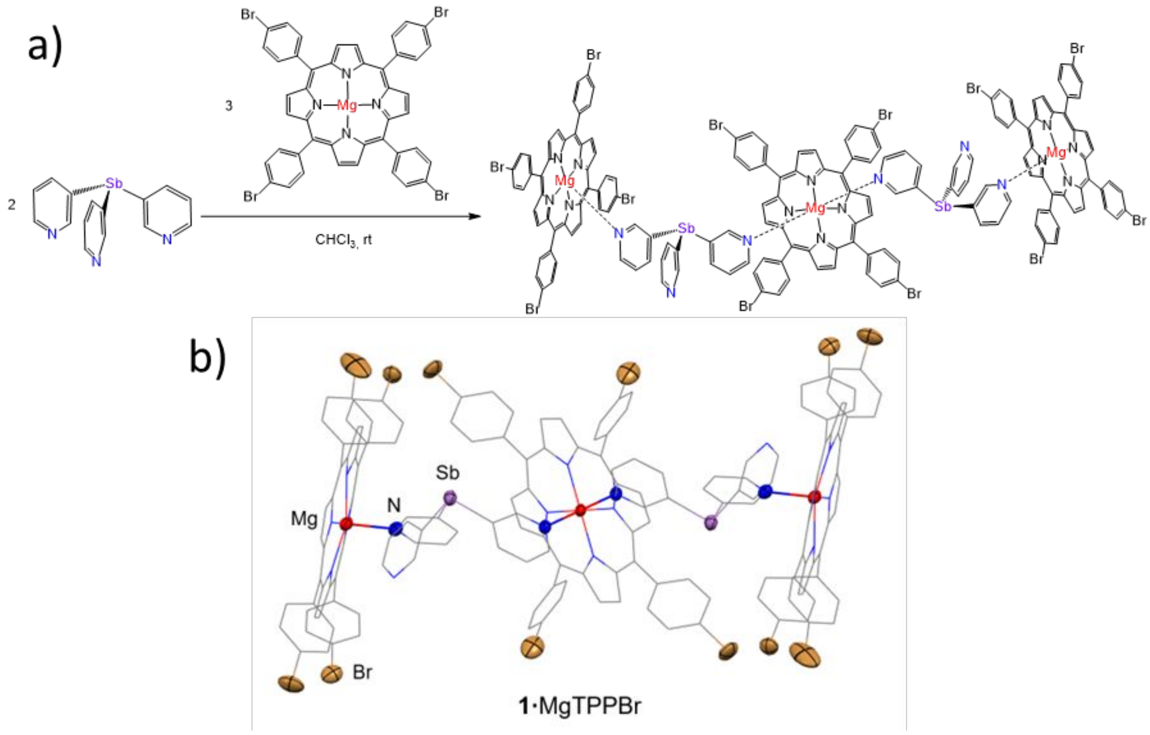

Figure S1. a) Synthesis and b) X-ray structures of the complex {[Sb(3-py)$_3$]$_2$·(MgTPPBr)$_3$} (**1**·MgTPPBr). In (b), displacement ellipsoids are shown at 50% probability. Solvent molecules and H-atoms are omitted for clarity. Selected bond lengths (Å) and angles (°): **1**·MgTPPBr, Sb–C$_{py}$ range 2.136(7)–2.145(5); N$_{py}$–Mg range 2.173(5)–2.300(5); C$_{py}$–Sb–C$_{py}$ range 92.6(2)–97.7(2). Color key: C (grey), Mg (red), N (blue), Br (brown), Sb (light purple).

NMR studies and spectra

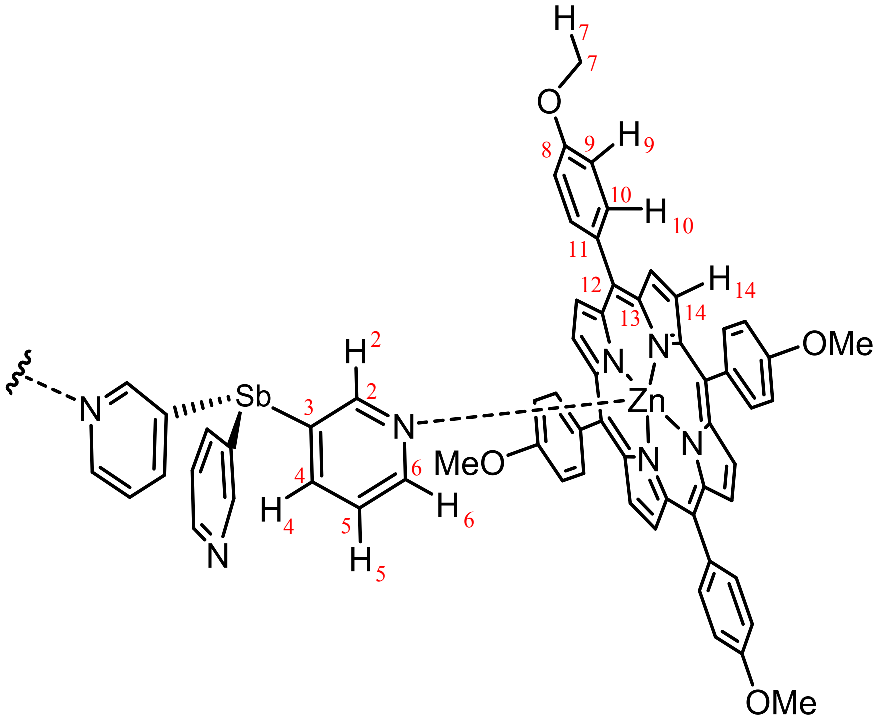

Figure S2. Compound **1**·ZnTPPOMe, {[Sb(3-py)$_3$]·(ZnTPPOMe)$_2$}, with the atom labelling used in the NMR studies.

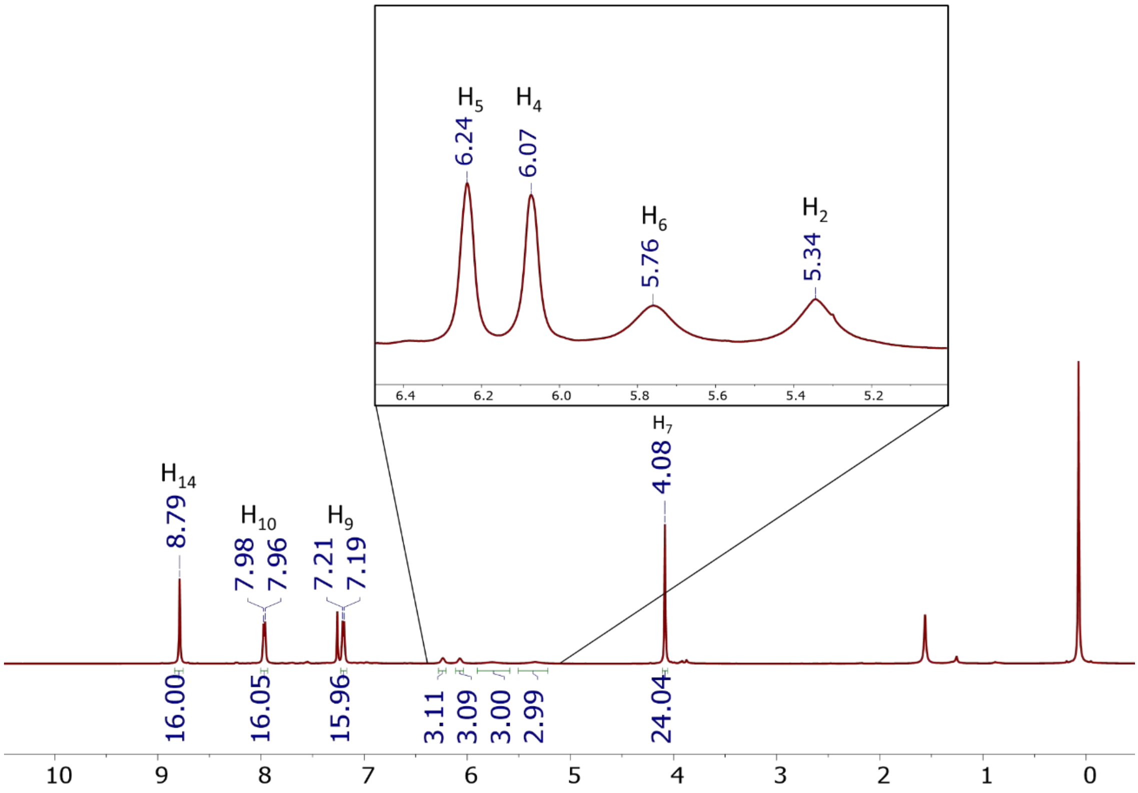

Figure S3. ^{1}H NMR (298 K, CDCl$_3$, 400 MHz) spectrum of **1**·ZnTPPOMe.

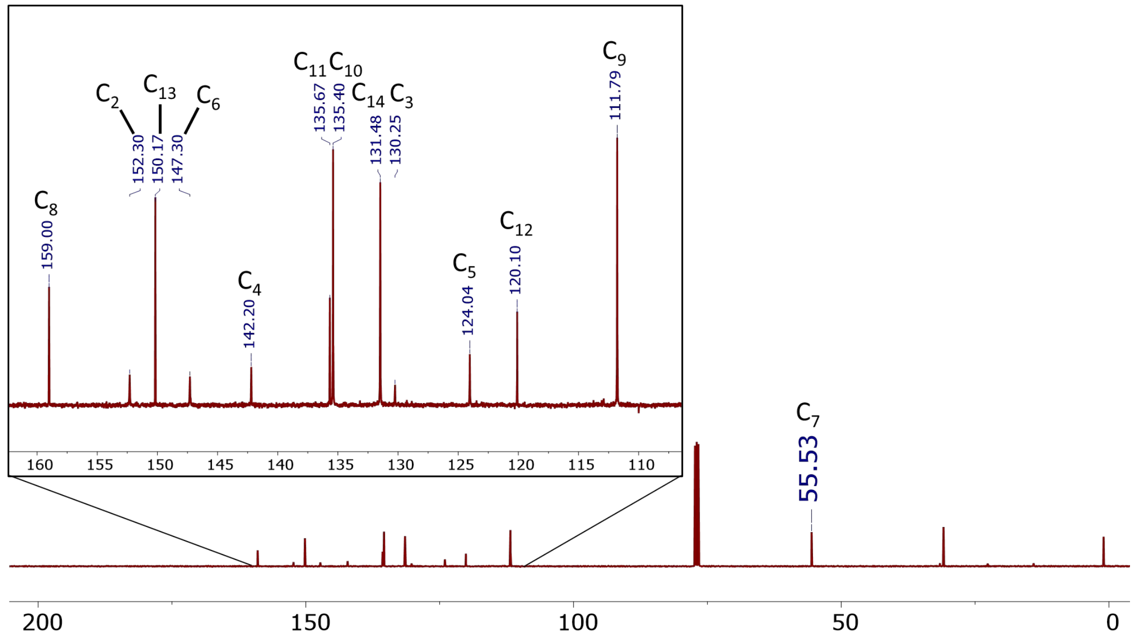

Figure S4. ^{13}C {^{1}H} NMR (298 K, $CDCl_3$, 100.25 MHz) spectrum of **1**·ZnTPPOMe.

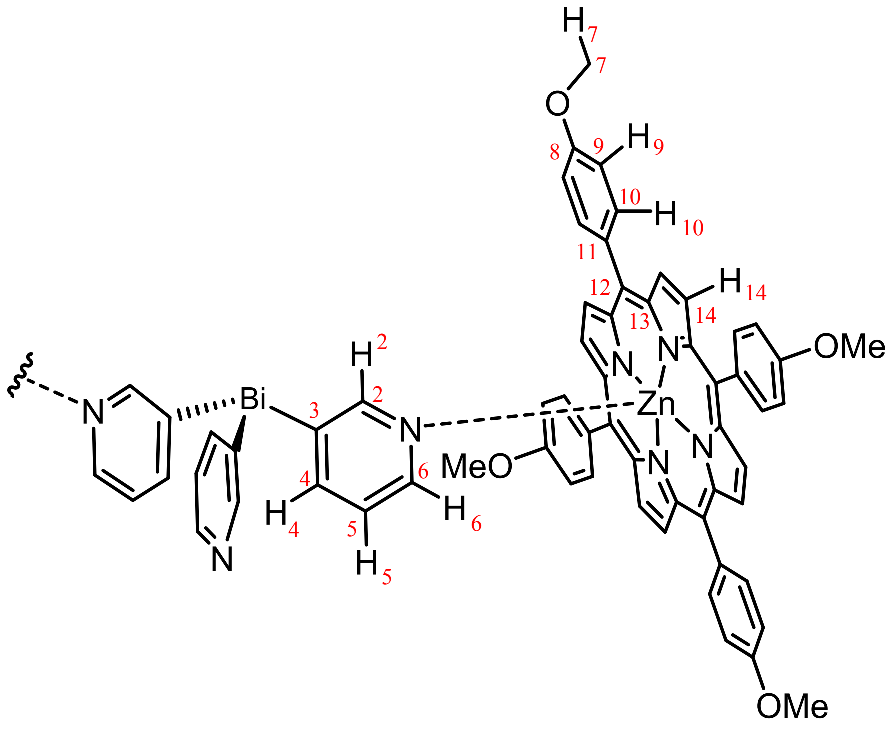

Figure S5. Compound **2**·ZnTPPOMe, {[Bi(3-py)$_3$]·(ZnTPPOMe)$_2$}, with the atom labelling used in the NMR studies.

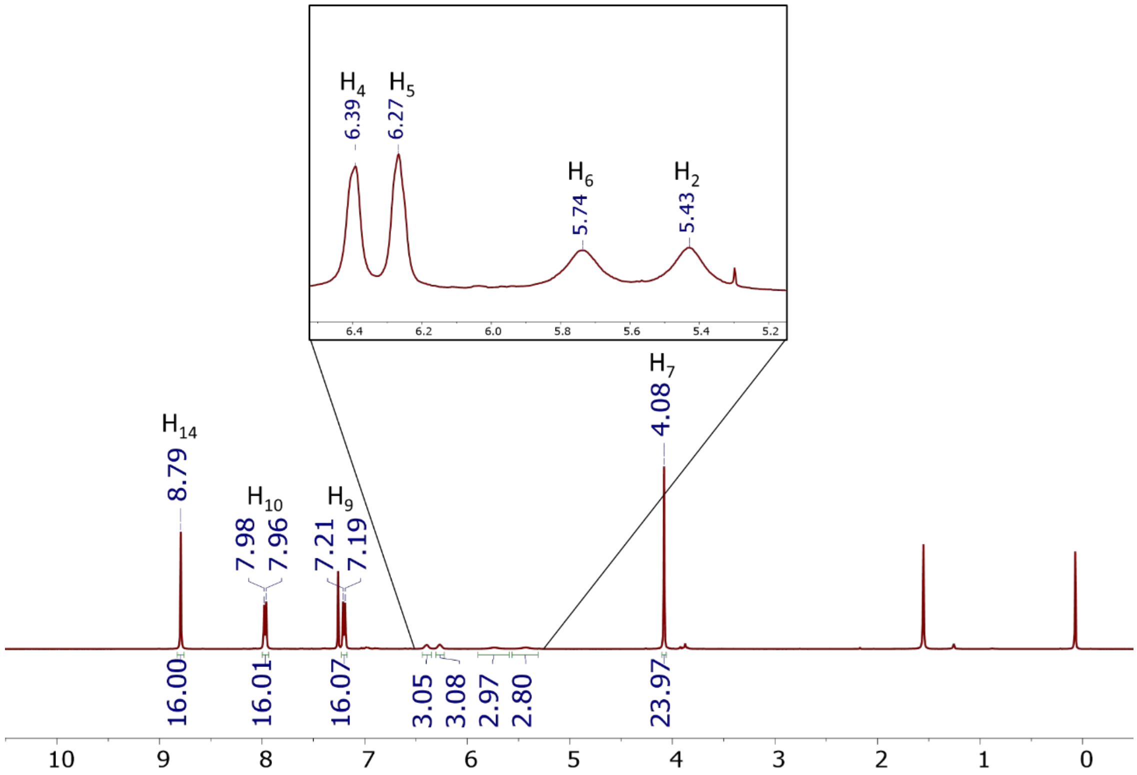

Figure S6. ^{1}H NMR (298 K, CDCl$_3$, 400 MHz) spectrum of **2**·ZnTPPOMe.

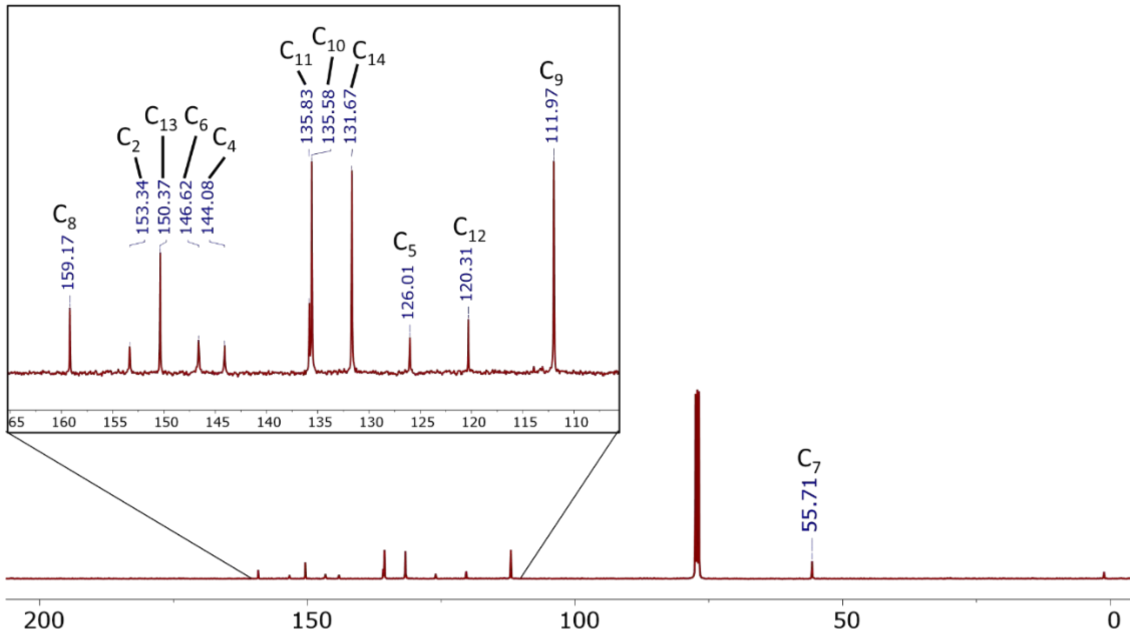

Figure S7. $^{13}C\ \{^{1}H\}$ NMR (298 K, $CDCl_3$, 100.25 MHz) spectrum of **2**·ZnTPPOMe.

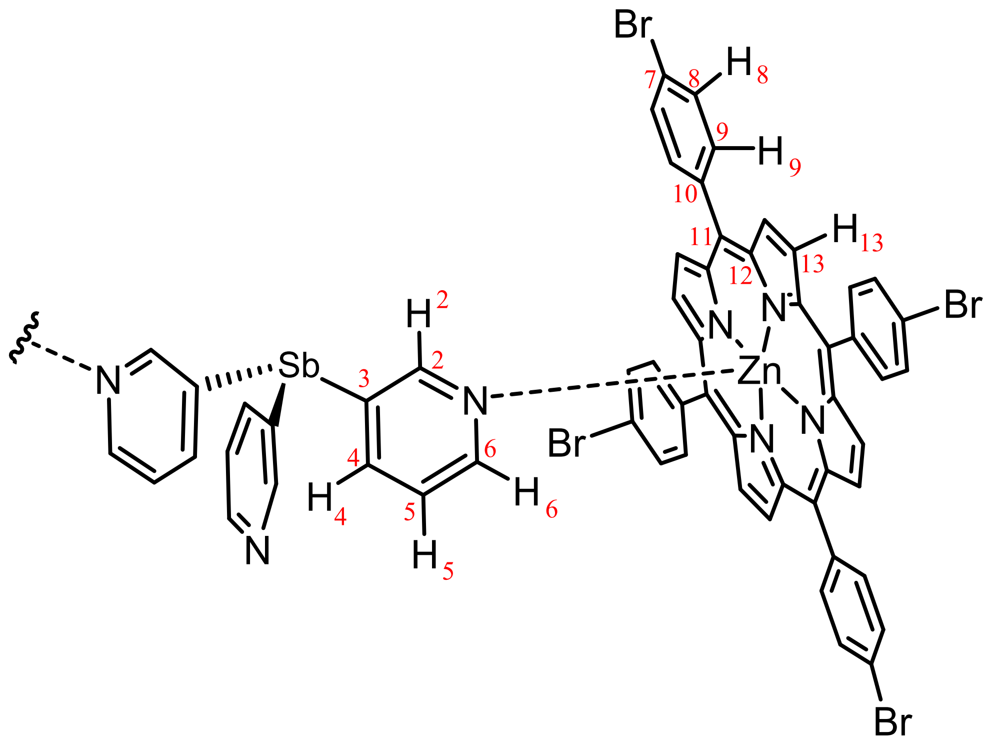

Figure S8. Compound **1**·ZnTPPBr, {[Sb(3-py)$_3$]·(ZnTPPBr)$_2$}, with the atom labelling used in the NMR studies.

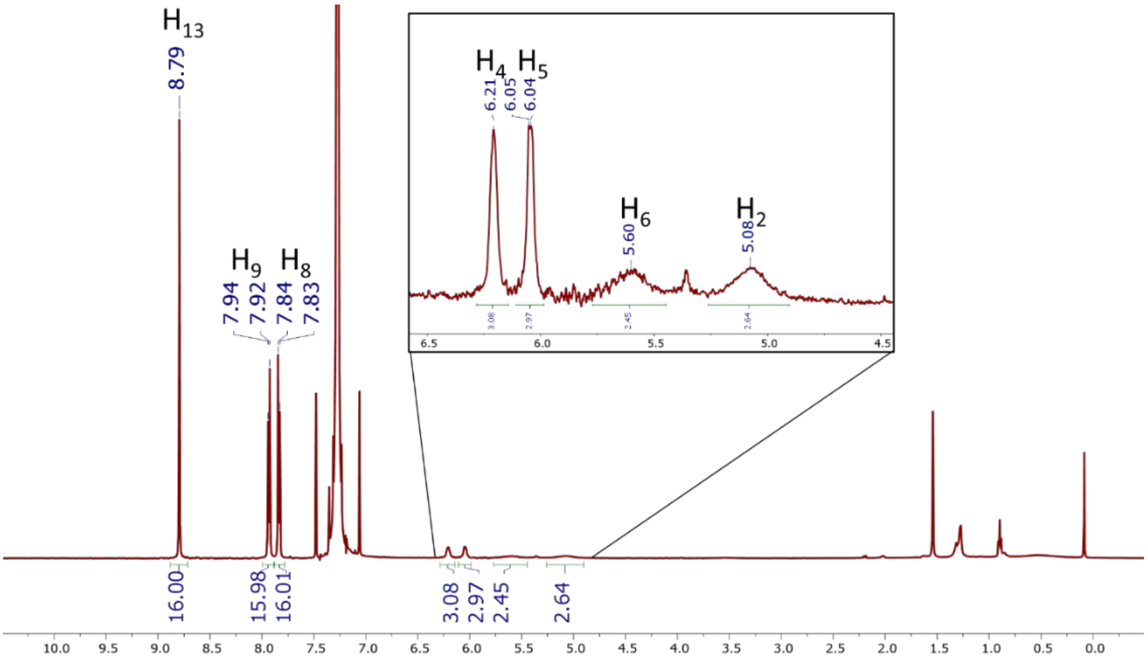

Figure S9. ^{1}H NMR (298 K, $CDCl_3$, 500 MHz) spectrum of **1**·ZnTPPBr.

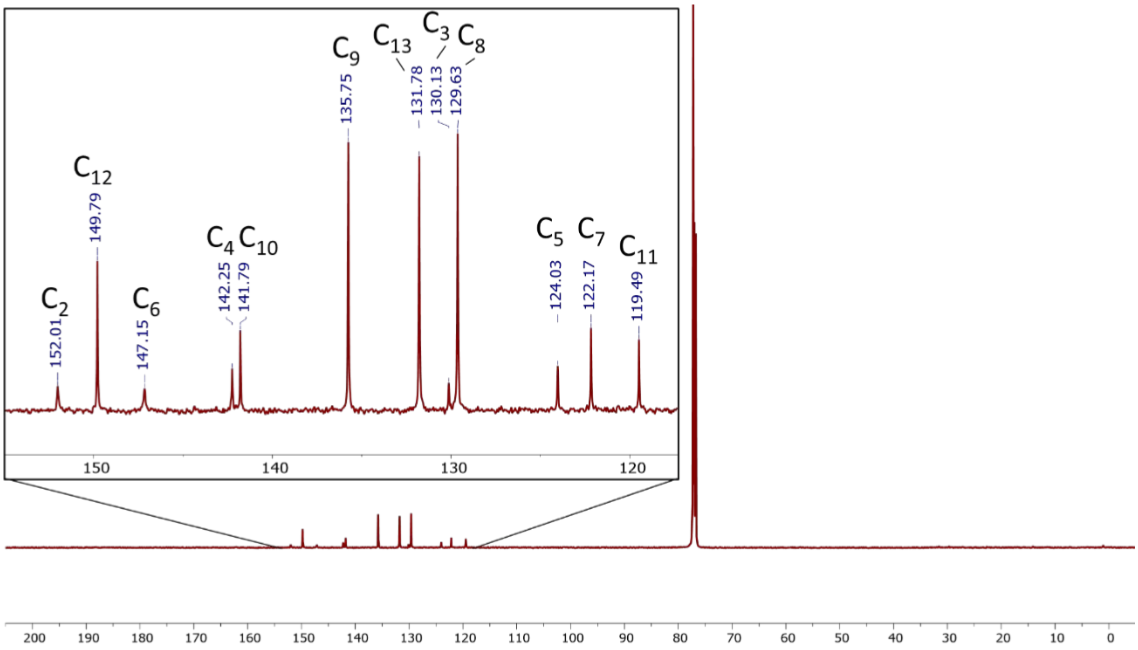

Figure S10. $^{13}C\{^{1}H\}$ NMR (298 K, $CDCl_3$, 125.67 MHz) spectrum of **1**·ZnTPPBr.

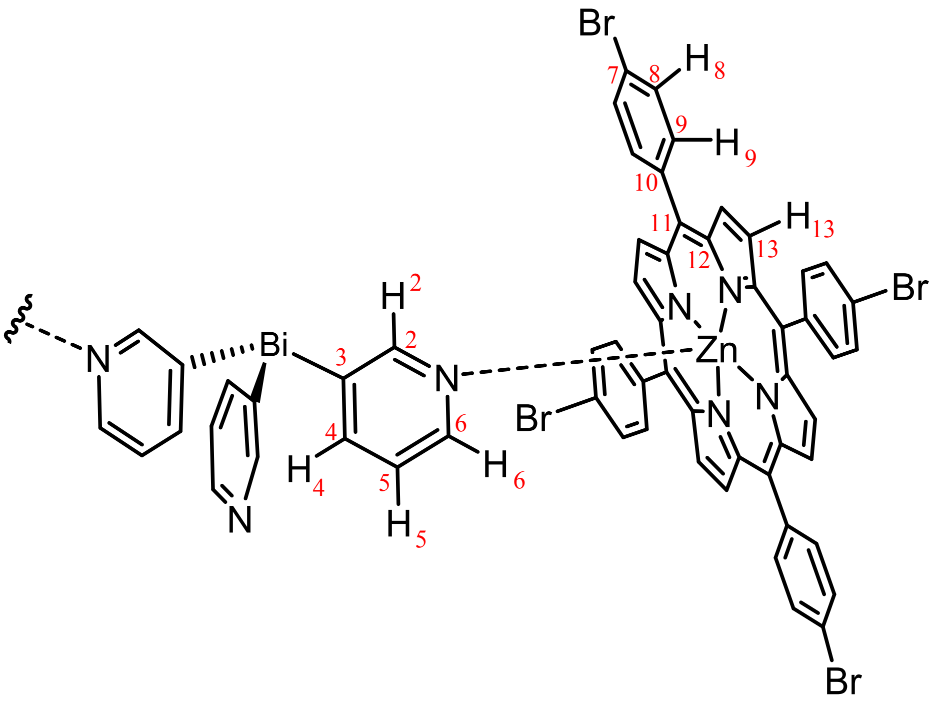

Figure S11. Compound **2**·ZnTPPBr, {Bi(3-py)$_3$·(ZnTPPBr)$_2$}, with the atom labelling used in the NMR studies.

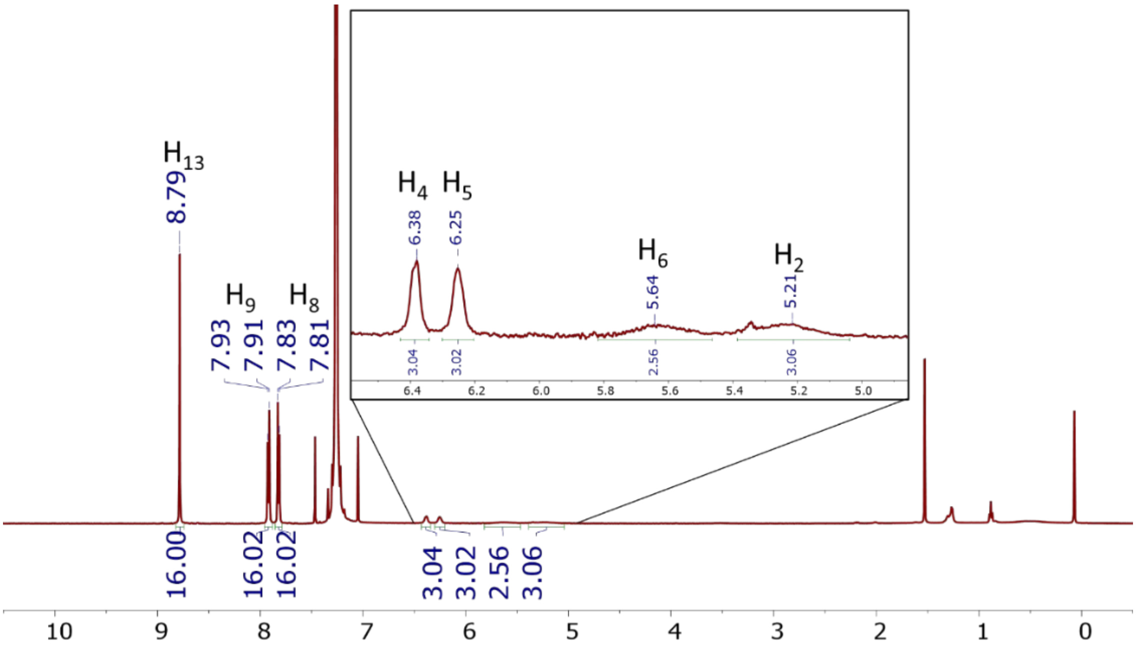

Figure S12. ^{1}H NMR (298 K, $CDCl_3$, 500 MHz) spectrum of **2**·ZnTPPBr.

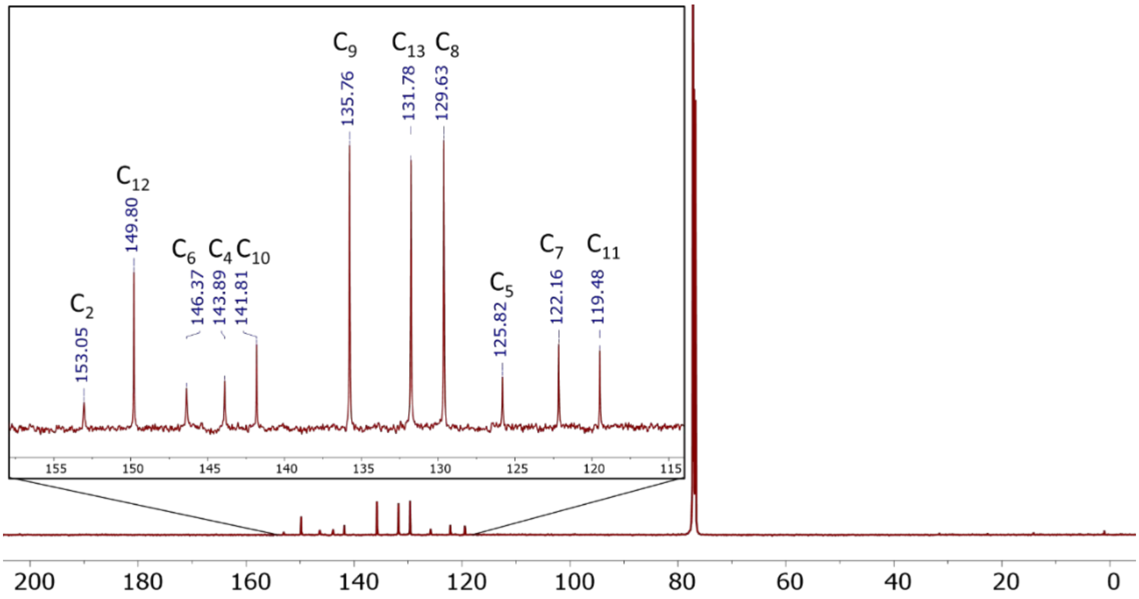

Figure S13. $^{13}C\ \{^{1}H\}$ NMR (298 K, $CDCl_3$, 125.67 MHz) spectrum of **2**·ZnTPPBr.

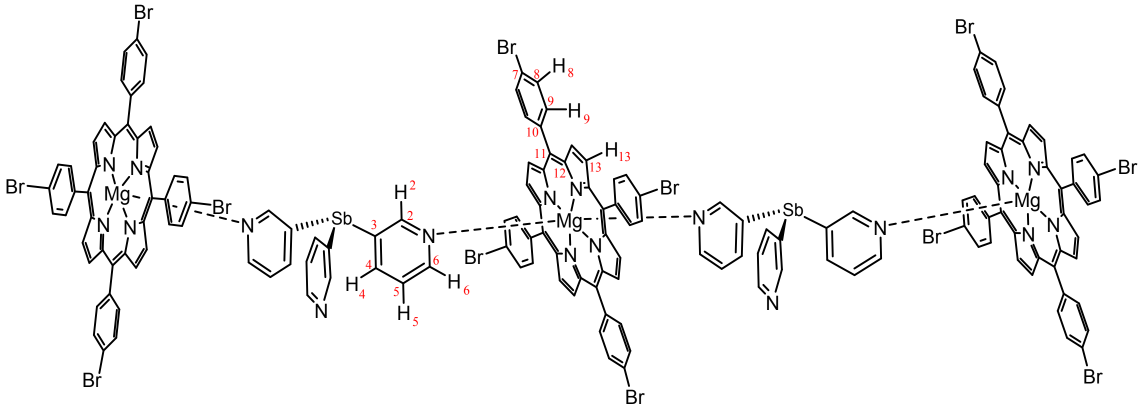

Figure S14. Compound **1**·MgTPPBr, {[Sb(3-py)3]$_2$·(ZnTPPBr)$_3$}, with the atom labelling used in the NMR studies.

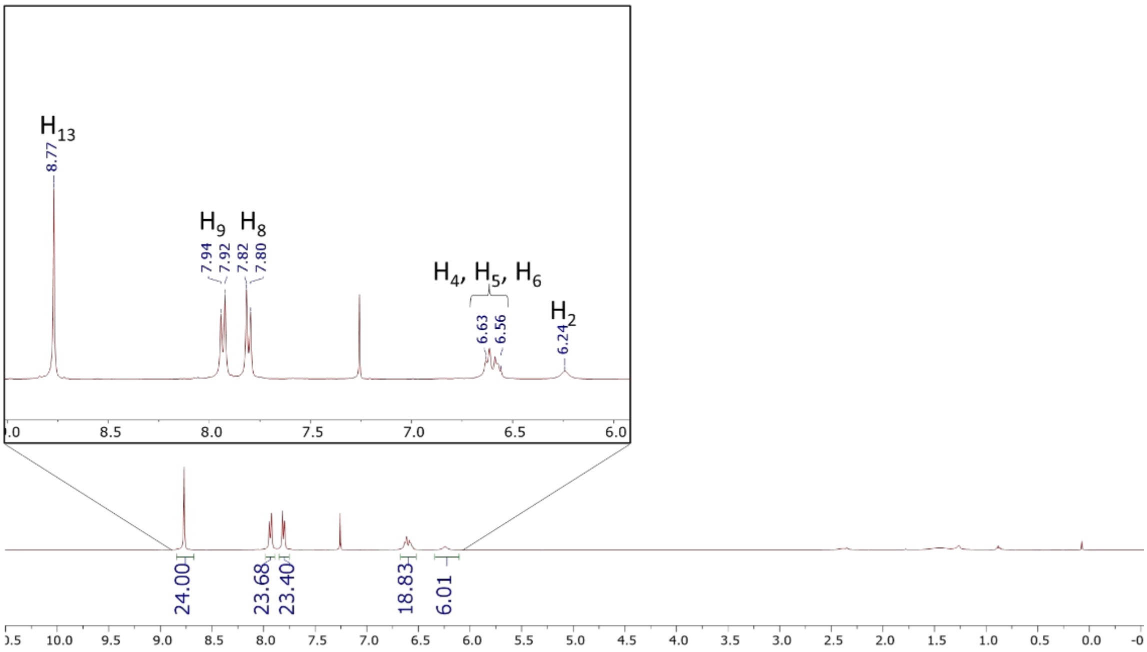

Figure S15. ^{1}H NMR (298 K, $CDCl_3$, 400 MHz) spectrum of **1**·MgTPPBr.

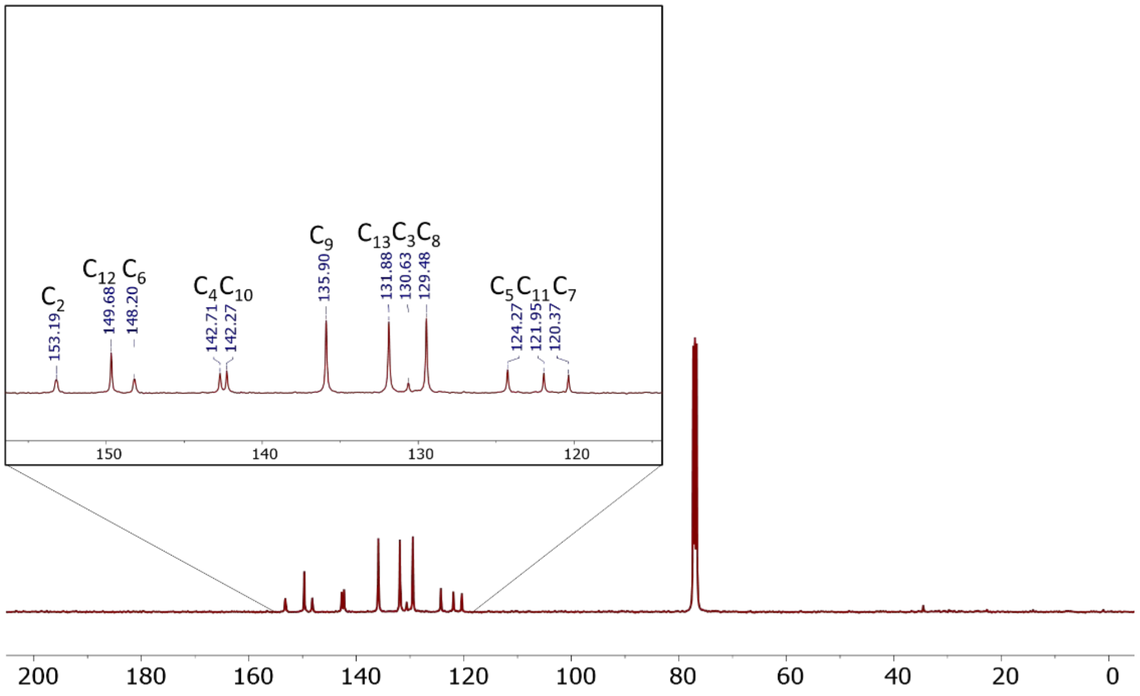

Figure S16. ^{13}C {^{1}H} NMR (298 K, $CDCl_3$, 100.25 MHz) spectrum of **1**·MgTPPBr.

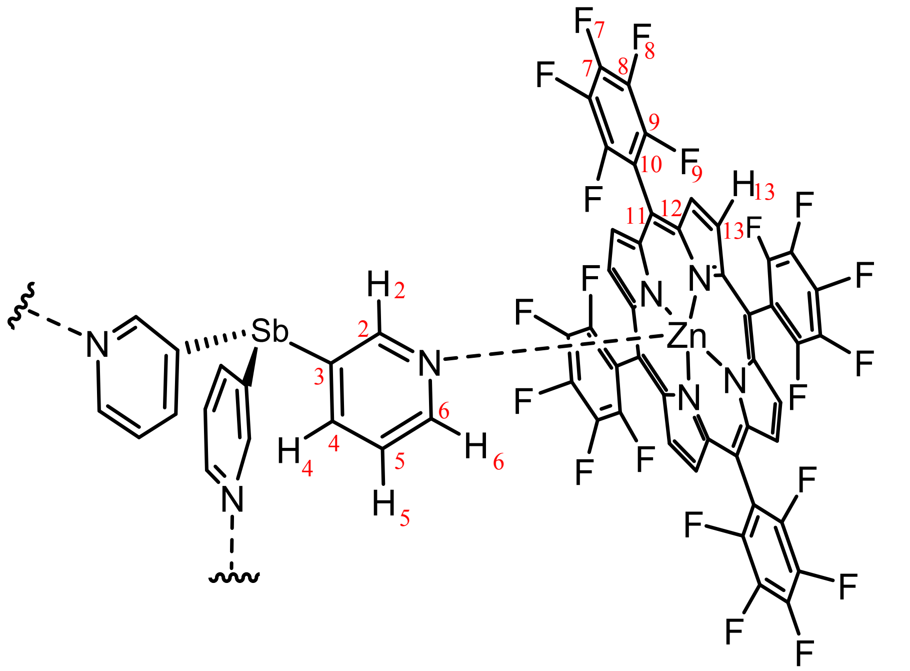

Figure S17. Compound **1**·ZnTPPF$_5$, {[Sb(3-py)$_3$]·(ZnTPPF$_5$)$_3$}, with the atom labelling used in the NMR studies.

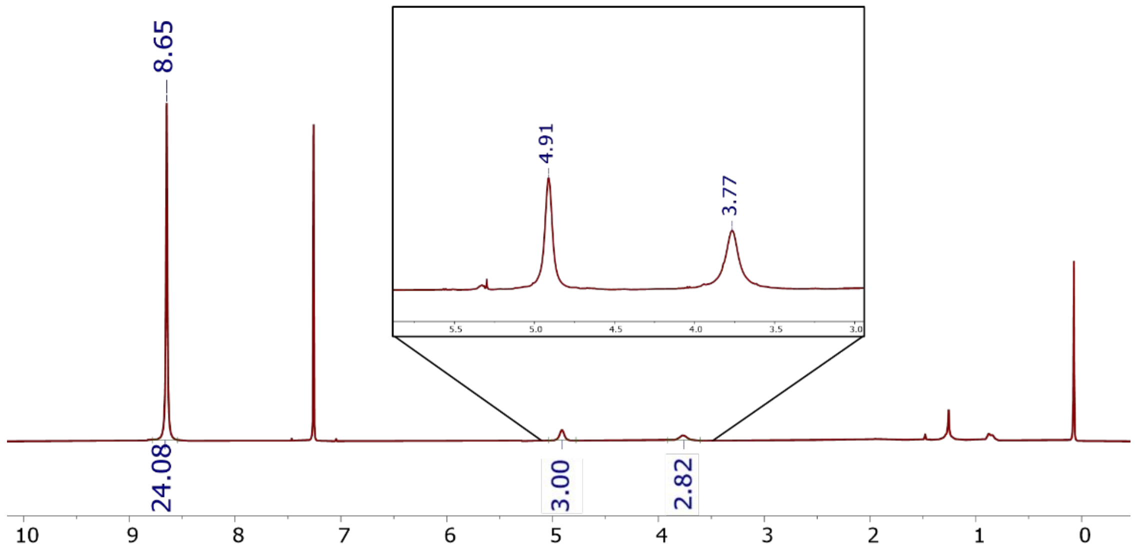

Figure S18. ^{1}H NMR (298 K, $CDCl_3$, 500 MHz) spectrum of **1**·ZnTPPF$_5$.

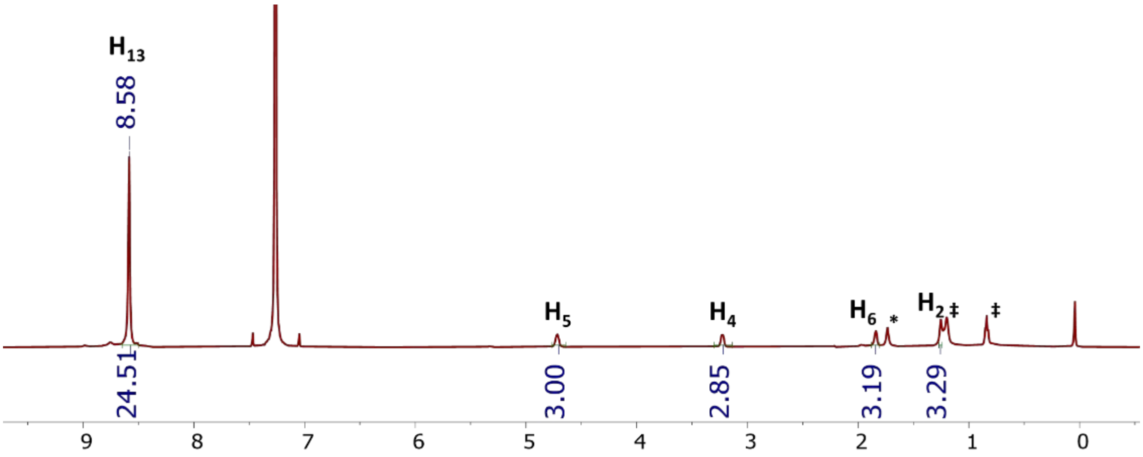

Figure S19. ^{1}H NMR (213 K, $CDCl_3$, 500 MHz) spectrum of **1**·$ZnTPPF_5$. Residual H_2O (*) and hexane (‡) are present.

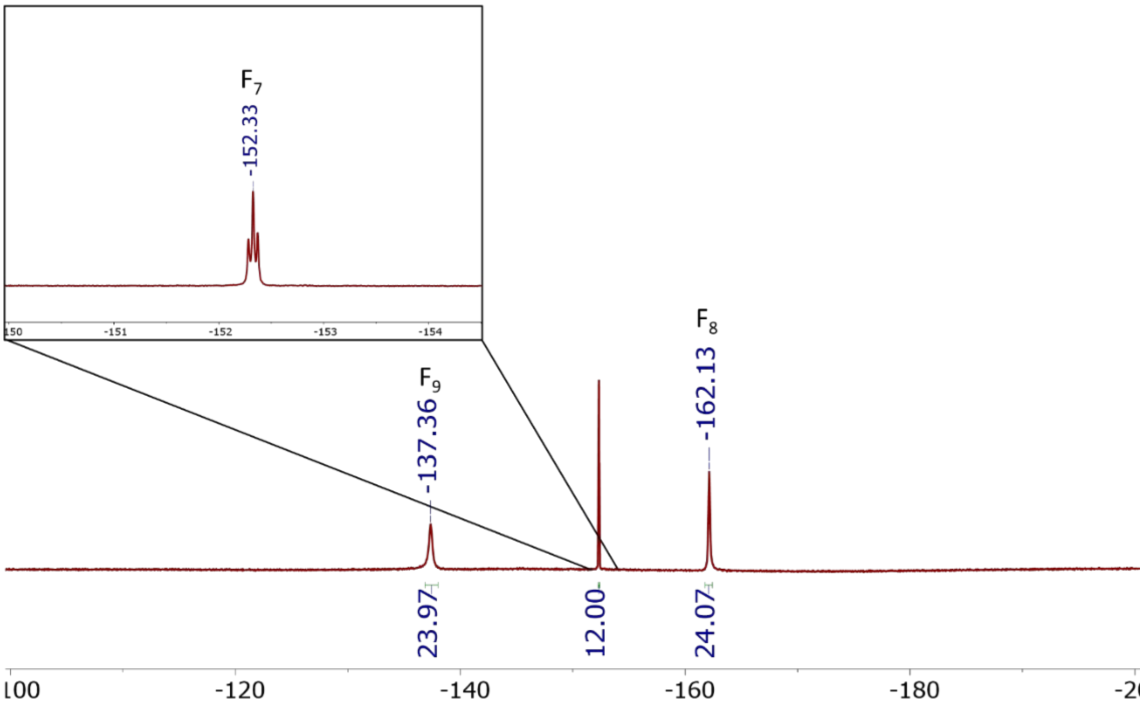

Figure S20. ^{19}F NMR (298 K, $CDCl_3$, 470.17 MHz) spectrum of **1**·$ZnTPPF_5$.

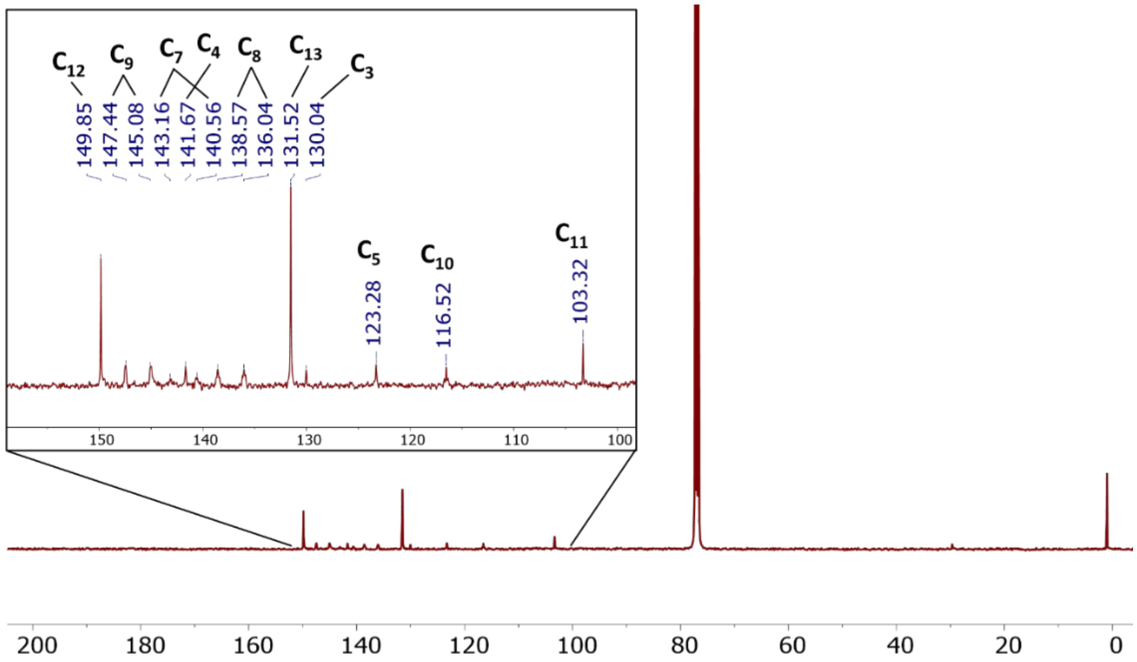

Figure S21. $^{13}C\{^{1}H\}$ NMR (298 K, $CDCl_3$, 470.17 MHz) spectrum of **1**·$ZnTPPF_5$.

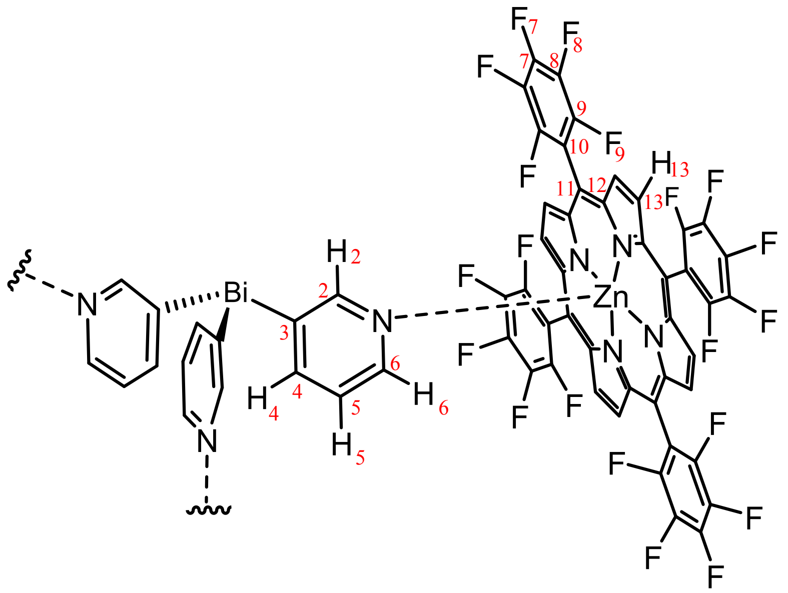

Figure S22. Compound **2**·ZnTPPF$_5$, {[Bi(3-py)$_3$]·(ZnTPPF$_5$)$_3$}, with the atom labelling used in the NMR studies.

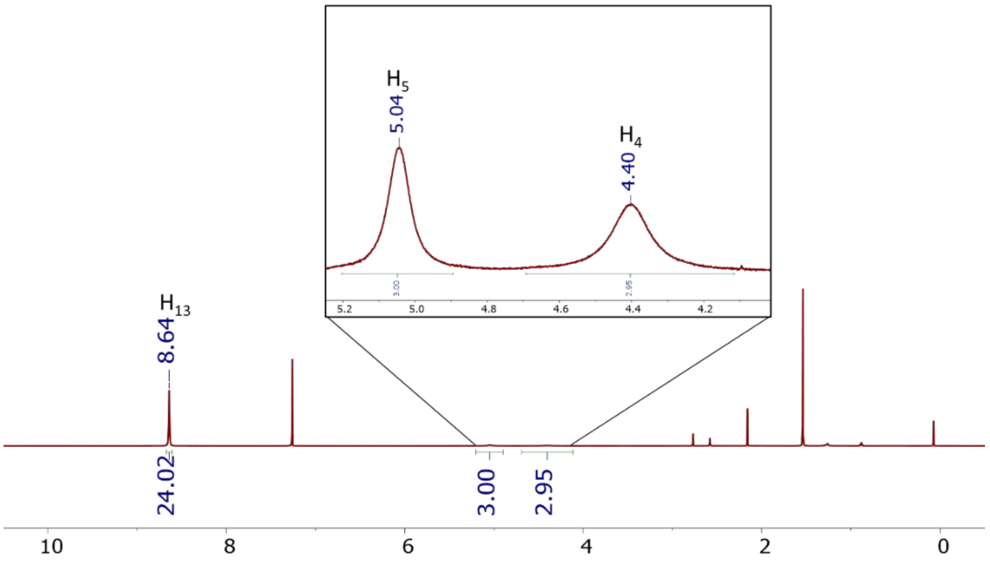

Figure S23. ^{1}H NMR (298 K, CDCl$_3$, 500 MHz) spectrum of **2**·ZnTPPF$_5$.

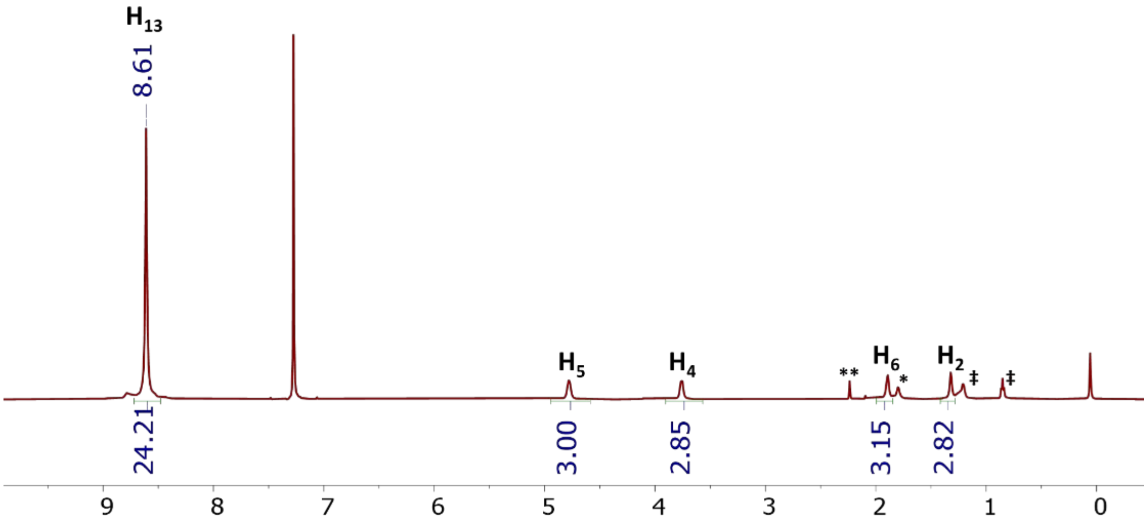

Figure S24. ^{1}H NMR (213 K, $CDCl_3$, 500 MHz) spectrum of **2**·ZnTPPF$_5$. Residual H_2O (*), acetone (**) and hexane (‡) are present.

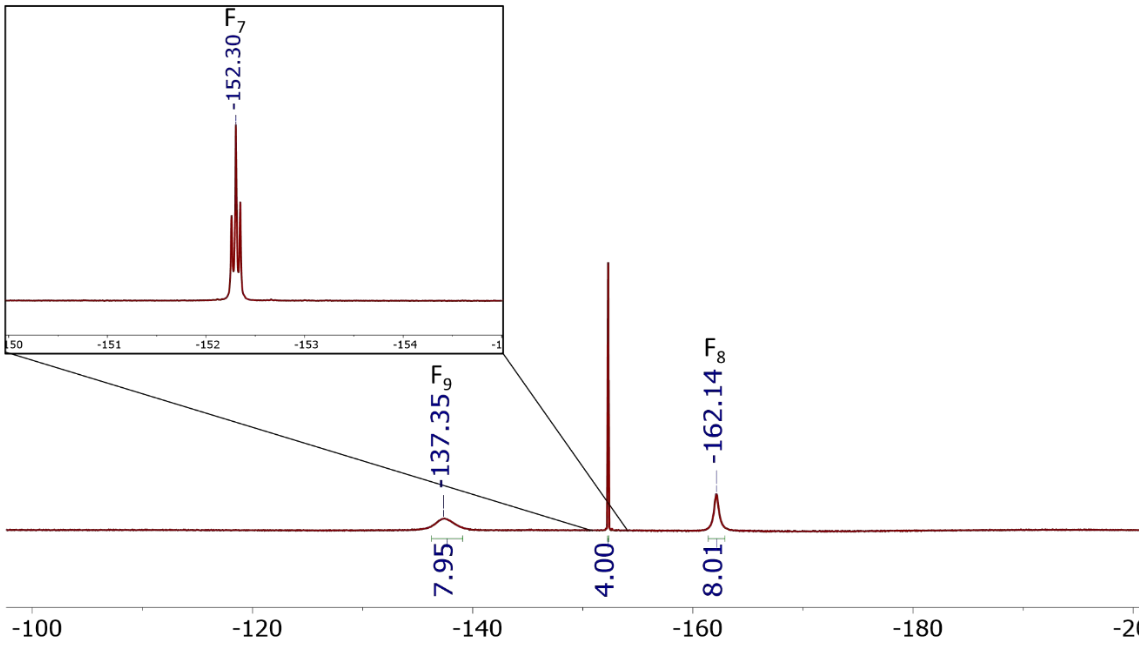

Figure S25. ^{19}F NMR (298 K, $CDCl_3$, 470.17 MHz) spectrum of **2**·ZnTPPF$_5$.

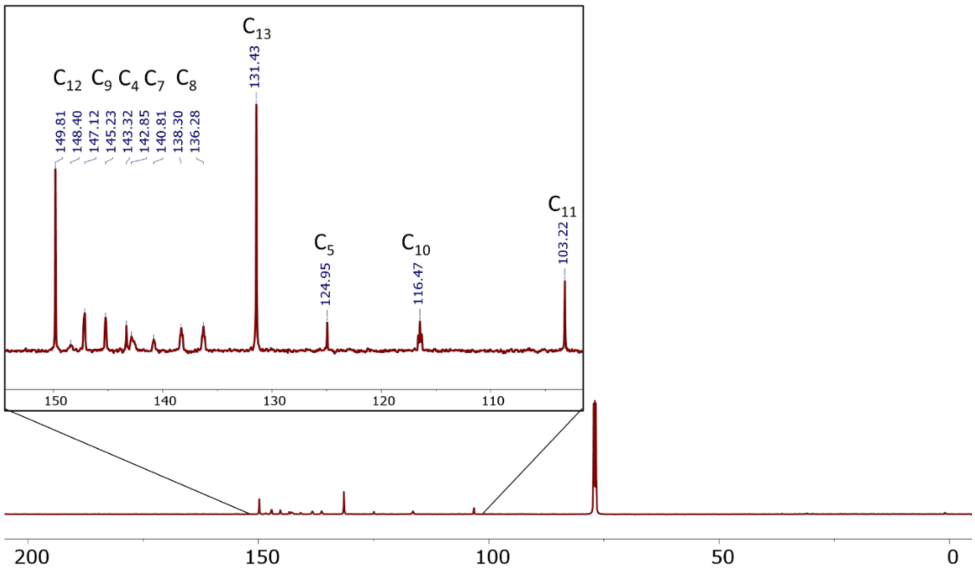

Figure S26. ^{13}C {1H} NMR (298 K, $CDCl_3$, 125.67 MHz) spectrum of 2·ZnTPPF$_5$.

DOSY experiments

[1]H-DOSY experiments were performed to estimate the hydrodynamic radii of the complexes **1**·ZnTPPOMe, **2**·ZnTPPOMe, **1**·ZnTPPBr, **2**·ZnTPPBr, **1**·ZnTPPF$_5$ and **2**·ZnTPPF$_5$ in $CDCl_3$ solution (**Figures S27–S29**).[8]

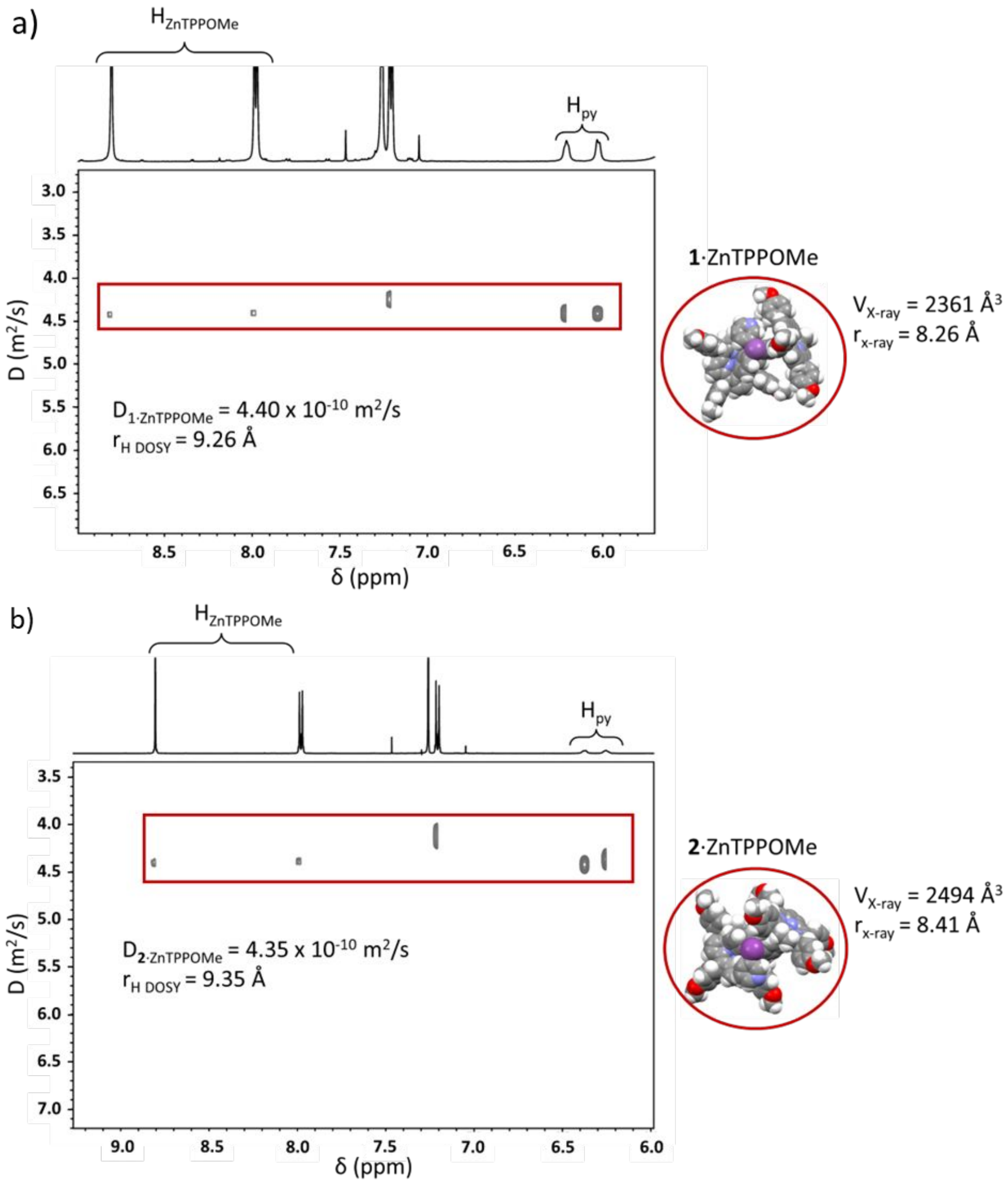

Figure S27. ^{1}H DOSY NMR spectra of (a) **1**·ZnTPPOMe and (b) **2**·ZnTPPOMe at 298 K. The DOSY NMR spectra show that all proton resonances present the same diffusion coefficient within experimental error in $CDCl_3$. The hydrodynamic radii calculated from the diffusion coefficient using the Stokes–Einstein equation ($r_{H\ DOSY}$) are 9.26 Å (a) and 9.35 Å (b). For both compounds, the $r_{H\ DOSY}$ value is slightly greater than the crystallographic radius.

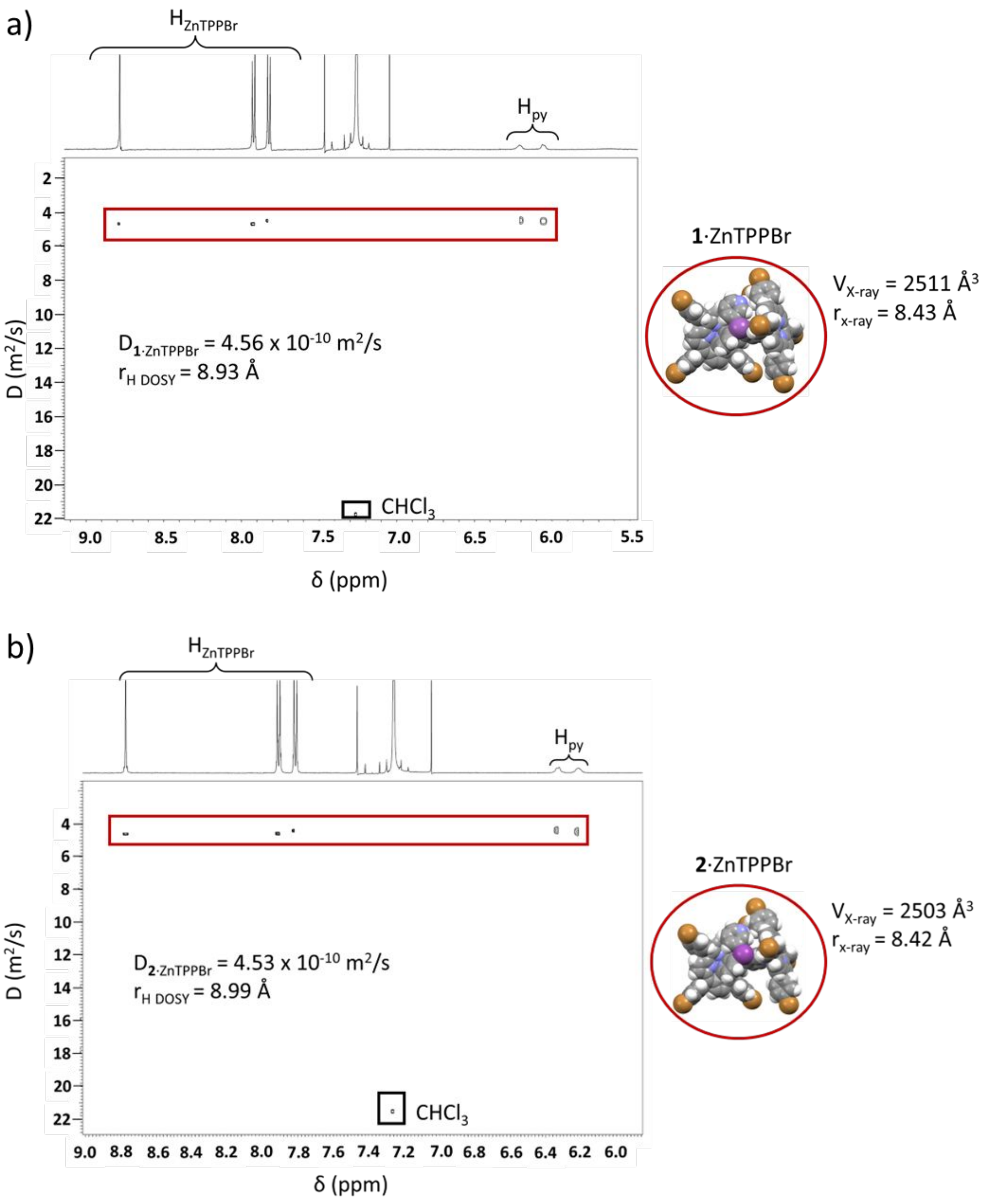

Figure S28. ^{1}H DOSY NMR spectra of (a) **1**·ZnTPPBr and (b) **2**·ZnTPPBr at 298 K. The DOSY NMR spectra show that all proton resonances present the same diffusion coefficient within experimental error in $CDCl_3$. The hydrodynamic radii calculated from the diffusion coefficient using the Stokes–Einstein equation ($r_{H\ \mathrm{DOSY}}$) are 8.93 Å (a) and 8.99 Å (b). For both compounds, the $r_{\mathrm{H\ DOSY}}$ value is slightly greater than the crystallographic radius.

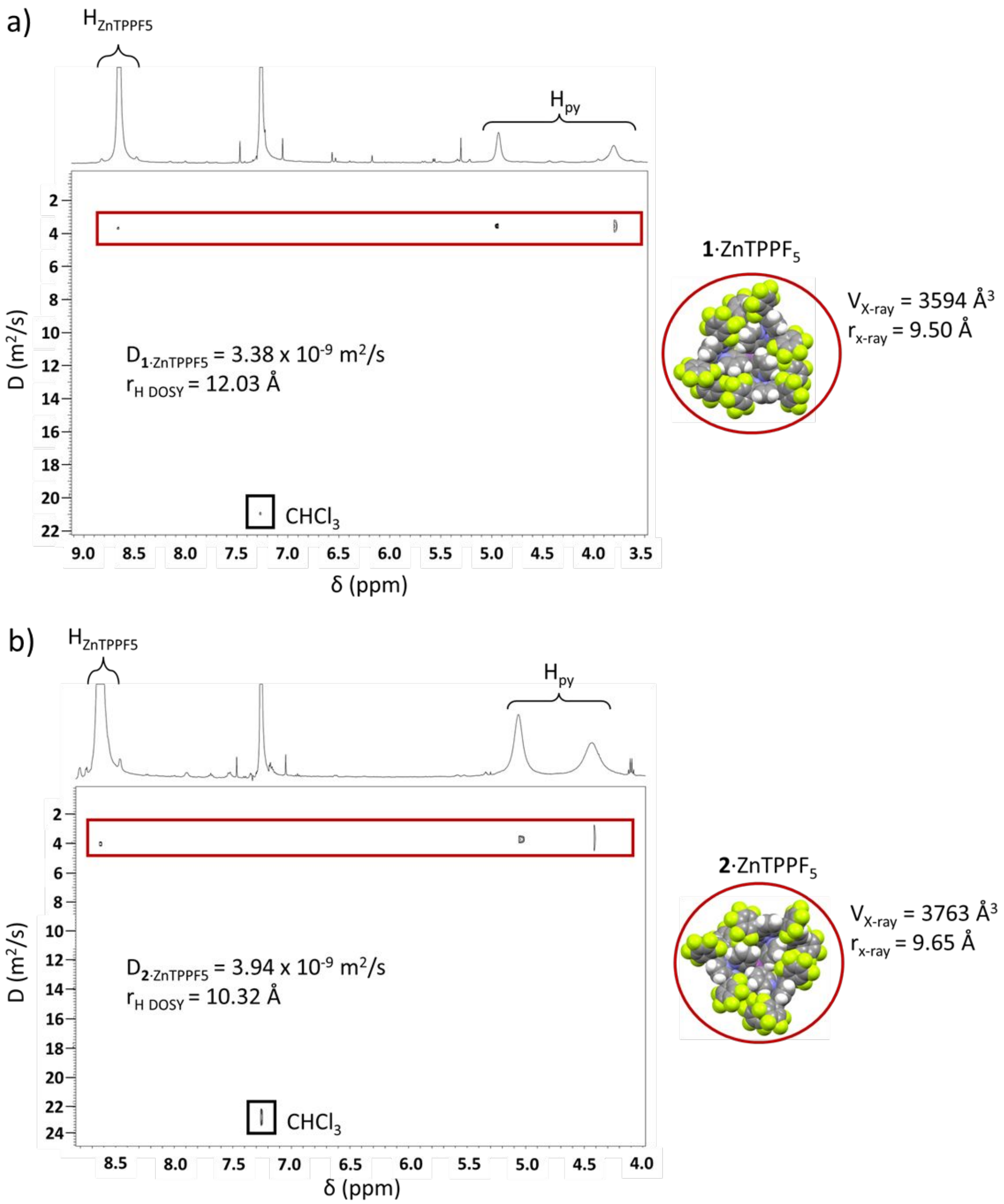

Figure S29. ^{1}H DOSY NMR spectra of (a) **1**·ZnTPPF$_5$ and (b) **2**·ZnTPPF$_5$ at 298 K. The DOSY NMR spectra show that all proton resonances present the same diffusion coefficient within experimental error in CDCl$_3$. The hydrodynamic radii calculated from the diffusion coefficient using the Stokes–Einstein equation ($r_{H\ \mathrm{DOSY}}$) are 12.03 Å (a) and 10.32 Å (b). For both compounds, the $r_{H\ \mathrm{DOSY}}$ value is slightly greater than the crystallographic radius. Please note that the NMR chemical shifts for the compounds are concentration dependent and therefore small variations depending on the conditions can be expected.

In order to evaluate the aggregation state of the complex **1**·MgTPPBr in solution, we estimated its MW via ^{1}H DOSY NMR using the Stalke method (ECC_{DSE}^{CDCl3}). The ^{1}H DOSY-ECC-MW experiment was carried out in $CDCl_3$ at 298 with adamantane as an internal reference, and the data was analyzed with the MW Estimation Software developed by Stalke.[9–11]

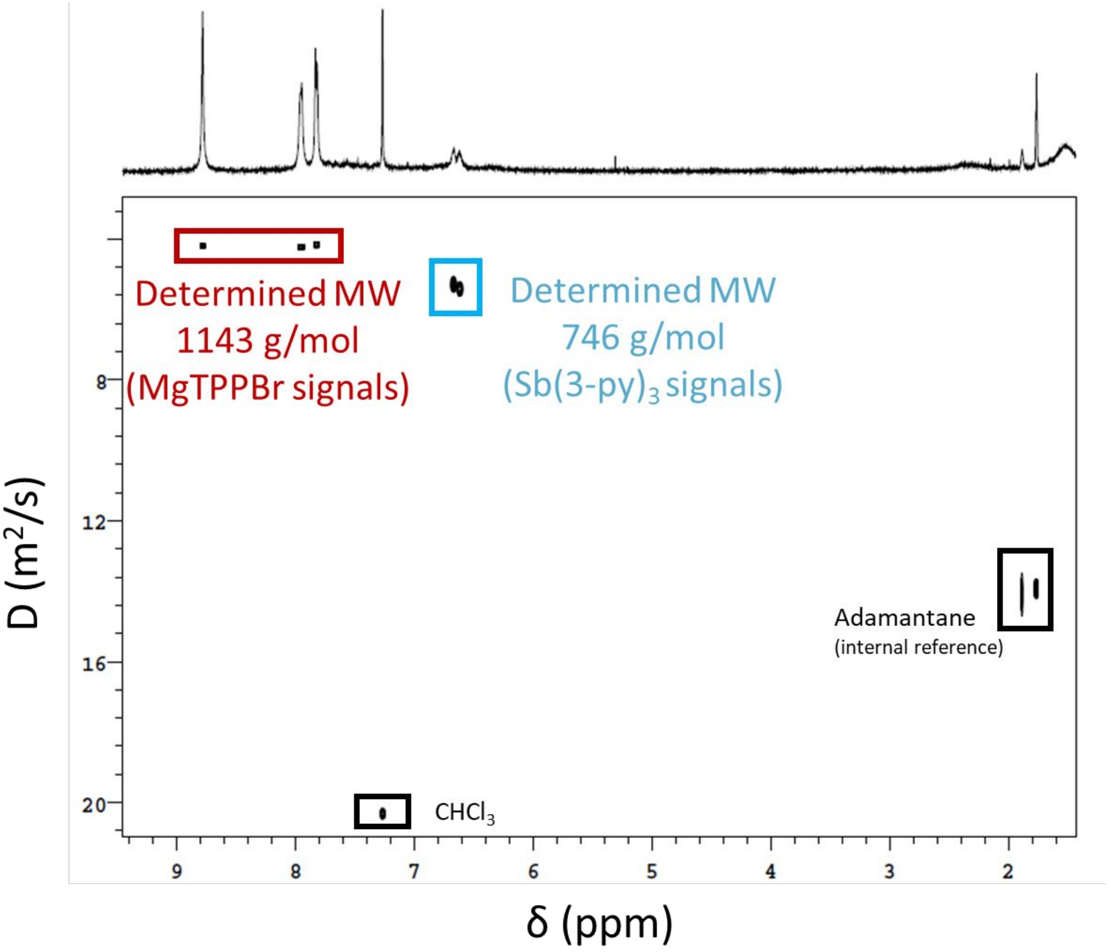

Figure S30. ^{1}H DOSY NMR spectrum of **1**·MgTPPBr and adamantane (internal reference) at 298 K in $CDCl_3$. The diffusion coefficients (*D*) corresponding to the $Sb(3\text{-}py)_3$ and MgTPPBr signals are indicated in blue and red, respectively.

Table S1. D-MW analysis using the ^{1}H DOSY NMR data obtained for the mixture of **1**·MgTPPBr and adamantane (internal reference) at 298 K in $CDCl_3$. [a] Real MW. [b] MW_{det}.

Compound	*D* (m²/s)	log*D*	log$D_{x,norm}$	MW (g/mol)
Adamantane	1.401E-09	-8.8536	---	136.23[a]
MgTPPBr	4.19E-10	-9.3778	-9.3393	1143[b]
$Sb(3\text{-}py)_3$	5.35E-10	-9.2716	-9.2332	746[b]

Variable temperature experiments

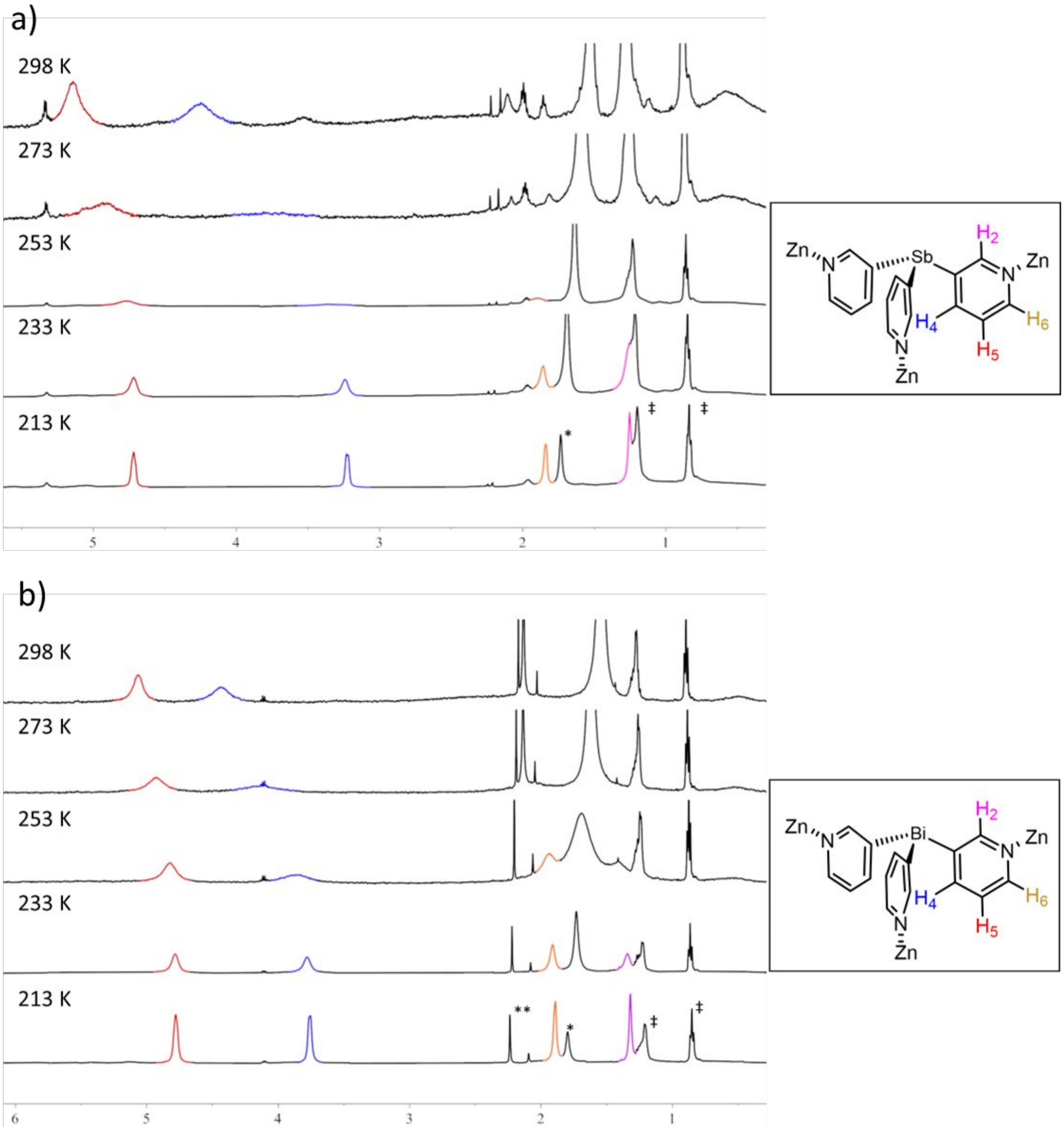

Figure S31. ^{1}H NMR ($CDCl_3$, 500 MHz) spectra of (a) **1**·$ZnTPPF_5$ and (b) **2**·$ZnTPPF_5$ at 298 K, 273 K, 253 K, 233 K and 213 K. As the temperature decreases, the signals corresponding to H_4 (blue) and H_5 (red) become sharper, and signals corresponding to H_6 (brown) and H_2 (purple) appear and become sharp in the spectra. Note: residual H_2O (*) and hexane (‡) were present in **1**·$ZnTPPF_5$ and **2**·$ZnTPPF_5$ (see also Fig. S4), as well as acetone (**) in **2**·$ZnTPPF_5$ (see also Fig. S24 and ^{19}F VT NMR experiments below2424242424).

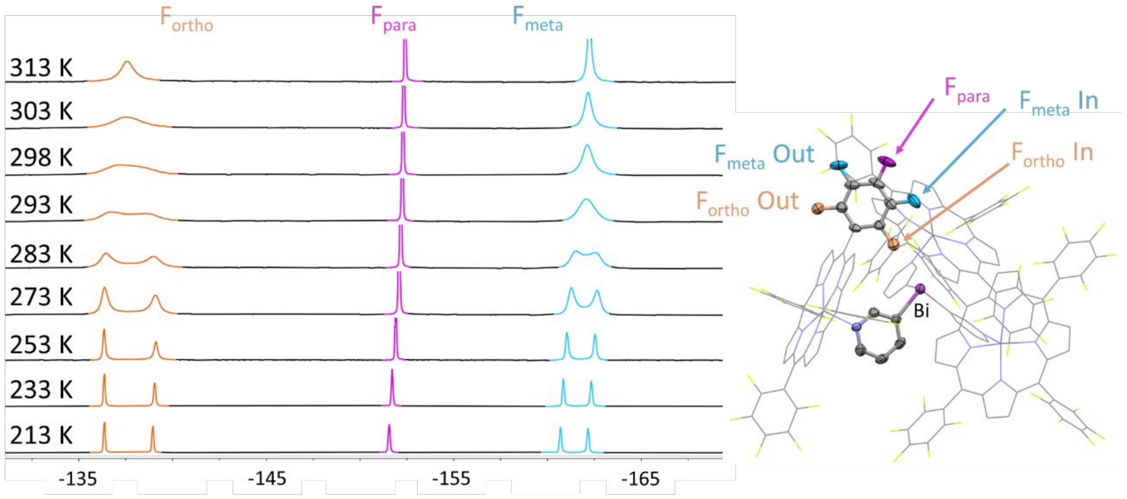

Figure S32. ^{19}F NMR ($CDCl_3$, 470.17 MHz) spectra of **2**·ZnTPPF$_5$ at 313 K, 303 K, 298 K, 293 K, 283 K, 273 K, 253 K, 233 K and 213 K. As the temperature decreases, the signals corresponding to the ortho (brown) and meta (blue) fluorine atoms split into two signals.

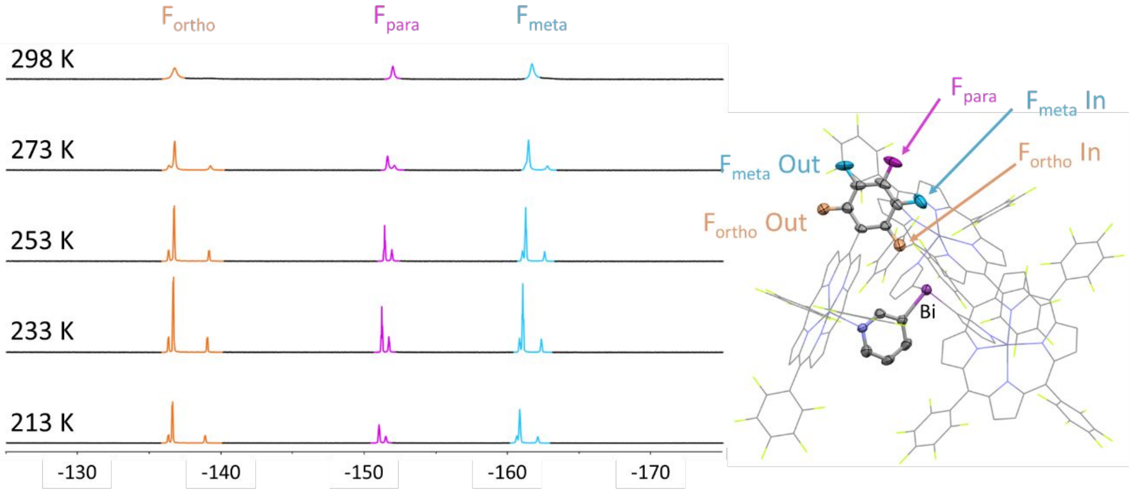

Figure S33. ^{19}F NMR ($CDCl_3$, 470.17 MHz) spectra of **2**·ZnTPPF$_5$ with 6 equivalents of ZnTPPF$_5$ at 298 K, 273 K, 253 K, 233 K and 213 K. As the temperature decreases, the signals corresponding to the ortho and meta fluorine atoms split into three signals, and free ZnTPPF$_5$ is also observed. See also Figure S34.

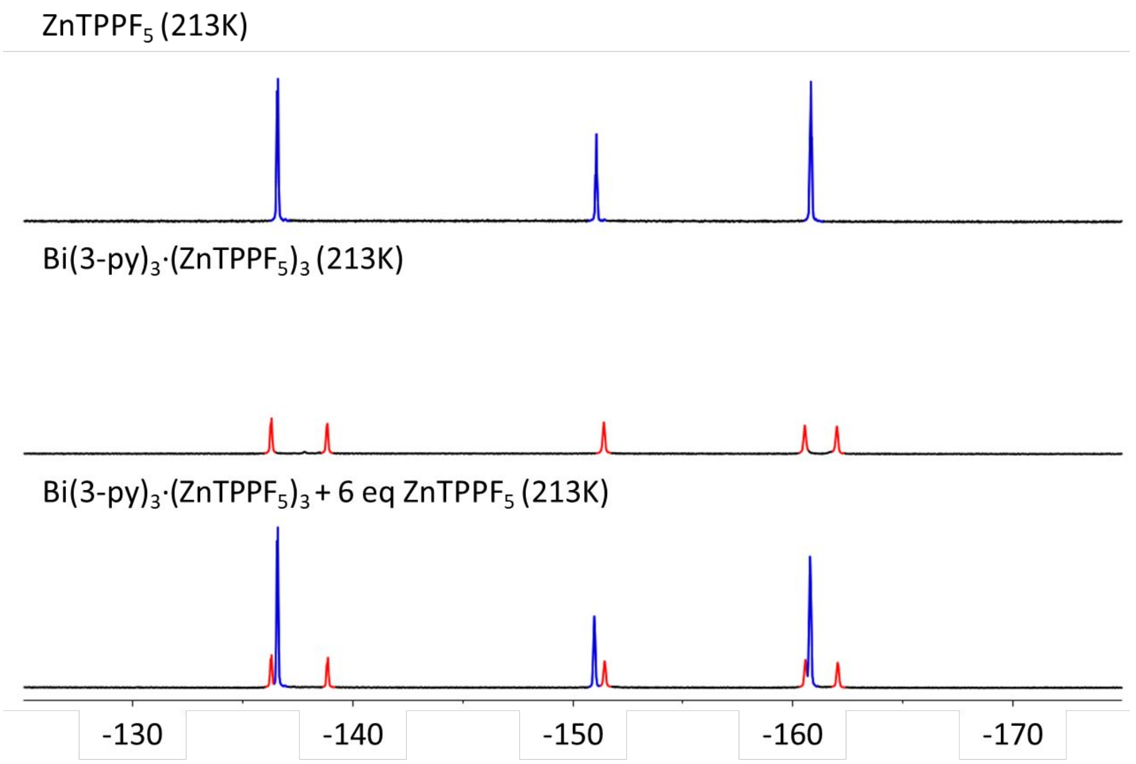

Figure S34. ^{19}F NMR ($CDCl_3$, 470.17 MHz) spectra of $ZnTPPF_5$, **2**·$ZnTPPF_5$ and **2**·$ZnTPPF_5$ + 6 eq $ZnTPPF_5$ at 213 K. The signals corresponding to **2**·$ZnTPPF_5$ and $ZnTPPF_5$ are indicated in red and blue, respectively.

Binding data analysis

Method of continuous variation (Job Plot)[12,13]

The method of continuous variation was used to estimate the stoichiometry of the adducts of ligands **1** (Sb(3-py)$_3$) and **2** (Bi(3-py)$_3$) with the porphyrins **ZnTPPF$_5$**, **ZnTPPOMe** and **ZnTPPBr**. 10 mM solutions of each ligand and porphyrin were prepared in $CDCl_3$. For each compound, the corresponding ligand and porphyrin were combined in 12 different molar fractions (Table S2). The resulting solutions were then transferred to an NMR tube and analyzed using ^{1}H NMR spectroscopy.

Table S2. Molar fractions of ligand and porphyrin in the prepared solutions

	χ_{ligand}	$\chi_{porphyrin}$
1	0.05	0.95
2	0.10	0.90
3	0.15	0.85
4	0.20	0.80
5	0.25	0.75
6	0.30	0.70
7	0.35	0.65
8	0.40	0.60
9	0.45	0.55
10	0.50	0.50
11	0.80	0.20
12	1.00	0.00

The Job plots showed maxima at χ_{ligand} = 0.75 for the adducts of **1** and **2** with the porphyrin ZnTPPF$_5$, confirming the expected 1:3 stoichiometry (Figures S39, S40). On the other hand, the Job plots of **1** and **2** with the porphyrins ZnTPPOMe and ZnTPPBr exhibited maxima at χ_{ligand} = 0.60 (Figures S35, S36) and χ_{ligand} = 0.66–0.70 (Figures S37, S38), respectively, corresponding to an approximately 1:2 stoichiometry. The slight deviations from the theoretical value of χ_{ligand} = 0.66 are unsurprising, as the accuracy of this method is limited in situations of low concentration species and/or low K_1:K_2 ratios.[14,15] Due to the inherent nature of the association constants ratio (see section ^{1}H NMR Titrations for determination of the associate constants), the data could not be improved by increasing the concentrations of the solutions. These findings were further supported by analysis of titration data using non-linear regressions (see section ^{1}H NMR Titrations).

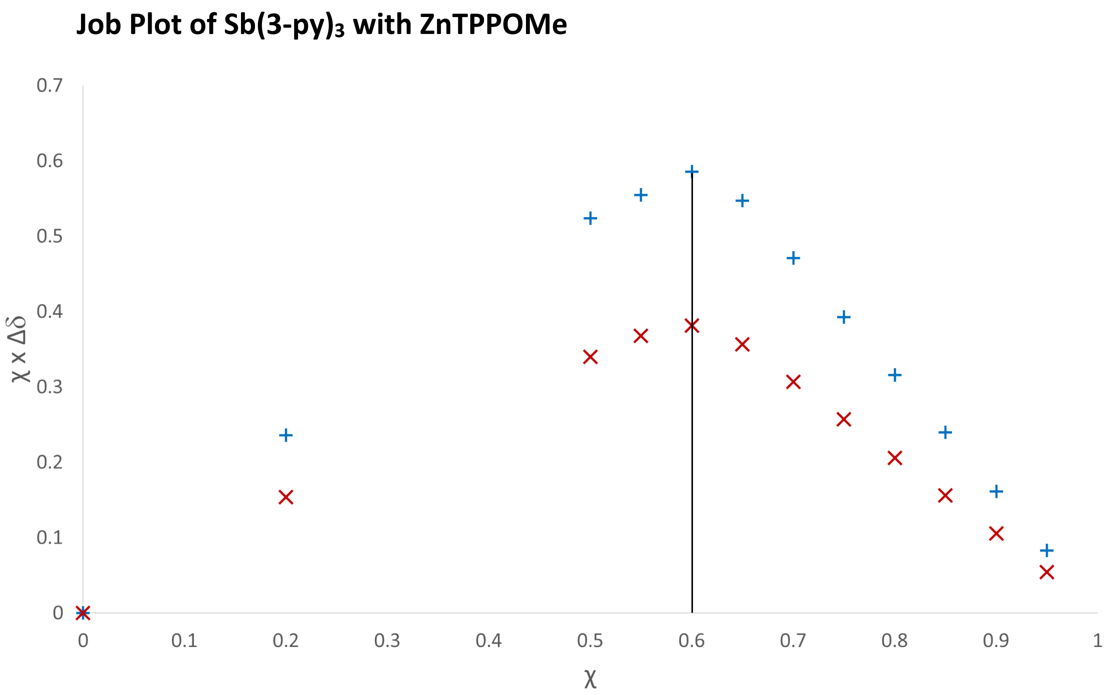

Figure S35. Job plot of $Sb(3\text{-}py)_3$ and ZnTPPOMe; the red “×” symbols correspond to H_5 and the blue “+” symbols to H_4. The maximum at 0.6 indicates a 1:2 stoichiometry, corresponding to **1**·ZnTPPOMe.

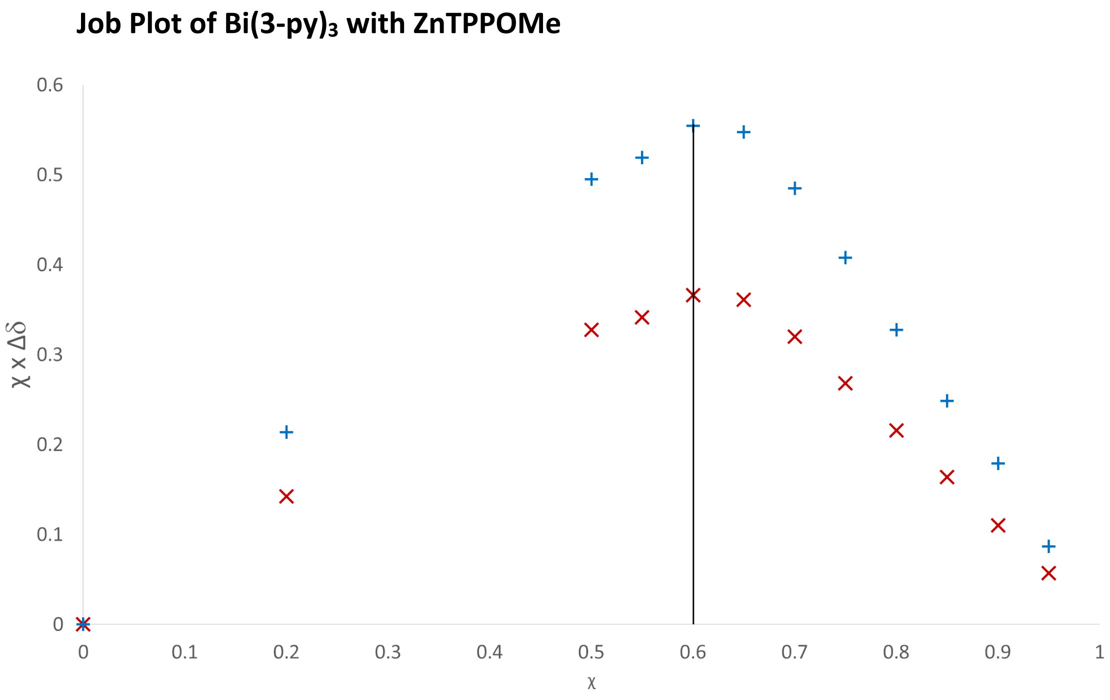

Figure S36. Job plot of $Bi(3\text{-}py)_3$ and ZnTPPOMe; the red “×” symbols correspond to H_5 and the blue “+” symbols to H_4. The maximum at 0.6 indicates a 1:2 stoichiometry, corresponding to **2**·ZnTPPOMe.

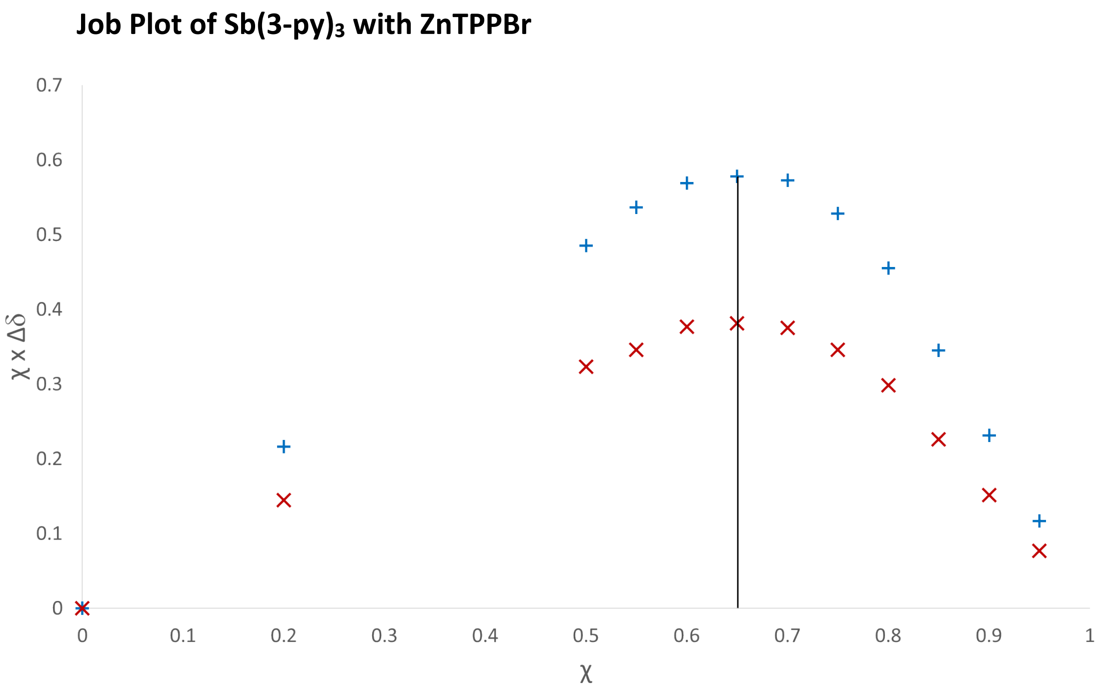

Figure S37. Job plot of Sb(3-py); the red “×” symbols correspond to H_5 and the blue “+” symbols to H_4. The maximum at 0.67 indicates a 1:2 approximately stoichiometry, corresponding to **1**·ZnTPPBr.

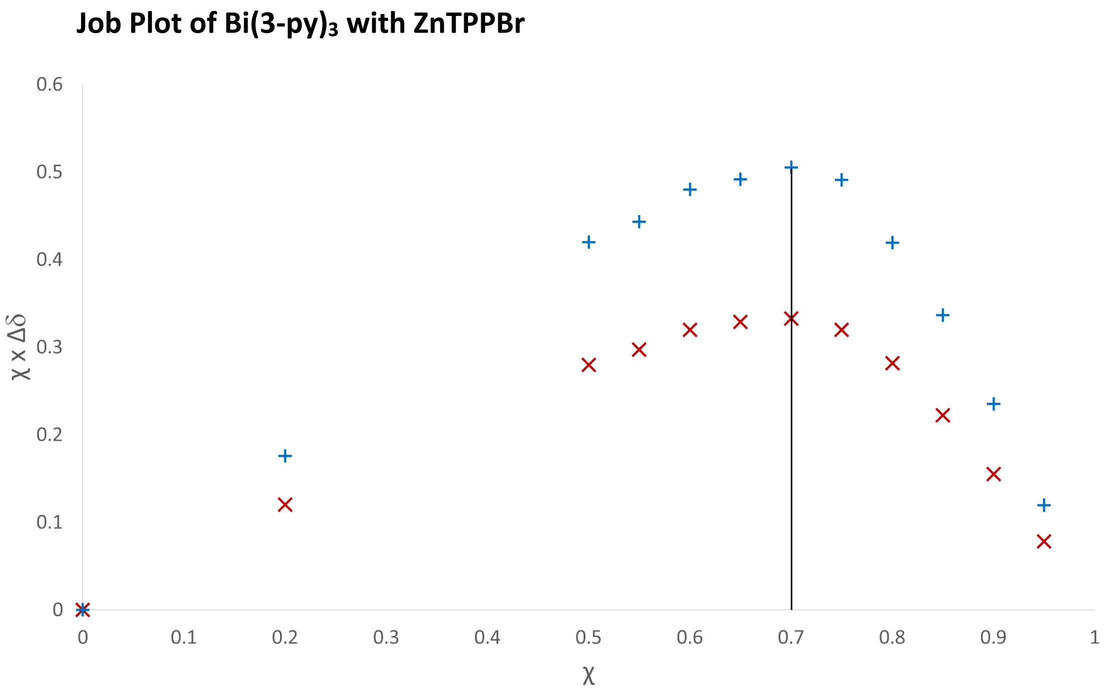

Figure S38. Job plot of $Bi(3\text{-}py)_3$ and ZnTPPBr; the red “×” symbols correspond to H_5 and the blue “+” symbols to H_4. The maximum at 0.7 indicates a 1:2 stoichiometry, corresponding to **2**·ZnTPPBr.

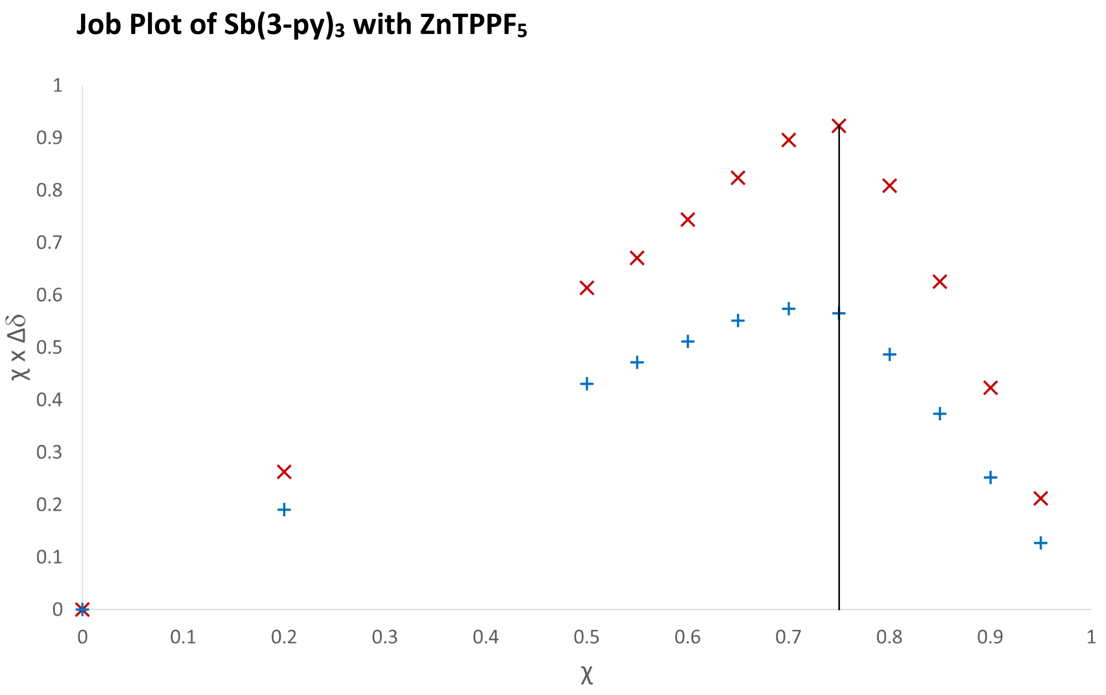

Figure S39. Job plot of Sb(3-py)$_3$ and ZnTPPF$_5$; the red "×" symbols correspond to H$_5$ and the blue "+" symbols to H$_4$. The maxima at 0.75 indicate a 1:3 stoichiometry, corresponding to **1**·ZnTPPF$_5$.

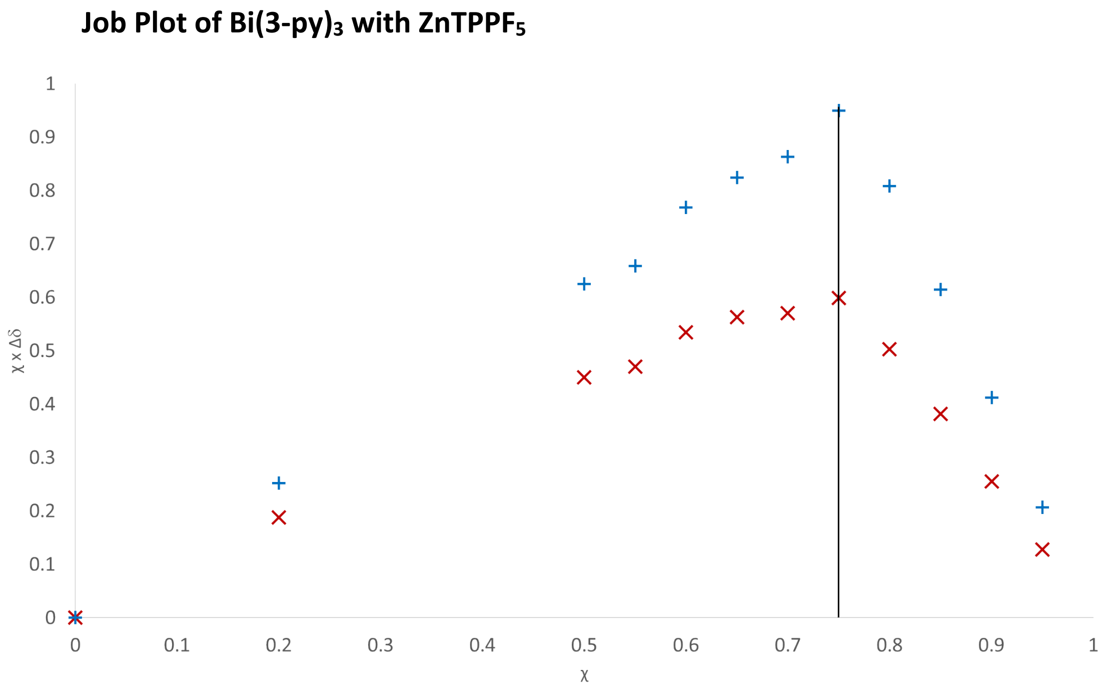

Figure S40. Job plot of Bi(3-py)$_3$ and ZnTPPF$_5$; the red "×" symbols correspond to H$_5$ and the blue "+" symbols to H$_4$. The maximum at 0.75 indicates a 1:3 stoichiometry, corresponding to **2**·ZnTPPF$_5$.

^{1}H NMR Titrations

In order to estimate the association constants (K_a) of the ligands **1** and **2** with the porphyrins **ZnTPPOMe**, **ZnTPPBr** and **ZnTPPF$_5$** ^{1}H NMR titrations were carried out. 10^{-2} M stock solutions of each porphyrin and 10^{-3} M stock solutions of each ligand were prepared in $CDCl_3$. For each titration, 500 µL of the ligand solution was transferred to an NMR tube, and portions of the porphyrin stock solution were added to cover a wide range of molar ratios. After each addition, the ^{1}H NMR spectrum was recorded at 298 K. The chemical shifts of selected protons were then plotted as a function of the molar fraction of the guest (porphyrin), and the resulting curve was fitted by a nonlinear method using the global analysis approach described below.

Fitting to 1:1 and 1:2 models

The NMR titration data was fitted to 1:1 and 1:2 binding models using Bindfit (supramolecular.org). The equations for these models have been detailed previously[16,17] and will not be repeated here. Links to all the fittings are provided below.

***1** vs ZnTPPOMe:*

- 1:1 model:
http://app.supramolecular.org/bindfit/view/90ed40a9-3c9d-4c52-a9aa-037aeb28b102

- 1:2 full model:
http://app.supramolecular.org/bindfit/view/d42493b2-24ea-4c48-9119-5232a2ae8946

- 1:2 additive model:
http://app.supramolecular.org/bindfit/view/05afe997-419f-45b5-8de6-482c51682b57

- 1:2 non-cooperative model:
http://app.supramolecular.org/bindfit/view/11682d5f-e883-49cc-91b8-b581c0e96cb4

- 1:2 statistic model:
http://app.supramolecular.org/bindfit/view/9e12bb43-b4fd-4ef4-9437-95511c1744b2

***2** vs ZnTPPOMe:*

- 1:1 model:
http://app.supramolecular.org/bindfit/view/2a915c4e-fba4-433c-8545-8ee2ce3715d9

- 1:2 full model:
http://app.supramolecular.org/bindfit/view/b7de2a41-16c0-40e6-a757-b670eb6b29b3

- 1:2 additive model:
http://app.supramolecular.org/bindfit/view/7166e7c1-96e0-42be-9792-fb8decee81ff

- 1:2 non-cooperative model:
http://app.supramolecular.org/bindfit/view/c0727eab-df0c-4f2f-99ff-c90b91dc430f

- 1:2 statistic model:
http://app.supramolecular.org/bindfit/view/916a5561-7b48-4ecd-814b-0769121ac218

1 *vs ZnTPPBr:*

- 1:1 model:
http://app.supramolecular.org/bindfit/view/8356bdca-b08a-4609-9425-01829715c13d

- 1:2 full model:
http://app.supramolecular.org/bindfit/view/4d6b719e-30e6-41f9-9d20-46c1fcaf594f

- 1:2 additive model:
http://app.supramolecular.org/bindfit/view/7f24585b-fc2a-4b4d-849d-c2ea2fa611be

- 1:2 non-cooperative model:
http://app.supramolecular.org/bindfit/view/29865cd0-92d4-4eb7-9957-97f49393e5ae

- 1:2 statistic model:
http://app.supramolecular.org/bindfit/view/9ae0f85d-1244-4b16-a9d8-1626dfd2ec5b

2 *vs ZnTPPBr:*

- 1:1 model:
http://app.supramolecular.org/bindfit/view/2d5078a5-68bd-4811-a96b-294c36b05b3e

- 1:2 full model:
http://app.supramolecular.org/bindfit/view/2982cce5-6f8a-480b-b1ec-4c0946a6b6c4

- 1:2 additive model:
http://app.supramolecular.org/bindfit/view/951eeb0c-43fd-4bc4-bb90-2aeea92ce342

- 1:2 non-cooperative model:
http://app.supramolecular.org/bindfit/view/08c1d0ba-dcb8-4282-9184-5b64cda96e03

- 1:2 statistic model:
http://app.supramolecular.org/bindfit/view/78e35050-77da-4f03-b3cc-3e015cd37dd2

1 *vs* $ZnTPPF_5$:

- 1:1 model:
http://app.supramolecular.org/bindfit/view/f315e4e2-47a5-4bff-81ce-02b24bd4be72

- 1:2 full model:
http://app.supramolecular.org/bindfit/view/b3972e59-97a4-4c71-9c3a-2083c1555786

- 1:2 additive model:
http://app.supramolecular.org/bindfit/view/32885474-b99f-42f1-828c-8d035fd8ac04

- 1:2 non-cooperative model:
http://app.supramolecular.org/bindfit/view/433cf54f-108e-447f-bb10-fc4f923eba04

- 1:2 statistic model:
http://app.supramolecular.org/bindfit/view/dc5515a3-dc48-4f64-b945-fedcfd44233c

***2** vs ZnTPPF$_5$:*

- 1:1 model:
http://app.supramolecular.org/bindfit/view/de900322-487c-4232-9cac-04f5c765c8db

- 1:2 full model:
http://app.supramolecular.org/bindfit/view/76912cc2-434a-4155-8d70-33428b22bde6

- 1:2 additive model:
http://app.supramolecular.org/bindfit/view/3c39146b-de17-4c3d-abad-bc0c6ee7d60d

- 1:2 non-cooperative model:
http://app.supramolecular.org/bindfit/view/c30da630-91e9-4bb1-83ca-c1eb157dcdad

- 1:2 statistic model:
http://app.supramolecular.org/bindfit/view/4edecf56-51ab-479e-8a4d-231961ca7569

Fitting to 1:3 models

The data was fitted to a 1:3 model using the approach previously described by Bownam-James, Thordarson, and co-workers in their ^{1}H NMR study of the 1:3 association of a phosphate macrocycle adduct.[18,19] The association equilibrium constants and mass balance equations are as follows:

$$K_1 = \frac{[\mathrm{HG}]}{[\mathrm{H}]\,[\mathrm{G}]} \quad \mathrm{eq\ 1;} \quad K_2 = \frac{[\mathrm{HG_2}]}{[\mathrm{HG}]\,[\mathrm{G}]} \quad \mathrm{eq\ 2;} \quad K_1 = \frac{[\mathrm{HG_3}]}{[\mathrm{HG_2}]\,[\mathrm{G}]} \quad \mathrm{eq\ 3}$$

$$[\mathrm{H}]_0 = [\mathrm{H}] + [\mathrm{HG}] + [\mathrm{HG_2}] + [\mathrm{HG_3}] \quad \mathrm{eq\ 4}$$

$$[\mathrm{G}]_0 = [\mathrm{G}] + [\mathrm{HG}] + 2[\mathrm{HG_2}] + 3[\mathrm{HG_3}] \quad \mathrm{eq\ 5}$$

The data was fitted to this model using the Matlab script provided by Bownam-James, Thordarson, and co-workers.[18]

For the 1:2 and 1:3 stoichiometry models, four 'flavors' were considered. Each of these flavors is described below using the 1:3 stoichiometry as an example.

- Stepwise (non-degenerate) full 1:3 binding model. In this model, no assumptions are made and all the parameters (K_1, K_2, K_3 $\Delta\delta_{\Delta HG}$, $\Delta\delta_{\Delta HG2}$, $\Delta\delta_{\Delta HG3}$) are evaluated independently.

- Stepwise (non-degenerate), additive 1:3 binding model. In this case, it is assumed that $\Delta\delta_{\Delta HG}$ = 1/2 $\Delta\delta_{\Delta HG2}$ = 1/3 $\Delta\delta_{\Delta HG3}$. Therefore, the analysis is simplified, and only four parameters are fitted (K_1, K_2, K_3, $\Delta\delta_{\Delta HG}$).

- Stepwise non-cooperative 1:3 binding model. In this case, it is assumed that $K_1 = 3K_2 = 9K_3$. Defining K_{1n} = as $K_{1n} = K_1 = 3K_2 = 9K_3$, this model also furnishes four parameters to be fitted ($\Delta\delta_{\Delta HG}$, $\Delta\delta_{\Delta HG2}$, $\Delta\delta_{\Delta HG3}$, K_{1n}).

- Statistical 1:3 model. In this case, both assumptions are made, *i.e.*, $K_1 = 3K_2 = 9K_3$ and $\Delta\delta_{\Delta HG} = 1/2\ \Delta\delta_{\Delta HG2} = 1/3\ \Delta\delta_{\Delta HG3}$. This provides the simplest fit, as only two parameters are evaluated ($\Delta\delta_{\Delta HG}$, K_{1n}).

All binding isotherms were systematically fit to all models and flavors, and the results are presented in Tables S7–S6.

Table S3. Association constants (M^{-1}) of host Sb(3-py)$_3$ with guest ZnTPPOMe obtained using the different binding models

Model	K_1	K_2	K_3	SSR[a]	SE_y[b]	cov_{fit} ratio[c]
**1-to-1**	1.44E+03	---	---	0.20	0.06	1.00
**1-to-2 full**	3.66E+03	3.01E+03	---	0.01	0.02	14.87
**1-to-2 additive**	7.63E+02	-2.00E+01	---	0.14	0.05	1.48
**1-to-2 non-coop**	7.29E+02	1.82E+02	---	0.11	0.05	1.81
**1-to-2 statistical**	9.19E+03	2.30E+03	---	0.02	0.02	11.54
**1-to-3 full**	1.26E+01	6.42E+05	1.72E+01	0.00	0.01	72.04
**1-to-3 additive**	7.66E+03	3.56E+03	9.39E+00	0.01	0.02	15.93
**1-to-3 non-coop**	1.66E+04	5.54E+03	1.85E+03	0.02	0.02	11.87
**1-to-3 statistical**	1.23E+05	4.10E+04	1.37E+04	0.35	0.08	0.63

Table S4. Association constants (M^{-1}) of host Bi(3-py)$_3$ with guest ZnTPPOMe obtained using the different binding models

Model	K_1	K_2	K_3	SSR[a]	SE_y[b]	cov_{fit} ratio[c]
**1-to-1**	1.70E+03	---	---	0.16	0.06	1.00
**1-to-2 full**	6.52E+03	2.42E+03	---	0.02	0.02	6.61
**1-to-2 additive**	1.09E+03	-1.46E+01	---	0.13	0.05	1.28
**1-to-2 non-coop**	9.39E+03	2.35E+03	---	0.02	0.02	6.51
**1-to-2 statistical**	1.17E+04	2.93E+03	---	0.03	0.03	5.14
**1-to-3 full**	1.12E-09	8.74E+15	3.31E+00	0.00	0.01	50.12
**1-to-3 additive**	2.87E+04	3.45E+03	1.40E+01	0.02	0.02	7.14
**1-to-3 non-coop**	1.38E+04	4.59E+03	1.53E+03	0.03	0.03	4.99
**1-to-3 statistical**	1.42E+05	4.74E+04	1.58E+04	0.65	0.12	0.28

Table S5. Association constants (M^{-1}) of host Sb(3-py)$_3$ with guest ZnTPPBr obtained using the different binding models

Model	K_1	K_2	K_3	SSR[a]	SE_y[b]	cov_{fit} ratio[c]
**1-to-1**	1.00E+03	---	---	0.30	0.08	1.00
**1-to-2 full**	7.22E+03	1.91E+03	---	0.02	0.02	19.81
**1-to-2 additive**	4.68E+02	-2.33E+01	---	0.20	0.06	1.51
**1-to-2 non-coop**	4.34E+02	1.08E+02	---	0.17	0.06	1.72

**1-to-2 statistical**	4.98E+03	1.25E+03	---	0.06	0.04	4.70
**1-to-3 full**	4.99E+03	2.86E+03	2.58E-07	0.02	0.02	15.49
**1-to-3 additive**	2.46E+03	4.22E+03	2.28E+01	0.02	0.02	14.97
**1-to-3 non-coop**	9.04E+03	3.01E+03	1.00E+03	0.03	0.03	8.83
**1-to-3 statistical**	3.53E+04	1.18E+04	3.92E+03	0.15	0.06	2.05

Table S6. Association constants (M^{-1}) of host Bi(3-py)$_3$ with guest ZnTPPBr obtained using the different binding models

Model	K_1	K_2	K_3	SSR[a]	SE_y[b]	cov_{fit} ratio[c]
**1-to-1**	1.13E+03	---	---	0.26	0.07	1.00
**1-to-2 full**	2.73E+03	2.62E+03	---	0.01	0.02	16.49
**1-to-2 additive**	5.24E+02	-2.33E+01	---	0.16	0.06	1.56
**1-to-2 non-coop**	4.79E+02	1.20E+02	---	0.13	0.05	1.85
**1-to-2 statistical**	6.02E+03	1.51E+03	---	0.04	0.03	6.63
**1-to-3 full**	3.00E+02	3.92E+04	3.05E+02	0.01	0.01	34.68
**1-to-3 additive**	3.31E+03	3.13E+03	8.03E+00	0.01	0.02	18.33
**1-to-3 non-coop**	1.18E+04	3.93E+03	1.31E+03	0.02	0.02	14.98
**1-to-3 statistical**	5.69E+04	1.90E+04	6.33E+03	0.21	0.07	1.29

Table S7. Association constants (M^{-1}) of host Sb(3-py)$_3$ with guest ZnTPPF$_5$ obtained using the different binding models

Model	K_1	K_2	K_3	SSR[a]	SE_y[b]	cov_{fit} ratio[c]
**1-to-1**	6.04E+02	---	---	2.34	0.22	1.00
**1-to-2 full**	7.24E+00	2.46E+05	---	0.06	0.04	40.48
**1-to-2 additive**	1.31E+02	-3.35E+01	---	1.24	0.16	1.88
**1-to-2 non-coop**	6.80E+03	1.70E+03	---	0.20	0.06	11.73
**1-to-2 statistical**	2.35E+03	5.89E+02	---	1.35	0.17	1.75
**1-to-3 full**	2.97E+04	1.40E+04	2.28E+03	0.00	0.01	1854.31
**1-to-3 additive**	1.17E+02	1.78E+04	6.81E-08	0.21	0.07	11.00
**1-to-3 non-coop**	2.21E+04	7.37E+03	2.46E+03	0.00	0.01	1059.54
**1-to-3 statistical**	1.11E+04	3.71E+03	1.24E+03	0.34	0.08	7.00

Table S8. Association constants (M^{-1}) of host Bi(3-py)$_3$ with guest ZnTPPF$_5$ obtained using the different binding models

Model	K_1	K_2	K_3	SSR[a]	SE_y[b]	cov_{fit} ratio[c]
**1-to-1**	1.00E+03	---	---	3.59	0.27	1.00
**1-to-2 full**	1.13E+01	8.39E+05	---	0.07	0.04	51.96
**1-to-2 additive**	2.89E+02	-3.23E+01	---	2.23	0.22	1.61
**1-to-2 non-coop**	2.43E+02	6.08E+01	---	2.00	0.21	1.81
**1-to-2 statistical**	5.45E+03	1.36E+03	---	1.63	0.18	2.24
**1-to-3 full**	1.90E+05	2.17E+05	5.64E+03	0.01	0.02	338.18
**1-to-3 additive**	9.87E-33	5.83E+38	2.73E-08	0.23	0.07	15.50
**1-to-3 non-coop**	5.05E+04	1.68E+04	5.61E+03	0.02	0.02	210.29
**1-to-3 statistical**	9.11E+04	3.04E+04	1.01E+04	0.15	0.06	22.84

[a] SSR is the squared sum of residuals:

$$SSR = \sum (y_{\mathrm{data}} - y_{\mathrm{calc}})^2 \quad \text{eq 10}$$

[b] SE_y is the error in the estimation of *y*:

$$\mathrm{SE}_y = \sqrt{\frac{\sum (y_{\mathrm{data}} - y_{\mathrm{calc}})^2}{N-k}} \quad \text{eq 11}$$

[c] The cov_{fit} ratio was calculated as:

$$\mathrm{cov_{fit}\ ratio} = \frac{\mathrm{cov_{fit}(simplest\ model)}}{\mathrm{cov_{fit}(complex\ model)}} \quad \text{eq 12}$$

The most significant model was chosen based on two important factors, namely, chemically meaningful values and statistical parameters.

Regarding the first factor, models with meaningless negative *K* values or remarkably aberrant trends (large variation between consecutive association constants) were ruled out. An example of the former is entry 3 in Table S7 (1-to-2 additive), which has a value of -33.5 for K_2. An example of the latter is entry 7 in Table S8 (1-to-3 additive), which has an extraordinarily low K_1 value (9.87×10^{-33}) and an absurdly high K_2 value (5.83×10^{38}). This indicates the inherent instability of the model and is usually accompanied by poorer statistical descriptors.

Regarding the second factor, the model was chosen based on the lowest fitting errors (SSR and SE_y) as well as the highest covariance fit ratios (*i.e.*, the ratio between the covariance of fit for a complex model with respect to a model of reference, which is usually the simplest 1:1 stoichiometry model). Typically, a ratio of 3 to 5 or greater justifies the selection of the complex model/flavor over the simpler one, as this indicates that the complex model describes the data significantly better.[14]

In light of the above rationale and the results shown in Tables S7–S6, the models with 1:3 stoichiometry and the full flavor were chosen for the guest $ZnTPPF_5$ with both hosts $E(3\text{-py})_3$. On the other hand, for the guests ZnTPPOMe and ZnTPPBr, the best model with both hosts $E(3\text{-py})_3$ proved to be the full 1:2 association one (Table S9).

Table S9. Binding constants for the chosen binding models for each ligand and porphyrin combination

**Ligand/Porphyrin**	**ZnTPPOMe**	**ZnTPPBr**	**ZnTPPF$_5$**
**Sb(3-py)$_3$**	$K_1 = (3.7 \pm 0.5) \times 10^3$	$K_1 = (7.2 \pm 1.8) \times 10^3$	$K_1 = (2.97 \pm 1.4) \times 10^4$
	$K_2 = (3.0 \pm 0.3) \times 10^3$	$K_2 = (1.9 \pm 0.1) \times 10^3$	$K_2 = (1.40 \pm 0.9) \times 10^4$
	---	---	$K_3 = (2.28 \pm 0.3) \times 10^3$
**Bi(3-py)$_3$**	$K_1 = (6.5 \pm 1.1) \times 10^3$	$K_1 = (2.7 \pm 0.4) \times 10^3$	$K_1 = (1.90 \pm 1.0) \times 10^5$
	$K_2 = (2.4 \pm 0.3) \times 10^3$	$K_2 = (2.6 \pm 0.3) \times 10^3$	$K_2 = (2.17 \pm 0.3) \times 10^5$
	---	---	$K_3 = (5.64 \pm 0.7) \times 10^3$

Titration study of $Sb(3\text{-}py)_3$ with ZnTPPOMe

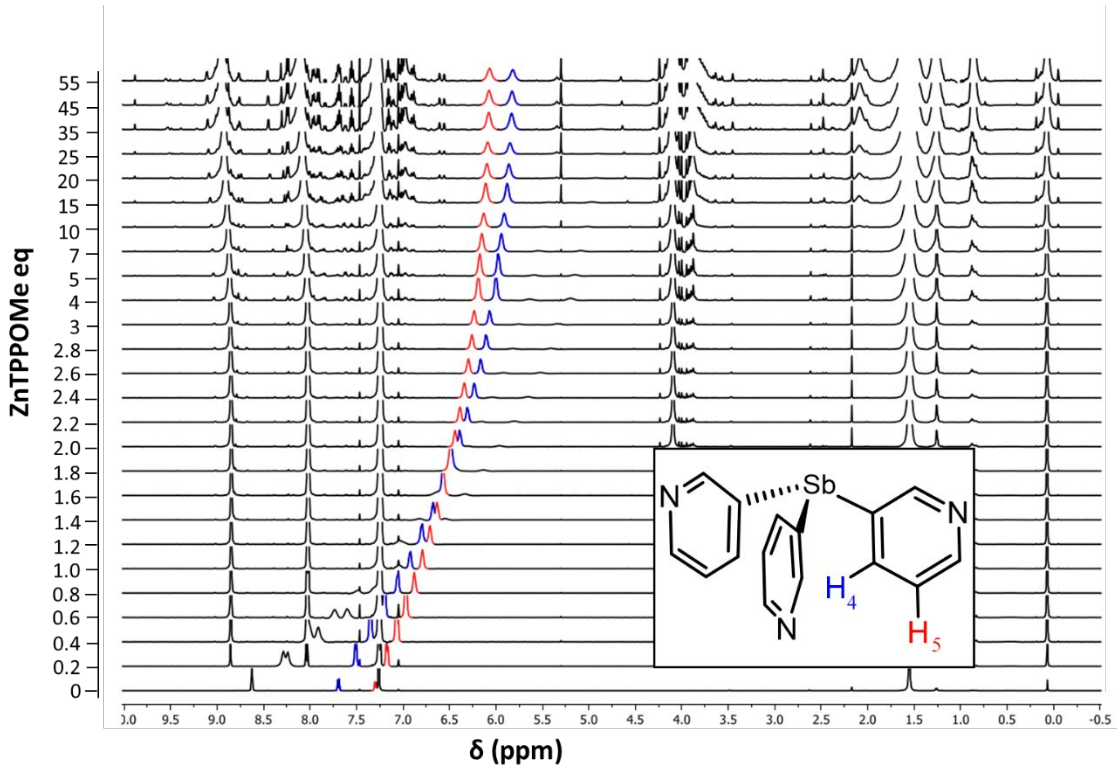

Figure S41. Stacked ^{1}H NMR spectra of the titration of ligand $Sb(3\text{-}py)_3$ with up to 55 equivalents of ZnTPPOMe. The signals of the ligands that were followed are indicated in blue (H_4) and red (H_5), respectively.

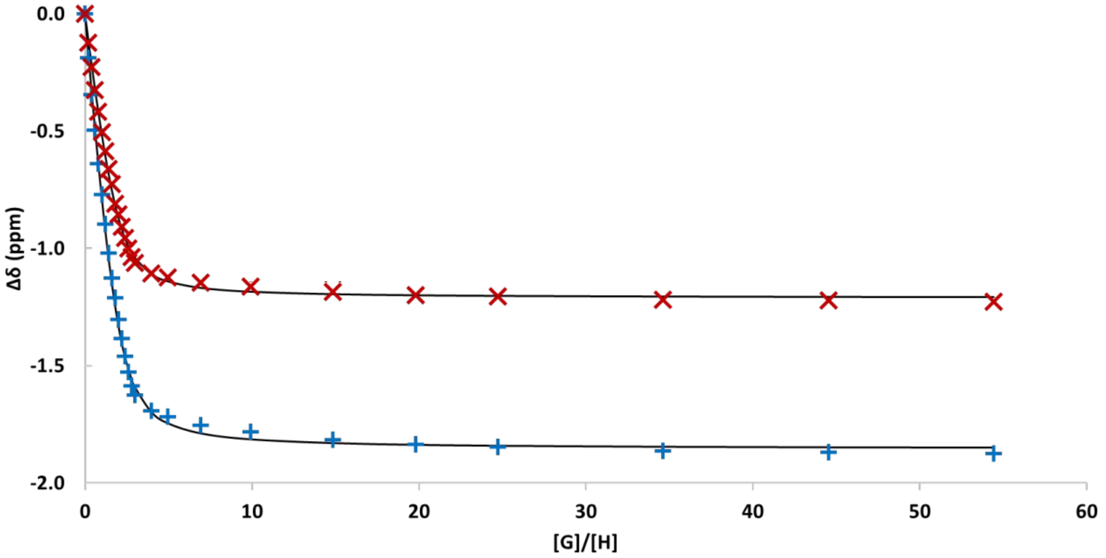

Figure S42. Changes in the chemical shifts of the $Sb(3\text{-}py)_3$ signals (H_4 in blue and H_5 in red) vs [G]/[H], and the theoretical values obtained from the non-linear regression to a 1:2 binding isotherm (black lines).

Titration study of Bi(3-py)$_3$ with ZnTPPOMe

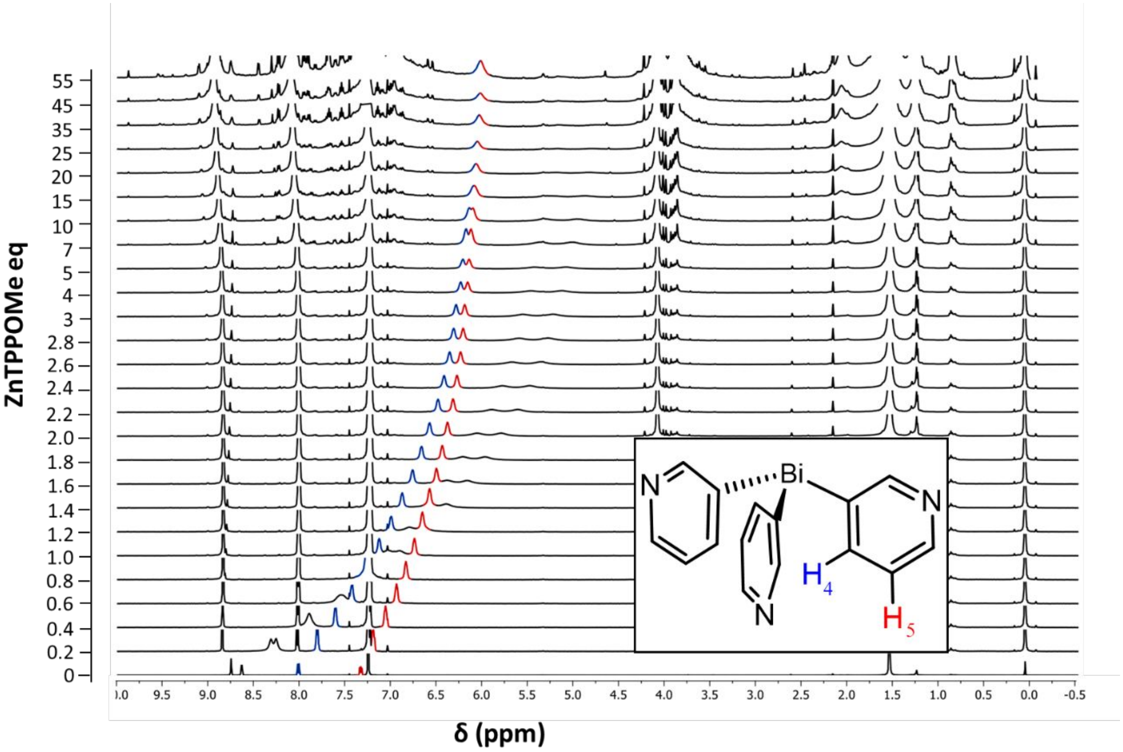

Figure S43. Stacked ^{1}H NMR spectra of the titration of ligand Bi(3-py)$_3$ with up to 55 equivalents of ZnTPPOMe. The signals of the ligands that were followed are indicated in blue (H_4) and red (H_5), respectively.

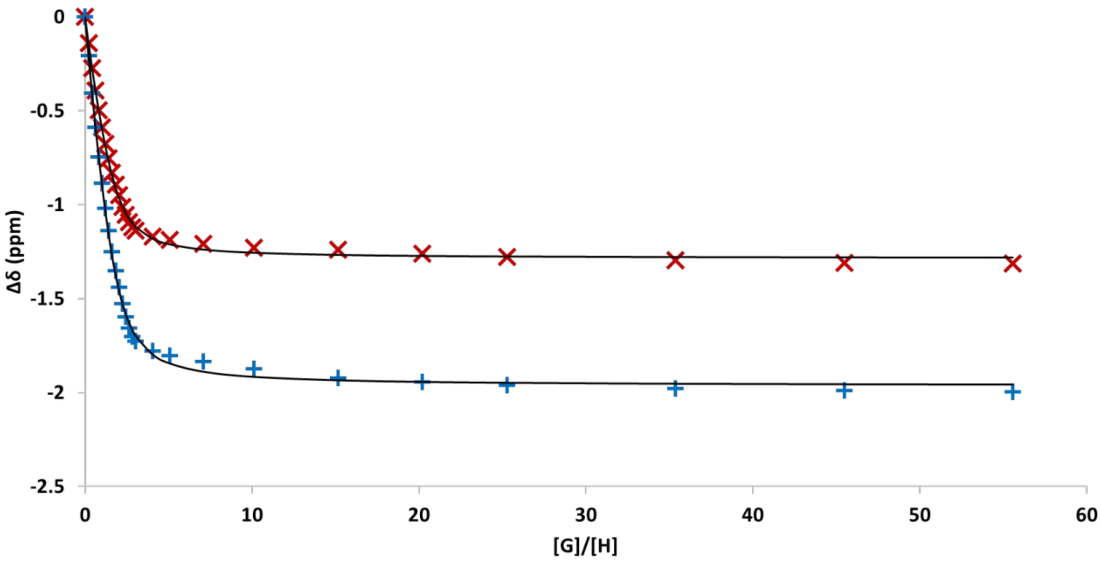

Figure S44. Changes in the chemical shifts of the Bi(3-py)$_3$ signals (H_4 in blue and H_5 in red) versus [G]/[H], and the theoretical values obtained from the non-linear regression to a 1:2 binding isotherm (black lines).

Titration study of Sb(3-py)$_3$ with ZnTPPBr

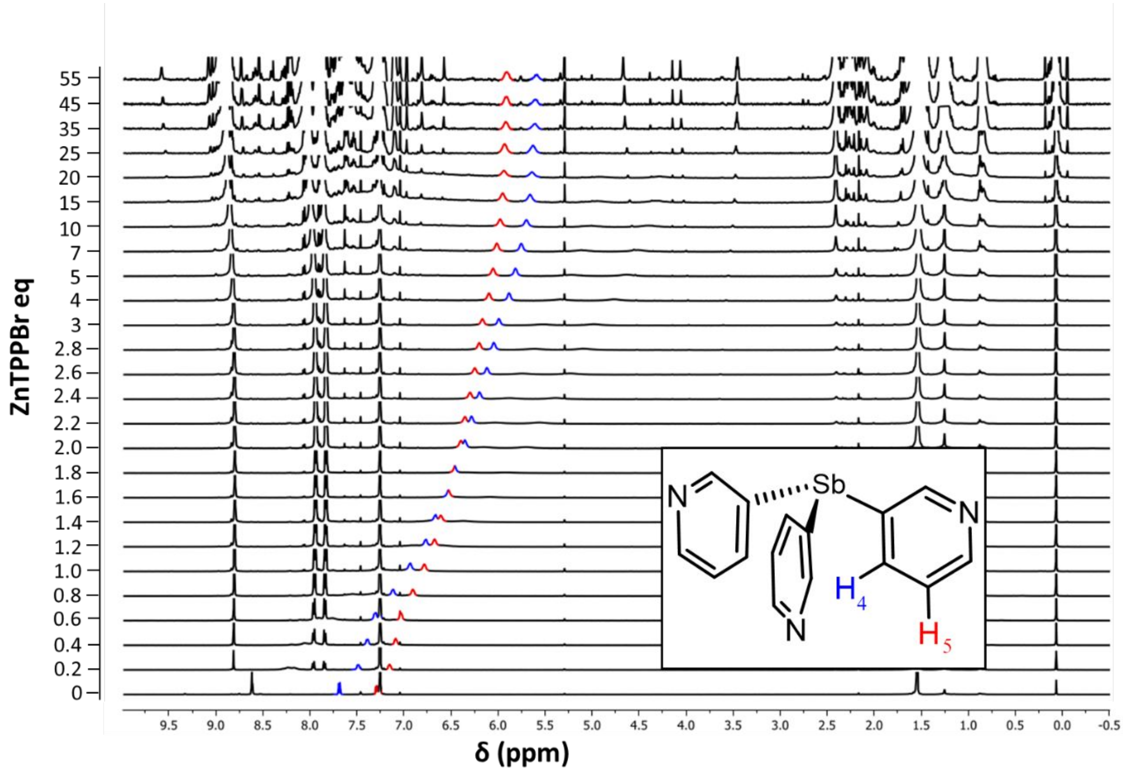

Figure S45. Stacked ^{1}H NMR spectra of the titration of ligand Sb(3-py)$_3$ with up to 55 equivalents of ZnTPPBr. The signals of the ligands that were followed are indicated in blue (H_4) and red (H_5), respectively.

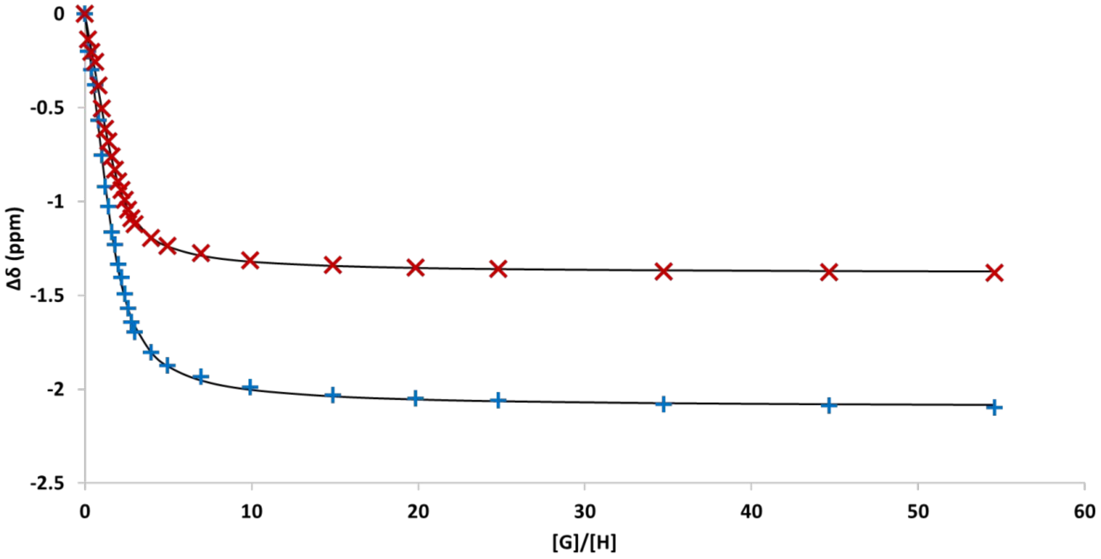

Figure S46. Changes in the chemical shifts of the Sb(3-py)$_3$ signals (H_4 in blue and H_5 in red) versus [G]/[H], and the theoretical values obtained from the non-linear regression to a 1:2 binding isotherm (black lines).

Titration study of Bi(3-py)$_3$ with ZnTPPBr

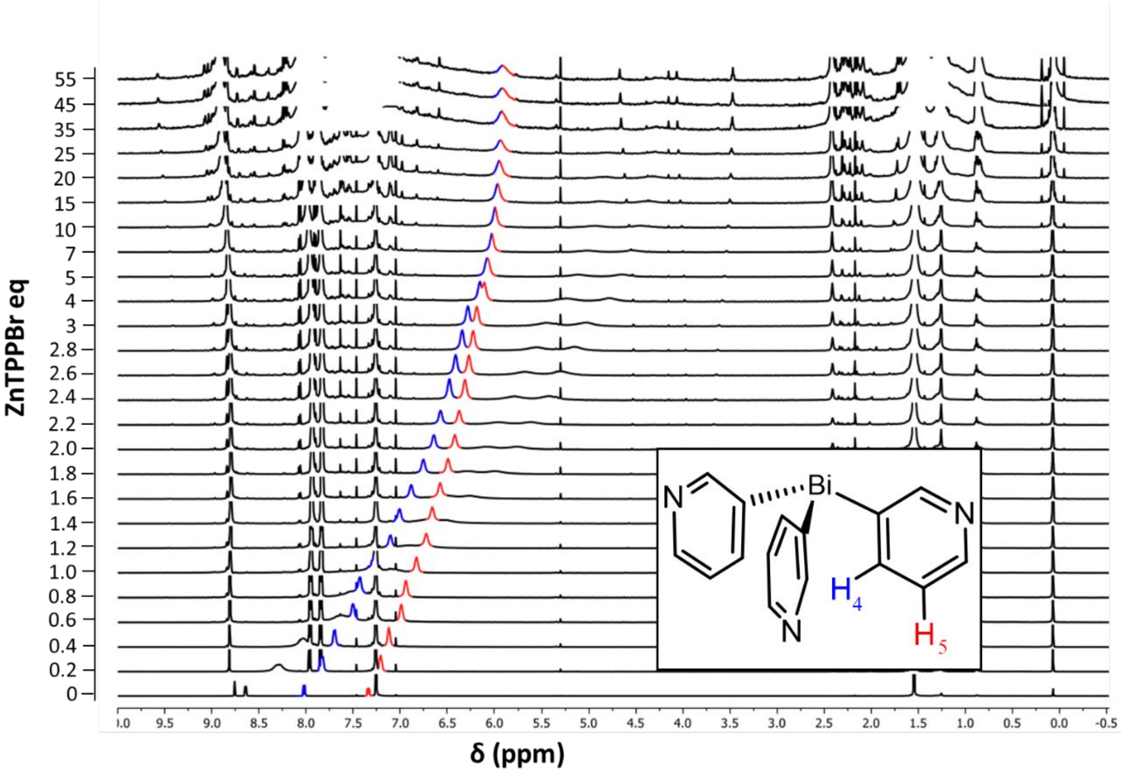

Figure S47. Stacked ^{1}H NMR spectra of the titration of ligand Bi(3-py)$_3$ with up to 55 equivalents of ZnTPPBr. The signals of the ligands that were followed are indicated in blue (H_4) and red (H_5), respectively.

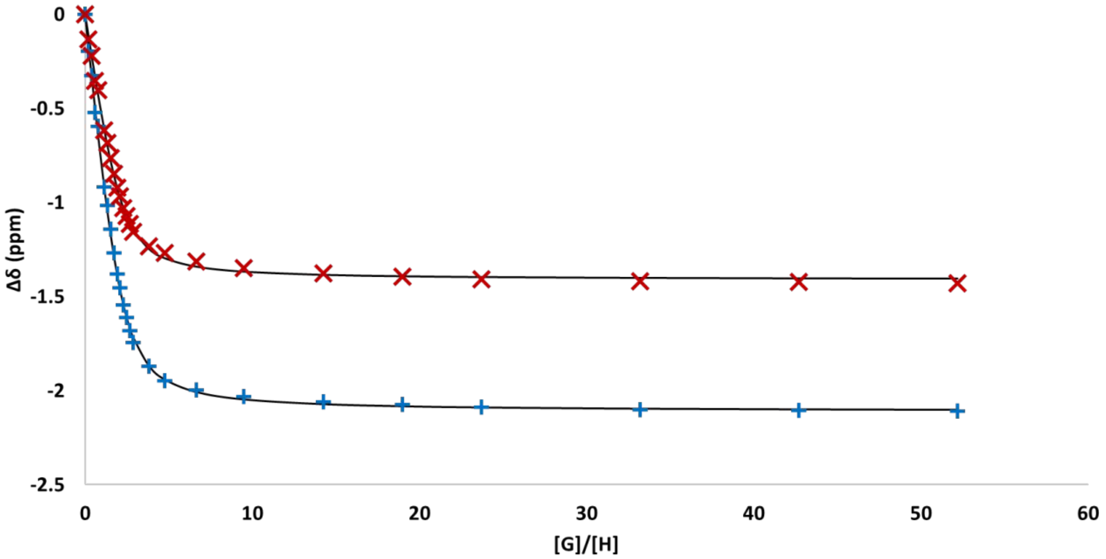

Figure S48. Changes in the chemical shifts of the Bi(3-py)$_3$ signals (H_4 in blue and H_5 in red) against [G]/[H], and the theoretical values obtained from the non-linear regression to a 1:2 binding isotherm (black lines).

Titration study of pyridine with $ZnTPPF_5$

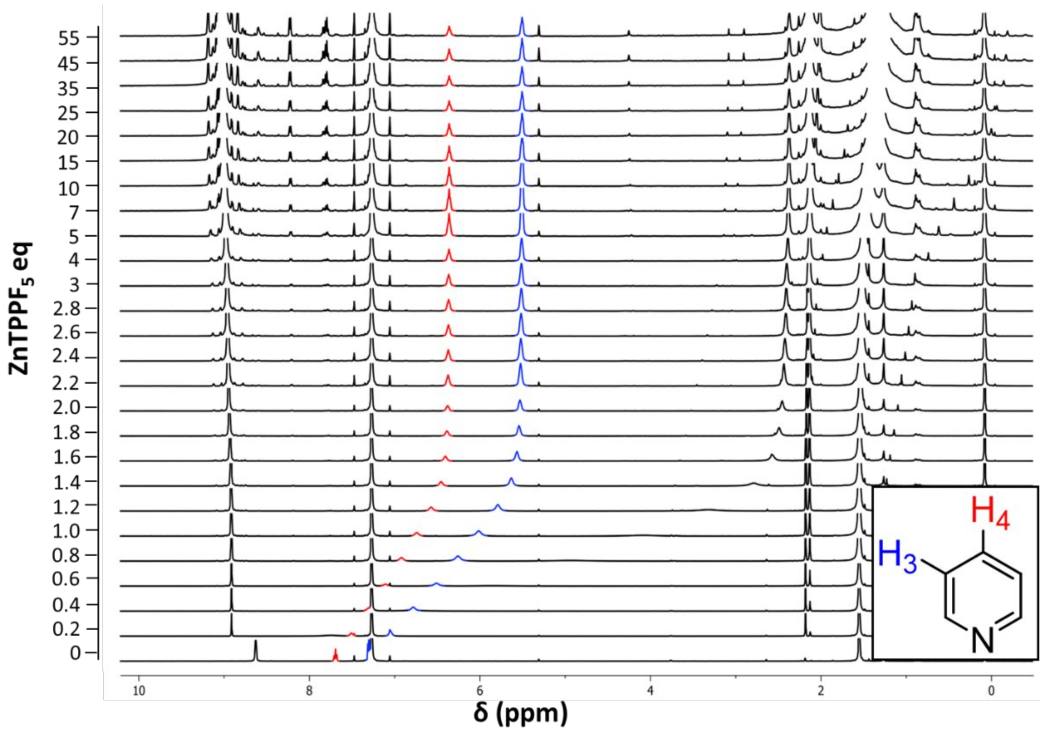

Figure S49. Stacked ^{1}H NMR spectra of the titration of pyridine with up to 55 equivalents of $ZnTPPF_5$. The signals of the pyridine that were followed are indicated in blue (H_3) and red (H_4), respectively. The resulting binding constant was determined to be $(1.3 \pm 0.3) \times 10^{4}$ M^{-1} from nonlinear regression to a 1:1 model.

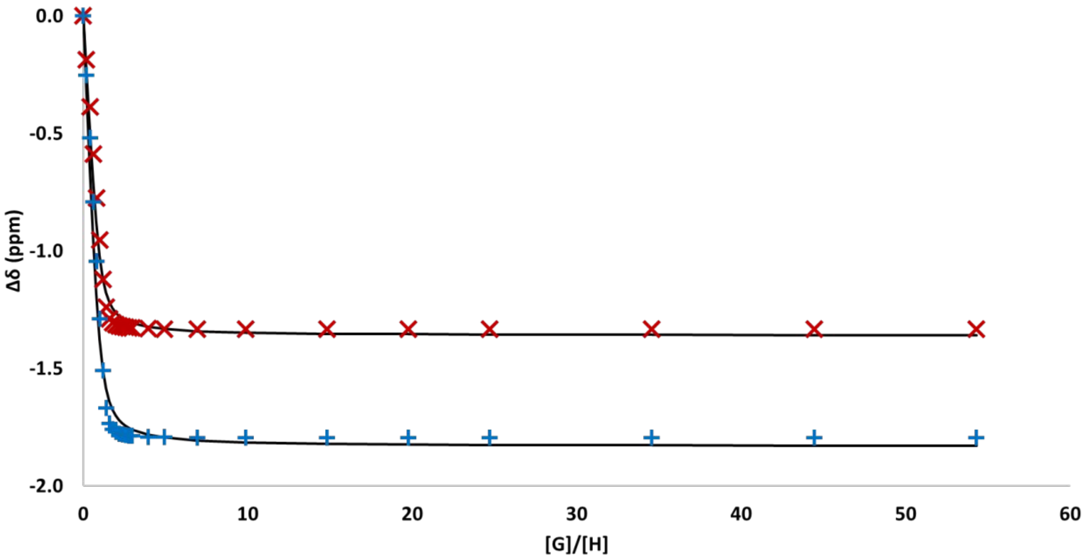

Figure S50. Changes in the chemical shifts of the pyridine signals (H_3 in blue and H_4 in red) vs [G]/[H], and the theoretical values obtained from the non-linear regression to a 1:1 binding isotherm (black lines).

Titration study of Sb(3-py)$_3$ with ZnTPPF$_5$

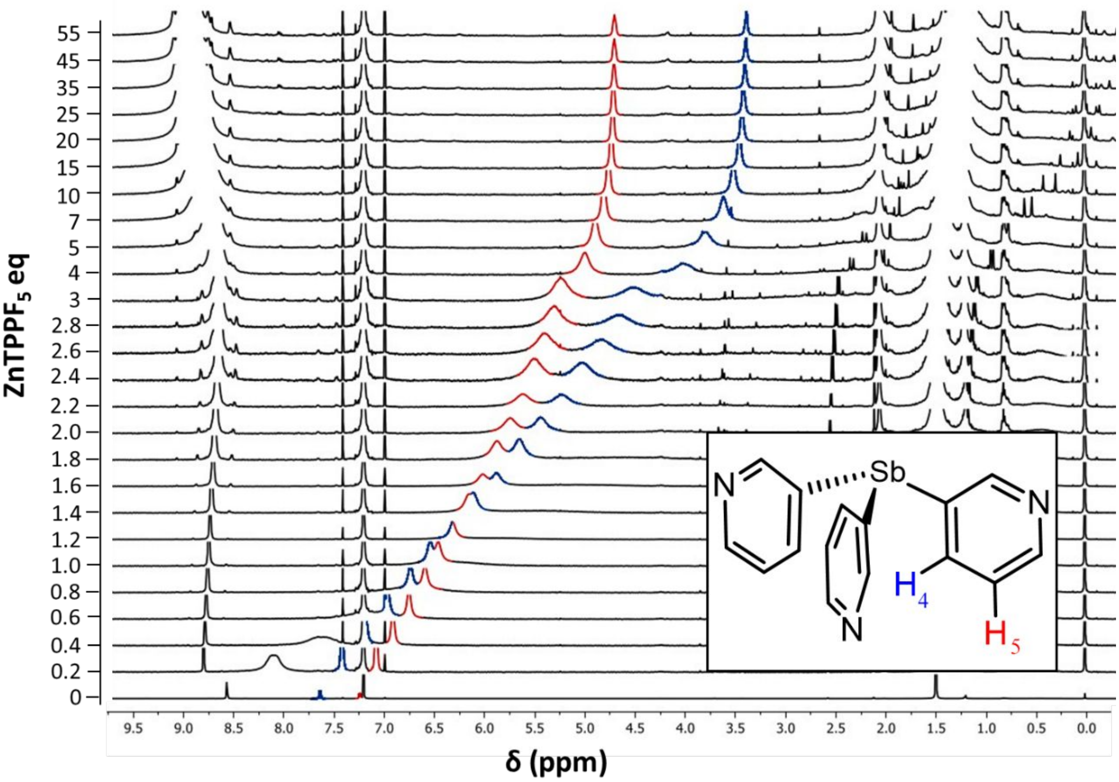

Figure S51. Stacked ^{1}H NMR spectra of the titration of ligand Sb(3-py)$_3$ with up to 55 equivalents of ZnTPPF$_5$. The signals of the ligands that were followed are indicated in blue (H$_4$) and red (H$_5$), respectively.

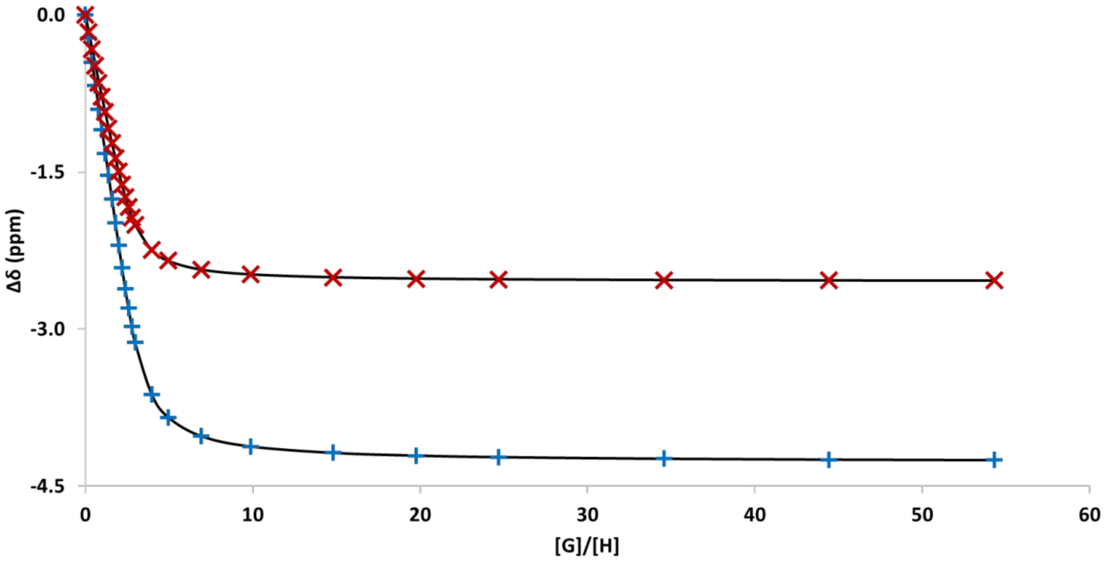

Figure S52. Changes in the chemical shifts of the Sb(3-py)$_3$ signals (H$_4$ in blue and H$_5$ in red) vs [G]/[H], and theoretical values obtained from the non-linear regression to a 1:3 binding isotherm (black lines).

Titration study of $Bi(3\text{-}py)_3$ with $ZnTPPF_5$

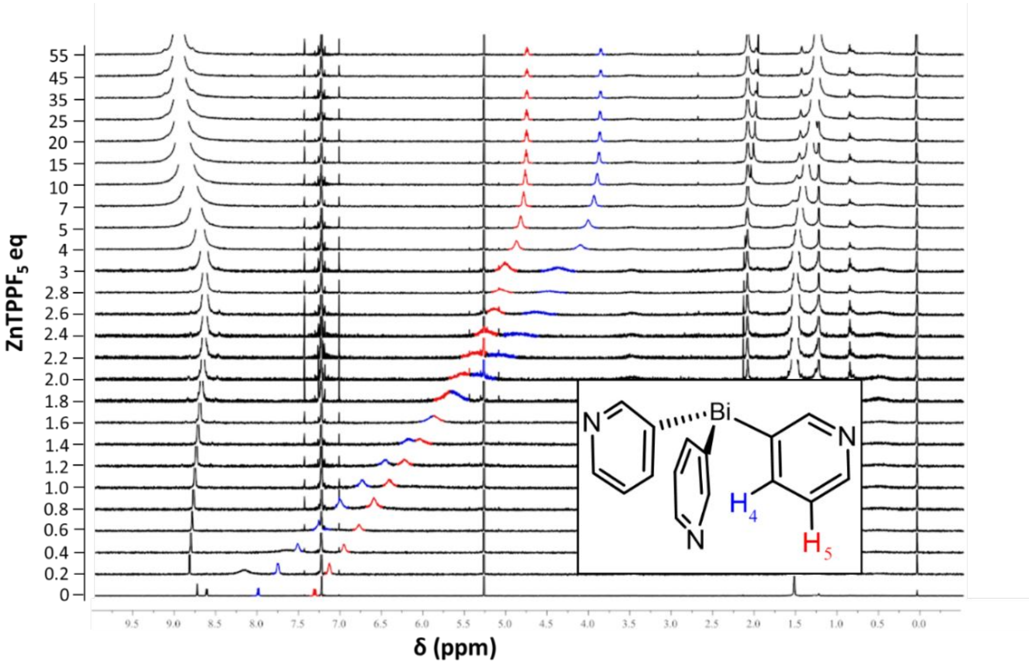

Figure S53. Stacked ^{1}H NMR spectra of the titration of ligand $Bi(3\text{-}py)_3$ with up to 55 equivalents of $ZnTPPF_5$. The signals of the ligands that were followed are indicated in blue (H_4) and red (H_5), respectively.

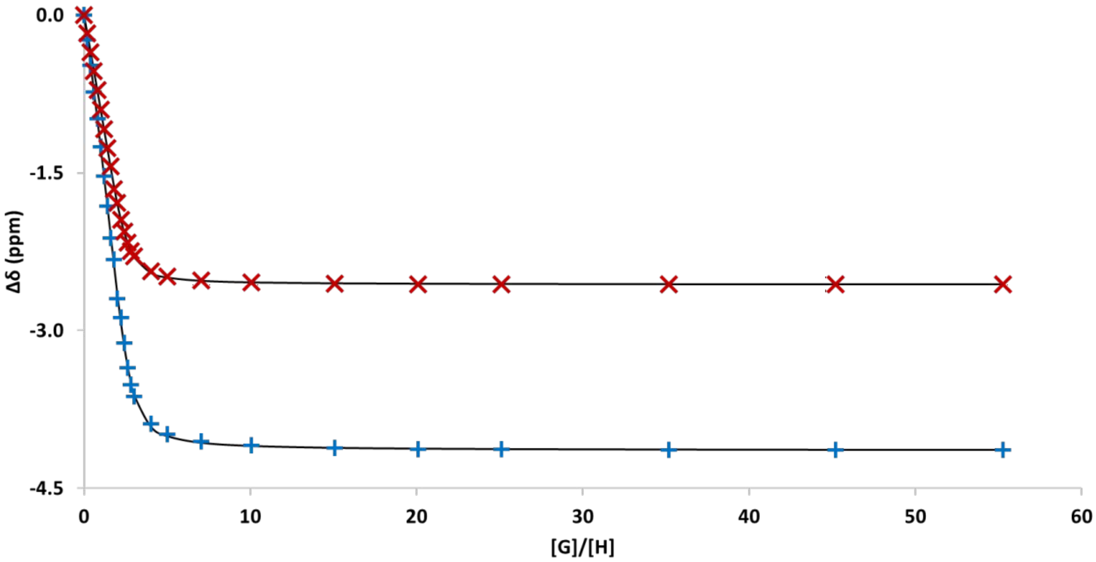

Figure S54. Changes in the chemical shifts of the Bi(3-py)3 signals (H4 in blue and H5 in red) vs [G]/[H], and the theoretical values obtained from the non-linear regression to a 1:3 binding isotherm (black lines).

X-ray crystallographic studies

Details of the data collections and structural refinements are given in Table S10. Further details of the methods of refinement of the structures are as follows:

1·ZnTPPOMe
One of the aryl groups was modeled as disordered over two positions with the help of geometrical restraints (DFIX, SADI and FLAT) as well as ADP restraints (RIGU). Two other aryl groups showed symptoms of an incipient disorder that was not modelled and for which RIGU constraints were used.

The structure also contains poorly resolved solvent that could not be modelled. The crystallization solvent was $CHCl_3$/hexane, and it is difficult to define reasonable molecules from the electron density. A solvent mask was calculated, and 70 electrons were found in a void with a volume of 282 cubic angstroms. This could be consistent with the presence of roughly one hexane molecule per unit cell (i.e., roughly 0.5 hexane molecules per formula unit), which accounts for 50 electrons per unit cell. However, due to the similar electron count of hexane and $CHCl_3$ (one $CHCl_3$ accounts for 58 electrons), the identity and quantity present of the solvent is uncertain, and a mixture of both solvents could even be present.

2·ZnTPPOMe
Several crystals from different batches were studied and all produced similar results. In all cases, the diffracted intensity was quite weak and dropped off rapidly with increasing diffraction angle. The presented structure is based on the best data set, for which the data is still very weak, and I/sigma(I) falls below 3 at around 1.4 Å resolution. The very high angle data was dominated by noise [I/sigma(I) < 1.0] and therefore was omitted at a resolution <0.9 Å. As a result of the crystal being very weakly diffracting, the R(int) value is high. Due to the poor quality of the crystals and less-than-ideal resolution**,** geometrical restraints (SADI) and thermal parameter restraints (RIGU) were applied. The latter were applied to all non-hydrogen atoms except for Zn and Bi, resulting in a fully anisotropic model.

Although the structure of **2·ZnTPPOMe** is clear, it contains solvent molecules that are not clearly resolved. The crystallization solvent was $CHCl_3$/hexane, and it is difficult to define reasonable molecules from the electron density. A solvent mask was calculated, and 216 electrons were found in a volume of 1044 cubic angstroms in two voids per unit cell. This could be consistent with the presence of roughly one hexane molecule per formula unit (i.e., roughly 4 hexane molecules per unit cell), which accounts for 200 electrons per unit cell. However, due to the similar electron count (4 $CHCl_3$ account for 232 electrons), the identity and quantity present of the solvent is uncertain, and a mixture of both solvents could even be present.

1·ZnTPPBr
One aryl group exhibited positional disorder and was modelled over two positions with enhanced rigid-body restraints applied to control the ADPs (RIGU). The site occupancy

was refined for the two positions and constrained to sum to unit. Restraints were applied to maintain sensible bond distances and geometry.

Two $CHCl_3$ solvent molecules were disordered over two positions. These solvent molecules were refined as rigid bodies with displacement parameter restraints (RIGU and SIMU), and their site occupancies were refined so that their sum equals 1.0.

2·ZnTPPBr
Several crystals from different batches were studied and all produced similar results. In all cases, the diffracted intensity was quite weak and dropped off rapidly with increasing diffraction angle. The presented structure is based on the best data set, for which the data is still very weak, and as a result of the crystal being very weakly diffracting, the *R*(int) value is relatively high.

One of the aryl groups was modeled as disordered over two positions with the help of geometrical restraints (DFIX, FLAT, SADI) as well as ADP restraints (RIGU). Two poorly resolved molecules of $CHCl_3$ were present in the lattice. These solvent molecules were refined using restraints to maintain sensible bond distances and geometry and they were treated as rigid bodies for the final refinement cycles with ADP restraints (RIGU).

1·MgTPPBr
The residual electron density shows poorly resolved solvent molecules, and it appears that it could comprise $CHCl_3$ molecules with overlaid hexane molecules. A poorly defined molecule of $CHCl_3$ seems to be present in the asymmetric unit and was refined as a rigid body with the site occupancies constrained to 0.5. After defining the CHCl3 molecule, clear peaks in the residual electron density suggested superimposed hexane molecules, which suggests that they occupy the site when the half-occupancy $CHCl_3$ is not present. A poorly defined hexane molecule on the inversion center was also treated as a rigid body, leading to a suboptimal refinement. This model produces R1 = 8.8% and WR2 = 29.5%. In addition to this, several clear peaks in the residual electron density in solvent-accessible voids were present, but there were no clearly identifiable solvent molecules. The solution provided involves removing these poorly resolved solvent molecules and applying the olex2 solvent mask (i.e., leaving only compound **1**·MgTPPBr with no solvent molecules). This leads to lower R-values (R1 = 5.9% and WR2 = 13.6%) and better bond and angle precision of compound **1**·MgTPPBr.

In the calculated solvent mask, 283 electrons were found in a volume of 1179 cubic angstroms in one void per unit cell volume. The crystallization solvent was $CHCl_3$/hexane, and this could be consistent with the presence of roughly two hexane and three $CHCl_3$ molecules per unit cell, which account for 274 electrons per unit cell. However, the exact quantity of the solvent remains effectively unknown.

1·ZnTPPF$_5$
There is one disordered $CHCl_3$ molecule near a crystallographic threefold axis, which has been refined with an occupation fixed at 0.33 and applying Rigu restraints.

2·ZnTPPF$_5$
There is one disordered $CHCl_3$ molecule near a crystallographic threefold axis, which has been refined with an occupation fixed at 0.33 and applying Rigu restraints.

Table S10. Crystallographic data.

Identification code	**1·ZnTPPOMe**	**2·ZnTPPOMe**
CCDC Number	2503793	2503794
Empirical formula	$C_{111}H_{84}N_{11}O_8SbZn_2$	$C_{111}H_{84}BiN_{11}O_8Zn_2$
Formula weight	1952.38	2039.61
Temperature/K	220(2)	219.9(6)
Crystal system	triclinic	monoclinic
Space group	P-1	$P2_1/c$
a/Å	10.3258(4)	21.7962(19)
b/Å	17.4629(7)	20.6552(14)
c/Å	26.4670(8)	23.1415(18)
α/°	95.894(3)	90
β/°	92.996(3)	106.770(9)
γ/°	94.305(3)	90
Volume/Å^3	4725.2(3)	9975.4(14)
Z	2	4
ρ_{calc}g/cm^3	1.372	1.358
μ/mm^{-1}	0.853	2.299
F(000)	2004	4136
Crystal size/mm^3	0.392 × 0.092 × 0.069	0.42 × 0.065 × 0.006
Radiation	MoKα (λ = 0.71073)	Mo Kα (λ = 0.71073)
2Θ range for data collection/°	4.342 to 50.136	6.654 to 46.514
Index ranges	-10 ≤ h ≤ 12, -20 ≤ k ≤ 20, -31 ≤ l ≤ 28	-21 ≤ h ≤ 24, -22 ≤ k ≤ 21, -24 ≤ l ≤ 25
Reflections collected	39056	36239
Independent reflections	16708 [R_{int} = 0.0727, R_{sigma} = 0.1312]	14285 [R_{int} = 0.2488, R_{sigma} = 0.3955]
Data/restraints/parameters	16708/292/1280	14285/1072/1204
Goodness-of-fit on F^2	0.991	0.908
Final R indexes [I>=2σ (I)]	R_1 = 0.0629, wR_2 = 0.1126	R_1 = 0.0902, wR_2 = 0.1266
Final R indexes [all data]	R_1 = 0.1331, wR_2 = 0.1426	R_1 = 0.2556, wR_2 = 0.1827
Largest diff. peak/hole /e Å^{-3}	0.67/-0.57	1.20/-0.80

Identification code	**1·ZnTPPBr**	**2·ZnTPPBr**	**1·MgTPPBr**
CCDC Number	2503795	2503792	2503789
Empirical formula	$C_{105}H_{62}Br_8Cl_6N_{11}SbZn_2$	$C_{105}H_{62}BiBr_8Cl_6N_{11}Zn_2$	$C_{162}H_{96}Br_{12}Mg_3N_{18}Sb_2$
Formula weight	2582.12	2669.35	3569.91
Temperature/K	220.00(14)	220.00(14)	220(2)
Crystal system	triclinic	triclinic	triclinic
Space group	P-1	P-1	P-1
a/Å	16.7946(6)	16.7925(9)	11.3044(4)
b/Å	16.9813(5)	16.9573(11)	17.0280(6)
c/Å	19.4749(7)	19.4756(13)	22.8856(6)
α/°	80.805(3)	80.745(5)	83.830(3)
β/°	75.575(3)	75.619(5)	85.986(3)
γ/°	69.535(3)	69.238(6)	85.554(3)
Volume/Å^3	5023.3(3)	5006.9(6)	4358.1(3)
Z	2	2	1
ρ_{calc}g/cm^3	1.707	1.771	1.36
μ/mm^{-1}	4.136	5.634	3.121
F(000)	2524	2588	1752
Crystal size/mm^3	0.36 × 0.26 × 0.13	0.34 × 0.16 × 0.14	0.6 × 0.131 × 0.073
Radiation	Mo Kα (λ = 0.71073)	Mo Kα (λ = 0.71073)	Mo Kα (λ = 0.71073)
2Θ range for data collection/°	4.144 to 49.538	4.586 to 49.518	4.112 to 49.8
Index ranges	-19 ≤ h ≤ 19, -19 ≤ k ≤ 20, -22 ≤ l ≤ 22	-19 ≤ h ≤ 19, -19 ≤ k ≤ 19, -19 ≤ l ≤ 22	-13 ≤ h ≤ 13, -17 ≤ k ≤ 20, -27 ≤ l ≤ 26
Reflections collected	93561	40649	35310
Independent reflections	17186 [R_{int} = 0.0896, R_{sigma} = 0.0741]	17071 [R_{int} = 0.0699, R_{sigma} = 0.1138]	15130 [R_{int} = 0.0655, R_{sigma} = 0.1273]
Data/restraints/parameters	17186/173/1273	17071/180/1250	15130/0/889
Goodness-of-fit on F^2	1.035	1.061	0.971
Final R indexes [I>=2σ (I)]	R_1 = 0.0692, wR_2 = 0.1643	R_1 = 0.0981, wR_2 = 0.2653	R_1 = 0.0592, wR_2 = 0.1188
Final R indexes [all data]	R_1 = 0.1183, wR_2 = 0.1927	R_1 = 0.1453, wR_2 = 0.2967	R_1 = 0.1217, wR_2 = 0.1361
Largest diff. peak/hole / e Å^{-3}	1.40/-1.15	4.21/-1.48	1.52/-1.10

Identification code	**1·ZnTPPF$_5$**	**2·ZnTPPF$_5$**
CCDC Number	2503790	2503791
Empirical formula	$C_{151}H_{40}Cl_{12}F_{60}N_{15}SbZn_3$	$C_{151}H_{40}BiCl_{12}F_{60}N_{15}Zn_3$
Formula weight	3947.24	4034.47
Temperature/K	220(2)	220(2)
Crystal system	cubic	cubic
Space group	Pa-3	Pa-3
a/Å	31.0547(3)	31.0434(4)
b/Å	31.0547(3)	31.0434(4)
c/Å	31.0547(3)	31.0434(4)
α/°	90	90
β/°	90	90
γ/°	90	90
Volume/Å^3	29948.9(9)	29916.2(12)
Z	8	8
ρ_{calc}g/cm^3	1.751	1.792
μ/mm^{-1}	1.001	1.997
F(000)	15488.0	15744.0
Crystal size/mm^3	0.384 × 0.333 × 0.252	0.665 × 0.575 × 0.298
Radiation	MoKα (λ = 0.71073)	Mo Kα (λ = 0.71073)
2Θ range for data collection/°	4.908 to 50.04	4.732 to 50.06
Index ranges	-8 ≤ h ≤ 31, -24 ≤ k ≤ 36, -24 ≤ l ≤ 31	-30 ≤ h ≤ 7, -23 ≤ k ≤ 36, -24 ≤ l ≤ 32
Reflections collected	27725	27743
Independent reflections	8825 [R_{int} = 0.0421, R_{sigma} = 0.0504]	8828 [R_{int} = 0.0630, R_{sigma} = 0.0787]
Data/restraints/parameters	8825/15/751	8828/15/751
Goodness-of-fit on F^2	1.035	1.015
Final R indexes [I>=2σ (I)]	R_1 = 0.0505, wR_2 = 0.1193	R_1 = 0.0557, wR_2 = 0.1221
Final R indexes [all data]	R_1 = 0.0865, wR_2 = 0.1412	R_1 = 0.1072, wR_2 = 0.1483
Largest diff. peak/hole / e Å^{-3}	0.77/-0.56	1.15/-1.18

Table S11. Selected bond length and angles.

(E = Sb, Bi)	E - C_{py}	C_{py} - E - C_{py}	N - M (M = Zn, Mg)
**1·ZnTPPF$_5$**	2.142(5)	95.44(17)	2.137(4)
**2·ZnTPPF$_5$**	2.227(6)	94.3(2)	2.127(5)
**1·ZnTPPOMe**	2.132(5) - 2.157(7)	95.7(2) - 100.2(2)	2.180(5) - 2.185(5)
**2·ZnTPPOMe**	2.25(2) - 2.30(1)	92.0(6) - 96.1(6)	2.16(1)
**1·ZnTPPBr**	2.139(9) - 2.16(1)	96.4(4) - 99.3 (4)	2.134(7) - 2.161(7)
**2·ZnTPPBr**	2.25(2) – 2.26(2)	93.1(7) – 97.9(6)	2.12(1) - 2.13(1)
**1·MgTPPBr**	2.136(7) - 2.145(5)	92.6(2) - 97.7(7)	2.173(5) - 2.300(5)

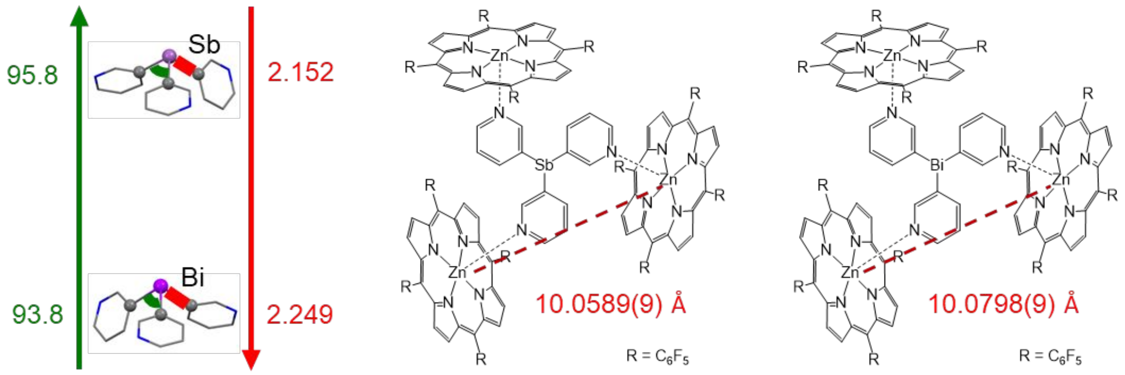

Figure S55. Determination of the size of the capsules **1**·ZnTPPF$_5$ (10.0589(9) Å) and **2**·ZnTPPF$_5$ (10.0798(9) Å) through Zn–Zn distances.

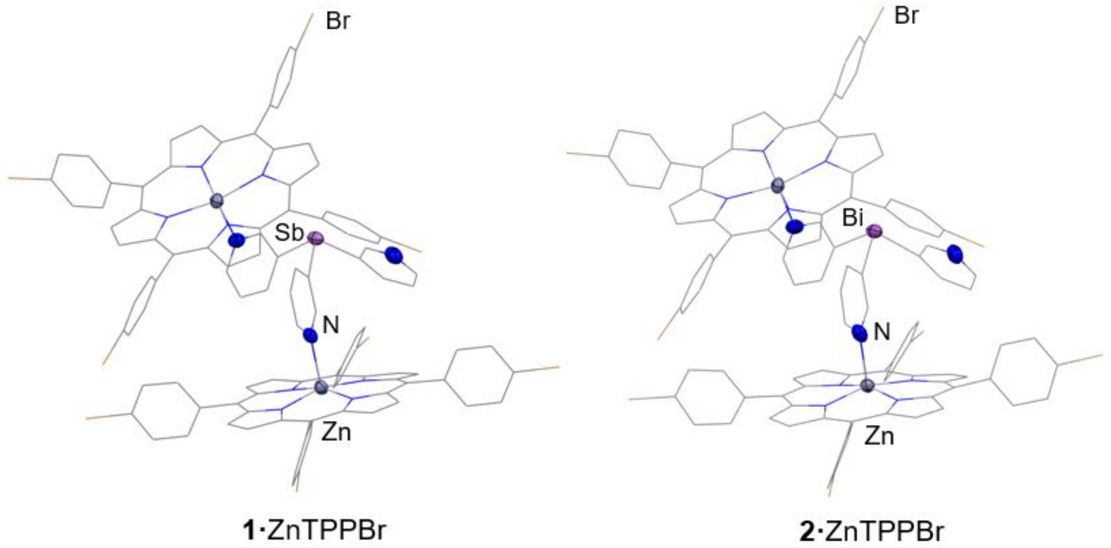

Figure S56. X-ray structures of the complexes **1**·ZnTPPBr (a) and **2**·ZnTPPBr (b). In both structures, the ligand coordinates two porphyrins through N–Zn interactions, while the third pyridyl arm remains uncoordinated. Displacement ellipsoids shown at 50% probability.

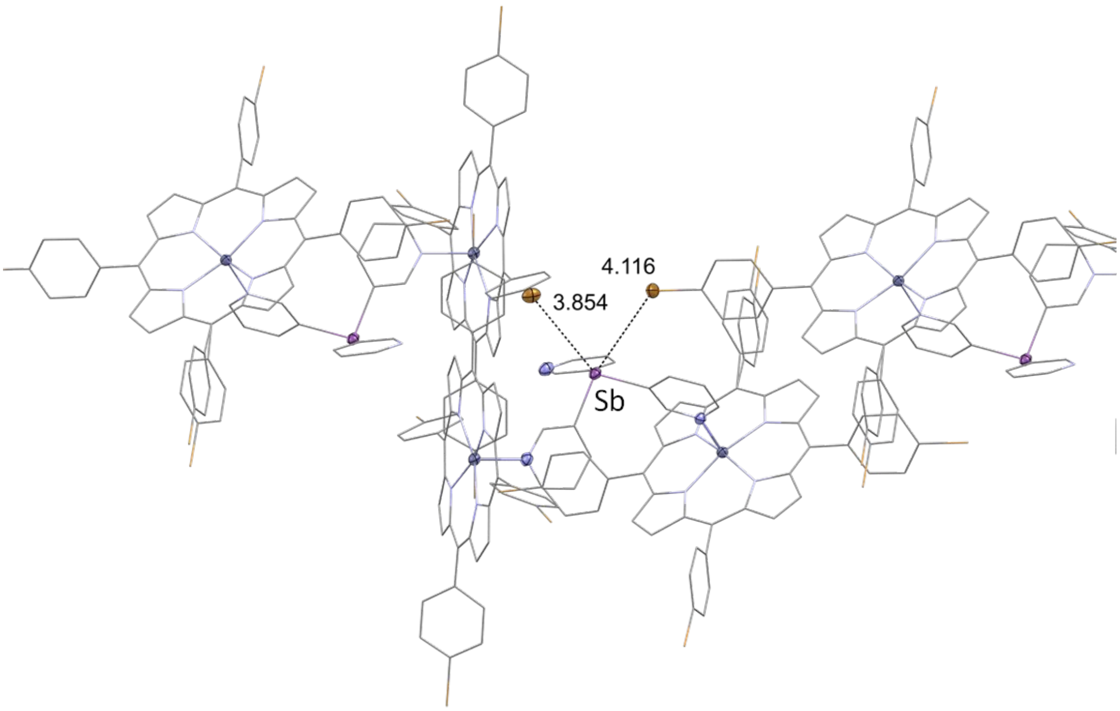

Figure S57. X-ray structure of **1**·ZnTPPBr showing intermolecular contacts between Sb···Br within (3.854 Å) or slightly above (4.116 Å) the sum of the van der Waals radii (cf. 3.89 Å ΣvdW (Sb−Br)).

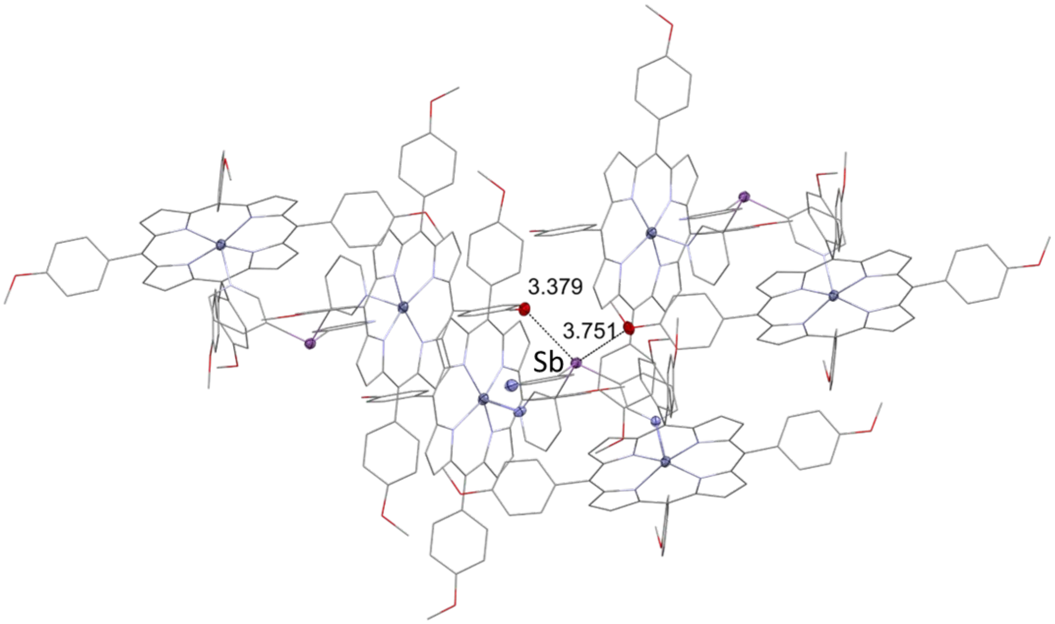

Figure S58. X-ray structure of **1**·ZnTPPOMe showing intermolecular contacts between Sb···O within (3.379 Å) or slightly above (3.751 Å) the sum of the van der Waals radii (cf. 3.58 Å ΣvdW (Sb−O)).

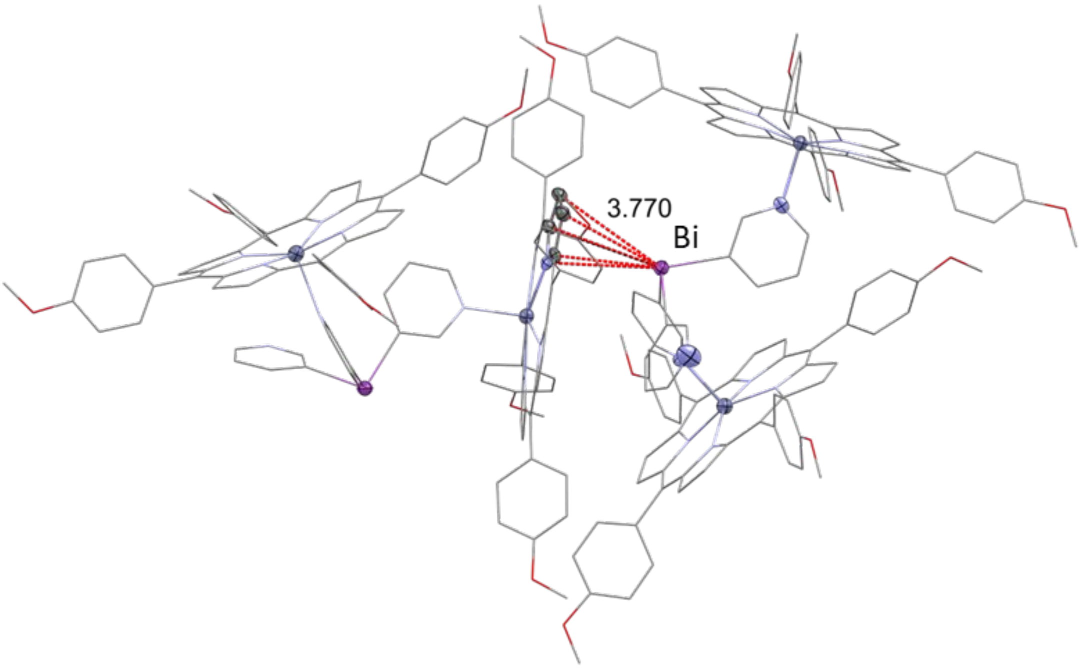

Figure S59. X-ray structure of 1·ZnTPPOMe showing intermolecular bismuth···π pyrrole contacts. Bi–pyrrolecentroid distance 3.770 Å.

High-resolution mass data

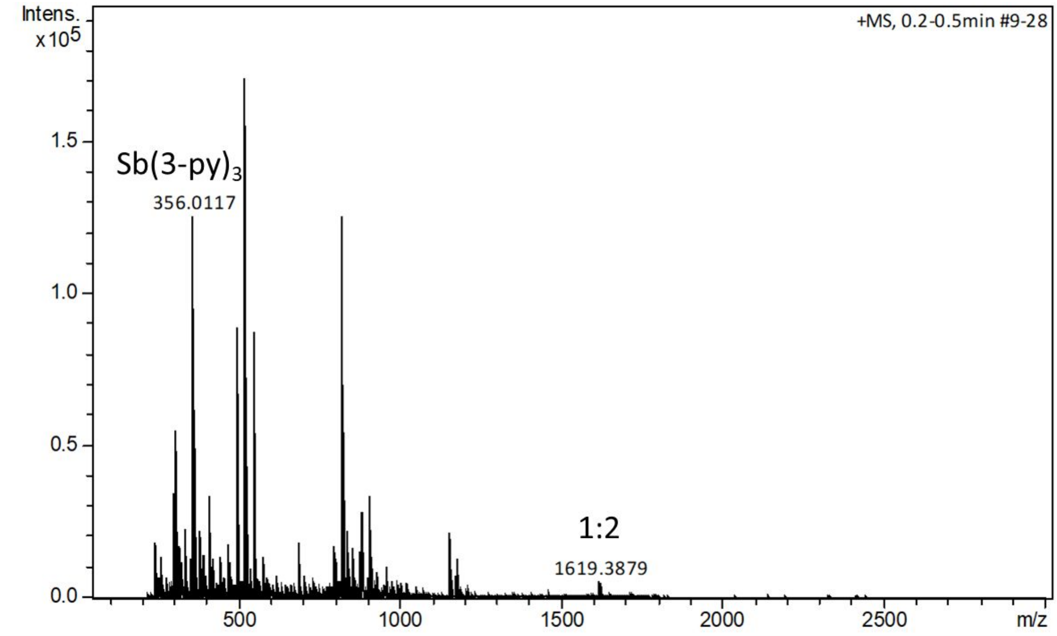

Figure S60. Full HR-MS (ESI-TOF) of {[Sb(3-py)$_3$]·(ZnTPPOMe)$_2$} (1·ZnTPPOMe) showing peaks corresponding to Sb(3-py)$_3$ (found: 356.0117) and 1:2 adduct [M+H]$^+$ (found: 1952.4101).

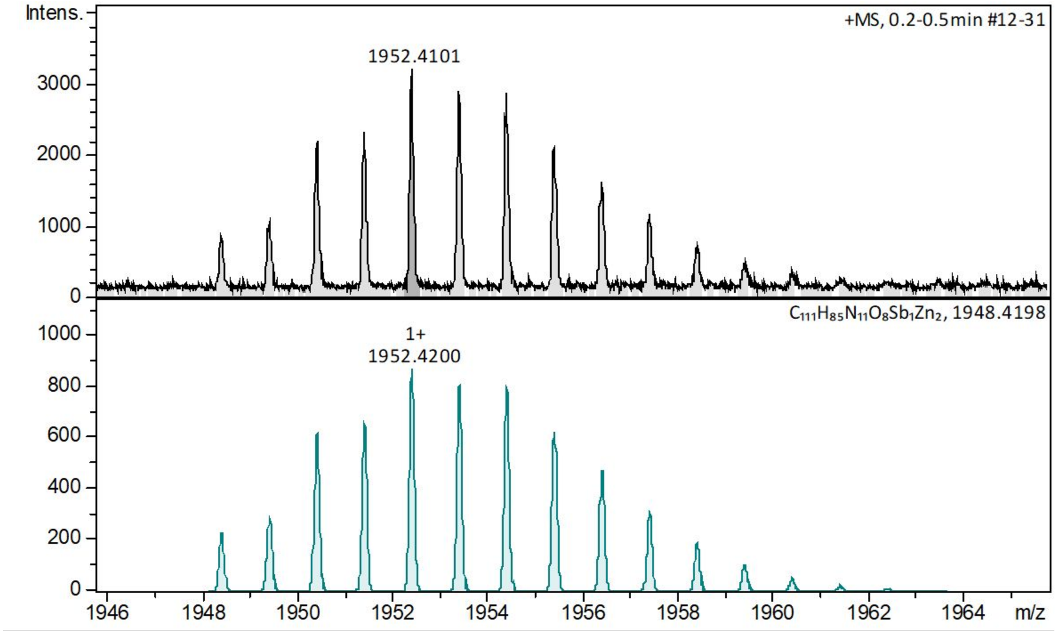

Meas. m/z	m/z	err [ppm]	Ion Formula
1952.4101	1952.4200	2.0	C111H84N11O8SbZn2

Figure S61. HR-MS (ESI-TOF) (positive mode) of {[Sb(3-py)$_3$]·(ZnTPPOMe)$_2$} (1·ZnTPPOMe) showing the expected [M+H]$^+$ peak at m/z 1952.4101 (calcd 1952.4200; 2.0 ppm error) (top) and a simulation (bottom).

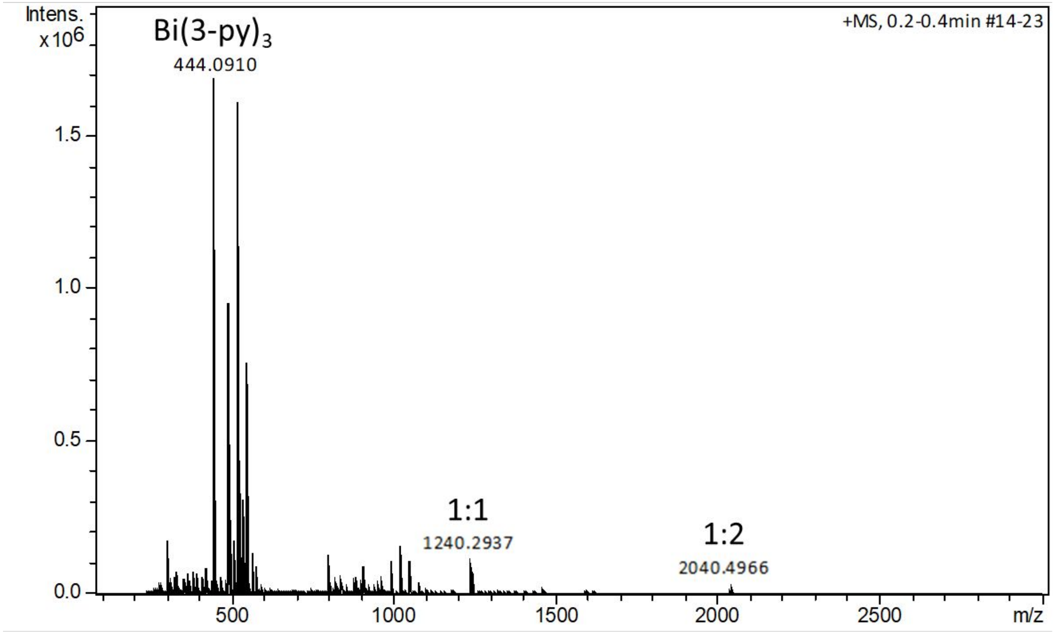

Figure S62. Full HR-MS (ESI-TOF) of {[Bi(3-py)$_3$]·(ZnTPPOMe)$_2$} (2·ZnTPPOMe) showing peaks corresponding to the Bi(3-py)$_3$ (found: 444.0910), 1:1 adduct $[M+H]^+$ (found: 1240.2937) and 1:2 adduct $[M+H]^+$ (found: 2040.4966).

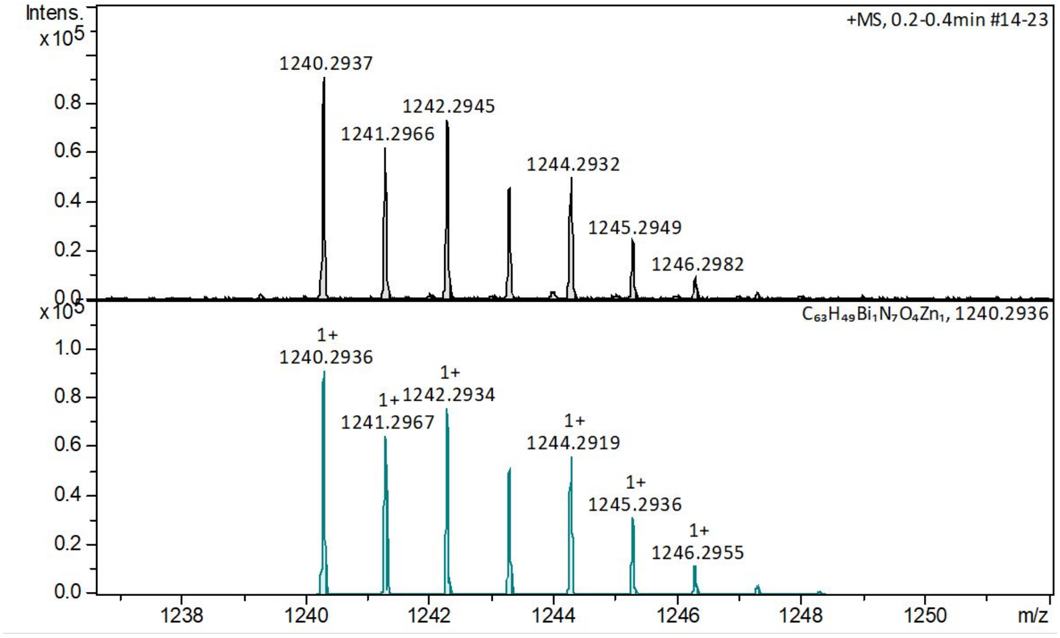

Meas. m/z	m/z	err [ppm]	mSigma	Ion Formula
1240.2937	1240.2936	-0.1	42.2	C63H49BiN7O4Zn

Figure S63. HR-MS (ESI-TOF) (positive mode) of {[Bi(3-py)$_3$]·(ZnTPPOMe)$_2$} (**2**·ZnTPPOMe) showing the $[M+H]^+$ peak at *m/z* 1240.2937 (calcd 1240.2936; -0.1 ppm error) (top) and a simulation (bottom) corresponding to the 1:1 adduct.

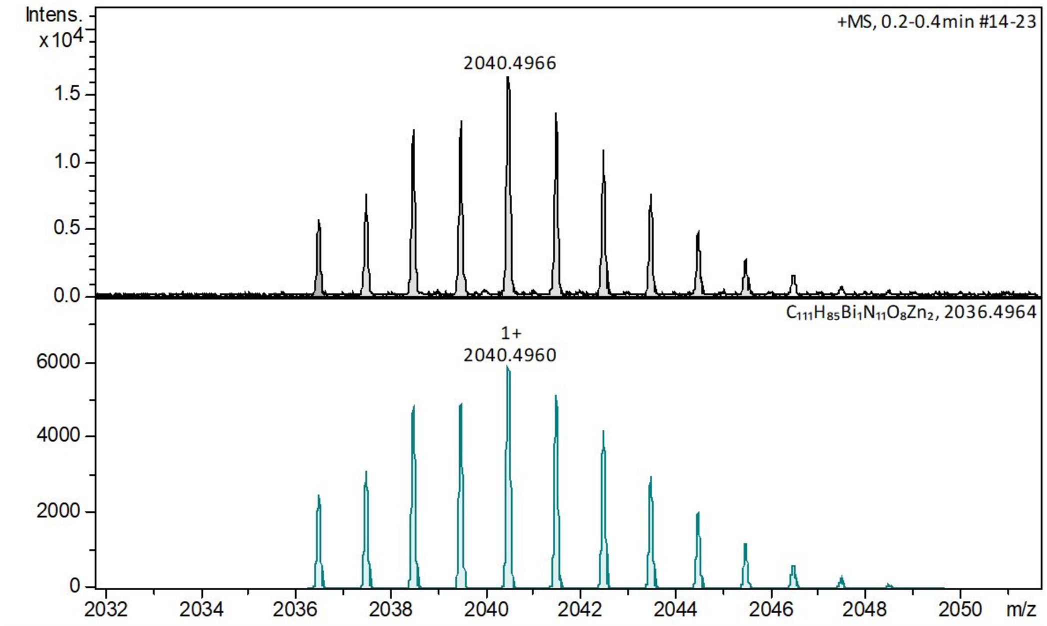

Meas. m/z	m/z	err [ppm]	Ion Formula
2040.4966	2040.4960	0.6	C111H85BiN11O8Zn2

Figure S64. HR-MS (ESI-TOF) (positive mode) of {[Bi(3-py)$_3$]·(ZnTPPOMe)$_2$} (**2**·ZnTPPOMe) showing the expected [M+H]$^+$ peak at *m/z* 2040.4966 (calcd 2040.4960; 0.6 ppm error) (top) and a simulation (bottom).

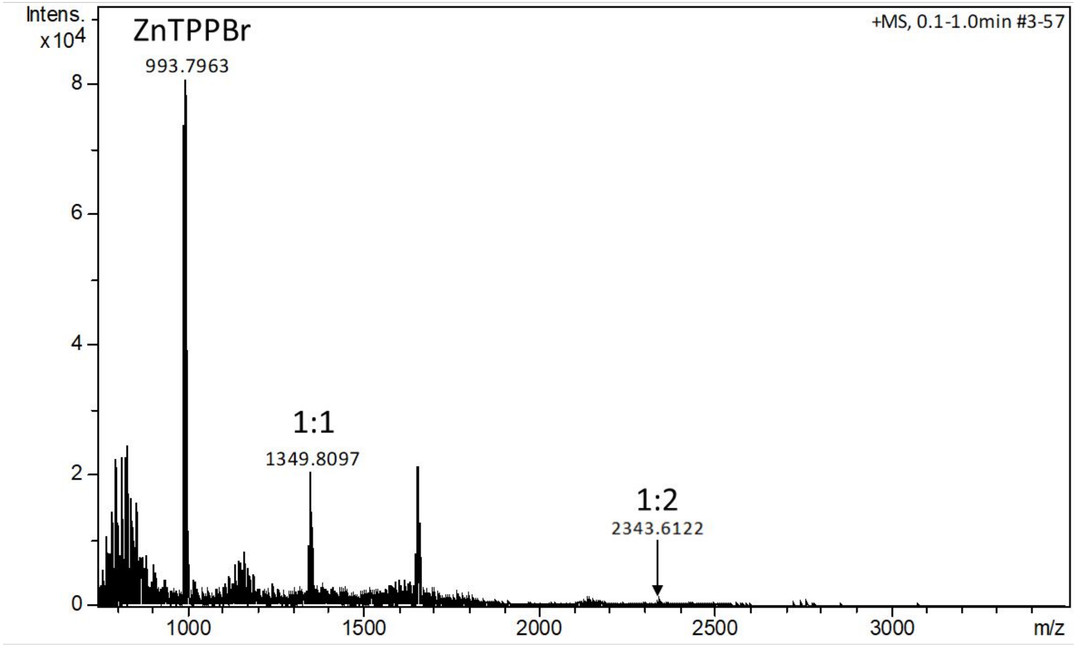

Figure S65. Full HR-MS (ESI-TOF) of {[Sb(3-py)$_3$]·(ZnTPPBr)$_2$} (1·ZnTPPBr) showing peaks corresponding to ZnTPPBr (found: 993.7963), 1:1 adduct [M+H]$^+$ (found: 1349.8097) and 1:2 adduct [M+H]$^+$ (found: 2343.6122).

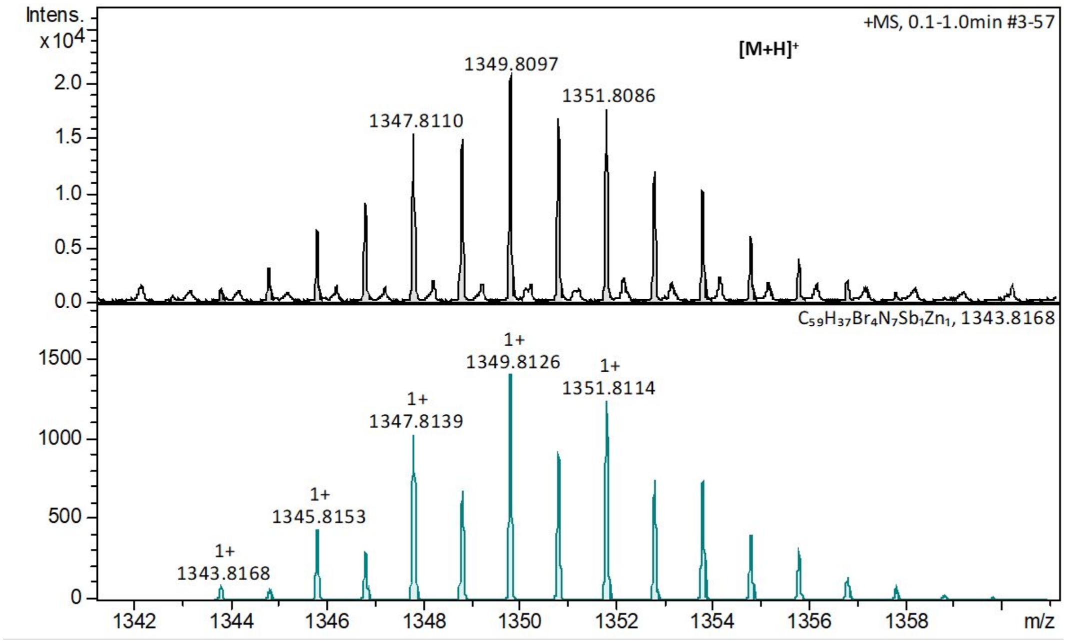

Meas. m/z	m/z	err [ppm]	Ion Formula
1349.8097	1349.8126	-2.9	C59H37Br4N7SbZn

Figure S66. HR-MS (ESI-TOF) (positive mode) of {[Sb(3-py)$_3$]·(ZnTPPBr)$_2$} (1·ZnTPPBr) showing the [M+H]$^+$ peak at m/z 1348.8034 (calcd 1238.8048; 1.3 ppm error) (top) and a simulation (bottom) corresponding to the 1:1 adduct.

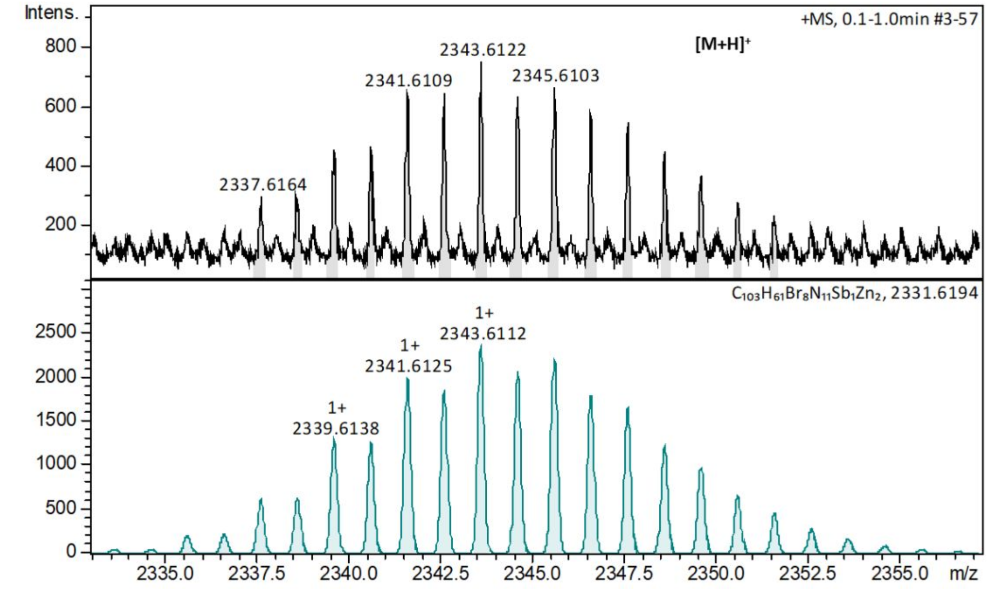

Meas. m/z	m/z	err [ppm]	Ion Formula
2343.6122	2343.6112	1.0	C103H61Br8N11SbZn2

Figure S67. HR-MS (ESI-TOF) (positive mode) of {[Sb(3-py)$_3$]·(ZnTPPBr)$_2$} (**1**·ZnTPPBr) showing the expected [M+H]$^+$ peak at *m/z* 2343.6122 (calcd 2342.6112; 1.0 ppm error) (top) and a simulation (bottom).

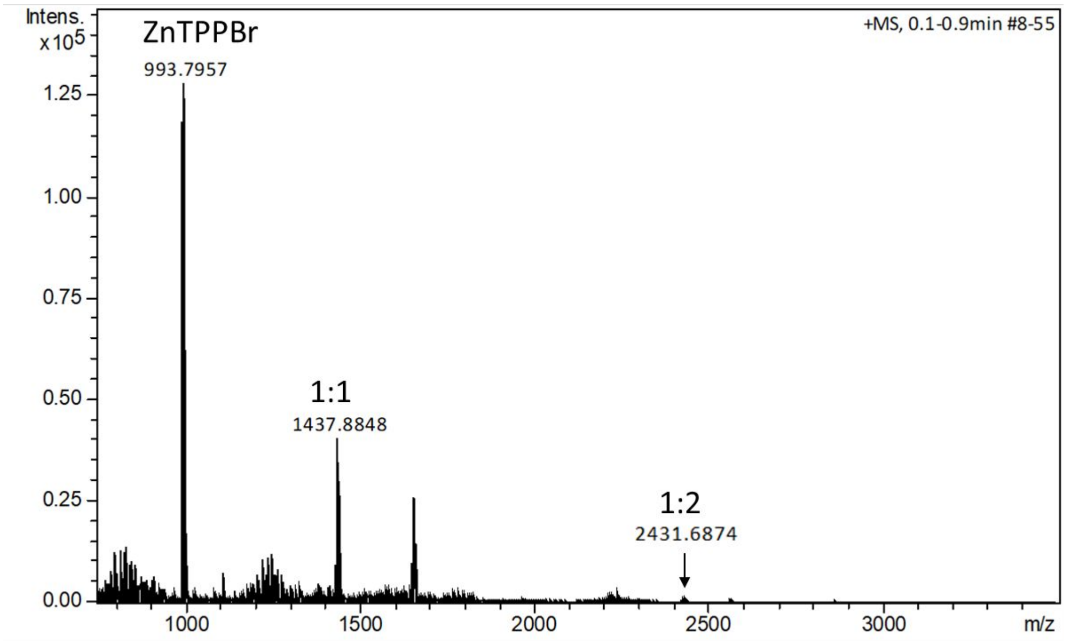

Figure S68. Full HR-MS (ESI-TOF) of {[Bi(3-py)$_3$]·(ZnTPPBr)$_2$} (1·ZnTPPBr) showing peaks corresponding to ZnTPPBr (found: 993.7957), 1:1 adduct [M+H]$^+$ (found: 1437.8848) and 1:2 adduct [M+H]$^+$ (found: 2431.6874).

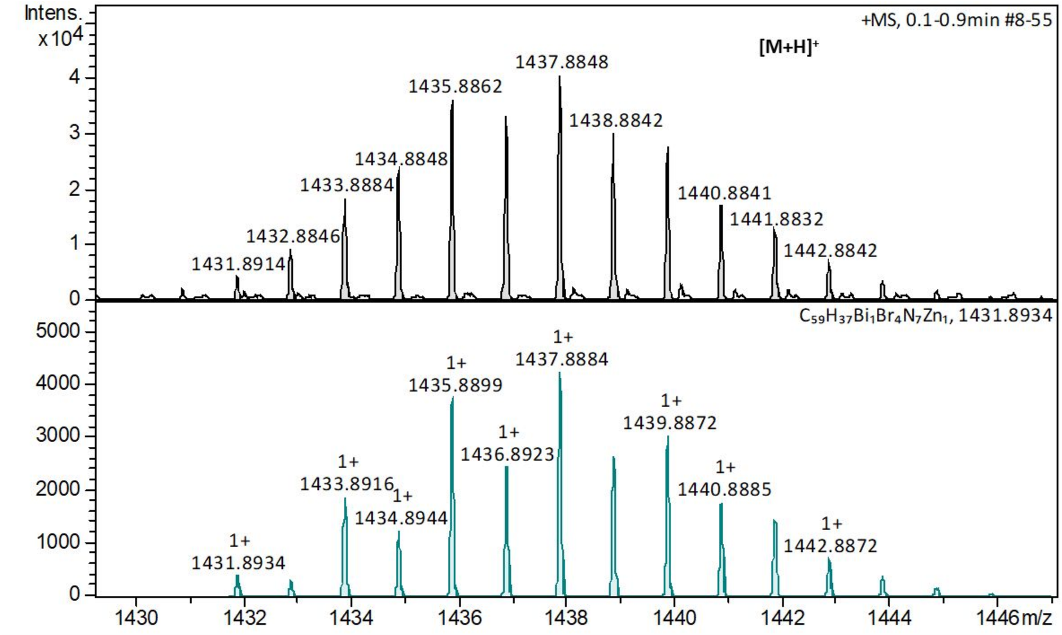

Meas. m/z	m/z	err [ppm]	Ion Formula
1437.8848	1437.8884	-3.6	C59H37Br4N7BiZn

Figure S69. HR-MS (ESI-TOF) (positive mode) of {[Bi(3-py)$_3$]·(ZnTPPBr)$_2$} (**2**·ZnTPPBr) showing the [M+H]$^+$ peak at *m/z* 1437.8848 (calcd 1437.8848; -3.6 ppm error) (top) and a simulation (bottom) corresponding to the 1:1 adduct.

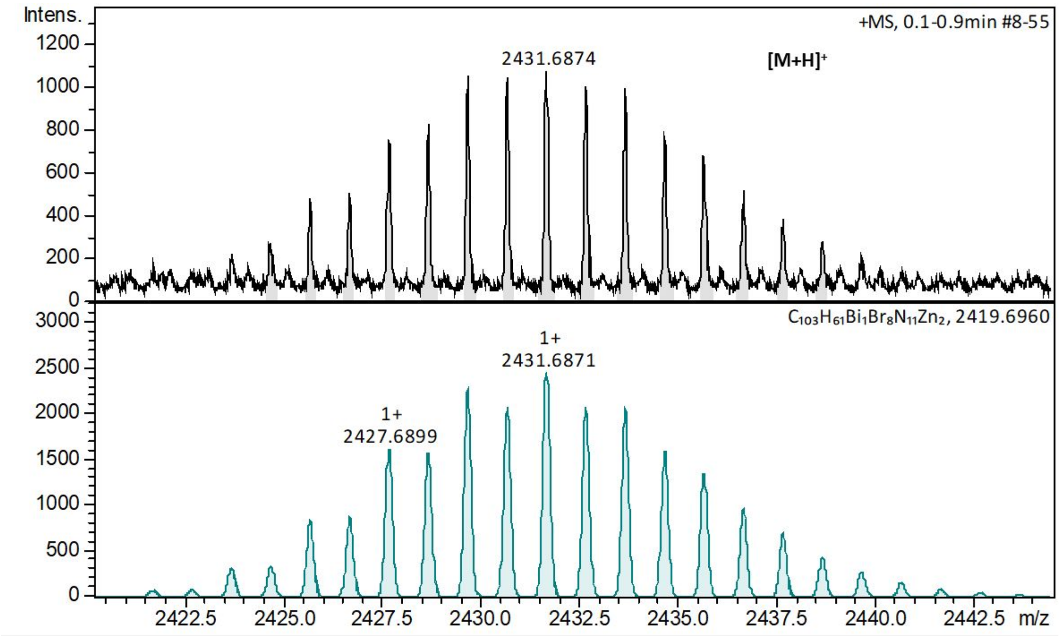

Meas. m/z	m/z	err [ppm]	Ion Formula
2431.6874	2431.6871	0.3	C103H61Br8N11BiZn2

Figure S70. HR-MS (ESI-TOF) (positive mode) of {[Bi(3-py)$_3$]·(ZnTPPBr)$_2$} (**2**·ZnTPPBr) showing the expected [M+H]$^+$ peak at *m/z* 2431.6874 (calcd 2431.6871; 0.3 ppm error) (top) and a simulation (bottom).

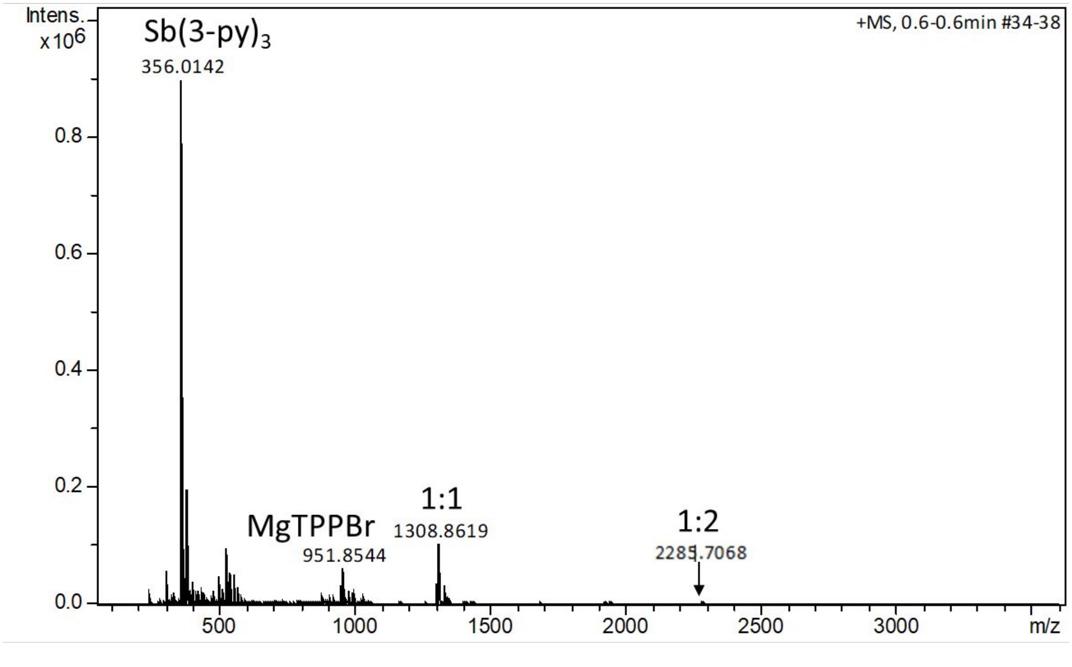

Figure S71. Full HR-MS (ESI-TOF) of {[Sb(3-py)$_3$]$_2$·(MgTPPBr)$_3$} (**1**·MgTPPBr) showing peaks corresponding to Sb(3-py)$_3$ (found: 356.0142), MgTPPBr (found: 951.8544), 1:1 adduct [M+H]$^+$ (found: 1308.8619) and 1:2 adduct [M+H]$^+$ (found: 2285.7068).

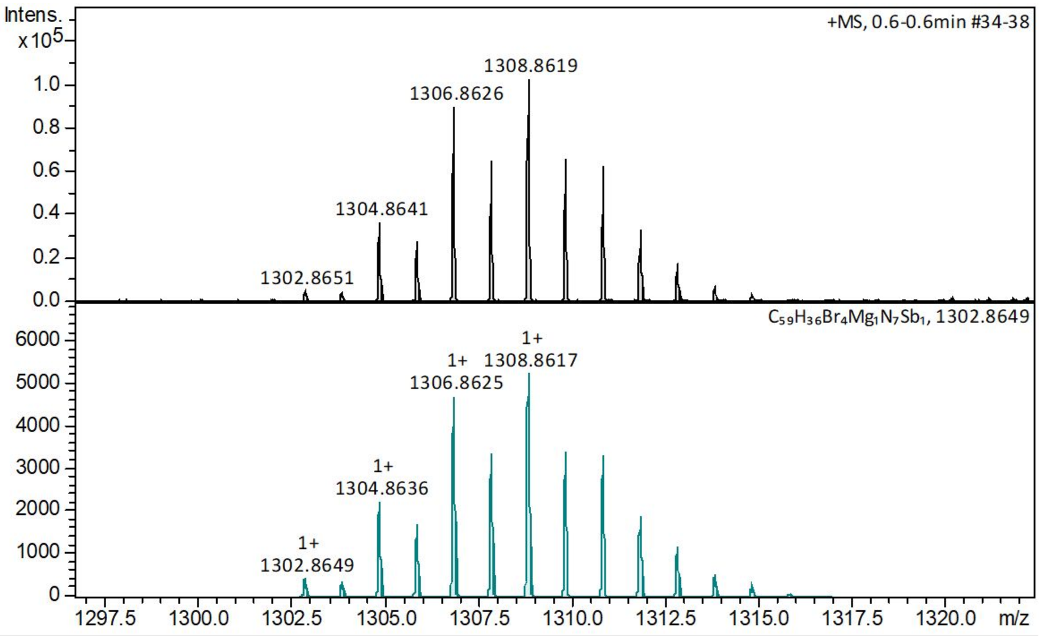

Meas. m/z	m/z	err [ppm]	Ion Formula
1302.8651	1302.8649	-0.2	C59H36Br4MgN7Sb

Figure S72. HR-MS (ESI-TOF) (positive mode) of {[Sb(3-py)$_3$]$_2$·(MgTPPBr)$_3$} (**1**·MgTPPBr) showing the [M+H]$^+$ peak at *m/z* 1302.8651 (calcd 1302.8649; -0.2 ppm error) (top) and a simulation (bottom) corresponding to the 1:1 adduct.

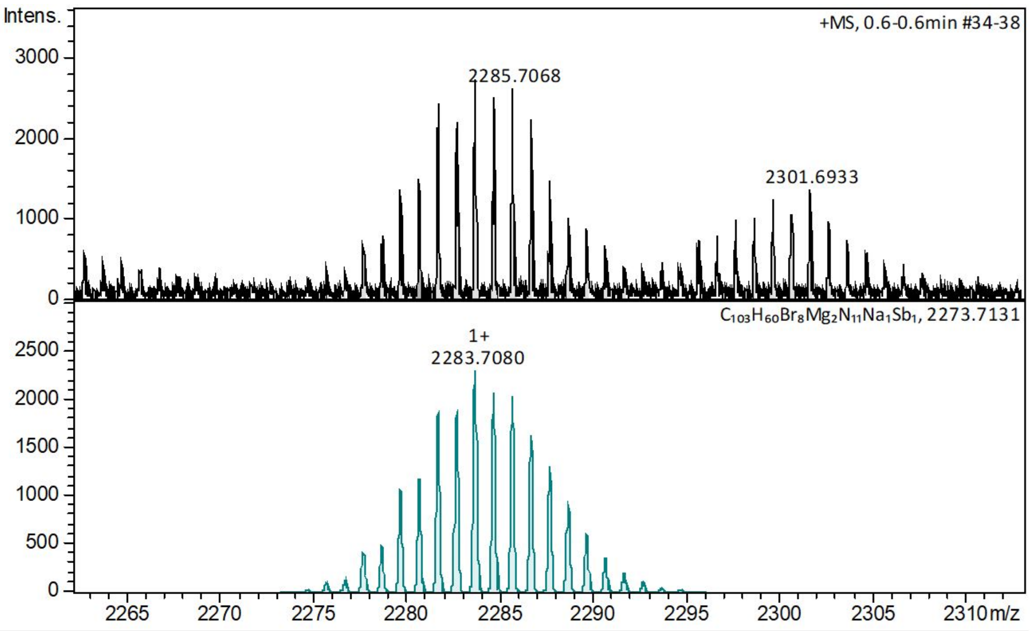

Meas. m/z	m/z	err [ppm]	Ion Formula
2283.7112	2283.708	-0.2	C103H60Br8Mg2N11Sb

Figure S73. HR-MS (ESI-TOF) (positive mode) of {[Sb(3-py)$_3$]$_2$·(MgTPPBr)$_3$} (**1**·MgTPPBr) showing the [M+H]$^+$ peak at *m/z* 2283.7112 (calcd 2283.7080; -0.2 ppm error) (top) and a simulation (bottom) corresponding to the 1:2 adduct.

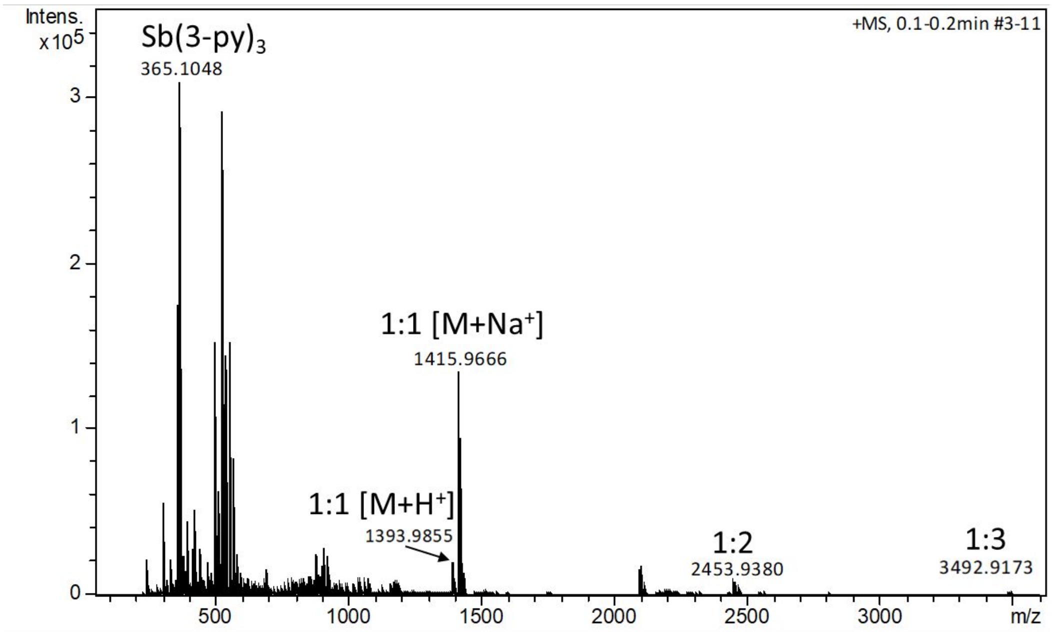

Figure S74. Full HR-MS (ESI-TOF) of {[Sb(3-py)$_3$]·(ZnTPPF$_5$)$_2$} (1·ZnTPPF$_5$) showing peaks corresponding to Sb(3-py)$_3$ (found: 365.1048), 1:1 adduct [M+H]$^+$ (found: 1393.9855), 1:1 adduct [M+Na]$^+$ (found: 1415.9666), 1:2 adduct [M+H]$^+$ (found: 2453.9380) and 1:3 [M+H]$^+$ adduct (found: 3492.9173).

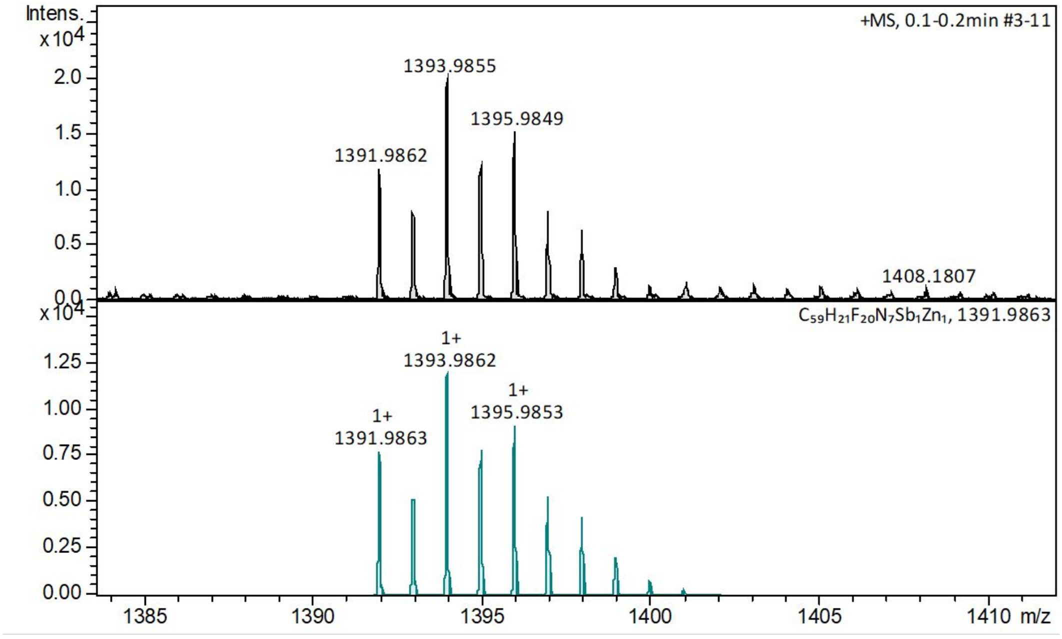

Meas. m/z	m/z	err [ppm]	Ion Formula
1391.9862	1391.9863	0.1	C59H21F20N7SbZn

Figure S75. HR-MS (ESI-TOF) (positive mode) of {[Sb(3-py)$_3$]·(ZnTPPF$_5$)$_3$} (**1**·ZnTPPF$_5$) showing the expected [M+Na]$^+$ peak at *m/z* 1391.9862 (calcd 1391.9863; 0.1 ppm error) (top) and a simulation (bottom) corresponding to the 1:1 adduct.

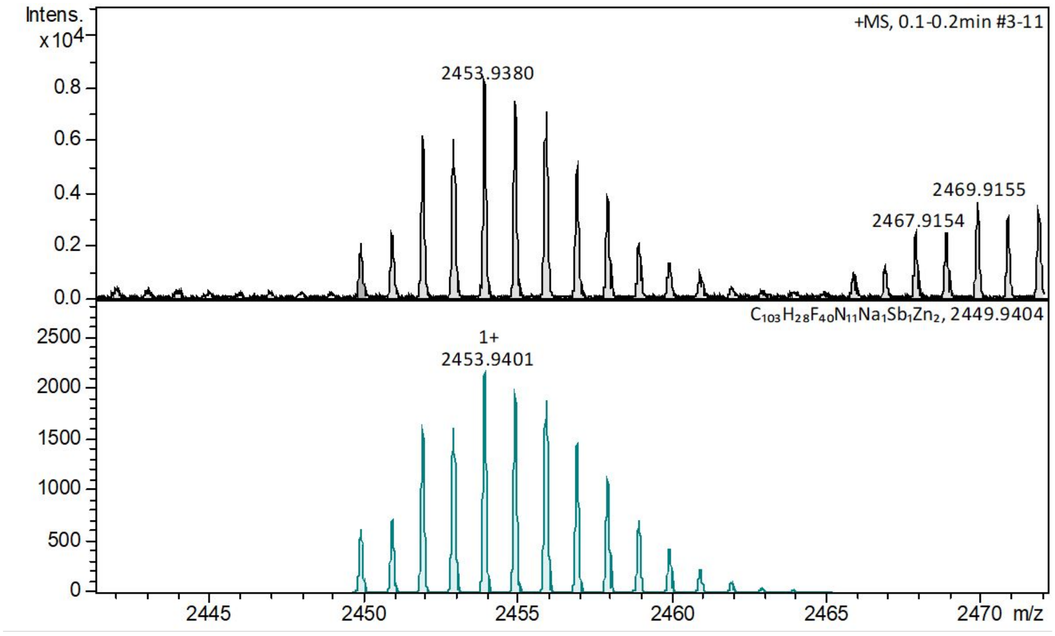

Meas. m/z	m/z	err [ppm]	Ion Formula
2449.9423	2449.9404	-0.8	C103H28F40N11NaSbZn2

Figure S76. HR-MS (ESI-TOF) (positive mode) of {[Sb(3-py)$_3$]·(ZnTPPF$_5$)$_3$} (**1**·ZnTPPF$_5$) showing the expected [M+Na]$^+$ peak at *m/z* 2449.9423 (calcd 2449.9404; -0.8 ppm error) (top) and a simulation (bottom) corresponding to the 1:2 adduct.

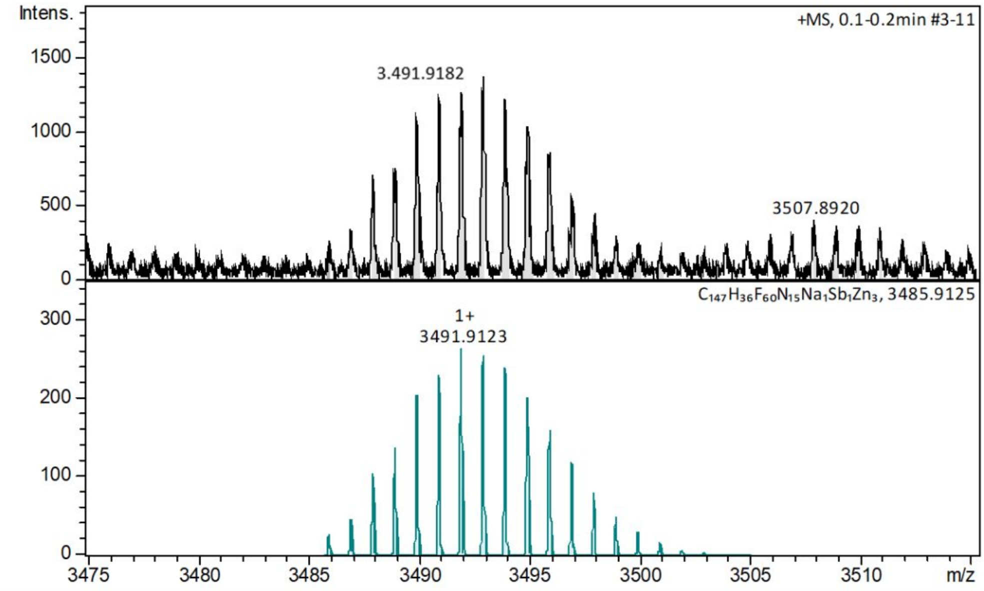

Meas. m/z	m/z	err [ppm]	Ion Formula
3.491.9182	3491.9123	5.9	C147H36F60N15NaSbZn3

Figure S77. HR-MS (ESI-TOF) (positive mode) of {[Sb(3-py)$_3$]·(ZnTPPF$_5$)$_3$} (**1**·ZnTPPF$_5$) showing the expected [M+Na]$^+$ peak at *m/z* 3491.9182 (calcd 3491.9123; 5.9 ppm error) (top) and a simulation (bottom).

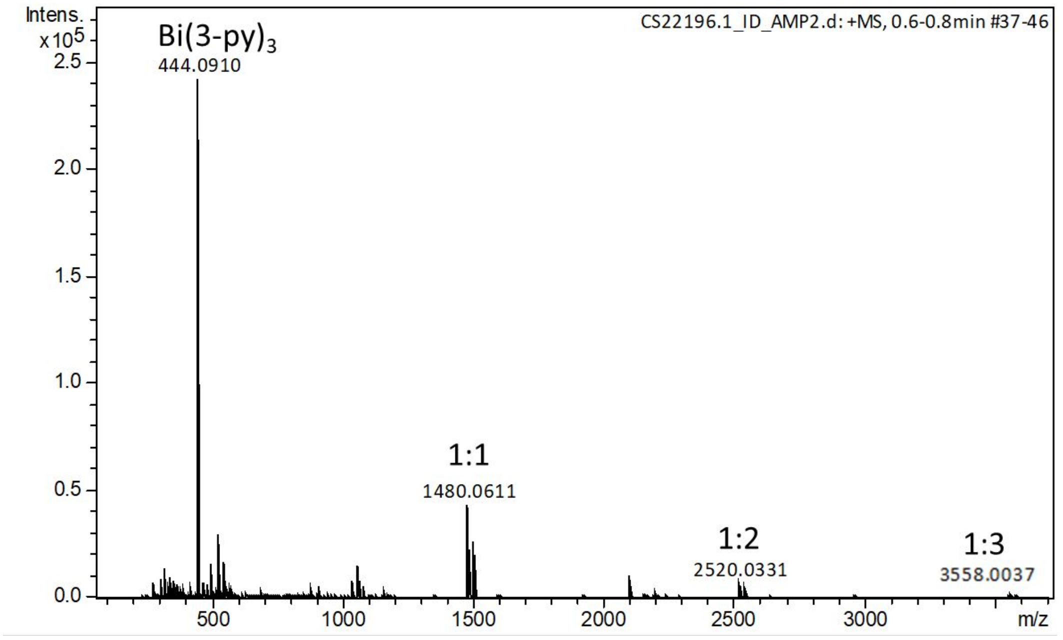

Figure S78. Full HR-MS (ESI-TOF) of {[Bi(3-py)$_3$]·(ZnTPPF$_5$)$_2$} (2·ZnTPPF$_5$) showing peaks corresponding to the Bi(3-py)$_3$ (found: 444.0910), 1:1 adduct [M+H]$^+$ (found: 1480.0611), 1:2 adduct [M+H]$^+$ (found: 2520.0331) and 1:3 adduct [M+H]$^+$ (found: 3558.0037).

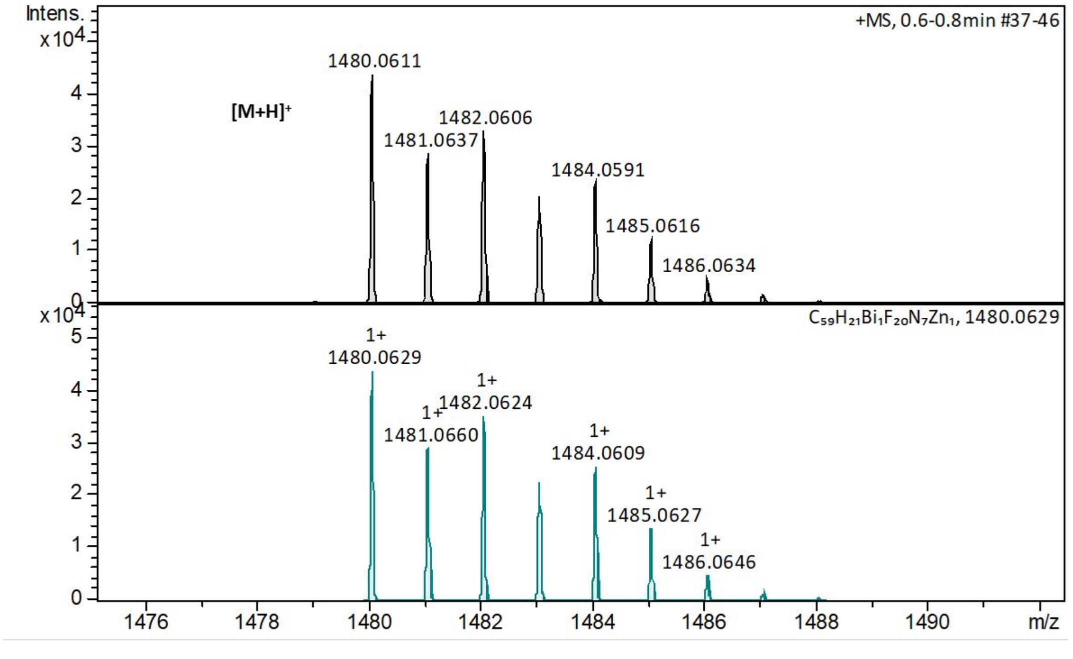

Meas. m/z	m/z	err [ppm]	Ion Formula
1480.0611	1480.0629	-1.2	C59H21BiF20N7Zn

Figure S79. HR-MS (ESI-TOF) (positive mode) of {[Bi(3-py)$_3$]·(ZnTPPF$_5$)$_3$} (**2**·ZnTPPF$_5$) showing the expected [M+H]$^+$ peak at *m/z* 1480.0611 (calcd 1480.0629; -1.2 ppm error) (top) and a simulation (bottom) corresponding to the 1:1 adduct.

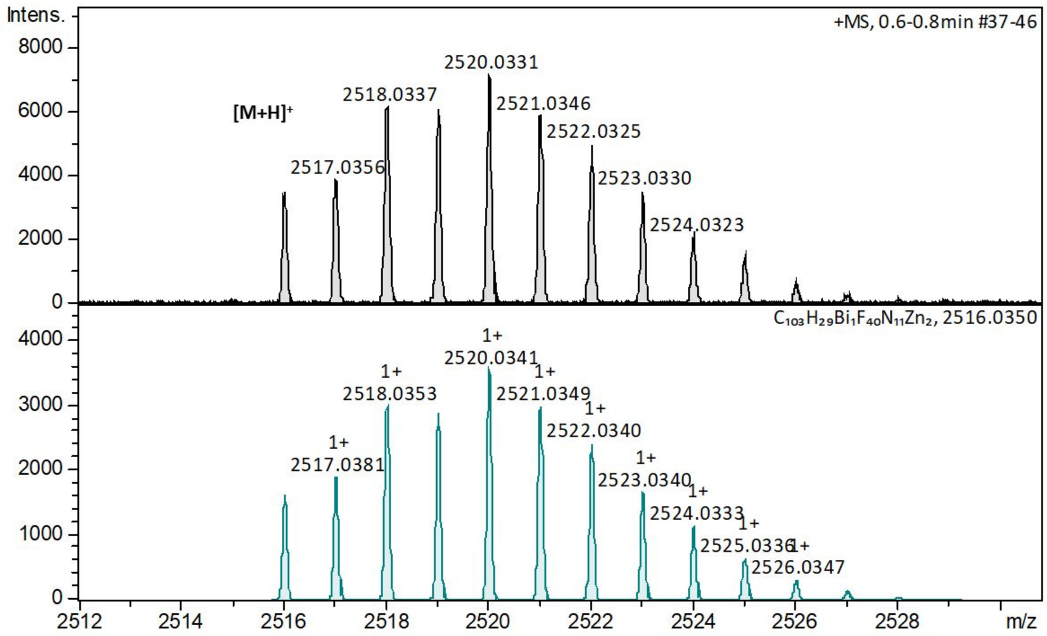

Meas. m/z	m/z	err [ppm]	Ion Formula
2520.0331	2520.0341	-1.8	C103H29BiF40N11Zn2

Figure S80. HR-MS (ESI-TOF) (positive mode) of {[Bi(3-py)$_3$]·(ZnTPPF$_5$)$_3$} (**2**·ZnTPPF$_5$) showing the expected [M+H]$^+$ peak at *m/z* 2520.0331 (calcd 2520.0341; -1.8 ppm error) (top) and a simulation (bottom) corresponding to the 1:2 adduct.

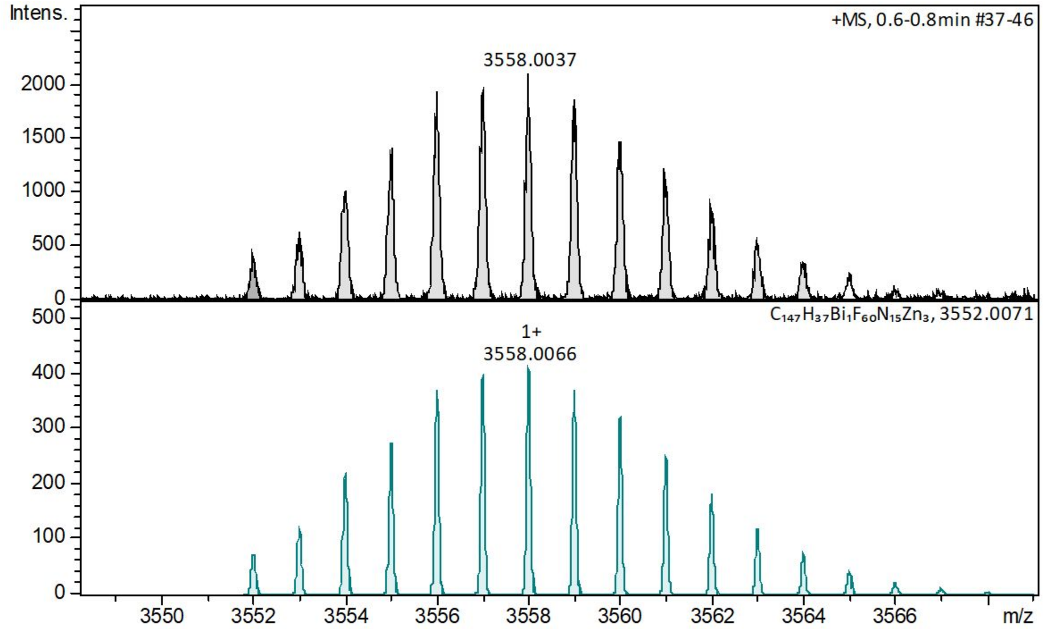

Meas. m/z	m/z	err [ppm]	Ion Formula
3558.0037	3558.0066	-2.9	C147H37BiF60N15Zn3

Figure S81. HR-MS (ESI-TOF) (positive mode) of {[Bi(3-py)$_3$]·(ZnTPPF$_5$)$_3$} (**2**·ZnTPPF$_5$) showing the expected [M+H]$^+$ peak at *m/z* 3558.0037 (calcd 3558.0066; -2.9 ppm error) (top) and a simulation (bottom).

Computational details

Effect of Remote Coordination on Pnictogen Bonding

In order to assess the impact of porphyrin coordination on the corresponding pnictogen sigma hole, the geometry of the parent adduct E(3-py)$_3$·(ZnTPPF$_5$)$_3$ (E = Bi, Sb) from the X-ray diffraction structure was simplified by replacing all perfluorinated aryl groups with hydrogen atoms without further optimization (ZnP, Figure S82). A single porphyrin guest was removed after every single-point calculation to finally reach the starting host E(3-py)$_3$ (E = Sb (**1**), Bi (**2**)). All surface extrema were also evaluated with Multiwfn 3.8,[20] and key local maxima located on the extension of the E-C$_{py}$ bond were detected as sigma holes after iterative inspection in VMD 1.9.2 [21] (see below). Additionally, to further assess the impact of E···F pnictogen bond formation, the same protocol was applied to the assembly E(3-py)$_3$·(ZnTPPF$_5$)$_1$ (i.e. with one coordinated porphyrin) and two simplified structures in which either all the fluorine atoms or only those at the ortho position were replaced with hydrogen atoms (ZnTPPH and ZnTPPH$_2$F$_3$, respectively, Figure S82). This would prevent pnictogen bonding with the bridgehead atom and, therefore, isolate the effect of coordination on charge depletion. Electrostatic potential (V$_{max}$) at those regions were therefore quantified in all cases (see below).

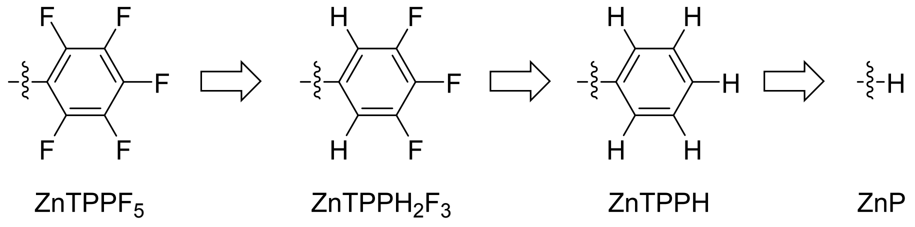

Figure S82. Simplification of the aryl substituents in the coordinated porphyrins for comparison with the E(3-py)$_3$·(ZnTPPF$_5$)$_3$ (E = Bi, Sb) systems using theoretical methods.

Optimized structures

Coordinates of assemblies:

1·ZnTPPF$_5$

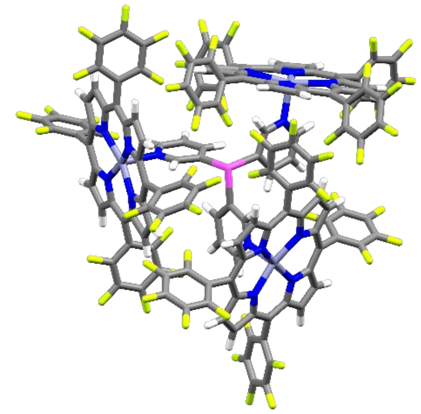

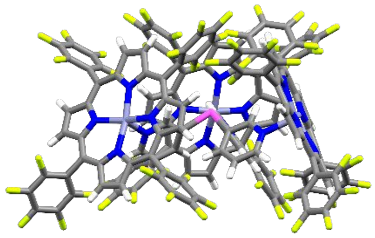

Sb	19.32191 19.32236 19.32212	Zn	16.81617 17.38766 24.2901	Zn	24.28971 16.81584 17.3881
Zn	17.38832 24.29022 16.81562	F	12.13674 18.3127 20.94596	F	20.94508 12.1366 18.31287
F	18.31261 20.94554 12.13649	F	11.08645 18.63558 18.47452	F	18.47352 11.08646 18.63536
F	18.63485 18.47401 11.08623	F	12.68124 18.50627 16.29921	F	16.29835 12.68142 18.50603
F	18.50547 16.29877 12.68111	F	15.32411 17.98737 16.57039	F	16.56973 15.32426 17.98723
F	17.98699 16.57011 15.32403	F	16.40621 17.7997 19.04273	F	19.04217 16.40623 17.79976
F	17.79983 19.0425 16.4061	F	16.09362 11.60224 24.53676	F	24.53666 16.09343 11.60281
F	11.60287 24.53667 16.09384	F	16.96537 9.18502 23.73579	F	23.73592 16.96527 9.18554
F	9.18573 23.7357 16.96584	F	18.94746 8.99383 21.89869	F	21.89894 18.94746 8.99424
F	8.99475 21.89887 18.9482	F	20.05432 11.24821 20.85278	F	20.85291 20.05433 11.24853
F	11.24922 20.85327 20.0551	F	19.10937 13.66004 21.56836	F	21.5683 19.10938 13.66042
F	13.66099 21.5689 19.11001	F	17.15609 15.40581 29.67453	F	29.67427 17.15489 15.40692
F	15.40691 29.67477 17.15435	F	18.56196 15.06461 31.94815	F	31.94814 18.56036 15.06578
F	15.06586 31.94875 18.55967	F	21.06092 16.08893 32.16281	F	32.16311 21.05941 16.08981
F	16.09012 32.16394 21.05861	F	22.15691 17.45681 30.09451	F	30.09484 22.15589 17.45735
F	17.45779 30.09579 22.15514	F	20.75723 17.8025 27.81703	F	27.81711 20.75662 17.80299
F	17.80335 27.81795 20.756	F	18.0561 22.39795 23.97716	F	23.97786 18.05826 22.39746
F	22.39825 23.97735 18.05575	F	17.99701 25.07141 24.27224	F	24.27219 17.99999 25.07104
F	25.07175 24.27219 17.99689	F	16.11446 26.21604 25.86377	F	25.86125 16.11612 26.21688
F	26.21674 25.86295 16.11392	F	14.2889 24.67092 27.13746	F	27.13325 14.28843 24.6729
F	24.67195 27.13615 14.28774	F	14.32734 21.99568 26.81904	F	26.81553 14.32605 21.99755
F	21.99667 26.818 14.32599	N	18.66948 17.97152 23.38093	N	23.38072 18.66931 17.97174
N	17.97194 23.38133 18.66915	N	17.03907 18.50751 26.01828	N	26.01772 17.03871 18.5082
N	18.50824 26.01836 17.03805	N	15.67773 18.92345 23.46627	N	23.46556 15.67755 18.92387
N	18.92391 23.46604 15.6772	N	16.107 16.15163 22.77792	N	22.77747 16.10685 16.15205
N	16.15216 22.7779 16.1069	N	17.44469 15.71513 25.33574	N	25.33551 17.44423 15.71571
N	15.71588 25.33598 17.44415	C	18.60632 18.3404 22.10164	C	22.10141 18.60637 18.34054
C	18.34085 22.10205 18.60613	H	17.62206 18.24862 21.63057	H	21.63028 17.6221 18.24908
H	18.24976 21.63107 17.62176	C	19.7058 18.81575 21.387	C	21.38681 19.70607 18.81549
C	18.81544 21.3873 19.70588	C	20.93398 18.86156 22.05263	C	22.05253 20.9342 18.86093
C	18.86047 22.05283 20.93413	H	21.83794 19.19854 21.53843	H	21.53838 21.83831 19.19759
H	19.19684 21.53856 21.83827	C	21.00157 18.464 23.38831	C	23.38825 21.00157 18.46344
C	18.46292 23.38854 21.00156	H	21.94629 18.48548 23.93349	H	23.93347 21.94627 18.48457
H	18.48378 23.93362 21.94635	C	19.83908 18.0338 24.02066	C	24.02054 19.83889 18.03367
C	18.03347 24.02097 19.83884	H	19.84074 17.72905 25.07174	H	25.07164 19.84036 17.729
H	17.72869 25.07204 19.84039	C	15.66641 20.20359 23.92472	C	23.92379 15.66643 20.20409
C	20.20409 23.92441 15.66572	C	14.99635 21.06095 22.96962	C	22.96851 14.99658 21.06141
C	21.06133 22.96917 14.99571	H	14.82655 22.12964 23.08229	H	23.081 14.82701 22.13016
H	22.13001 23.08177 14.82581	C	14.61295 20.26662 21.93318	C	21.93221 14.61304 20.26696
C	20.26689 21.93276 14.61244	H	14.07482 20.56138 21.0376	H	21.03658 14.07496 20.56164
H	20.56155 21.03713 14.07435	C	15.05622 18.92728 22.25499	C	22.25424 15.05611 18.92761
C	18.92762 22.25469 15.0558	C	14.918 17.81506 21.40614	C	21.4055 14.91787 17.81531
C	17.81533 21.4059 14.91779	C	14.30223 18.03101 20.06888	C	20.06818 14.30217 18.03113
C	18.03107 20.06858 14.30205	C	12.93543 18.25812 19.89718	C	19.89635 12.93537 18.25819
C	18.25798 19.89679 12.93523	C	12.38128 18.41851 18.62776	C	18.62688 12.38129 18.41841
C	18.41806 18.62732 12.38109	C	13.20065 18.34795 17.50446	C	17.50364 13.20075 18.3478
C	18.34743 17.50406 13.20051	C	14.56345 18.10025 17.6448	C	17.6441 14.56355 18.10016
C	18.09995 17.64449 14.56333	C	15.09943 17.9816 18.92228	C	18.92163 15.09944 17.98164
C	17.98157 18.92201 15.09929	C	15.39841 16.51988 21.67582	C	21.67533 15.39827 16.52015
C	16.52022 21.67571 15.39835	C	15.24452 15.38398 20.79154	C	20.79118 15.2444 15.38415
C	15.38424 20.79151 15.24469	H	14.70489 15.3767 19.8482	H	19.84783 14.70478 15.37677
H	15.37681 19.84814 14.7051	C	15.89691 14.34262 21.37662	C	21.37638 15.89679 14.34287
C	14.34303 21.37668 15.89723	H	15.98269 13.32421 21.00414	H	21.00402 15.98258 13.32442
H	13.32461 21.00427 15.9832	C	16.43392 14.84086 22.62446	C	22.62417 16.43379 14.84126
C	14.84144 22.62453 16.43405	C	17.17189 14.06522 23.53339	C	23.5332 17.17173 14.06571
C	14.06594 23.53356 17.17204	C	17.57914 12.70855 23.08376	C	23.08368 17.57905 12.70903
C	12.70933 23.084 17.57955	C	17.04126 11.53755 23.61884	C	23.6188 17.04115 11.53805
C	11.53827 23.61891 17.04165	C	17.4914 10.27971 23.21879	C	23.21888 17.49132 10.28019
C	10.28047 23.21886 17.4919	C	18.50712 10.17812 22.26952	C	22.26968 18.50709 10.17854
C	10.17899 22.26974 18.50776	C	19.06543 11.33313 21.72517	C	21.72527 19.06541 11.33352
C	11.33407 21.72556 19.06611	C	18.57757 12.57382 22.11801	C	22.118 18.57754 12.57425
C	12.57472 22.11841 18.57816	C	17.6223 14.47828 24.79965	C	24.7995 17.62197 14.47885
C	14.47907 24.79992 17.62211	C	18.3633 13.64635 25.72382	C	25.7238 18.36291 13.64701
C	13.64727 25.72426 18.36304	H	18.65294 12.61094 25.55985	H	25.55991 18.65264 12.61161

H	12.6119	25.56034	18.65288	C	18.61944	14.41304	26.81881	C	26.8188	18.61884	14.41376
C	14.41398	26.81933	18.61876	H	19.15752	14.12445	27.71902	H	27.7191	19.15682	14.12523
H	14.12547	27.71968	19.15666	C	18.04442	15.71491	26.5565	C	26.55634	18.04382	15.7156
C	15.71576	26.55687	18.04359	C	18.15027	16.82079	27.41788	C	27.41764	18.14959	16.82155
C	16.82168	27.41825	18.14911	C	18.9083	16.62363	28.68101	C	28.68091	18.9074	16.62444
C	16.62461	28.68158	18.90684	C	18.37089	15.91795	29.75868	C	29.75856	18.36974	15.91892
C	15.91903	29.75917	18.36915	C	19.08919	15.73193	30.93828	C	30.93829	19.08783	15.73293
C	15.73308	30.93896	19.08716	C	20.3742	16.2593	31.0507	C	31.05087	20.37289	16.26016
C	16.26042	31.05165	20.37216	C	20.93597	16.96457	29.98759	C	29.98778	20.93491	16.96525
C	16.96559	29.98861	20.93421	C	20.19937	17.13701	28.81907	C	28.81913	20.19852	17.13767
C	17.13796	28.81991	20.1979	C	17.67051	18.11563	27.15638	C	27.15593	17.67	18.11641
C	18.11647	27.15658	17.66933	C	17.82737	19.24861	28.044	C	28.04338	17.82693	19.24952
C	19.24953	28.04414	17.826	H	18.27532	19.22249	29.03502	H	29.03446	18.27477	19.22348
H	19.2235	29.03523	18.2738	C	17.30467	20.32579	27.39605	C	27.39518	17.30449	20.32668
C	20.32665	27.396	17.30341	H	17.24785	21.35228	27.752	H	27.75094	17.24777	21.35324
H	21.35317	27.75184	17.24652	C	16.81381	19.84345	26.12292	C	26.12209	16.81369	19.84419
C	19.84419	26.12284	16.81276	C	16.20991	20.64661	25.14211	C	25.14109	16.20997	20.64725
C	20.64724	25.14183	16.20902	C	16.18382	22.11474	25.37869	C	25.37733	16.18425	22.11545
C	22.1154	25.37825	16.18294	C	17.12139	22.93502	24.75091	C	24.75042	17.12292	22.93514
C	22.93551	24.75071	17.12083	C	17.1064	24.31775	24.89963	C	24.89878	17.10837	24.31791
C	24.31825	24.89932	17.10597	C	16.13967	24.90737	25.71203	C	25.70989	16.14095	24.90816
C	24.90805	25.71133	16.13903	C	15.20152	24.11033	26.36596	C	26.36295	15.20168	24.11171
C	24.11118	26.36502	15.20055	C	15.23173	22.72675	26.19396	C	26.19134	15.23149	22.72807
C	22.72758	26.19316	15.23066								

2·ZnTPPF$_5$

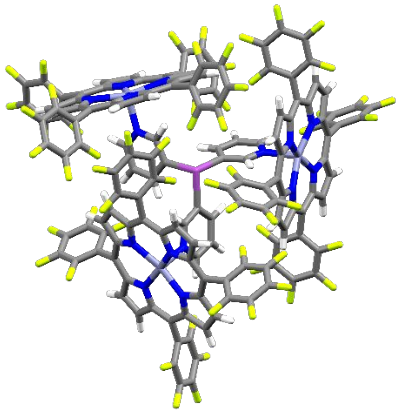

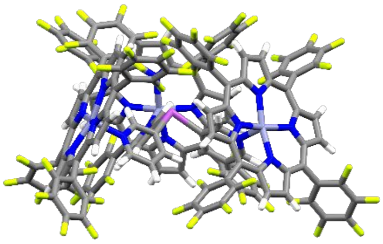

Bi	19.21921	27.34674	3.6959	Zn	16.7708	29.21605	8.79735	Zn	24.33119	29.78267	1.84004
Zn	17.35342	22.24225	1.25188	F	12.07625	28.30244	5.44375	F	20.98816	34.48533	2.75041
F	18.28423	25.58576	-3.44599	N	18.61835	28.63919	7.88296	N	23.41066	27.93715	2.41296
N	17.92568	23.15943	3.09941	C	18.54593	28.27517	6.6014	C	22.12863	28.01284	2.77463
C	18.291	24.44053	3.02554	H	17.54856	28.3347	6.15336	H	21.68369	29.01165	2.71583
H	18.23517	24.88654	2.02703	F	11.04763	27.9364	2.97159	F	18.51796	35.52022	3.11219
F	18.65536	28.05536	-4.47879	N	16.06844	30.45014	7.27683	N	22.81541	30.49006	0.60302
N	16.12276	23.76277	0.54364	C	19.64747	27.8471	5.86308	C	21.38616	26.91282	3.19939
C	18.71591	25.1808	4.12699	F	12.65826	28.02603	0.80599	F	16.34827	33.91549	3.01623
F	18.56212	30.22454	-2.87318	N	15.61381	27.68722	7.98547	N	23.51904	30.94081	3.3678
N	18.88608	23.05059	0.0975	C	20.88711	27.83447	6.50734	C	22.02663	25.67121	3.21113
C	18.72372	24.53914	5.36802	H	21.79362	27.53221	5.97559	H	21.49152	24.76578	3.51072
H	19.02326	25.07259	6.27444	F	15.30762	28.53312	1.09377	F	16.62998	31.26598	2.50679
F	18.04618	29.9429	-0.22487	N	16.97591	28.10953	10.53503	N	26.06597	29.57235	2.95012
N	18.4577	20.50383	1.46291	C	20.96439	28.22025	7.84667	C	23.36648	25.59053	2.82787
C	18.33658	23.2003	5.44679	H	21.91835	28.22363	8.37608	H	23.89298	24.63497	2.8239
H	18.32943	22.67294	6.40187	F	16.36269	28.77394	3.56693	F	19.10079	30.20451	2.26991
F	17.80008	27.47232	0.83481	N	17.41086	30.89317	9.83045	N	25.36638	29.14106	0.16471
N	15.67364	21.21194	1.88941	C	19.80013	28.60992	8.50201	C	24.02611	26.75346	2.44146
C	17.9504	22.54282	4.28257	H	19.81056	28.90877	9.5548	H	25.07946	26.74041	2.14472
H	17.65072	21.49028	4.29414	F	14.31097	24.6632	11.44922	F	26.97716	32.23379	6.40147
F	21.91072	19.58192	-1.19102	C	16.41852	31.75302	7.1084	C	22.64888	30.14123	-0.70045
C	14.8191	23.93303	0.88995	F	14.24677	21.99206	11.79107	F	27.3125	32.2946	9.07354
F	24.58175	19.23768	-1.24707	C	15.89266	32.24587	5.85378	C	21.3968	30.67082	-1.19573
C	14.32873	25.18703	0.3603	H	15.99682	33.2582	5.46983	H	21.0148	30.56833	-2.20897
H	13.31648	25.57208	0.46105	F	15.98313	20.40814	10.44229	F	25.95728	30.5585	10.65222
F	26.1619	20.58801	0.49148	C	15.22345	31.20897	5.27963	C	20.82219	31.34094	-0.15968
C	15.36788	25.75891	-0.30735	H	14.68549	31.21464	4.3352	H	19.87926	31.88151	-0.16709
H	15.36443	26.70222	-0.84728	F	17.80166	21.51048	8.74988	F	24.26498	28.7436	9.54373
F	25.05588	22.28452	2.30397	C	15.35695	30.08099	6.17733	C	21.717	31.20424	0.97023
C	16.49475	24.86054	-0.16903	F	17.88647	24.17935	8.42776	F	23.94937	28.66214	6.87395
F	22.38706	22.60905	2.38069	C	14.86163	28.789	5.91931	C	21.45735	31.69928	2.262
C	17.78832	25.11649	-0.66131	F	17.10159	31.23702	14.16459	F	29.70193	29.44027	-0.16975
F	15.32645	16.87767	1.58572	C	14.25011	28.56417	4.58181	C	20.12094	32.31409	2.48437
C	18.01601	26.45257	-1.27484	F	18.4987	31.58766	16.44231	F	31.97709	28.038	-0.51648
F	14.96957	14.60246	2.98536	C	12.8842	28.33855	4.40191	C	19.94422	33.68024	2.71113
C	18.24625	26.62936	-2.64041	F	20.99029	30.54985	16.67698	F	32.20366	25.54519	0.5202
F	16.00011	14.37063	5.48023	C	12.34116	28.15524	3.13047	C	18.67386	34.22651	2.89227
C	18.43224	27.89947	-3.18562	F	22.08765	29.15897	14.62493	F	30.14608	24.45176	1.90597
F	17.38995	16.42297	6.57835	C	13.16909	28.20368	2.01206	C	17.55333	33.40164	2.84057
C	18.38192	29.01973	-2.3603	F	20.69639	28.80367	12.34349	F	27.86716	25.84819	2.25733
F	17.75148	18.7019	5.18455	C	14.53196	28.44762	2.15994	C	17.69808	32.03866	2.59538

C	18.1334	28.87498	-0.99793	F	19.14124	32.88024	6.06437	F	21.60034	27.42157	-1.83149
F	13.68565	24.98311	3.60804	C	15.05363	28.59231	3.43998	C	18.97701	31.51368	2.45284
C	17.98606	27.59619	-0.47393	F	20.1167	35.27171	5.32235	F	20.86065	26.44975	-4.22515
F	11.29225	25.72913	4.5757	C	14.99001	27.68116	6.77543	C	22.31064	31.56779	3.37166
C	18.89499	24.25955	-0.52836	F	19.01794	37.55228	6.31549	F	21.86111	27.54769	-6.50293
F	9.01371	24.73635	3.47245	C	14.53348	26.3438	6.46319	C	21.99662	32.02406	4.70868
C	20.23392	24.56976	-0.98169	H	13.99289	26.04668	5.56966	H	21.10391	32.56685	5.00424
H	20.53343	25.46205	-1.52299	F	17.01079	37.41033	8.1306	F	23.68102	29.55011	-6.35588
F	9.15933	22.91753	1.46892	C	14.90985	25.55346	7.50504	C	23.03566	31.64422	5.50107
C	21.02223	23.52782	-0.60133	H	14.72831	24.48765	7.6257	H	23.15436	31.82449	6.56732
H	22.08843	23.40585	-0.77971	F	16.10647	35.01463	8.95965	F	24.50754	30.45059	-3.95784
F	11.55667	22.08453	0.5726	C	15.59062	26.41061	8.45297	C	23.98372	30.96168	4.64547
C	20.16231	22.58186	0.07868	C	16.13134	25.97051	9.67302	C	25.20137	30.41745	5.08785
C	20.59981	21.36236	0.62272	C	16.0918	24.50461	9.91924	C	25.44427	30.45498	6.55438
C	22.06559	21.11476	0.58767	C	15.17188	23.91338	10.78561	C	26.31047	31.37302	7.14872
C	22.65867	20.24634	-0.3291	C	15.12881	22.53198	10.97071	C	26.4922	31.41433	8.53062
C	24.04002	20.05998	-0.36795	C	16.02085	21.71504	10.27799	C	25.79617	30.52242	9.34487
C	24.85503	20.7535	0.52524	C	16.95446	22.28286	9.41308	C	24.93136	29.59063	8.7739
C	24.28533	21.62049	1.45577	C	16.98336	23.66399	9.25179	C	24.77346	29.56346	7.39235
C	22.90426	21.783	1.48048	C	16.74118	26.77607	10.6489	C	26.17754	29.80587	4.28397
C	19.79167	20.38826	1.23202	C	17.22865	26.29951	11.92577	C	27.45228	29.31522	4.76291
C	20.26564	19.11172	1.72284	H	17.16709	25.27598	12.289	H	27.81348	29.37532	5.78726
H	21.28899	18.74739	1.66468	C	17.75694	27.37781	12.56686	C	28.09449	28.78625	3.6856
C	19.18527	18.47252	2.24919	H	18.20444	27.40771	13.55795	H	29.08464	28.33657	3.65748
H	19.15316	17.48218	2.69817	C	17.60867	28.50551	11.671	C	27.20135	28.9373	2.55611
C	18.05886	19.36926	2.09642	C	18.0973	29.79858	11.92434	C	27.45628	28.44889	1.26326
C	16.76425	19.11802	2.58203	C	18.85094	30.00039	13.18943	C	28.72002	27.6924	1.06364
C	16.55911	17.85429	3.33706	C	20.13822	29.48033	13.33747	C	28.86391	26.40444	1.58317
C	17.07539	17.70773	4.62602	C	20.87025	29.65768	14.50819	C	30.03327	25.66977	1.40783
C	16.8948	16.53833	5.35935	C	20.30779	30.37477	15.56297	C	31.09089	26.23021	0.69335
C	16.17823	15.4834	4.7965	C	19.02656	30.90903	15.44029	C	30.97238	27.51206	0.15967
C	15.64771	15.60462	3.51359	C	18.31277	30.718	14.25878	C	29.79216	28.22853	0.34866
C	15.84195	16.78483	2.79851	C	18.00543	30.89833	11.05341	C	26.58789	28.54352	0.16173
C	15.66565	19.98995	2.48594	C	18.59509	32.19584	11.30541	C	26.84121	27.95411	-1.13564
C	14.36636	19.74026	3.07264	H	19.13216	32.48702	12.20538	H	27.74048	27.41504	-1.42526
H	14.07285	18.84146	3.61041	C	18.35464	32.9539	10.20082	C	25.73881	28.19777	-1.89587
C	13.61009	20.84526	2.82844	H	18.6579	33.98384	10.02717	H	25.56658	27.89559	-2.92638
H	12.57955	21.0205	3.12878	C	17.6075	32.12152	9.28199	C	24.82006	28.94661	-1.06494
C	14.44535	21.76198	2.08196	C	17.1684	32.52642	8.00924	C	23.54936	29.38935	-1.47231
C	14.04289	23.03436	1.63952	C	17.59924	33.87073	7.5442	C	23.08605	28.96055	-2.81789
C	12.6979	23.50149	2.06598	C	18.61185	33.9794	6.58982	C	22.12935	27.95043	-2.9292
C	12.58743	24.45778	3.07659	C	19.11581	35.20952	6.18389	C	21.72462	27.44841	-4.16051
C	11.35633	24.86576	3.57649	C	18.56163	36.37884	6.70087	C	22.24531	28.00214	-5.32838
C	10.18801	24.34899	3.02003	C	17.53307	36.30251	7.63848	C	23.18538	29.02826	-5.24942
C	10.26621	23.40947	1.99336	C	17.06631	35.05552	8.05323	C	23.5988	29.49306	-4.00125
C	11.51406	22.99269	1.53072								

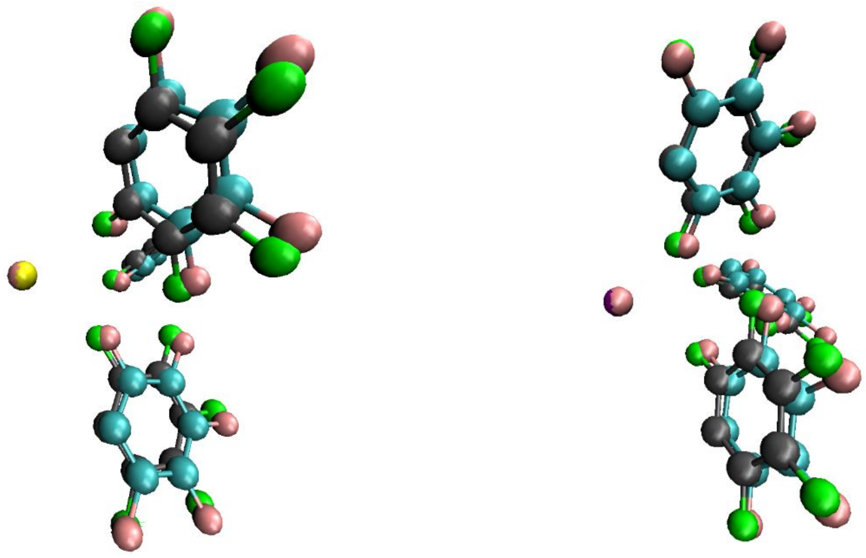

Figure S83. Superimposed structures of the optimized and solid-state geometries of **1·ZnTPPF$_5$** (left) and **2·ZnTPPF$_5$** (right) for comparison of their E–F distances. All other fragments have been omitted for clarity. The fluorine atoms of the computed structures (shown in green) are clearly closer to the pnictogen atom than the fluorine atoms of the X-ray structure (shown in pink). For quantitative distances, see below.

NBO analysis

Table S12. Attractive energies (kcal/mol) and distances (Å) of the interactions present in the prepared capsules in the solid state and in their optimized structures

Compound	Interaction type	Non-optimized Distance (Å)	Non-optimized Energy (kcal/mol)	Optimized Distance (Å)	Optimized Energy (kcal/mol)
**1**·ZnTPPF$_5$	E–F	3.47	3.09	3.30	6.09
	F–H$_β$	2.53	2.13	2.64	0.75
	F–H$_{py}$	2.50	0.93	2.63	2.37
**2**·ZnTPPF$_5$	E–F	3.36	6.75	3.19	11.22
	F–H$_β$	2.54	2.01	2.56	1.83
	F–H$_{py}$	2.57	1.11	2.67	2.07

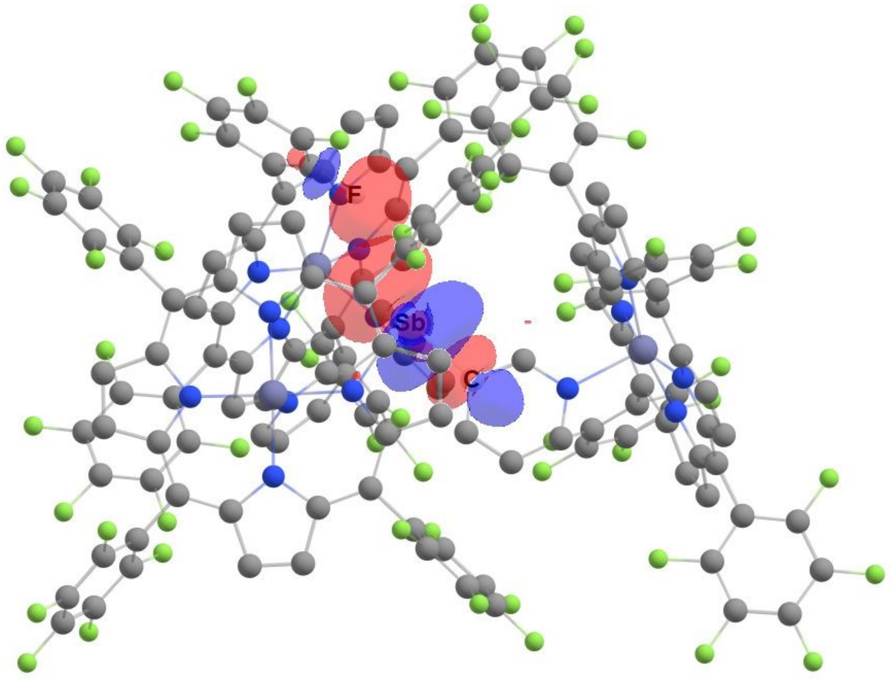

Figure S84. Representation of the NBO molecular orbitals for the interaction between fluorine and antimony in **1**·ZnTPPF$_5$.

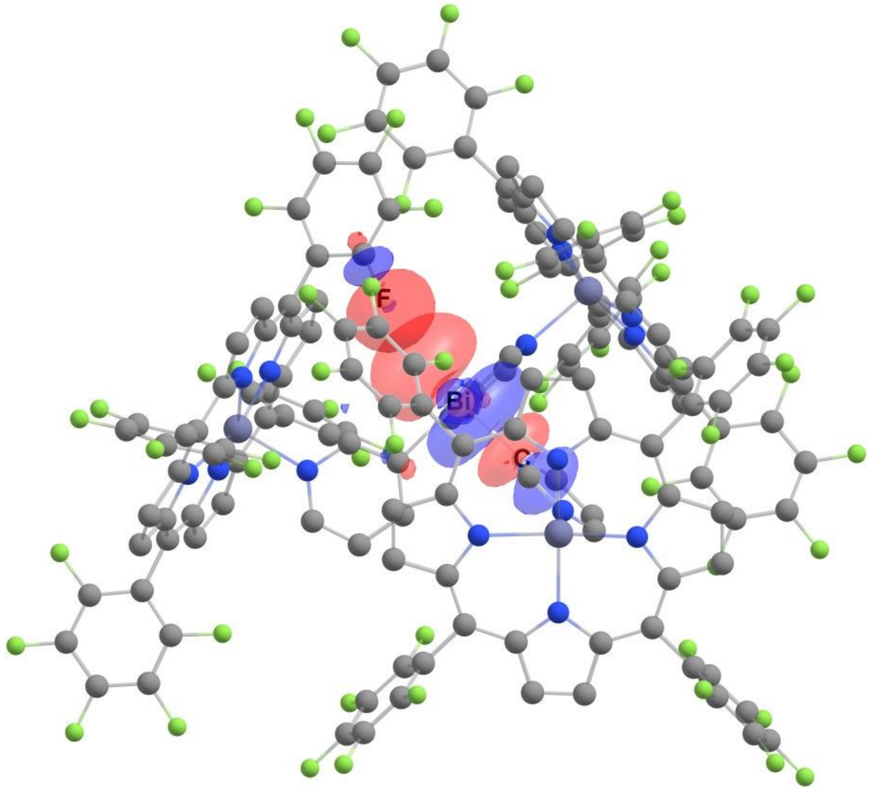

Figure S85. Representation of the NBO molecular orbitals for the interaction between fluorine and bismuth in **2**·ZnTPPF$_5$.

Topology analysis

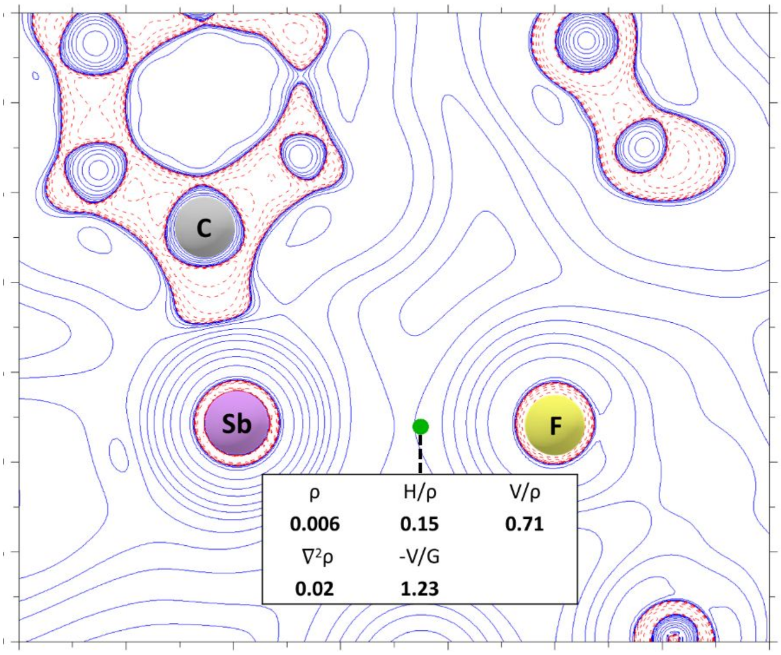

Figure S86. 2D Quantum Theory of Atoms in Molecules (QTAIM) analysis of **1**·$ZnTPPF_5$. Contour lines show the Laplacian of the electron density ($\nabla^2\rho$) (blue: $\nabla^2\rho > 0$; red: $\nabla^2\rho < 0$). Atomic critical points (3, +3) are displayed as atom labels. Bond critical points (3, −1) are displayed as green dots. The values of the electron density (ρ), the Laplacian of the electron density ($\nabla^2\rho$), the relative energy density (*H*/ρ), the relative kinetic energy density (*V*/ρ) and the ratio of kinetic and potential energy densities (−*V*/*G*) at the bond critical point are given in atomic units (a.u.).

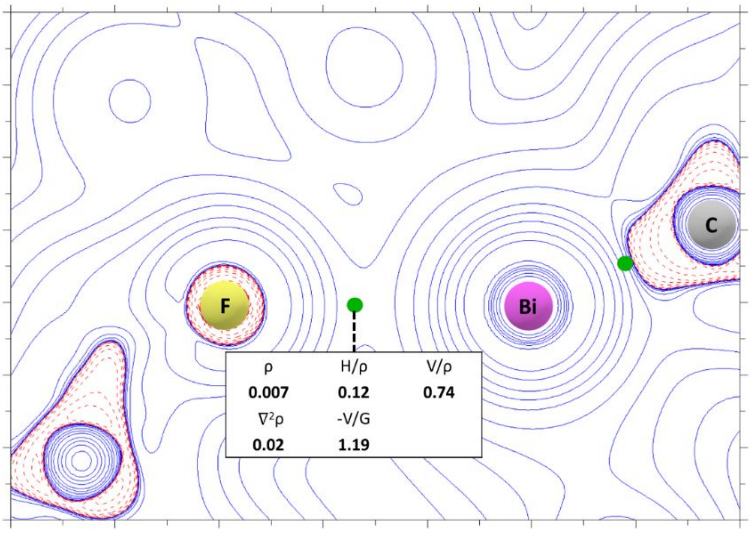

Figure S87. 2D Quantum Theory of Atoms in Molecules (QTAIM) analysis of **2**·$ZnTPPF_5$. Contour lines show the Laplacian of the electron density ($\nabla^2\rho$) (blue: $\nabla^2\rho > 0$; red: $\nabla^2\rho < 0$). Atomic critical points (3, +3) are displayed as atom labels. Bond critical points (3, −1) are displayed as green dots. The values of the electron density (ρ), the Laplacian of the electron density ($\nabla^2\rho$), the relative energy density (H/ρ), the relative kinetic energy density (V/ρ) and the ratio of kinetic and potential energy densities (−V/G) at the bond critical point are given in atomic units (a.u.).

ESP maps

The ESP maps of free ligands **1** and **2** show three regions of high electrostatic potential (sigma holes) at the bridgehead (V_{max} 19.68 and 22.88 kcal mol^{-1}, respectively), which are located along the axes of the E–C_{py} covalent bonds.

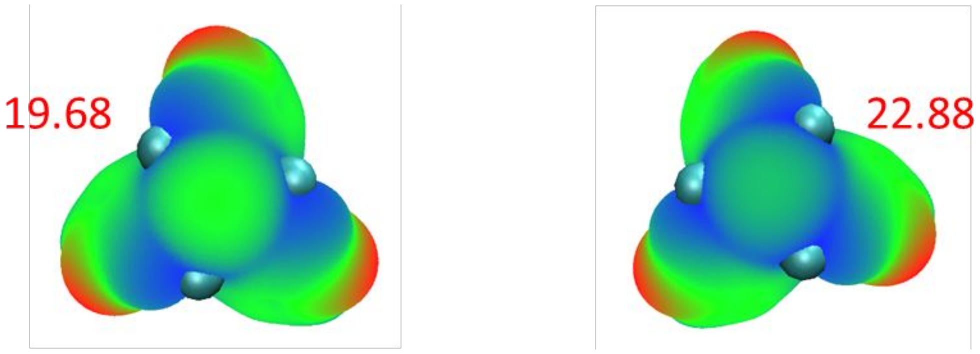

Figure S88. ESP representation of Sb(3-py)$_3$ (left) and Bi(3-py)$_3$ (right), with the regions of high electrostatic potential indicated with cyan spheres. V_{max} values are indicated in red (kcal/mol).

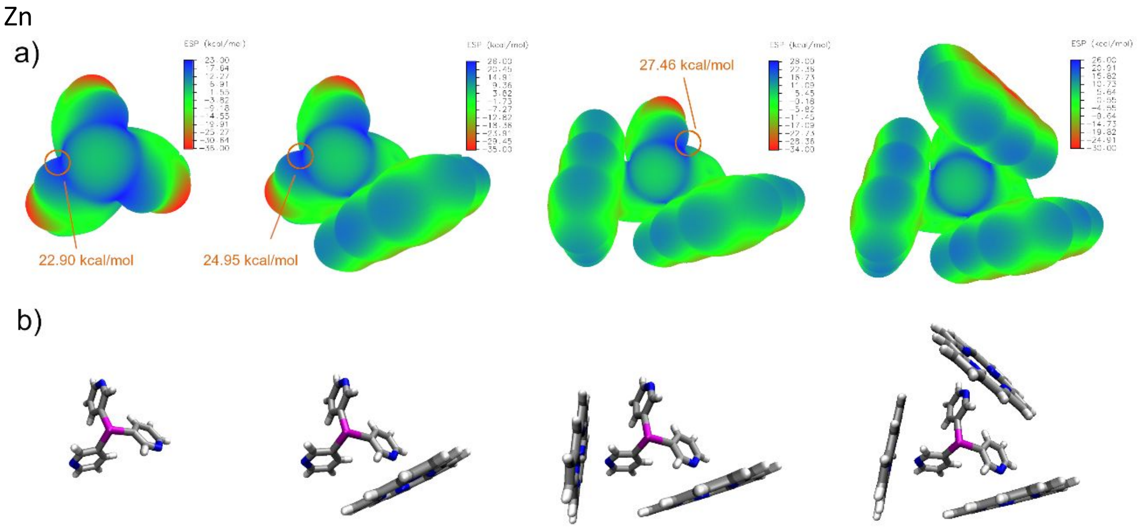

Figure S89. a) ESP maps of compound Bi(3-py)$_3$ (**2**) and its corresponding mono-, bis- and tris-adducts with porphyrin ZnP, along with their color scale bars (in kcal/mol). V_{max} values are shown. b) Molecular representations of the above-described species.

The electrostatic potential (ESP) of the linkers **1** and **2** upon coordination of 1, 2 or 3 simple ZnP units (no substitution on the meso positions of the porphyrin) was examined (Figure S90). The V_{max} values of the sigma holes were plotted against the number of coordinated porphyrins, which clearly revealed increasing charge depletion at these points (i.e., more positive values) with the coordination of additional porphyrins. Linear fitting of the plot yielded R^2 values of 0.999 and 0.991 for $Sb(3\text{-}py)_3$ and $Bi(3\text{-}py)_3$, respectively, and the slope indicated a charge depletion of 2.21 and 2.29 kcal/mol per coordinated porphyrin unit showing sigma hole deepening.

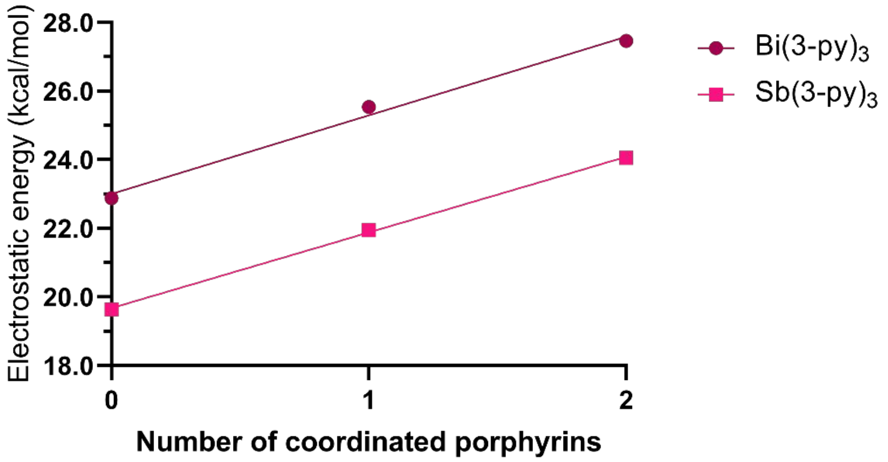

Figure S90. Plot of V_{max} of the sigma holes for compounds $Sb(3\text{-}py)_3$ and $Bi(3\text{-}py)_3$ (**1** and **2**) as a function of the number of coordinated porphyrin units ZnP.

However, due to the proximity of the π-electron density of the coordinated porphyrin, the changes in the nearby local maxima were damped, precluding the analysis when the number of coordinated porphyrins was 3. In other words, the increase in charge depletion (V_{max}) was less pronounced than expected in sigma hole regions near the π-electron density of the porphyrin.

Interestingly, the V_{max} values of these sigma holes in close proximity to a coordinated porphyrin also showed a linear correlation with the number of coordinated porphyrins (Figure S91), with similar charge depletion of 2.14 and 2.26 kcal/mol per coordinated porphyrin unit for $Sb(3\text{-}py)_3$ and $Bi(3\text{-}py)_3$, respectively (R^2 = 1.000 and 0.995). In summary, the coordination of each porphyrin ZnP increases the V_{max} of the pnictogen sigma hole by ca. 2.2 kcal/mol.

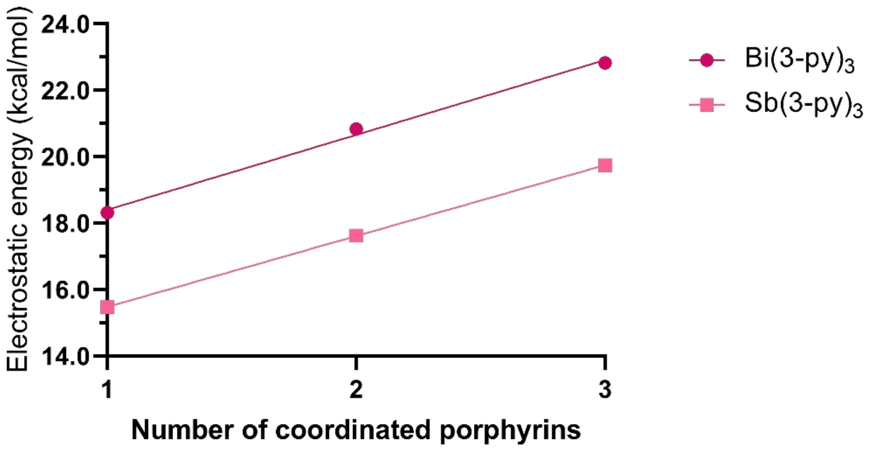

Figure S91. Plot of V_{max} of the sigma holes for compounds $Sb(3\text{-}py)_3$ and $Bi(3\text{-}py)_3$ (**1** and **2**) next to the π-electron density of a coordinated porphyrin as a function of the number of coordinated porphyrin units ZnP.

Additionally, porphyrin coordination impacts the charge located on the pnictogen atom, which attributed to its lone pair, increasing it at a rate of 1.1 kcal/mol with the attachment of each ZnP fragment (Figure S92). The R^2 values were again very good at 0.9998 for both the $Sb(3\text{-}py)_3$ and $Bi(3\text{-}py)_3$ bridgeheads.

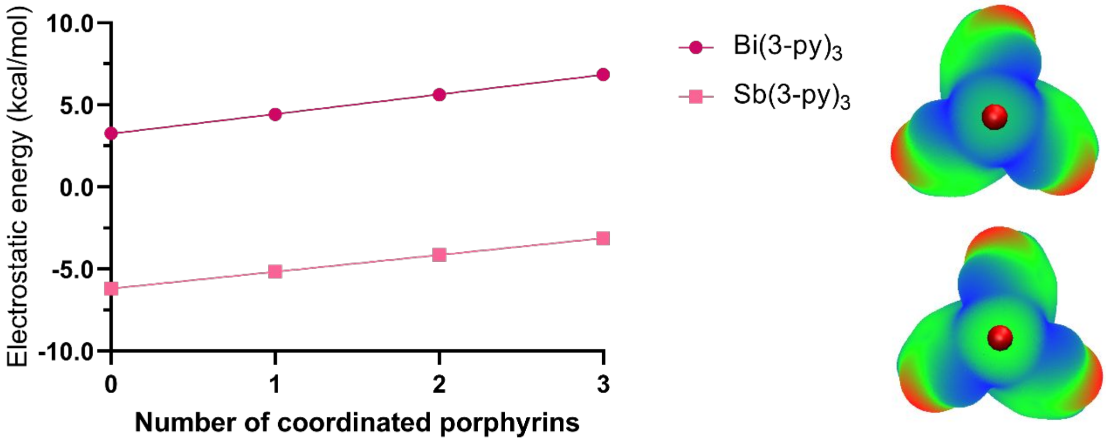

Figure S92. Plot of the electrostatic potential of the local minima of $Sb(3\text{-}py)_3$ and $Bi(3\text{-}py)_3$ (**1** and **2**) located on the pnictogen atom (pnictogen lone pair) as a function of the number of coordinated units ZnP (left). ESP maps with the key local minima attributed to lone pairs are indicated by red spheres (right).

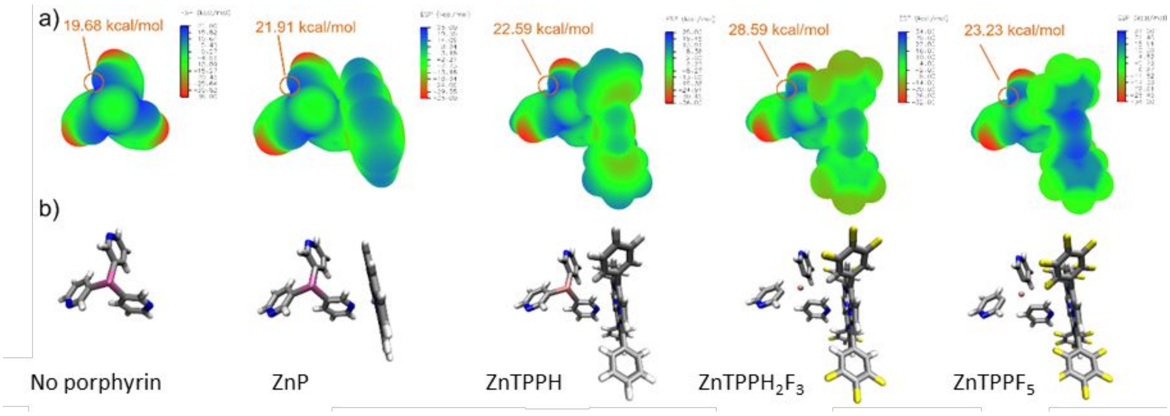

Figure S93. a) ESP maps of compound Sb(3-py)$_3$ (**1**) and its corresponding mono-adducts with the porphyrins ZnP, ZnTPPH, ZnTPPH$_2$F$_3$ and ZnTPPF$_5$ along with their color scale bars (in kcal/mol). V_{max} values are shown. b) Molecular representations of above-described species.

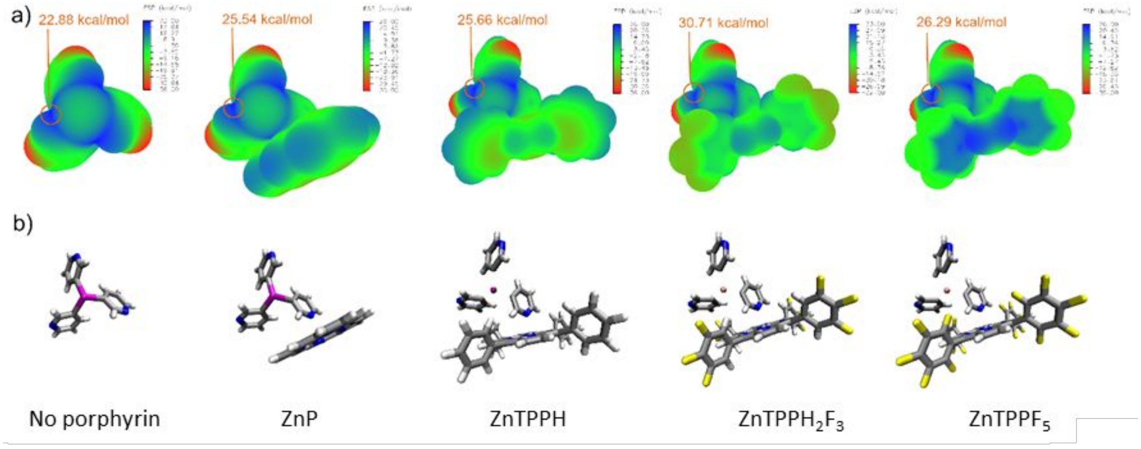

Figure S94. a) ESP maps of compound Bi(3-py)$_3$ (**2**) and its corresponding mono-adducts with the porphyrins ZnP, ZnTPPH, ZnTPPH$_2$F$_3$ and ZnTPPF$_5$, along with their color scale bars (in kcal/mol). V_{max} values are shown. b) Molecular representations of the above-described species.

Based on the ESP plots in Figures 9 and S93, as well as the trend in the electrostatic potential in Figure S92, the following conclusions can be extracted: (1) The effect of adding an unsubstituted phenyl moiety at the meso position of the porphyrin increases V_{max} negligibly (by an average of ca. 0.2 kcal/mol); (2) the substitution of three hydrogen atoms in the phenyl moiety for fluorine atoms dramatically increases V_{max} by 5 kcal/mol, deepening the sigma hole; and (3) perfluorinated aryls negatively impact the sigma hole, decreasing its V_{max} by -4.1 kcal/mol as a consequence of the pnictogen bonding of the fluorine atoms in the ortho position with the bridging atom (E = Sb, Bi). The overall sigma hole deepening effect (ΔV_{max}) for the coordination of a single porphyrin ZnTPPF$_5$ is 3.81 and 3.33 kcal/mol for Bi(3-py)$_3$ and Sb(3-py)$_3$, respectively.

Table S13. Electrostatic charges on the bridgehead atom calculated through the NBO program for the free ligands (entry 1) and the adducts formed with ZnP porphyrin (entries 2 through 4) and the adducts formed with $ZnTPPF_5$ porphyrin (entries 5 through 7).

Entry	Molecule	$Sb(3\text{-}py)_3$	$Bi(3\text{-}py)_3$
1	Free ligand	1.074	1.075
2	Ligand · $(ZnP)_1$	1.090	1.095
3	Ligand · $(ZnP)_2$	1.106	1.112
4	Ligand · $(ZnP)_3$	1.122	1.135
5	Ligand · $(ZnTPPF_5)_1$	1.104	1.115
6	Ligand · $(ZnTPPF_5)_2$	1.132	1.154
7	Ligand · $(ZnTPPF_5)_3$	1.159	1.191

Intermolecular pnictogen bond studies

The above-described computational protocol (NBO, QTAIM and ESP) was thus applied to $E(3\text{-}py)_3\cdot(ZnTPPBr)_2$ (E = Bi, Sb) semi-capsules. In these cases, the studied E···Br pnictogen bond corresponds to an intermolecular interaction between the bridgehead atom (Sb or Bi) and the bromine atom from a contiguous porphyrin pertaining to another semi-capsule, denoted here simply as *short contact* (Figure S95). Please note that intermolecular interactions are inherently more complex, and the computational model employed here represents a simplified approximation of these effects.

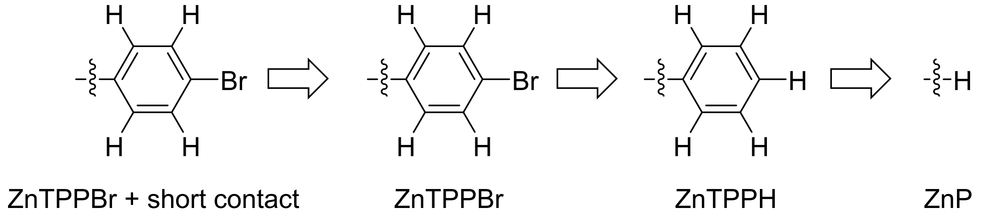

Figure S95. Simplification of the aryl substituents in the coordinated porphyrins for comparison with the $E(3\text{-}py)_3\cdot(ZnTPPBr)_2$ (E = Bi, Sb) systems using theoretical methods.

NBO analyses of **1**·ZnTPPBr and **2**·ZnTPPBr show an overlap between unoccupied σ*(E–C) orbitals and the lone pairs of the bromine atom (Figure S96 and Figure S97) of an adjacent molecule.

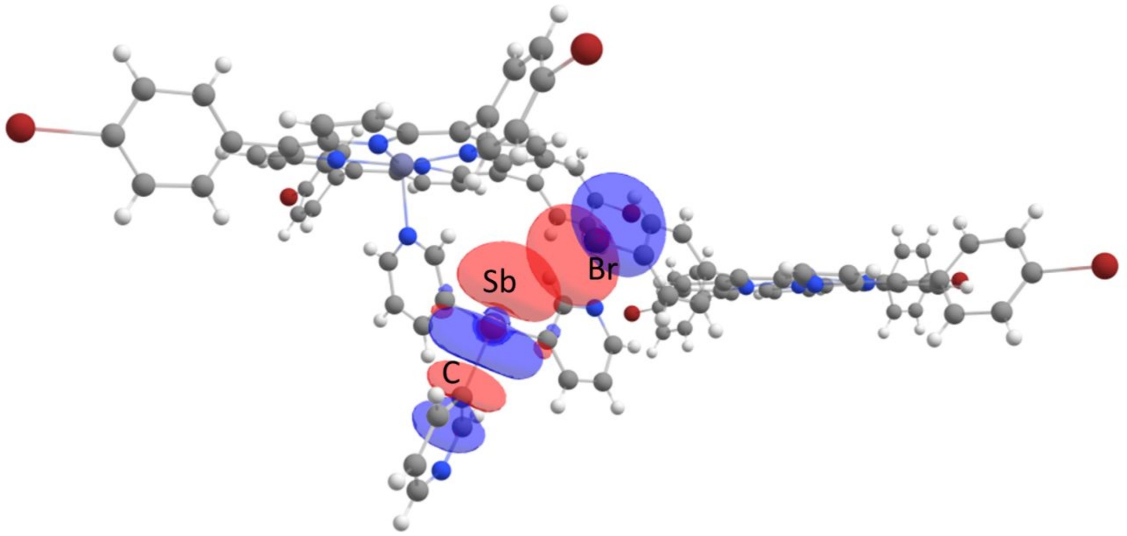

Figure S96. Representation of the NBO molecular orbitals for the intermolecular interaction between bromine and antimony in **1**·ZnTPPBr.

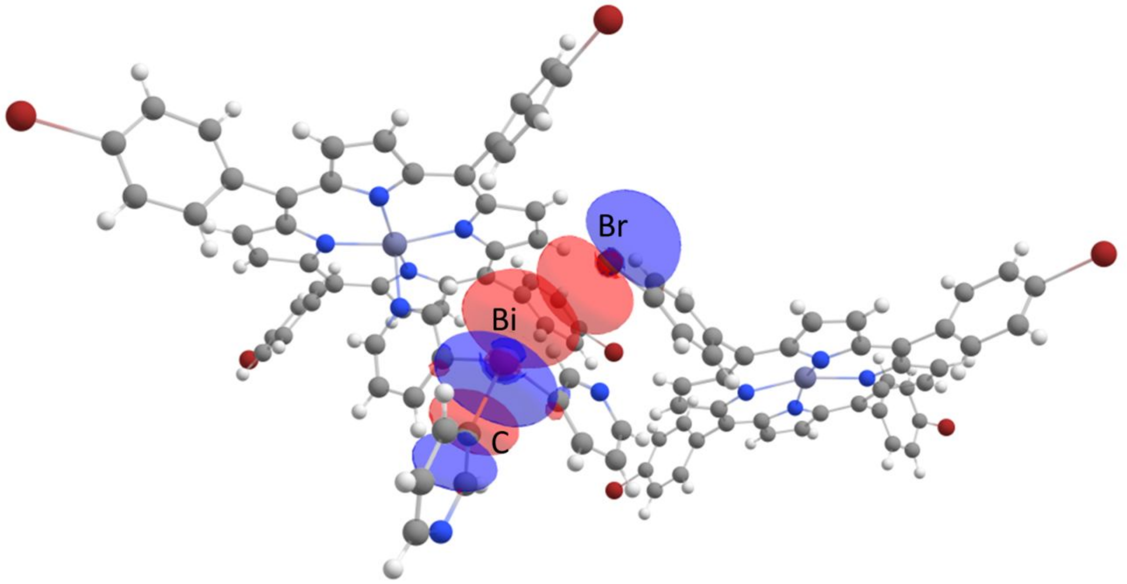

Figure S97. Representation of the NBO molecular orbitals for the intermolecular interaction between bromine and antimony in **2**·ZnTPPBr.

QTAIM analyses revealed bond critical points (BCPs) along each E···Br axis computed for the shorter pnictogen-bromine distance as measured in the solid state for both systems (Figure S98 and Figure S99), consistent with pnictogen bonding.

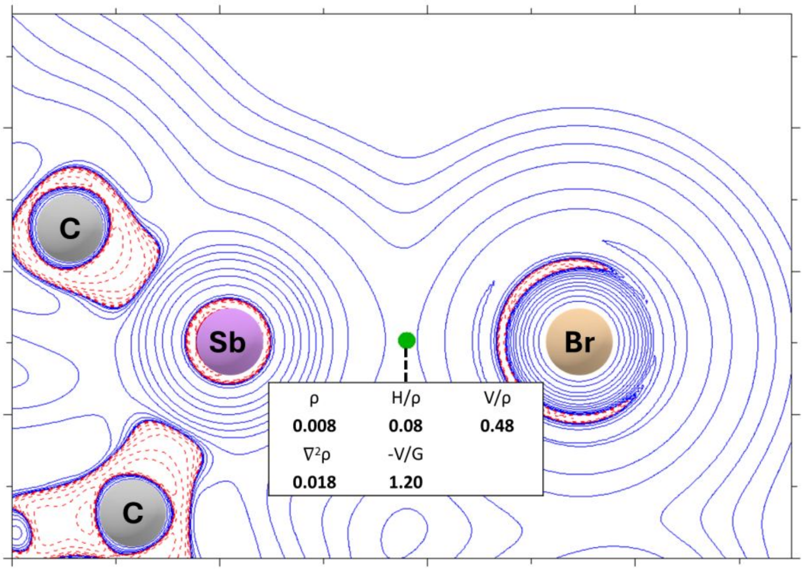

Figure S98. 2D Quantum Theory of Atoms in Molecules (QTAIM) analysis of **1**·ZnTPPBr. Contour lines show the Laplacian of the electron density ($\nabla^2\rho$) (blue: $\nabla^2\rho > 0$; red: $\nabla^2\rho < 0$). Atomic critical points (3, +3) are displayed as atom labels. Bond critical points (3, −1) are displayed as green dots. The values of the electron density (ρ), the Laplacian of the electron density ($\nabla^2\rho$), the relative energy density (*H*/ρ), the relative kinetic energy density (*V*/ρ) and the ratio of kinetic and potential energy densities (−*V*/*G*) at the bond critical point are given in atomic units (a.u.).

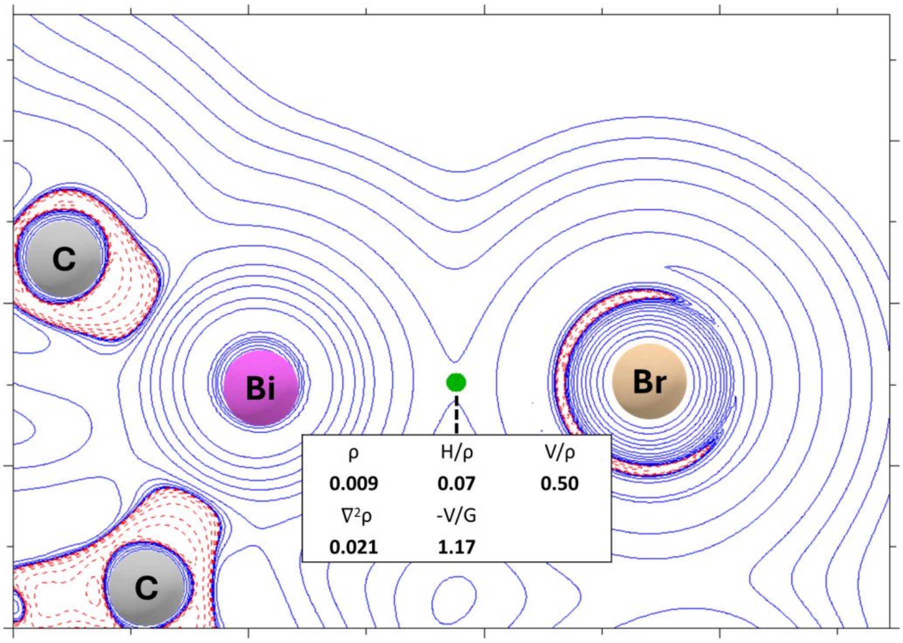

Figure S99. 2D Quantum Theory of Atoms in Molecules (QTAIM) analysis of **2**·ZnTPPBr. Contour lines show the Laplacian of the electron density ($\nabla^2\rho$) (blue: $\nabla^2\rho > 0$; red: $\nabla^2\rho < 0$). Atomic critical points (3, +3) are displayed as atom labels. Bond critical points (3, −1) are displayed as green dots. The values of the electron density (ρ), the Laplacian of the electron density ($\nabla^2\rho$), the relative energy density (*H*/ρ), the relative kinetic energy density (*V*/ρ) and the ratio of kinetic and potential energy densities (−*V*/*G*) at the bond critical point are given in atomic units (a.u.).

The obtained ESP surfaces, as well as relevant V_{max} regions, were compared to extract information about the electrostatic evolution of the targeted sigma hole according to the simplification scheme described in Figure S95 (see Figure S100 and Figure S101). The trend in charge depletion (σ-hole deepening) followed by depth reduction resulted in behavior similar to that observed for the E(3-py)$_3$·(ZnTPPF$_5$)$_3$ (E = Bi, Sb) systems (Figure S102).

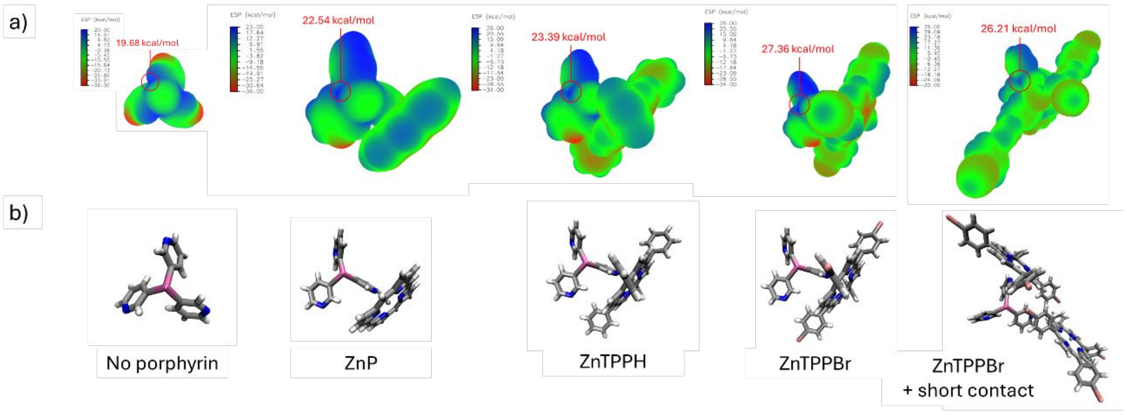

Figure S100. a) ESP maps of compound Sb(3-py)$_3$ (**1**) and its corresponding mono-adducts with the porphyrins ZnP, ZnTPPH, ZnTPPBr and ZnTPPBr + short contact along with their color scale bars (in kcal/mol). V_{max} values are shown. b) Molecular representations of above-described species.

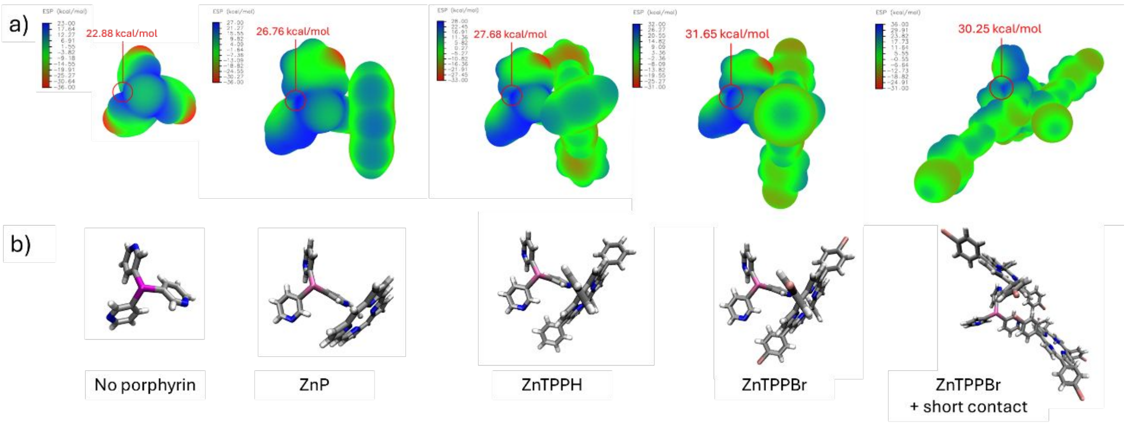

Figure S101. a) ESP maps of compound Bi(3-py)$_3$ (**2**) and its corresponding mono-adducts with the porphyrins ZnP, ZnTPPH, ZnTPPBr and ZnTPPBr + short contact along with their color scale bars (in kcal/mol). V_{max} values are shown. b) Molecular representations of above-described species.

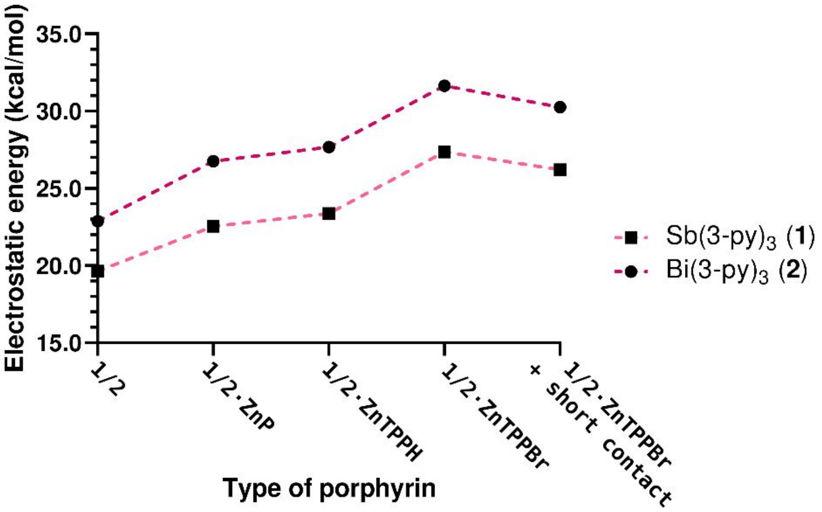

Figure S102. c) Sigma holes (V_{max}) for the mono-adducts with ZnP, ZnTPPH, ZnTPPBr and ZnTPPBr + short contact and Sb(3-py)$_3$ (light pink) and Bi(3-py)$_3$ (dark pink). As previously mentioned, 'short contact' refers to the intermolecular PnB.

Catalytic tests

Procedure for Sb-catalyzed oxidations of α-hydroxyketones.

For the kinetic experiments, the corresponding α-hydroxyketone (0.02 mmol) and the catalytic Sb species (10 mol%) were dissolved in 1 ml of $CDCl_3$ in a J. Young NMR tube under air. ^1H NMR was subsequently used to determine the yield after 24 h. No product formation was observed under the same conditions in the absence of catalyst (with or without the presence of metalloporphyrin). Dibromomethane was used as an internal reference.

References

(1) Fadda, A. A.; El-Mekawy, R. E.; El-Shafei, A.; Freeman, H. S.; Hinks, D.; El-Fedawy, M. Design, Synthesis, and Pharmacological Screening of Novel Porphyrin Derivatives. *J. Chem.* **2013**, *2013* (1). https://doi.org/10.1155/2013/340230.

(2) Liu, Y.; Guo, X.; Xiang, N.; Zhao, B.; Huang, H.; Li, H.; Shen, P.; Tan, S. Synthesis and Photovoltaic Properties of Polythiophene Stars with Porphyrin Core. *J. Mater. Chem.* **2010**, *20* (6), 1140–1146. https://doi.org/10.1039/B916935K.

(3) Peters, M. K.; Röhricht, F.; Näther, C.; Herges, R. One-Pot Approach to Chlorins, Isobacteriochlorins, Bacteriochlorins, and Pyrrocorphins. *Org. Lett.* **2018**, *20* (24), 7879–7883. https://doi.org/10.1021/acs.orglett.8b03433.

(4) Ferrero, S.; Barbero, H.; Miguel, D.; García-Rodríguez, R.; Álvarez, C. M. Dual-Tweezer Behavior of an Octapodal Pyrene Porphyrin-Based System as a Host for Fullerenes. *J. Org. Chem.* **2019**, *84* (10), 6183–6190. https://doi.org/10.1021/acs.joc.9b00362.

(5) Choudhury, A. K.; Sarkar, R.; Bhuyan, J. Histamine-Bound Magnesium Porphyrins: Diverse Coordination Modes, Inhibitory Role in Photodegradation of Chlorophyll a and Antioxidant Activity. *Dalt. Trans.* **2023**, *52* (32), 11085–11095. https://doi.org/10.1039/d3dt01309j.

(6) González López, E. J.; Palacios, Y. B.; Martinez, S. R.; Durantini, A. M.; Durantini, E. N.; Abraham, G. A.; Bongiovanni Abel, S.; Heredia, D. A. Light-Activated Antibacterial Ethylcellulose Electrospun Nanofibrous Mats Containing Fluorinated Zn(II) Porphyrin. *ACS Appl. Polym. Mater.* **2024**, *6* (13), 7691–7704. https://doi.org/10.1021/acsapm.4c01167.

(7) García-Romero, Á.; Miguel, D.; Wright, D. S.; Álvarez, C. M.; García-Rodríguez, R. Structural and Dimensional Control of Porphyrin Capsules Using Group 15 Tris(3-Pyridyl) Linkers. *Chem. Sci.* **2023**, *14* (24), 6522–6530. https://doi.org/10.1039/D3SC02151C.

(8) Macchioni, A.; Ciancaleoni, G.; Zuccaccia, C.; Zuccaccia, D. Determining Accurate Molecular Sizes in Solution through NMR Diffusion Spectroscopy. *Chem. Soc. Rev.* **2008**, *37* (3), 479–489. https://doi.org/10.1039/B615067P.

(9) Bachmann, S.; Gernert, B.; Stalke, D. Solution Structures of Alkali Metal Cyclopentadienides in THF Estimated by ECC-DOSY NMR-Spectroscopy (Incl. Software). *Chem. Commun.* **2016**, *52* (87), 12861–12864. https://doi.org/10.1039/c6cc07273a.

(10) Bachmann, S.; Neufeld, R.; Dzemski, M.; Stalke, D. New External Calibration Curves (ECCs) for the Estimation of Molecular Weights in Various Common NMR Solvents. *Chem. – A Eur. J.* **2016**, *22* (25), 8462–8465. https://doi.org/10.1002/chem.201601145.

(11) Neufeld, R.; Stalke, D. Accurate Molecular Weight Determination of Small Molecules via DOSY-NMR by Using External Calibration Curves with Normalized Diffusion Coefficients. *Chem. Sci.* **2015**, *6* (6), 3354–3364.

https://doi.org/10.1039/C5SC00670H.

(12) Liptay, W. F. J. C. Rossotti and H. Rossotti, The Determination of Stability Constants and Other Equilibrium Constants in Solution , McGraw-Hill Book Company, Inc., New York, Toronto, London 1961 (Series in Advanced Chemistry), 425 Seiten. Preis: 97 S. *Zeitschrift für Elektrochemie, Berichte der Bunsengesellschaft für Phys. Chemie* **1962**, *66* (3), 280–280. https://doi.org/10.1002/bbpc.19620660326.

(13) Ingham, K. C. On the Application of Job's Method of Continuous Variation to the Stoichiometry of Protein-Ligand Complexes. *Anal. Biochem.* **1975**, *68* (2), 660–663. https://doi.org/10.1016/0003-2697(75)90666-1.

(14) Brynn Hibbert, D.; Thordarson, P. The Death of the Job Plot, Transparency, Open Science and Online Tools, Uncertainty Estimation Methods and Other Developments in Supramolecular Chemistry Data Analysis. *Chem. Commun.* **2016**, *52* (87), 12792–12805. https://doi.org/10.1039/c6cc03888c.

(15) Ulatowski, F.; Dąbrowa, K.; Bałakier, T.; Jurczak, J. Recognizing the Limited Applicability of Job Plots in Studying Host–Guest Interactions in Supramolecular Chemistry. *J. Org. Chem.* **2016**, *81* (5), 1746–1756. https://doi.org/10.1021/acs.joc.5b02909.

(16) Thordarson, P. Determining Association Constants from Titration Experiments in Supramolecular Chemistry. *Chem. Soc. Rev.* **2011**, *40* (3), 1305–1323. https://doi.org/10.1039/C0CS00062K.

(17) Thordarson, P. Binding Constants and Their Measurement. In *Supramolecular Chemistry*; Wiley, 2012. https://doi.org/10.1002/9780470661345.smc018.

(18) Pramanik, S.; Thordarson, P.; Day, V. W.; Bowman-James, K. Oligomeric Phosphate Clusters in Macrocyclic Channels. *CrystEngComm* **2022**, *24* (46), 8047–8051. https://doi.org/10.1039/d2ce00756h.

(19) Álvarez-Llorente, N.; Stasyuk, A. J.; Diez-Varga, A.; Ferrero, S.; Solà, M.; Barbero, H.; Álvarez, C. M. Multitopic Corannulene–Porphyrin Hosts for Fullerenes: A Three-Layer Scaffold for Precisely Designed Supramolecular Ensembles. *Org. Lett.* **2025**, *27* (1), 357–362. https://doi.org/10.1021/acs.orglett.4c04385.

(20) Lu, T.; Chen, F. Multiwfn: A Multifunctional Wavefunction Analyzer. *J. Comput. Chem.* **2012**, *33* (5), 580–592. https://doi.org/10.1002/jcc.22885.

(21) Humphrey, W.; Dalke, A.; Schulten, K. VMD: Visual Molecular Dynamics. *J. Mol. Graph.* **1996**, *14* (1), 33–38. https://doi.org/10.1016/0263-7855(96)00018-5.
